# A Pilot Study on the Effects of Transcranial Direct Current Stimulation on Brain Rhythms and Entropy during Self-Paced Finger Movement using the Epoc Helmet

**DOI:** 10.3389/fnhum.2017.00201

**Published:** 2017-04-25

**Authors:** Florian C. A. A. Bodranghien, Margot Langlois Mahe, Serge Clément, Mario U. Manto

**Affiliations:** ^1^Unité d’Etude du Mouvement (UEM-GRIM), Fonds de la Recherche Scientifique, Université Libre De BruxellesBruxelles, Belgium; ^2^Haute Ecole Libre de Bruxelles Ilya Prigogine (HELB)Bruxelles, Belgium

**Keywords:** entropy, wearable EEG, spectral, direct current stimulation, sequential movements

## Abstract

Transcranial direct current stimulation (tDCS) of the cerebellum is emerging as a novel non-invasive tool to modulate the activity of the cerebellar circuitry. In a single blinded study, we applied anodal tDCS (atDCS) of the cerebellum to assess its effects on brain entropy and brain rhythms during self-paced sequential finger movements in a group of healthy volunteers. Although wearable electroencephalogram (EEG) systems cannot compete with traditional clinical/laboratory set-ups in terms of accuracy and channel density, they have now reached a sufficient maturity to envision daily life applications. Therefore, the EEG was recorded with a comfortable and easy to wear 14 channels wireless helmet (Epoc headset; electrode location was based on the 10–20 system). Cerebellar neurostimulation modified brain rhythmicity with a decrease in the delta band (electrode F3 and T8, *p* < 0.05). By contrast, our study did not show any significant change in entropy ratios and laterality coefficients (LC) after atDCS of the cerebellum in the 14 channels. The cerebellum is heavily connected with the cerebral cortex including the frontal lobes and parietal lobes via the cerebello-thalamo-cortical pathway. We propose that the effects of anodal stimulation of the cerebellar cortex upon cerebral cortical rhythms are mediated by this key-pathway. Additional studies using high-density EEG recordings and behavioral correlates are now required to confirm our findings, especially given the limited coverage of Epoc headset.

## Introduction

Transcranial direct current stimulation (tDCS) is a non-invasive neuromodulatory technique aiming to induce short to prolonged functional changes in Central Nervous System (CNS) circuits and to promote neuroplasticity (Priori et al., 2014). The cerebellum is particularly suited for application of tDCS for both anatomical and electrophysiological reasons (Grimaldi et al., 2014; Ferrucci et al., 2015). Cerebellar tDCS induces neurophysiological changes by modulating cerebellum-brain inhibition (CBI) through effects upon the cerebellar cortex (Grimaldi et al., 2016).

Anodal tDCS (atDCS) exerts an excitatory effect upon the Purkinje cell layer, unlike cathodal tDCS (ctDCS). There is currently a growing interest in the potential applications of tDCS to the cerebellum given the major contributions of the cerebellar circuitry not only in motor domain but also in the so-called cognitive operations and in the emotional processing (Koziol et al., 2014; Lupo et al., 2015). Recent studies point out that tDCS of the cerebellum is even emerging as a potential therapeutical tool to tune down symptoms in cerebellar ataxias. For instance, it has been demonstrated that a single session of anodal cerebellar tDCS transiently improves symptoms in patients with cerebellar ataxia and thus represents a promising technique for future rehabilitative approaches (Benussi et al., 2015).

Electroencephalogram (EEG) brain rhythms are markers of the cerebral activity and can be used to control a brain-computer interface (BCI). It has been shown that tDCS can modulate brain rhythms and that atDCS can significantly impact the alpha band (Spitoni et al., 2013). This modulation is particularly interesting and shows promising perspective to enhance BCI event related desynchronization/synchronization. It could enhance BCI wearable BCI application such as the one developed by Looned et al. (2014) in the upper limb for a drinking task.

Brain entropy is currently used to estimate the information conveyed in a signal and is especially applied to characterize EEG signals in disorders of the CNS, such as neurodegenerative diseases, epilepsy and coma. One typical example is the study of Abásolo et al. (2006). The authors have assessed the variations of EEG entropy in Alzheimer’s disease and have tested the hypothesis that the regularity of the patients’ EEG was higher compared to age-matched controls by computing spectral entropy and sample entropy. They found that the analysis of sample entropy could distinguish patients from control subjects (at electrodes P3, P4, O1 and O2), bringing the possibility of an early diagnosis for a very common devastating disorder in the elderly. No difference was found for spectral entropy. In the field of epilepsy, Kannathal et al. (2005) have compared different entropy estimators for the detection of seizures. Their method reached a 90% classification accuracy between control and epileptic EEG. In disorders of consciousness, entropy studies show reduced information processing in minimally-conscious-state and unresponsive-wakefulness-syndrome, in line with impaired information flow (Thul et al., 2016). Overall, entropy allows a better estimation of the information flow in the brain. However, very little is known on the EEG entropy as a neural correlate in a context of voluntary movements of the upper limbs.

In the current study, our hypothesis was that tDCS of the cerebellum modulates brain rhythms and/or entropy during self-paced sequential finger movements. The rationale to assess the effects of tDCS of the cerebellum on brain entropy and rhythms is that the cerebellar circuitry is essential in the computation of critical movement parameters such as the timing, the organization of motor sequences or the reconstruction of sequences of events to predict the future (Braitenberg, 1967; Ivry and Spencer, 2004; Molinari et al., 2008; Leggio and Molinari, 2015).

## Materials and Methods

### Participants

The participants were asked to sign a written consent, following approval of the study by the Ethical Committee of the Hôpital Erasme-ULB (Study reference: P2014/378). The following exclusion criteria were applied:

- History of neurological disease or head trauma
- Intake of psychotropic drug
- Alcohol intake in the previous 12 h
- Metal implant in the body (i.e., pace-maker)
- Skin lesion over the skull
- Pregnancy

**Figure 1:**
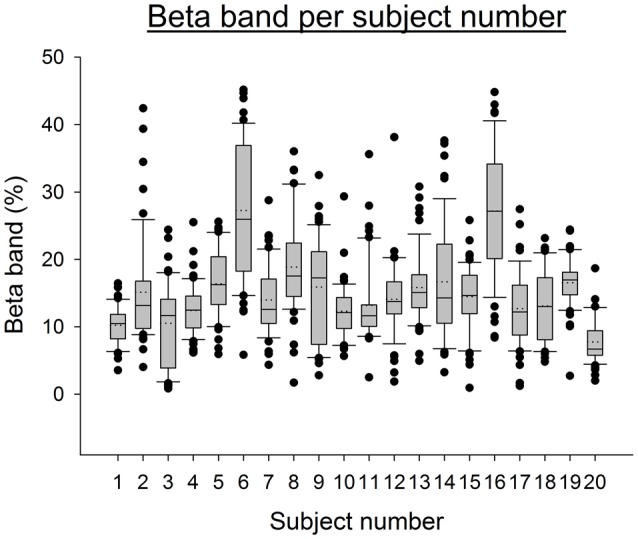
**Boxplot representing the EEG Beta band for all electrodes and conditions per subject.** The boxes correspond to the 25–75 percentiles, the continuous lines to the median values, the dotted lines to the mean values and the whiskers represent the 10 and 90 percentiles. The dots are the outlying values. Note the excess of Beta activity for subjects 6 and 16. The Beta band is expressed in percent of the complete spectrum (%). Z-scores for subject 6 and 16 are respectively: 5.86 and 5.82.

We included 20 healthy subjects (mean age ± SD: 24.0 ± 8.3 years; 12 males/8 females). In order to determine the dominant side in the upper limbs, the Edinburgh handedness inventory was used (Oldfield, 1971). The majority of subjects were right handed (*n* = 18). Due to the discovery of abnormal excess of power in the beta band during whole protocol, subjects 6 and 16 were excluded from the analysis (see Figure 1 and “EEG Analysis” Section below on data analysis, *z*-scores of average beta band for subjects 6 and 16 compared to the other subjects were respectively: 5.86 and 5.82). The results thus relate to a group of *n* = 18 subjects.

### Characteristics of the EEG Helmet and Wireless Recording

**Figure 2:**
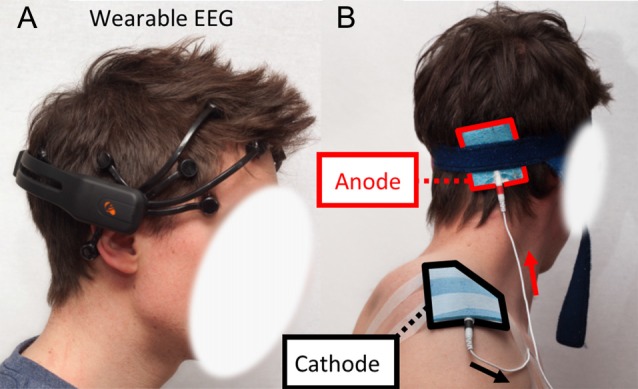
**(A)** Emotiv Epoc electroencephalogram (EEG) headset installed on the skull of a subject. **(B)** Electrodes placement for anodal transcranial Direct Current Stimulation (atDCS). Anode is located halfway between the inion and the mastoid process. Cathode is on the acromion.

The EEG helmet was a wearable, comfortable and wireless headset to avoid bulky equipment and time-consuming procedures that would prevent further potential applications in the “real world” (Berka et al., 2004; Debener et al., 2012; Callan et al., 2015). Indeed, current EEG devices used in the clinic are usually stationary, wired and cumbersome systems (Mihajlovic et al., 2015). Although it is obvious that wearable EEG systems still require developments and cannot compete with traditional clinical/laboratory set-ups in terms of accuracy and channel density (for instance, the channels FC3, FC4, C3, C4, CP3 and CP4 are not present on this helmet which a drawback for motor task. Future study should address this issue), they have now reached a sufficient maturity to envision daily life applications (Casson et al., 2010; Balanou et al., 2013; Zao et al., 2014) and most probably become one of the systems used in BCI applications in the near future. The helmet was an *Epoc* headset (emotiv, Eveleigh, NSW, Australia). This is a 14 channels wireless and unobtrusive EEG helmet now widely used in literature (Debener et al., 2012; Ekandem et al., 2012; Badcock et al., 2013; Steinhubl et al., 2015)^1^. Channels location is based on the 10–20 system. Sampling rate is 128 Hz (first acquired at 2048 Hz then down sampled), with a resolution of 0.51 μV. The bandwidth is 0.2–43 Hz. This wireless EEG also includes a two-axis gyroscope to record head rotation (sensitivity is 2.0 mV/deg/s; 1 bit corresponds to about 1.61 deg/s). The EEG helmet was placed on the subjects’ head as recommended by the manufacturer to obtain reproducible results (the two front electrodes are located two fingers above the supra-orbital ridge and the two bottom electrodes are placed on the mastoids, see Figure 2A). The continuous monitoring of impedance of each electrode was performed during the experiments (the value of the impedance is displayed online as a color code). The electrodes were regularly moisturized with physiological saline (NaCl 0.9%) using an adjustable pipette. This operation is critical to maintain the effectiveness of the helmet. Data were transferred to a laptop computer during the acquisition.

Wireless EEG recordings (duration of each recording: 3 min) were performed in a quiet room at rest, and subsequently during the execution of sequential thumb-fingers movements in the basal state, after sham session and after application of cerebellar atDCS (see below). The sequence: (a) basal recording; (b) post-sham; and (c) post-atDCS has been described earlier in a study demonstrating the modulation of long-latency EMG responses by non-invasive modulation of cerebellar activity (Grimaldi and Manto, 2013). In such settings, atDCS has an after-effect lasting at least 1 h (Nitsche and Paulus, 2000, 2001; Nitsche et al., 2003). In rodents, we have observed strong effects of atDCS of the cerebral cortex (duration of stimulation of 20 min) on CBI with changes lasting more than 2 h (Manto et al., 2011). Therefore, sham session has to be administered before the actual stimulation.

For the first recording *at rest*, subjects were instructed not to move the eyes/the head/the limbs, to keep the eyes closed, to avoid jaw movements and deep breathing, and to remain calm during the whole recording. Subjects were also asked to remain silent during EEG recordings. The second recording was the *basal condition* during the execution of self-paced movements. Subsequently, the sham stimulation was applied during 20 min. Then the subject was instructed to perform again the finger movements (third recording: *post-sham)*. Afterwards, atDCS of the cerebellum was applied during 20 min, followed by the last EEG recording (fourth recording: *post-atDCS*) during sequential finger movements. The instruction to avoid movements of the eyes/the head/the jaw and to avoid deep breathing was repeated before each recording.

### Self-Paced Sequential Finger Movements

Subjects were comfortably seated in an armchair, with their elbows on the armrests. The dominant forearm remained in the vertical position during the task and the hand was recorded on a video to check that correct sequential finger movements were performed. The task was a self-paced sequential thumb to fingers opposition movement (between the thumb and the four other fingers) at a slow and comfortable speed as reported earlier in a study comparing motor execution and mental imagery (Sauvage et al., 2011).

### tDCS

A tDCS stimulator (model: Constant Current Generator—RES 4.50 mA, TCT Research, Hong Kong) was used. Sham sessions consisted of a 30 s ramp-up from 0 mA to 1.5 mA followed by a 30 s ramp down to 0 mA. No current was delivered during the following 19 min. Sham session gives to the subject the feeling of being stimulated but does not have any physiological effect (Gandiga et al., 2006; Nitsche et al., 2008). For the active stimulation sessions, the current was set to 1.5 mA and the duration to 20 min (including a 30 s ramp up at the beginning) as reported earlier (Grimaldi and Manto, 2013). Subjects were single-blinded: they were not aware if sham or actual stimulation was applied. The anode (size: 5 × 6 cm) and the cathode (size: 9 × 7 cm) consisted in sponge electrodes with conductive rubber inset. The anode was located halfway from the subject’s mastoid process and the inion (Figure 2B) on the dominant side (Grimaldi and Manto, 2013). The cathode was located on the subject’s acromion. This electrodes’ setting ensures stimulation of the lateral cerebellar cortex (Rampersad et al., 2014).

## EEG Analysis

**Figure 3:**
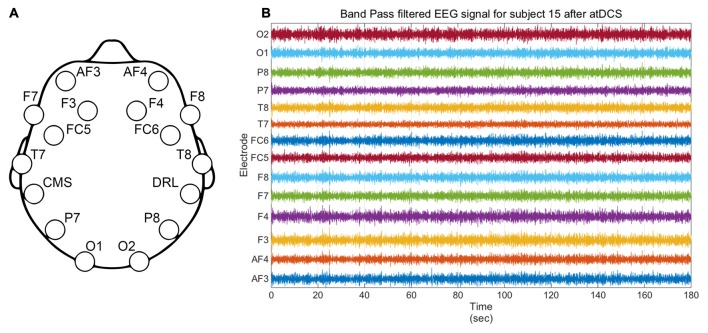
**(A)** Emotiv *Epoc* EEG headset electrodes location. CMS and DRL respectively stand for Common Mode Sense and Driven Right Leg and are used for reference and noise cancellation. **(B)** Illustrative filtered EEG signal from all electrodes for subject 15 for the active stimulation condition.

EEG signals were analyzed using Matlab (release 2014b, The MathWorks, Inc., Natick, MA, USA). First, the average offset was removed for every EEG electrode and the signal was band pass filtered using a 4th order Butterworth filter with cutoff frequencies of 1 and 50 Hz (see Figure 3). Then the signal was split into 2-s windows. Visual inspection of EEG signals did not reveal contamination by eye-blinks or muscle bursts and therefore no other artifact rejection technique was applied. Fast Fourier Transform (FFT) was used to extract the Power Spectral Density (PSD) of the signal. We applied a Hanning Window and a zero padding (equal to the next power of 2 of the window length plus 2^9^; this is a general practice as computation time is not an issue in this study) to increase accuracy (McNames, 2013).

The following quantities were computed for every channel on both the PSD averaged across all the time windows and the PSD computed from the complete signal. The Power Spectral Entropy (PSE) was computed using the following formula (Freeman and Quiroga, 2013):

(1) $$E=-\sum_{k=1}^{N}p_{k}log_{2}p_{k}$$

where *p*_k_ is the PSD of the kth frequency divided by the sum of the complete PSD vector and *N* is the number of frequency bins between the two cut frequencies of the band pass filter (*p*_k_ corresponds to the ratio of the kth frequency bin in the spectrum). This ratio ranges from 0 when only one frequency bin has a probability of 1 to log_2_ *N* for an equal frequency distribution (white noise).

The Kullback-Leibler Divergence (KLD) was also computed using the following formula (Freeman and Quiroga, 2013):

(2) $$KLD=\sum_{k=1}^{N}p_{k}log_{2}\frac{p_{k}}{q_{k}}$$

where *q*_k_ is the same as *p*_k_ but computed for the rest or basal condition only (both rest and basal reference have been used in this study). This ratio is always positive and equals zero for two similar probability distributions. It gets larger the further the distributions are.

From the power spectra, the following frequency bands were computed using a trapezoidal integration: Delta (1–4 Hz), Theta (4–8 Hz), Alpha (8–12 Hz), Beta (12–30 Hz) and Gamma (30–45 Hz). The integral between the frequency boundaries was divided by the integral of the complete spectra (1–45 Hz) and converted to percent. We also extracted the following ratios of frequency bands integrals: Theta/Delta, Alpha/Delta, Beta/Delta, Gamma/Delta, Beta/Alpha, Gamma/Alpha and Beta^2^/Alpha. The frequency bands were also computed using the rest session as a common reference. The band integrals were all divided by the integrals of the complete spectra from the resting state condition (rest referenced frequency bands).

From these frequency ratios, the Laterality Coefficient (LC ratio) was computed using the following formula:

(3) $$LC=\frac{C-I}{C+I}$$

where *C* is Beta/Alpha ratio of the contralateral limb and *I* is the Beta/Alpha ratio of the ipsilateral limb.

It should be pointed out that this is not the first study on frequency bands computed from EEG signals collected with the Epoc headset. In particular, this headset has been used to assess the neurological and cardiovascular responses during meditation (Steinhubl et al., 2015). The authors used Matlab to compute the power produced in five frequency bands and to identify the changes occurring during meditation. The same headset has also been used to assess the brain response to marketing stimuli (Khushaba et al., 2013). Furthermore, a study by Wang et al. (2011) has used the EEG signals collected from this headset to compute frequency bands ratio (Theta/Beta ratio) and establish a fractal dimension based neurofeedback in serious games. This ratio is a convenient technique to show an increase or decrease in the PSD frequencies of the signal. We used a similar approach.

## Statistical Procedures

Statistical analysis was performed using Matlab and Sigmaplot^®^ (Jandel Scientific, Germany). Descriptive statistics were computed. Mean, median, quartiles and SD values were extracted. Data normality was assessed using the Shapiro-Wilk test on mean normalized and variance-standardized data (data mean was subtracted and data was divided by variance). To test the difference in ratio distribution between conditions, an analysis of variance (ANOVA) on ranks was performed using a Kruskal-Wallis test. Normal data group of equal variance were also tested using a one-way ANOVA. Multi-comparison testing was done using the Student–Newman–Keuls (SNK) method. We took into account the hand dominance (two subjects were left-handed) to average EEG parameters (inversion of channels). Statistical significance was set at 0.05.

## Results

**Figure 4:**
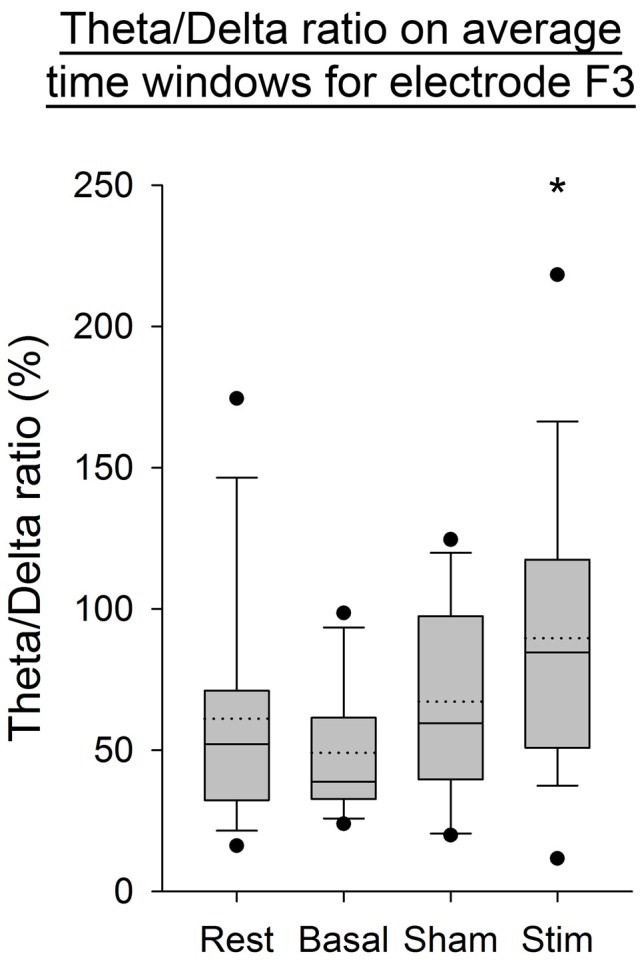
**EEG Theta/Delta ratio for the F3 electrode, from the average of power spectral density (PSD) over time windows for all subjects per condition.** The boxes correspond to the 25–75 percentiles, the continuous lines are the median values, the dotted lines are the mean values and the whiskers represent the 10 and 90 percentiles. The dots are the outlying values. Note the increase for the active stimulation condition. The Theta/Delta ratio is expressed in percent (%), *p < 0.05 as compared to the three other conditions.

**Figure 5:**
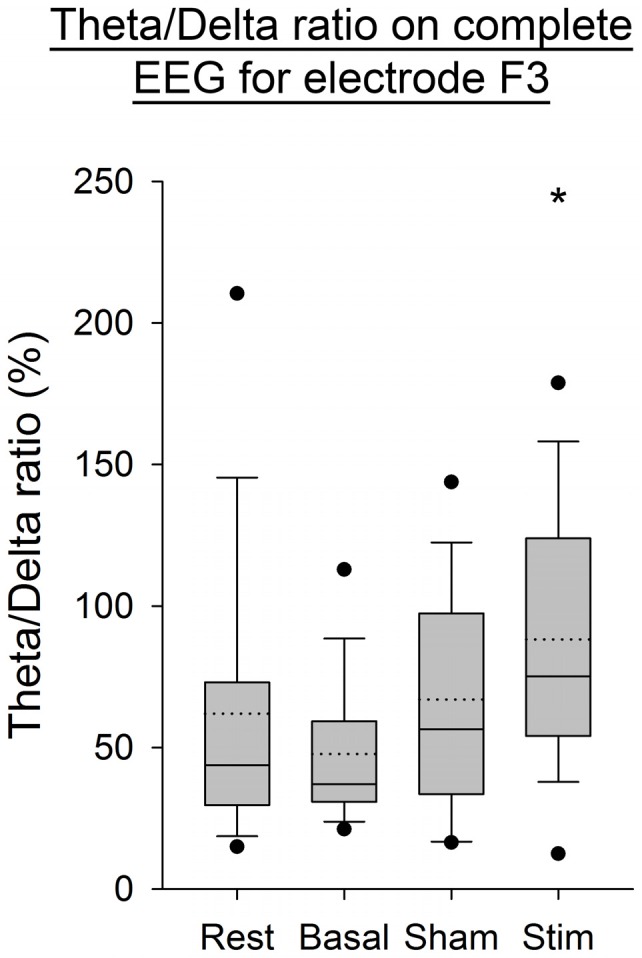
**EEG Theta/Delta ratio (complete EEG signal) for the F3 electrode for all subjects per condition.** The boxes correspond to the 25–75 percentiles, the continuous lines are the median values, the dotted lines are the mean values and the whiskers represent the 10 and 90 percentiles. The dots are the outlying values. Note the increase for the active stimulation condition. The Theta/Delta ratio is expressed in percent (%), *p < 0.05 as compared to the three other conditions.

As illustrated in Figure 4, an increase in the Theta/Delta ratio computed from the average of PSD over time windows was observed after application of atDCS over the cerebellum compared to the three other conditions. This was statistically significant for the electrode F3, contralaterally to the cerebellar stimulation (*p* < 0.05). The same ratio computed on the complete EEG signal showed the same increase on electrode F3 (*p* < 0.05, Figure 5).

**Figure 6:**
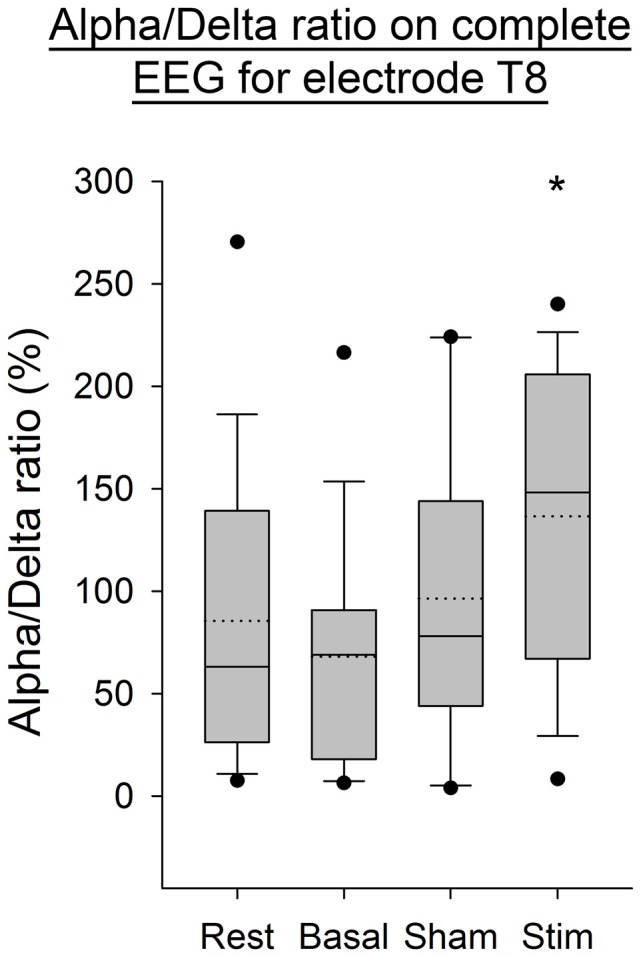
**EEG Alpha/Delta ratio (complete EEG signal) for the T8 electrode for all subjects per condition.** The boxes correspond to the 25–75 percentiles, the continuous lines are the median values, the dotted lines are the mean values and the whiskers represent the 10 and 90 percentiles. The dots are the outlying values. Note the increase for the stimulation condition. The Alpha/Delta ratio is expressed in percent (%), *p < 0.05 as compared to the three other conditions.

The Alpha/Delta ratio computed on the complete EEG signal, showed a significant increase after stimulation for the electrode T8 (*p* < 0.05, Figure 6) compared to the three other conditions. The normality of data and the equal variance were respected. A one-way ANOVA showed that only data of the basal condition were statistically different compared to data of the post-atDCS condition (*p* < 0.05).

**Figure 7:**
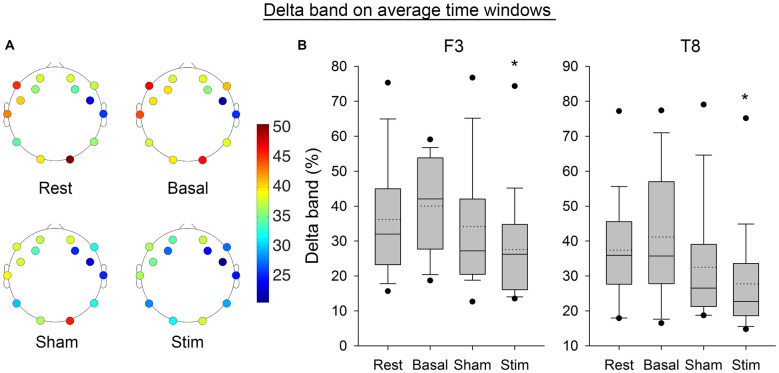
**(A)** Average EEG Delta band for all subjects per electrode and condition. **(B)** Boxplot representing the same band for the electrode F3 and T8, from the average of PSD over time windows for all subjects per condition. The boxes correspond to the 25–75 percentiles, the continuous lines are the median values, the dotted lines are the mean values and the whiskers represent the 10 and 90 percentiles. The dots are the outlying values. Note the decrease for the active stimulation condition. The Delta band is expressed in percent of the complete spectrum (%), *p < 0.05 as compared to the three other conditions.

**Figure 8:**
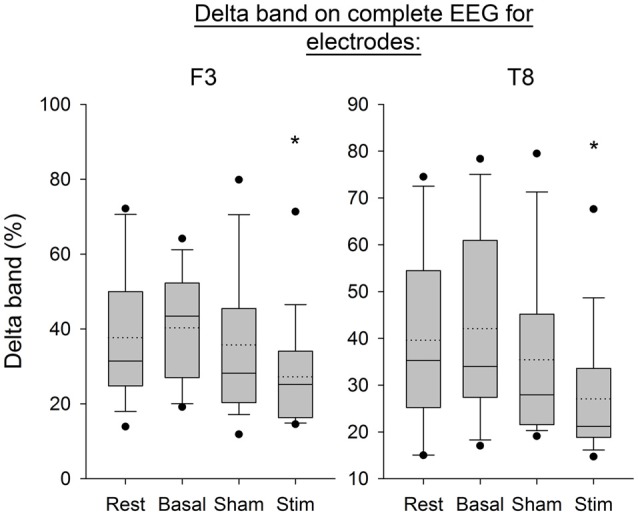
**EEG Delta band on complete signal for the electrodes F3 and T8 for all subjects per condition.** The boxes correspond to the 25–75 percentiles, the continuous lines are the median values, the dotted lines are the mean values and the whiskers represent the 10 and 90 percentiles. The dots are the outlying values. Note the decrease for the active stimulation condition. The Delta band is expressed in percent (%), *p < 0.05 as compared to the three other conditions.

Figure 7 shows the Delta frequency band computed from the average of PSD over time windows for the electrodes F3 and T8. The SNK showed a significant decrease in the frequency band after stimulation for both electrodes (*p* < 0.05) compared to the three previous conditions. Figure 8 shows the same band computed on the complete EEG signal. The same results were found with a significant decrease in Delta band after atDCS (*p* < 0.05 for electrodes F3 and T8).

**Figure 9:**
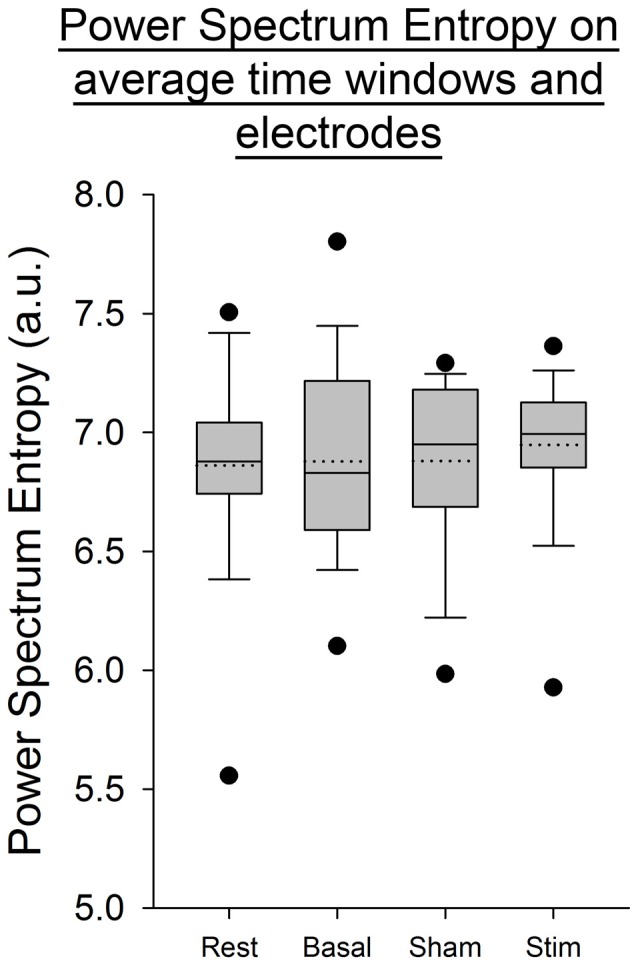
**Power spectrum entropy (PSE) from the average of PSD over electrodes and time windows for all subjects per condition.** The boxes correspond to the 25–75 percentiles, the continuous lines are the median values, the dotted lines are the mean values and the whiskers represent the 10 and 90 percentiles. The dots are the outlying values. Note the unchanged values of PSE across conditions. PSE is expressed in arbitrary units (a.u.).

**Figure 10:**
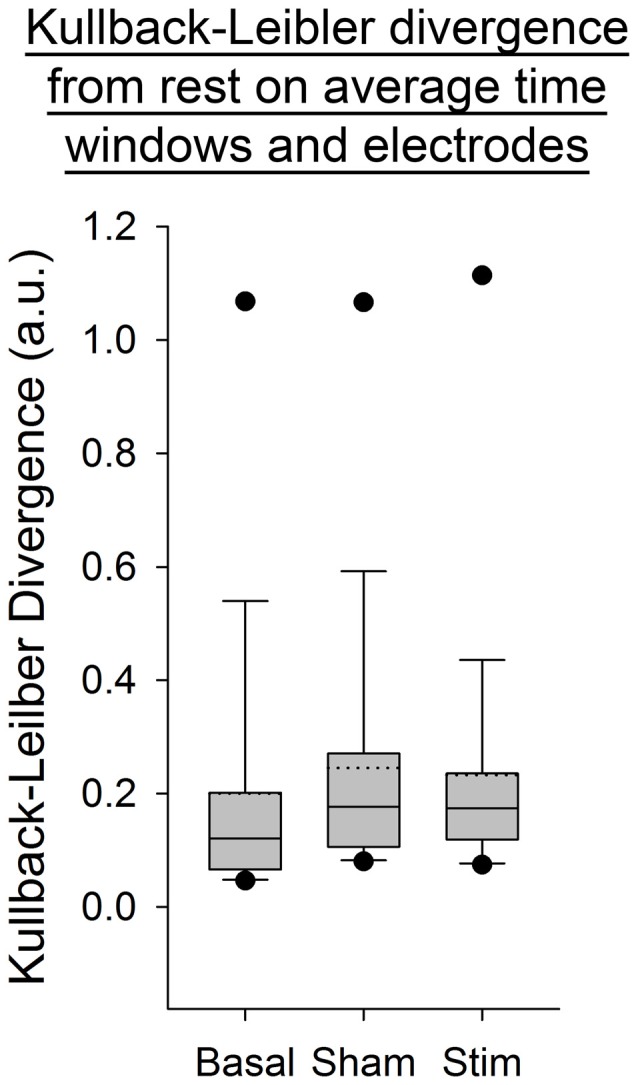
**Kullback-Leibler Divergencey (KLD) from the average of PSD over electrodes and time windows for all subjects per condition.** The boxes correspond to the 25–75 percentiles, the continuous lines are the median values, the dotted lines are the mean values and the whiskers represent the 10 and 90 percentiles. The dots are the outlying values. Note the unchanged values of KLD across conditions. KLD is expressed in arbitrary units (a.u.).

**Figure 11:**
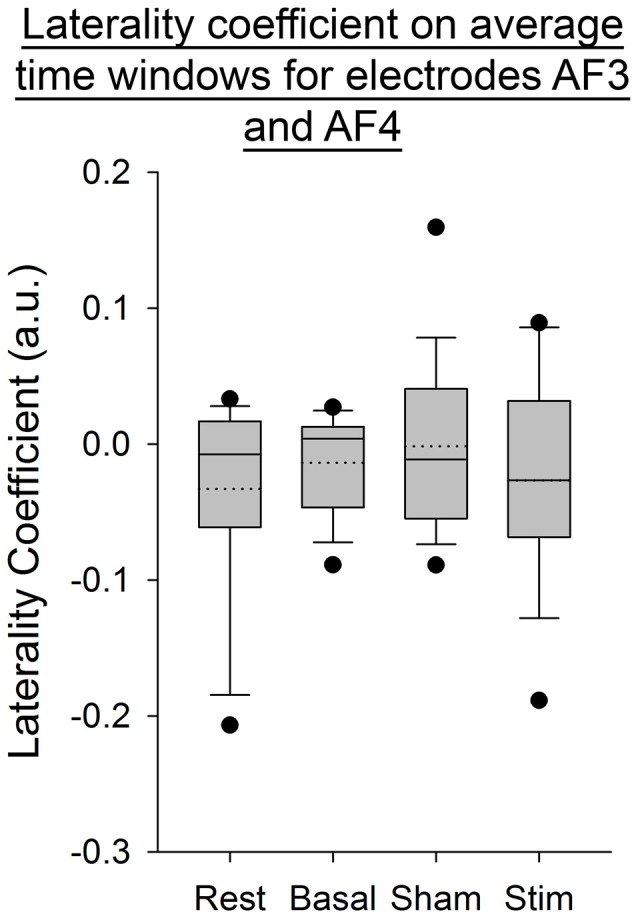
**Laterality coefficient (LC) between electrodes AF3 and AF4, from the average of PSD over time windows for all subjects per condition.** The boxes correspond to the 25–75 percentiles, the continuous lines are the median values, the dotted lines are the mean values and the whiskers represent the 10 and 90 percentiles. The dots are the outlying values. Note the unchanged values of LC across conditions. LC is expressed in arbitrary units (a.u.).

For all the other ratios, frequency bands and entropies, we did not find any difference between conditions (*p* > 0.05; Figures 9–11). Figure 9 shows the PSE from the average of PSD over all channels and time windows. Figure 10 shows the KLD referenced to the resting state condition averaged for all time windows and EEG channels. Figure 11 shows the LC ratio between the electrodes AF3 and AF4 from the average of PSD over time windows; atDCS did not impact on these ratios^2^.

## Discussion

Our findings show a decrease in the Delta band for the electrodes F3 and T8 during self-paced finger movements. By contrast, EEG PSE and KLD showed no statistically significant change after atDCS, suggesting that the cerebellar cortex could not be a major modulator of brain entropy during self-paced sequential finger movements. The LC ratio also did not show any significant change, indicating that atDCS does not bring any significant lateralized modification including in the Beta/Alpha ratio in this study and electrode configuration. Also the fact that both ratios of entropy (PSE and KLD) remain unaffected by atDCS suggests that this non-invasive neurostimulation method does not bring brain rhythms in a more disordered state at our electrodes locations.

We have excluded from the analysis two subjects due to abnormal excess of power in the beta band. High level of beta power can be observed in anxiety states (Beran, 2012), after intake of drugs such as benzodiazepines or barbiturates (Blume, 2006), after alcohol intake and is also common in children suffering from hyperactivity (Clarke et al., 2013). Recently, an excess of beta activity has also been observed in case of conduct disorder and psychopathic traits (Calzada-Reyes et al., 2016). Intake of psychotropic drug was denied by our subjects and thus the origin of abnormal beta band for these two subjects remains unclear. The excess of high frequency oscillations likely reflects an imbalance in the excitation-inhibition homeostasis in the cerebral cortex (Orekhova et al., 2007). Given the key-role of high frequency EEG rhythms for both perceptual and cognitive processes, probably genetically determined abnormalities in the neuronal mechanisms generating high frequency EEG rhythms have been incriminated (Orekhova et al., 2007).

It is important to point out that this study has several limitations. First, although the EEG helmet is easy to wear and is very comfortable, it has a low electrode density and a small bandwidth compared to more conventional laboratory grade EEG systems. Therefore, a study comparing the results obtained with our wearable EEG device and a conventional EEG should be carried out to confirm our findings. Second, atDCS was applied over one cerebellar hemisphere and not over the entire cerebellar cortex. Third, we applied one current intensity (1.5 mA) and we did not assess the dose-response. Fourth, we did not administer ctDCS to compare with atDCS and we did not repeat the administration of atDCS over several days. Finally, our study was single blinded. However, regarding this latter limitation, it should be emphasized that our subjects could not distinguish the sham vs. anodal stimulation.

Further research with a conventional high-density EEG equipment and behavioral correlates should be undertaken to establish the effects of atDCS during sequential finger movements. Our study did not include dedicated measurements of the kinematic profiles of finger flexion/extension movements. In terms of behavioral correlates, the cortico-muscular coherence should be studied to assess the coupling between EEG signals and voluntary muscle discharges (Larsen et al., 2016; Zheng et al., 2016).

This is the first study in human showing that atDCS of the cerebellum tunes brain rhythmicity during self-paced sequential finger movements. The anatomo-physiological substratum of these changes could be the cerebello-thalamo-cortical pathways (Strick et al., 2009) In particular, stimulation of the cerebellar cortex modulates the activity of dentate nucleus which is heavily and diffusely connected with the cerebral cortex (Dum and Strick, 2003). The thalamus is a central node for the relay and processing of sensitive and motor signals. Generation of rhythmic activity occurs through the interaction of stereotyped patterns of connectivity together with intrinsic membrane and synaptic properties (McCormick et al., 2015). Indirect modulation of the thalamic nuclei via the cerebello-thalamic projections could modify the general brain dynamics and redistribute its rhythms. Furthermore, it is now established that the cerebellum is anatomically connected with basal ganglia in reciprocal loops (Shakkottai et al., 2017). Therefore, atDCS of the cerebellum could impact directly on the slow frequency waves generated within basal ganglia to allow coherence in the high frequency bands across the cortico-basal ganglia network (López-Azcárate et al., 2013). Such mechanism is suggested to contribute to the fine-tuning of the timing of synchronization events across different structures in the brain.

We found an effect of unilateral atDCS upon the EEG activity of both cerebral hemispheres. What could be the anatomical substrate? Not only the corpus callosum heavily links the two cerebral hemispheres, but also the parallel fibers within the cerebellar cortex cross the midline and allow a direct anatomical communication between cerebellar hemispheres (Bastian et al., 1998). Motor but also cognitive operations such as executive demands are known to be associated with bilateral cerebellar activations in fMRI paradigms (Küper et al., 2016). These bilateral activations are subserved by numerous loops between cerebral, cerebellar and brainstem nodes to ensure the anatomical connectivity between both cerebral hemispheres.

AtDCS of the cerebellum tunes fundamental brain rhythms. This might have both fundamental and clinical implications. Zhang et al. (2013) showed that an occlusion of the middle cerebral artery in rodents induces a decrease in the alpha/delta ratio and is associated with a loss of motor control. Application of atDCS to the cerebellum could prevent this effect. This could be tested at a very early stage of a stroke in human. Also the modulation of brain rhythms induced by atDCS of the cerebellum could be used in the future to enhance the performance of wearable and multimodal BCIs aiming to assist neurological patients. Indeed, BCI inefficiency remains currently a major challenge (Zhang et al., 2015).

## Conclusion

We demonstrate that atDCS of the cerebellum exerts an effect in terms of brain rhythmicity without affecting brain entropy. We suggest that atDCS of the cerebellum should be assessed in neurological disorders characterized by an excess of low frequencies EEG activity. We also suggest that there is a need to include behavioral measurements in addition to conventional high-density EEG measurements covering also sensorimotor areas in future studies. Our data do not support potential applications of atDCS of the cerebellum to modify brain entropy.

## Presentation of Results

Part of the results were presented at the Seventh International Symposium of the Society for Cerebellar Research, Brussels, Belgium (May 2015).

## Author Contributions

All authors contributed equally to this research work.

## Conflict of Interest Statement

The authors declare that the research was conducted in the absence of any commercial or financial relationships that could be construed as a potential conflict of interest.

## Footnotes

^1^For instance, Grummett et al. (2015) made a comparative study of different wireless EEG systems including the Epoc and showed that, even with limitations, these devices were suitable for research paradigms. One example of practical use of this helmet to assist upper limb movements on the basis of EEG signals was provided by Looned et al. (2014).

^2^Details of the procedures and results are available as Supplementary Material.
