# Supplementary figures and images for: A Pilot Study on the Effects of Transcranial Direct Current Stimulation on Brain Rhythms and Entropy during Self-Paced Finger Movement using the Epoc Helmet (part 2 of 2)
Source: Front Hum Neurosci. 2017 Apr 25;11:201. doi: 10.3389/fnhum.2017.00201 (PMC5408787; doi:10.3389/fnhum.2017.00201)

**Gamma/Delta ratio on average  
PSD windows for electrode: FC5**

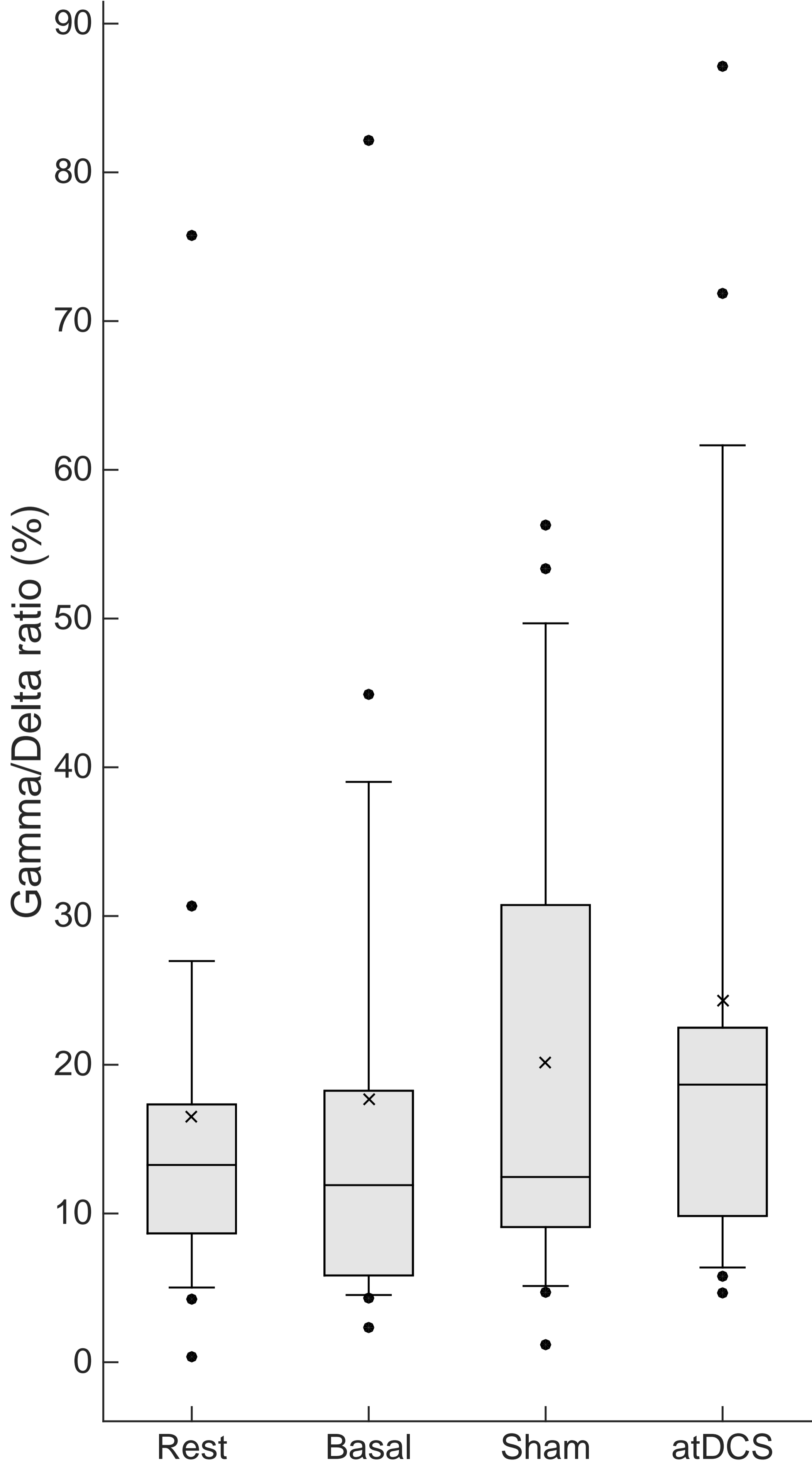

Supplement: Supplementary file 1 [file Data_Sheet_1.zip › Complementary_results/Band_ratios_average_PSD_windows/Gamma_Delta/Gamma-Delta_mean-win_FC5.pdf]

**Gamma/Delta ratio on average  
PSD windows for electrode: FC6**

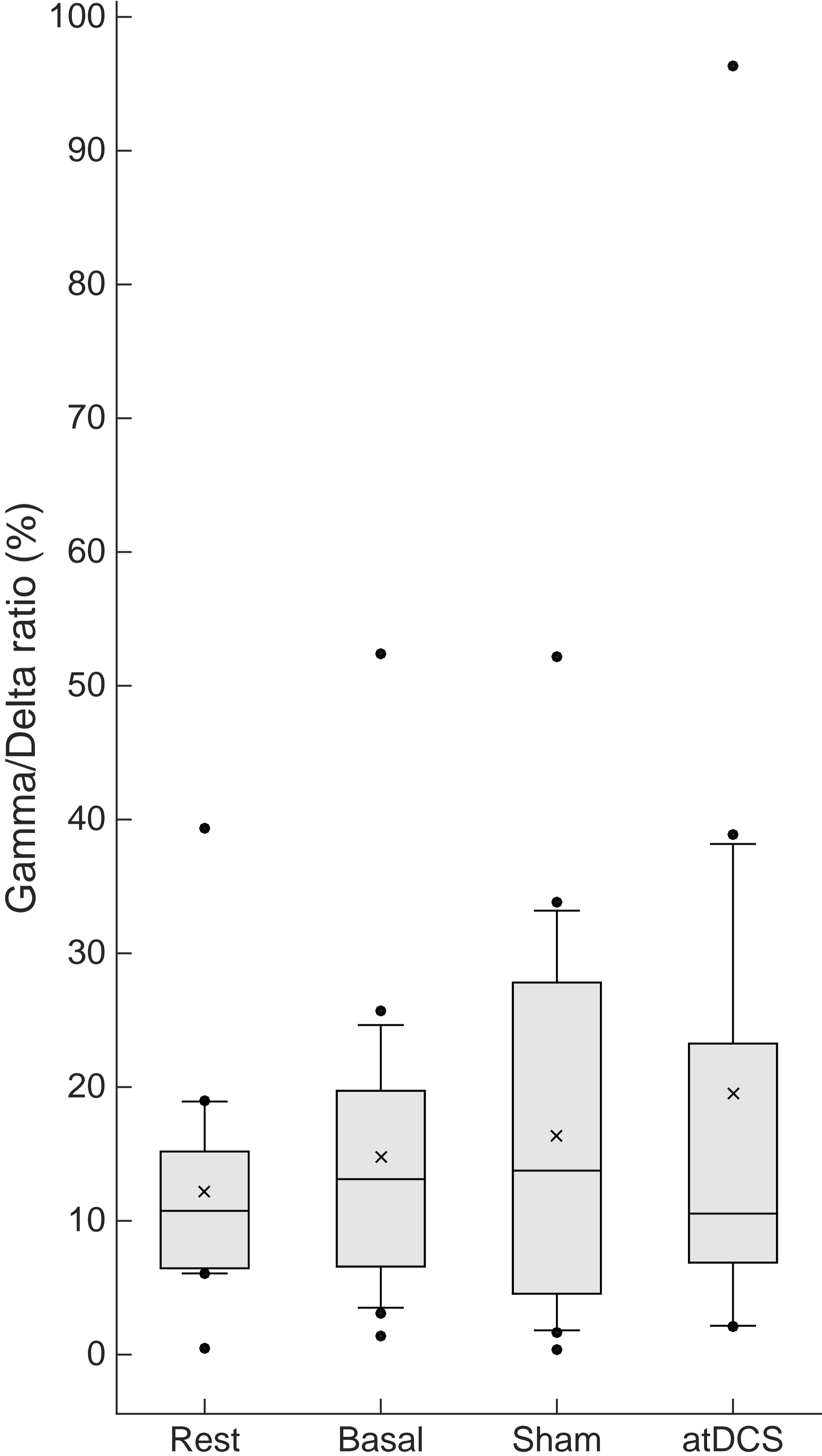

Supplement: Supplementary file 1 [file Data_Sheet_1.zip › Complementary_results/Band_ratios_average_PSD_windows/Gamma_Delta/Gamma-Delta_mean-win_FC6.pdf]

# Gamma/Delta ratio on average PSD windows for electrode: O1

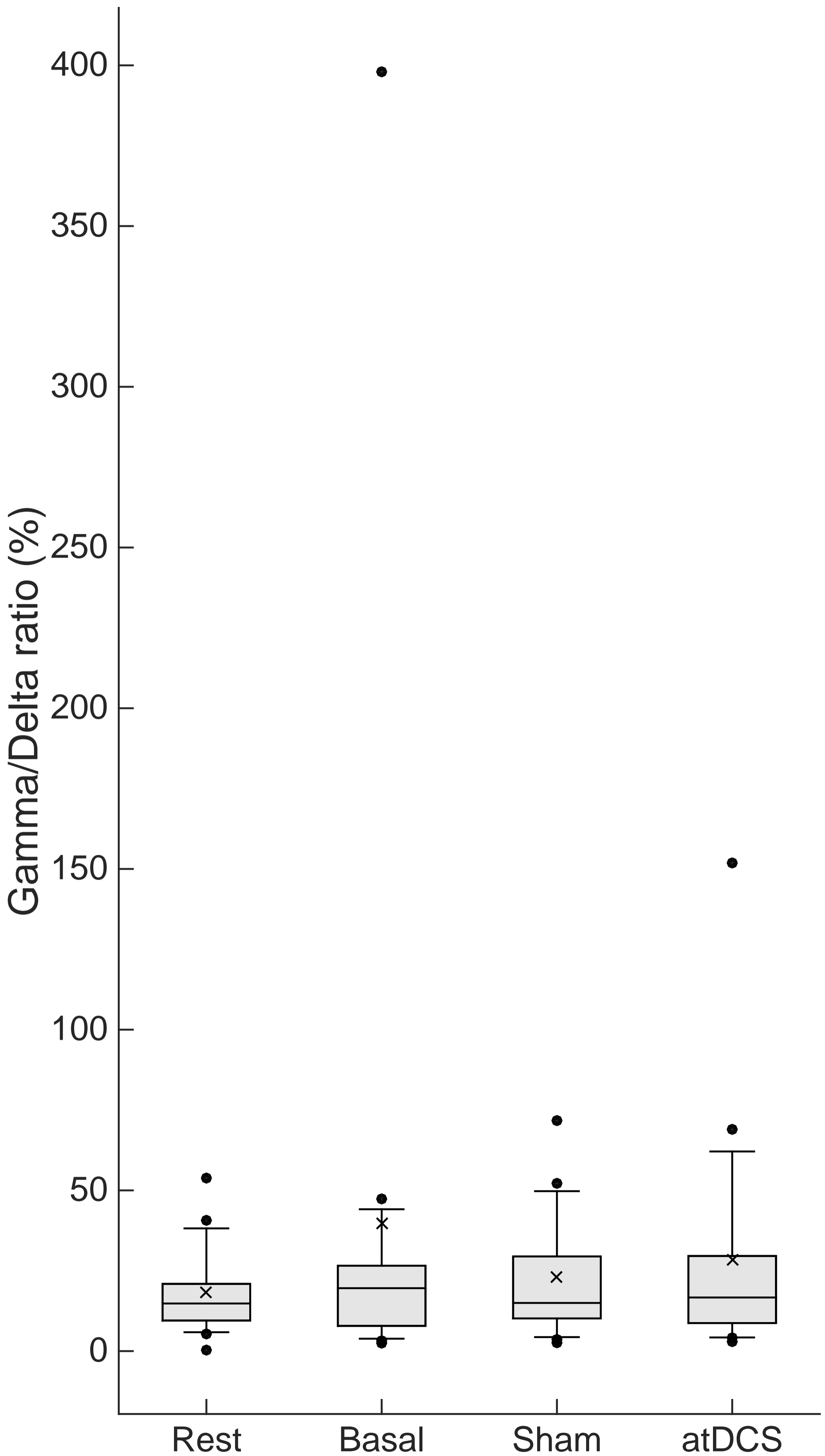

Supplement: Supplementary file 1 [file Data_Sheet_1.zip › Complementary_results/Band_ratios_average_PSD_windows/Gamma_Delta/Gamma-Delta_mean-win_O1.pdf]

# Gamma/Delta ratio on average PSD windows for electrode: O2

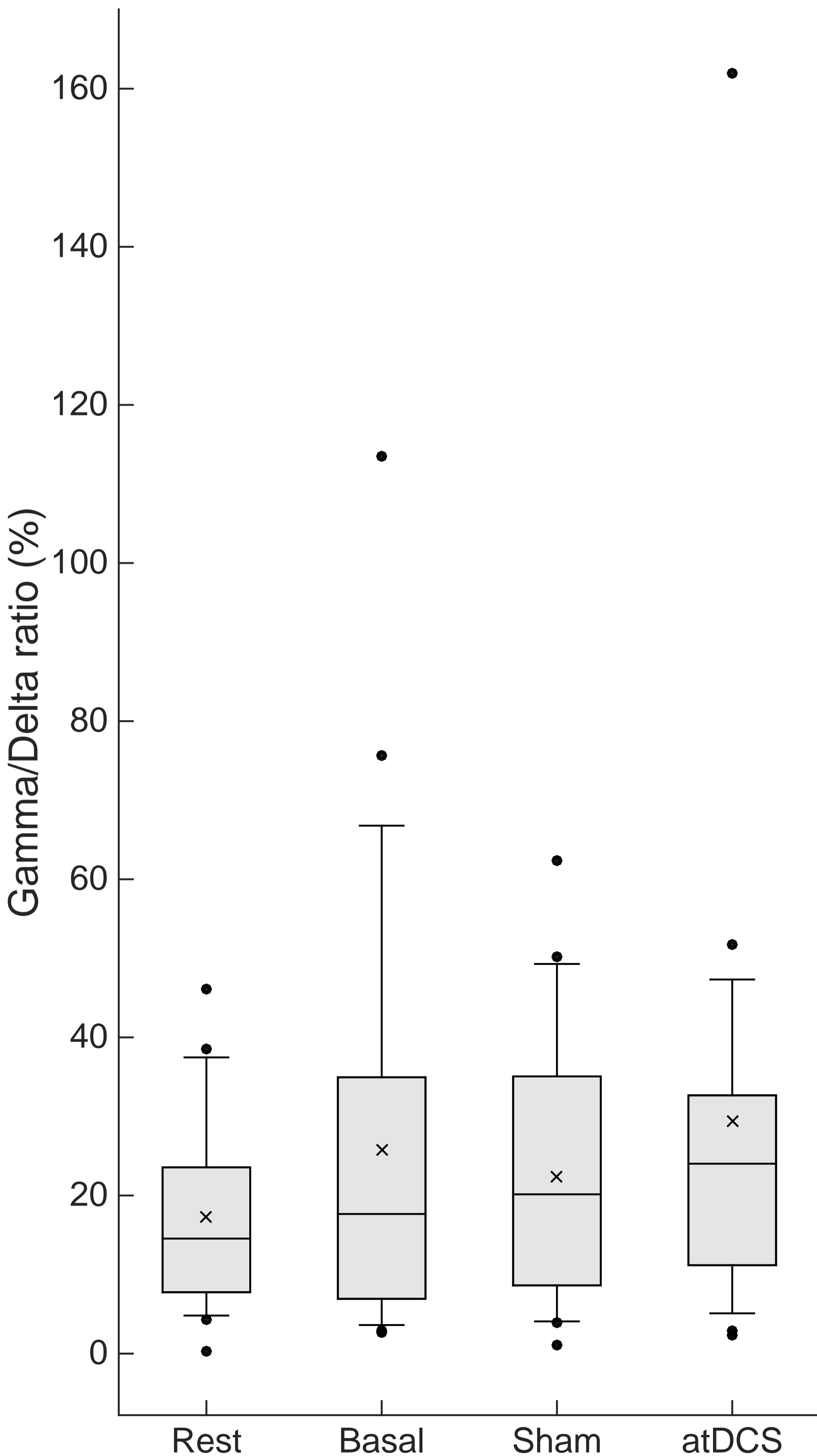

Supplement: Supplementary file 1 [file Data_Sheet_1.zip › Complementary_results/Band_ratios_average_PSD_windows/Gamma_Delta/Gamma-Delta_mean-win_O2.pdf]

**Gamma/Delta ratio on average  
PSD windows for electrode: P7**

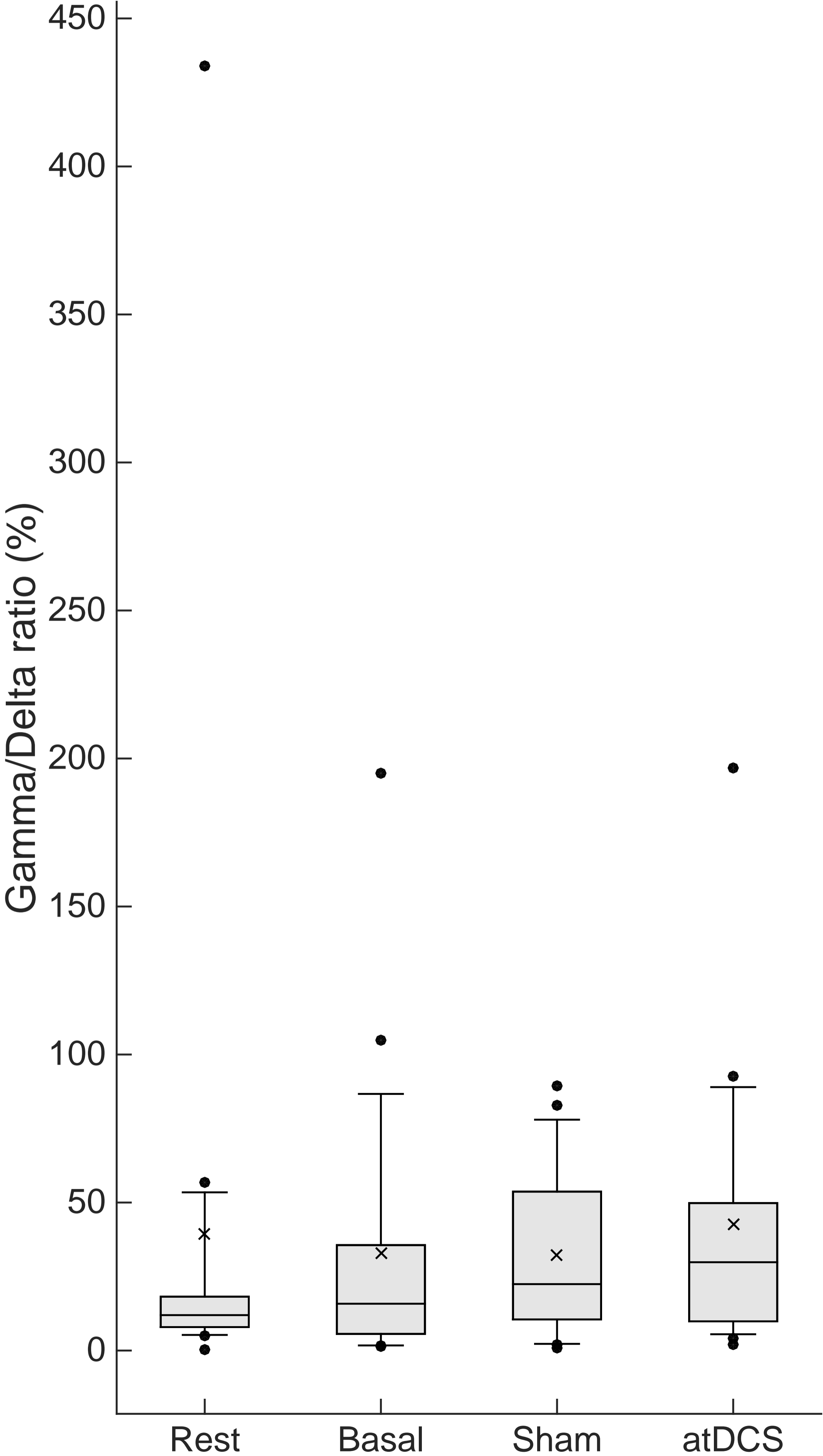

Supplement: Supplementary file 1 [file Data_Sheet_1.zip › Complementary_results/Band_ratios_average_PSD_windows/Gamma_Delta/Gamma-Delta_mean-win_P7.pdf]

**Gamma/Delta ratio on average  
PSD windows for electrode: P8**

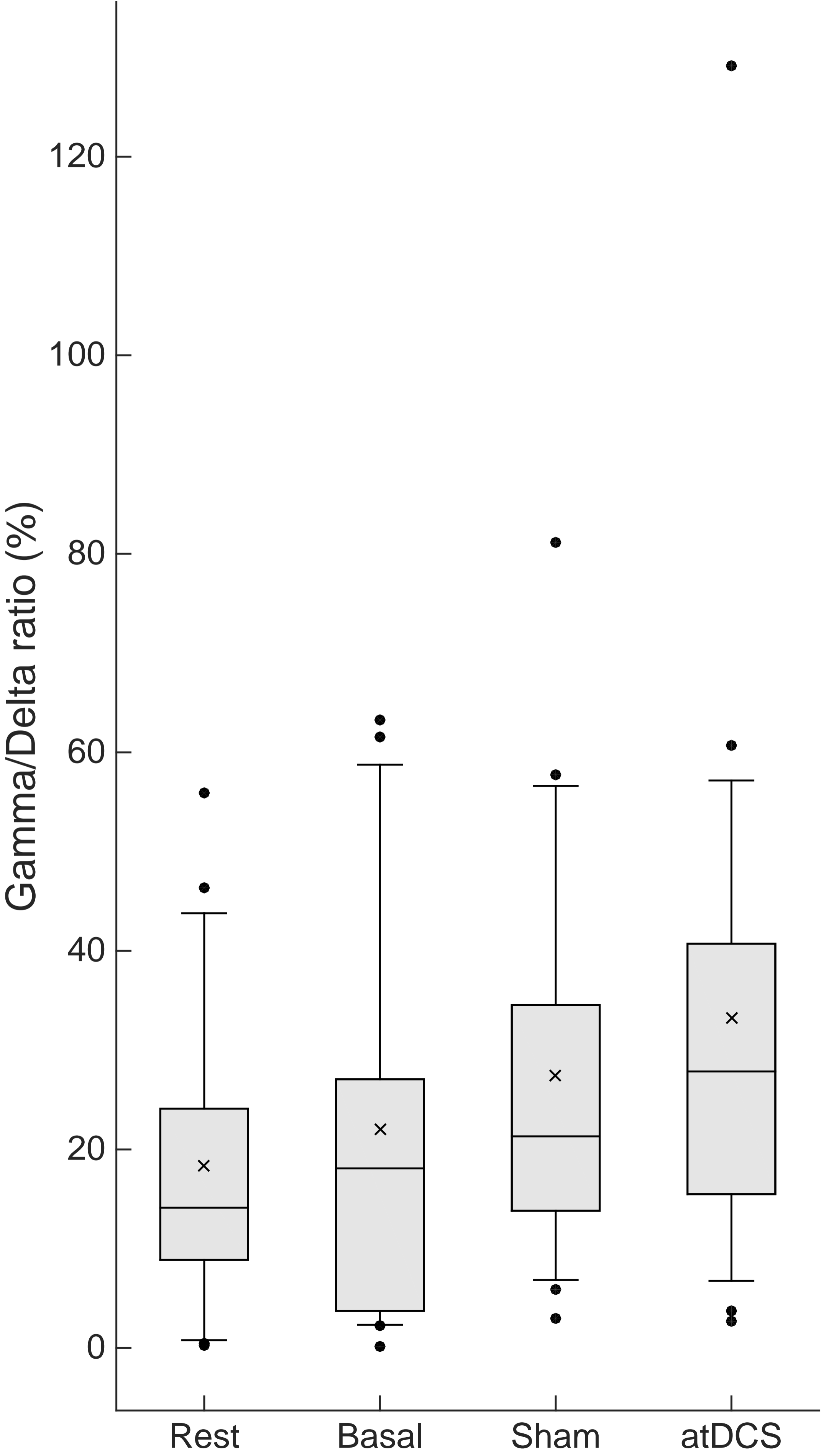

Supplement: Supplementary file 1 [file Data_Sheet_1.zip › Complementary_results/Band_ratios_average_PSD_windows/Gamma_Delta/Gamma-Delta_mean-win_P8.pdf]

**Gamma/Delta ratio on average  
PSD windows for electrode: T7**

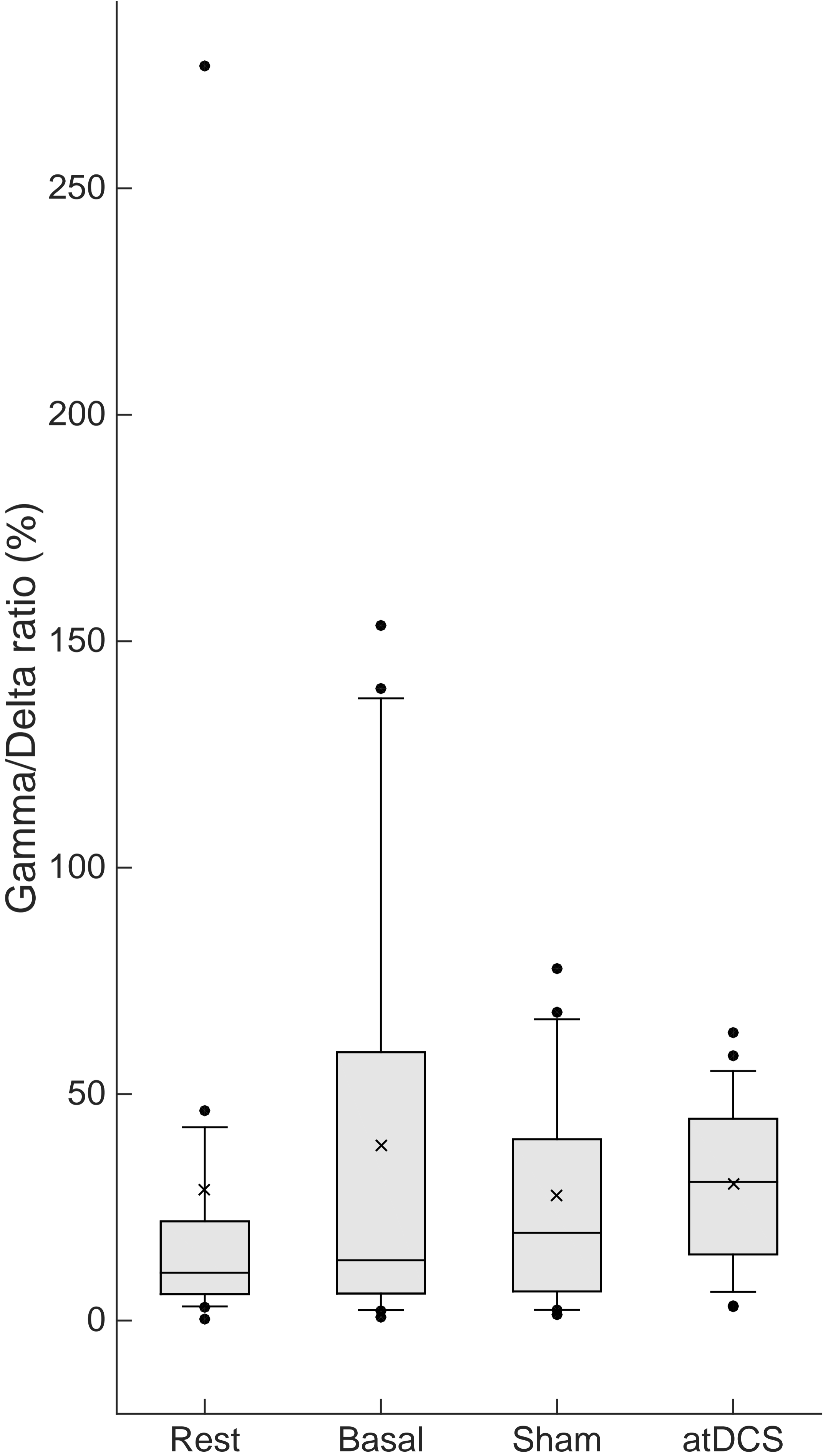

Supplement: Supplementary file 1 [file Data_Sheet_1.zip › Complementary_results/Band_ratios_average_PSD_windows/Gamma_Delta/Gamma-Delta_mean-win_T7.pdf]

**Gamma/Delta ratio on average  
PSD windows for electrode: T8**

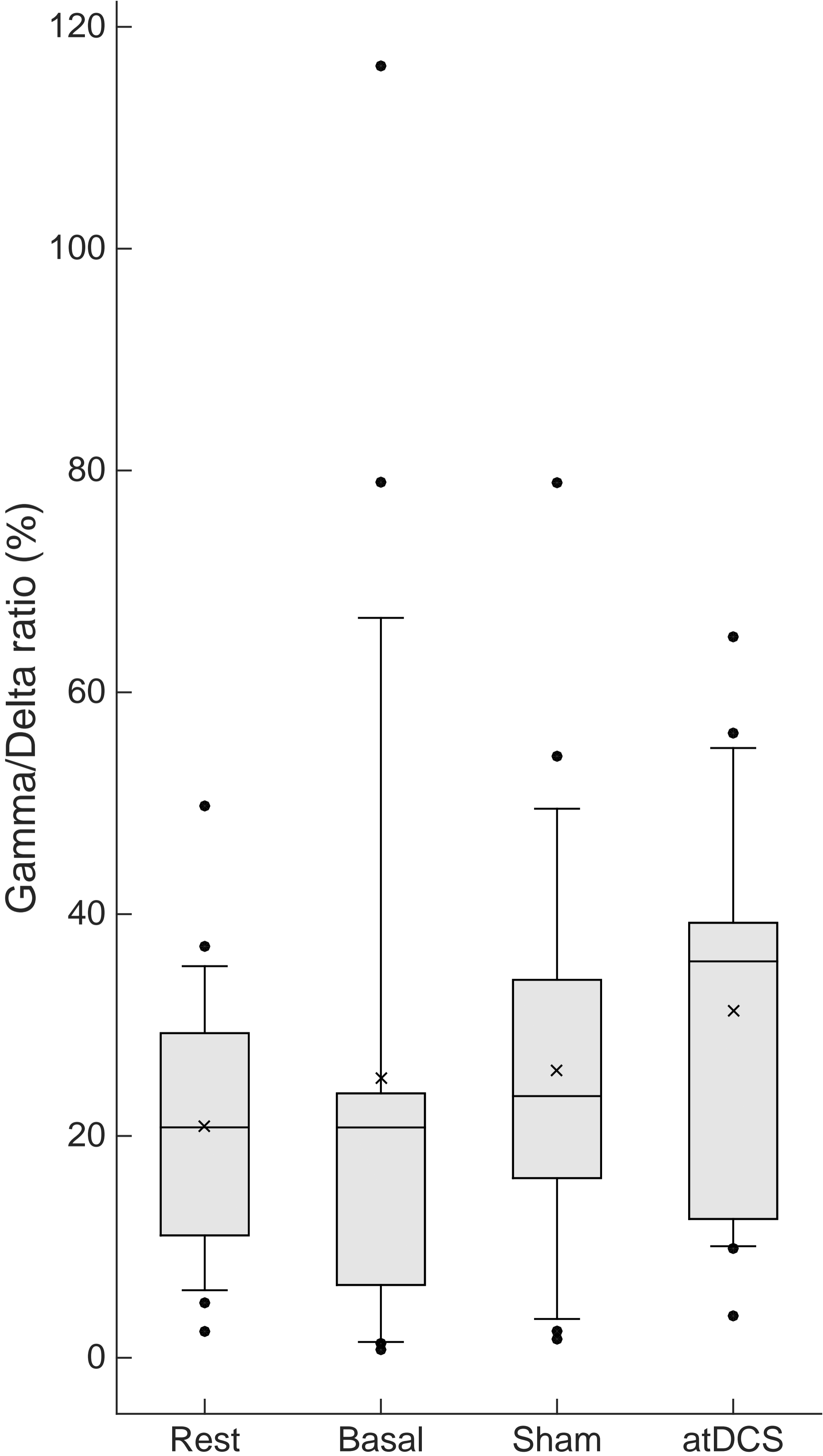

Supplement: Supplementary file 1 [file Data_Sheet_1.zip › Complementary_results/Band_ratios_average_PSD_windows/Gamma_Delta/Gamma-Delta_mean-win_T8.pdf]

**Theta/Delta ratio on average  
PSD windows for electrode: AF3**

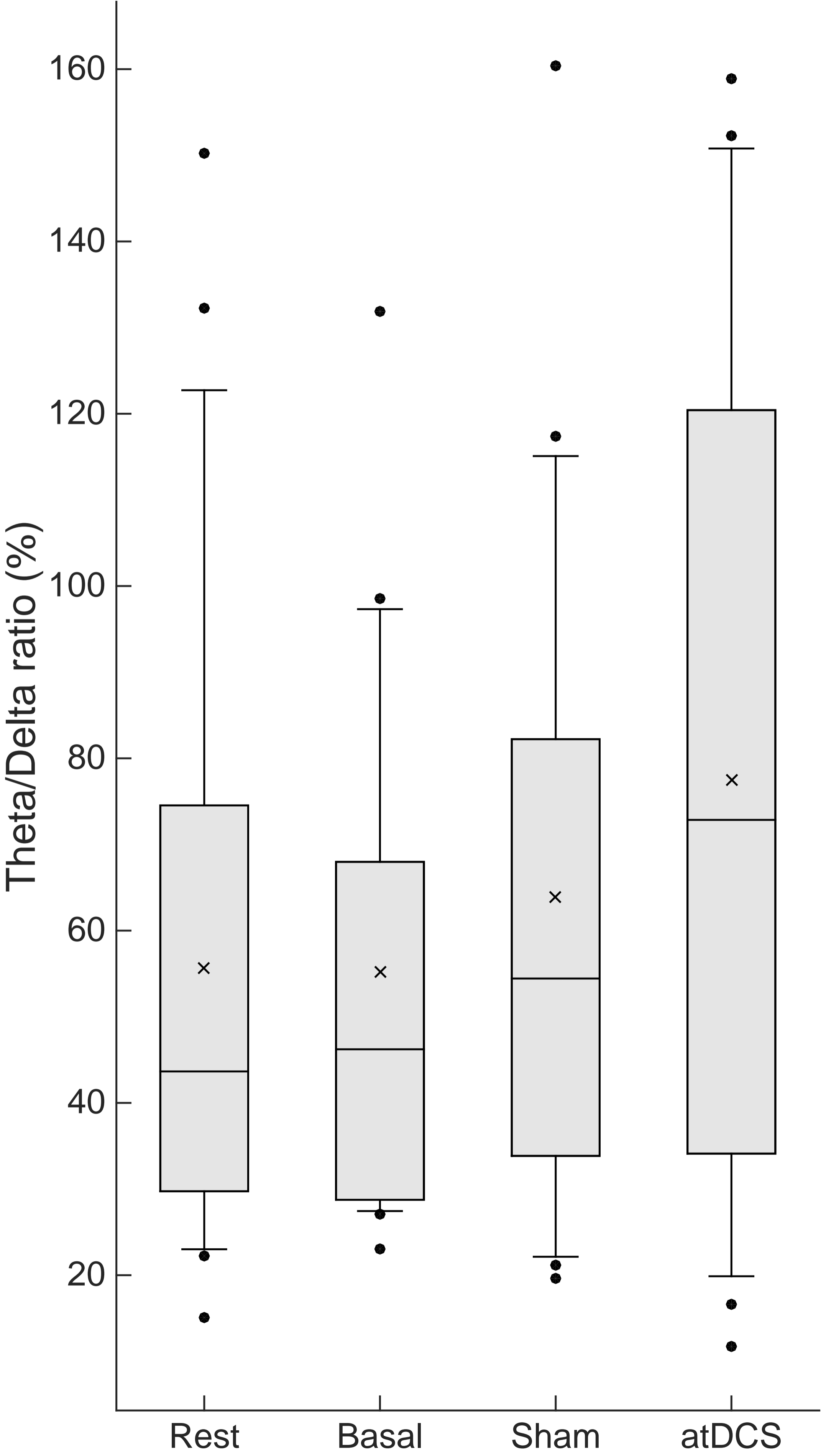

Supplement: Supplementary file 1 [file Data_Sheet_1.zip › Complementary_results/Band_ratios_average_PSD_windows/Theta_Delta/Theta-Delta_mean-win_AF3.pdf]

**Theta/Delta ratio on average  
PSD windows for electrode: AF4**

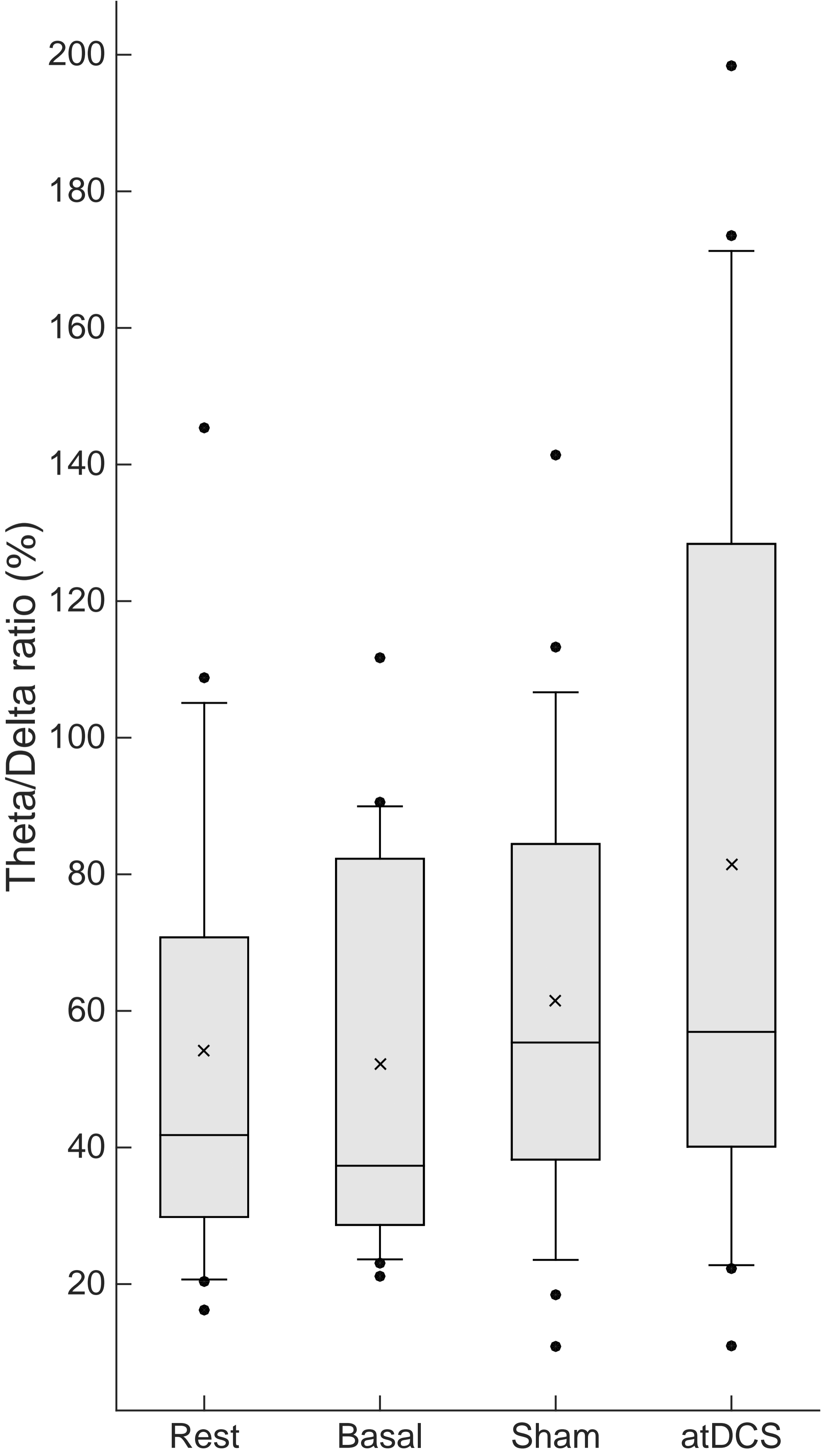

Supplement: Supplementary file 1 [file Data_Sheet_1.zip › Complementary_results/Band_ratios_average_PSD_windows/Theta_Delta/Theta-Delta_mean-win_AF4.pdf]

**Theta/Delta ratio on average**  
**PSD windows for electrode: Avg AF3-F3-F7**

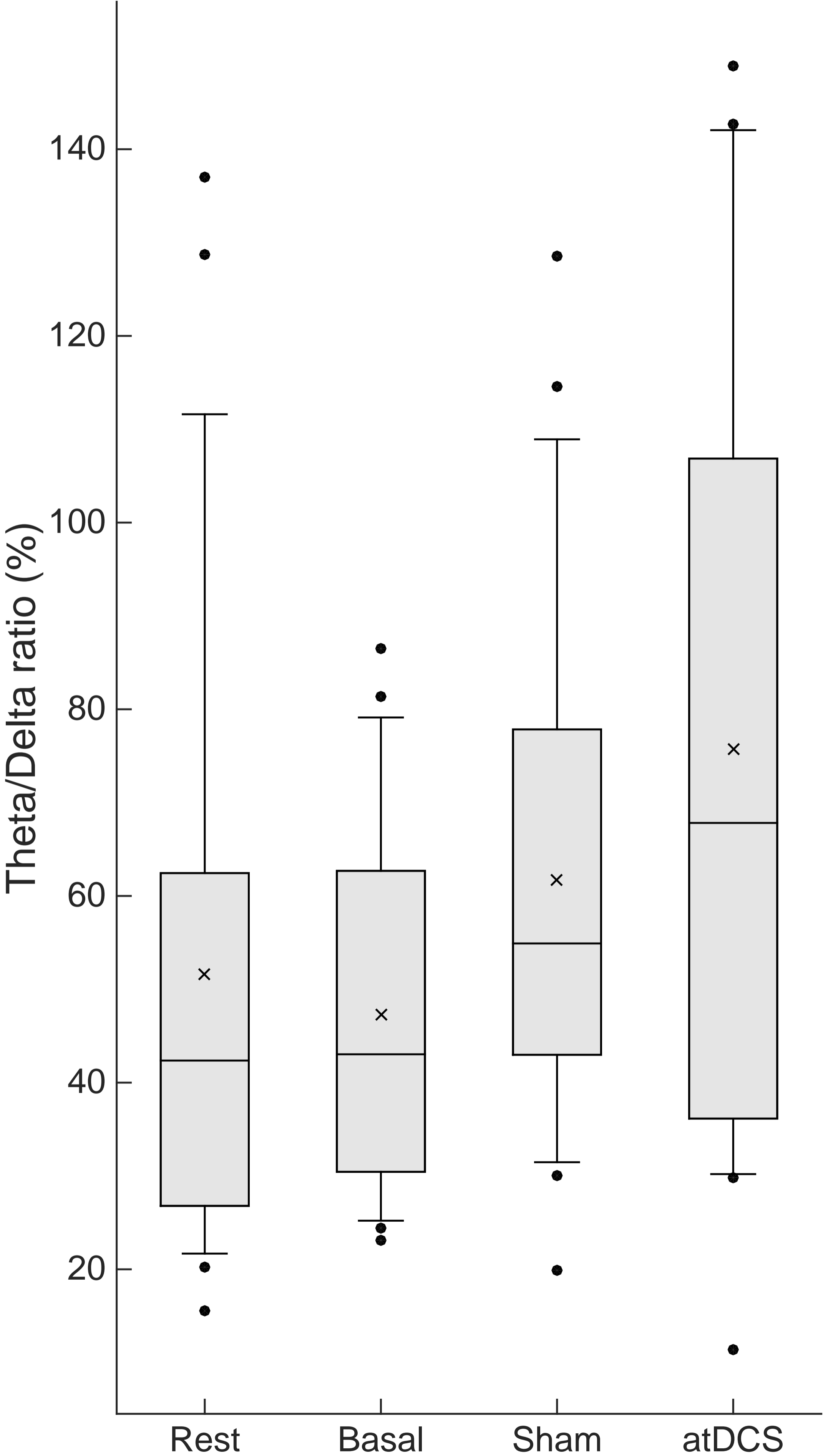

Supplement: Supplementary file 1 [file Data_Sheet_1.zip › Complementary_results/Band_ratios_average_PSD_windows/Theta_Delta/Theta-Delta_mean-win_Avg AF3-F3-F7.pdf]

**Theta/Delta ratio on average**  
**PSD windows for electrode: Avg AF4-F4-F8**

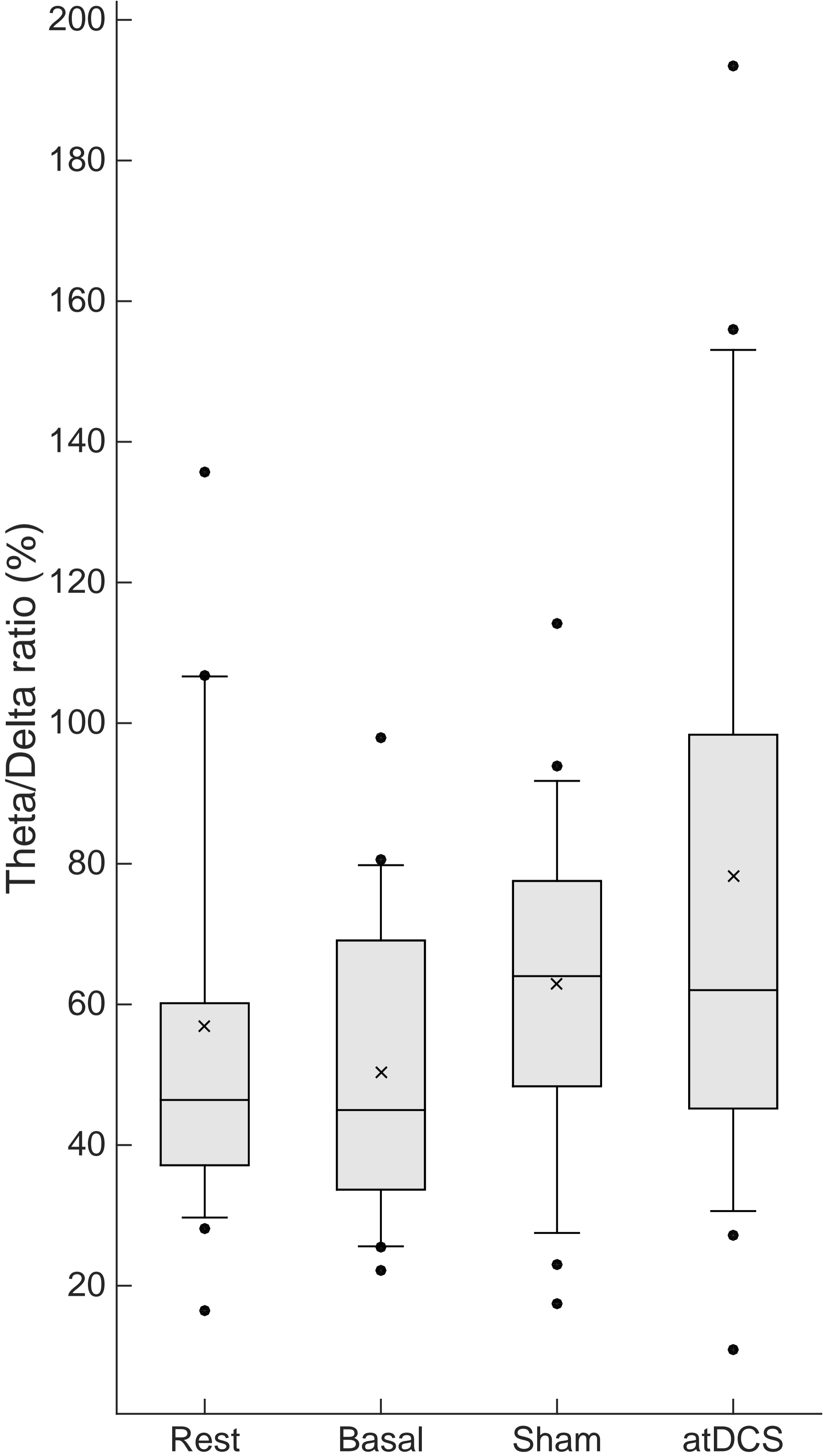

Supplement: Supplementary file 1 [file Data_Sheet_1.zip › Complementary_results/Band_ratios_average_PSD_windows/Theta_Delta/Theta-Delta_mean-win_Avg AF4-F4-F8.pdf]

**Theta/Delta ratio on average**  
**PSD windows for electrode: Avg F3-F7-FC5**

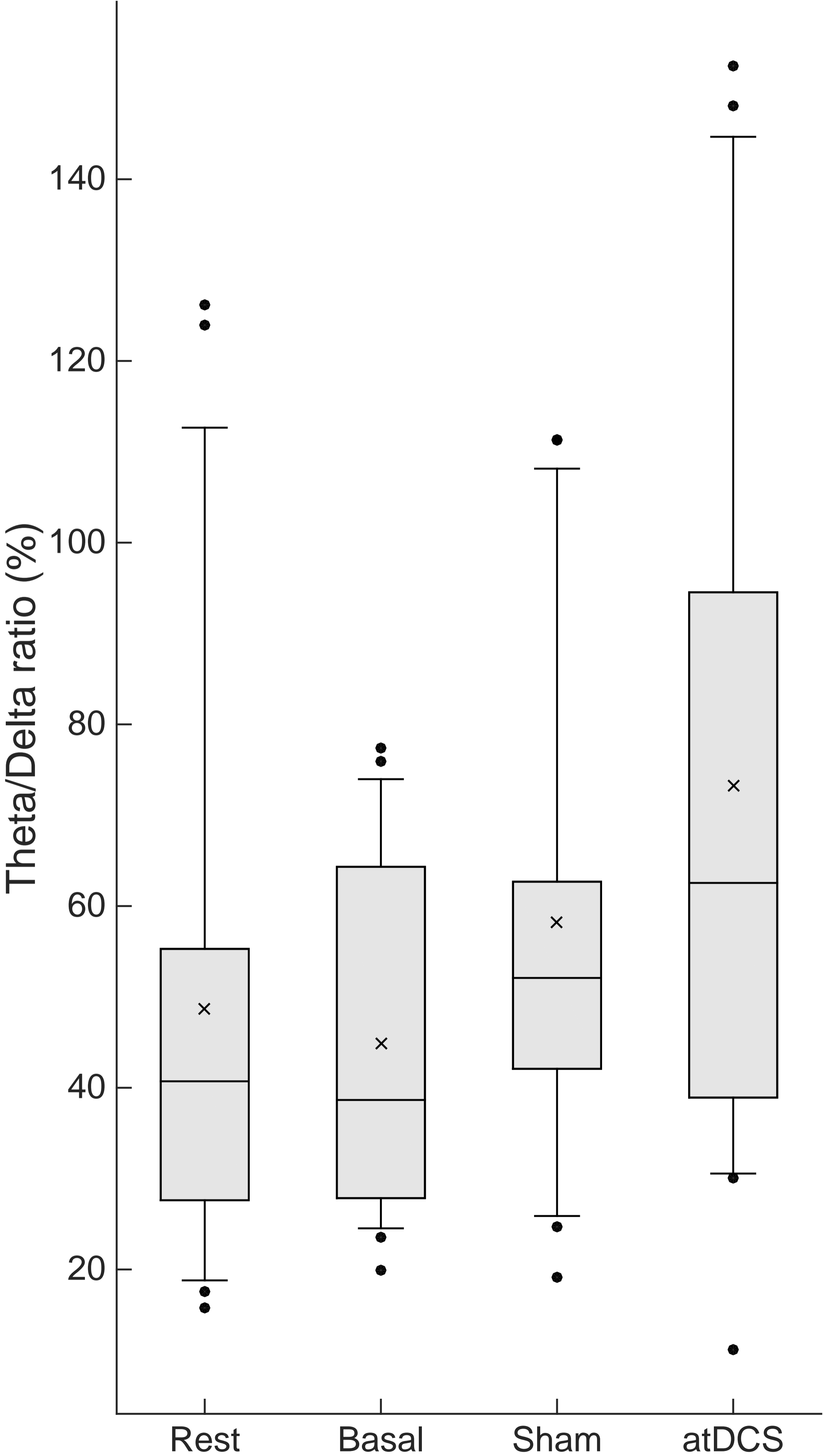

Supplement: Supplementary file 1 [file Data_Sheet_1.zip › Complementary_results/Band_ratios_average_PSD_windows/Theta_Delta/Theta-Delta_mean-win_Avg F3-F7-FC5.pdf]

**Theta/Delta ratio on average**  
**PSD windows for electrode: Avg F4-F8-FC6**

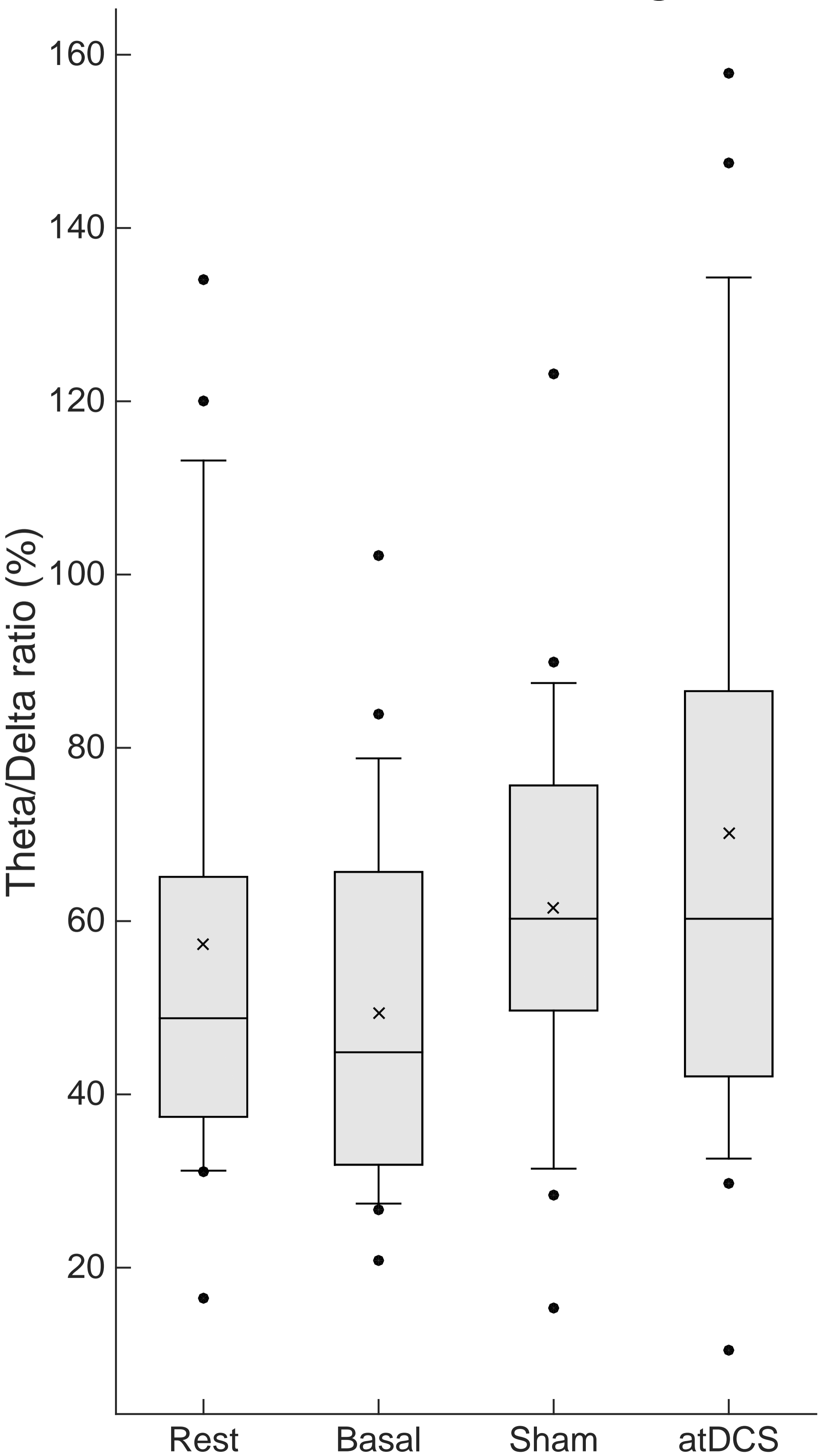

Supplement: Supplementary file 1 [file Data_Sheet_1.zip › Complementary_results/Band_ratios_average_PSD_windows/Theta_Delta/Theta-Delta_mean-win_Avg F4-F8-FC6.pdf]

**Theta/Delta ratio on average  
PSD windows for electrode: F3**

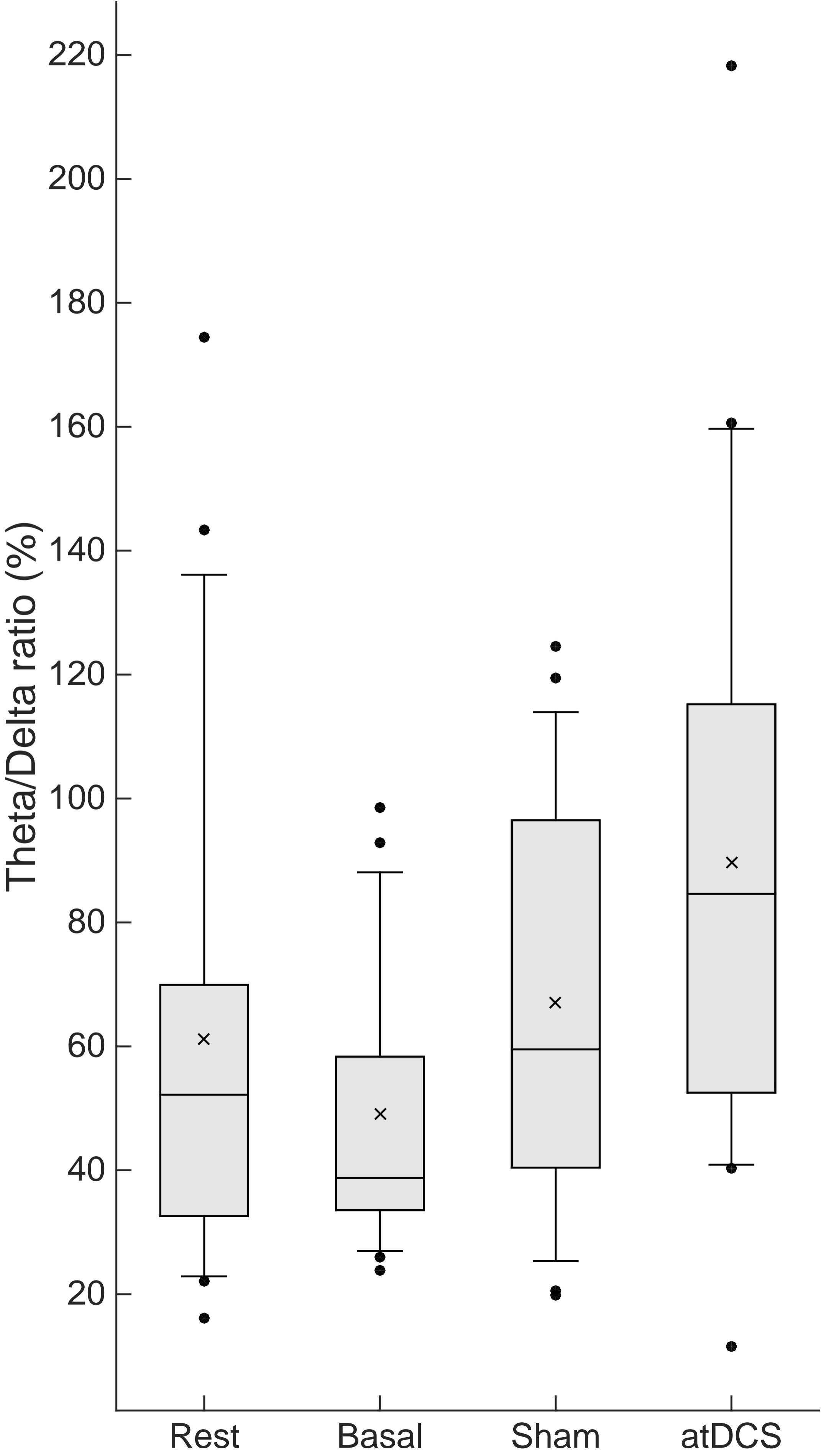

Supplement: Supplementary file 1 [file Data_Sheet_1.zip › Complementary_results/Band_ratios_average_PSD_windows/Theta_Delta/Theta-Delta_mean-win_F3.pdf]

Theta/Delta ratio on average  
PSD windows for electrode: F4

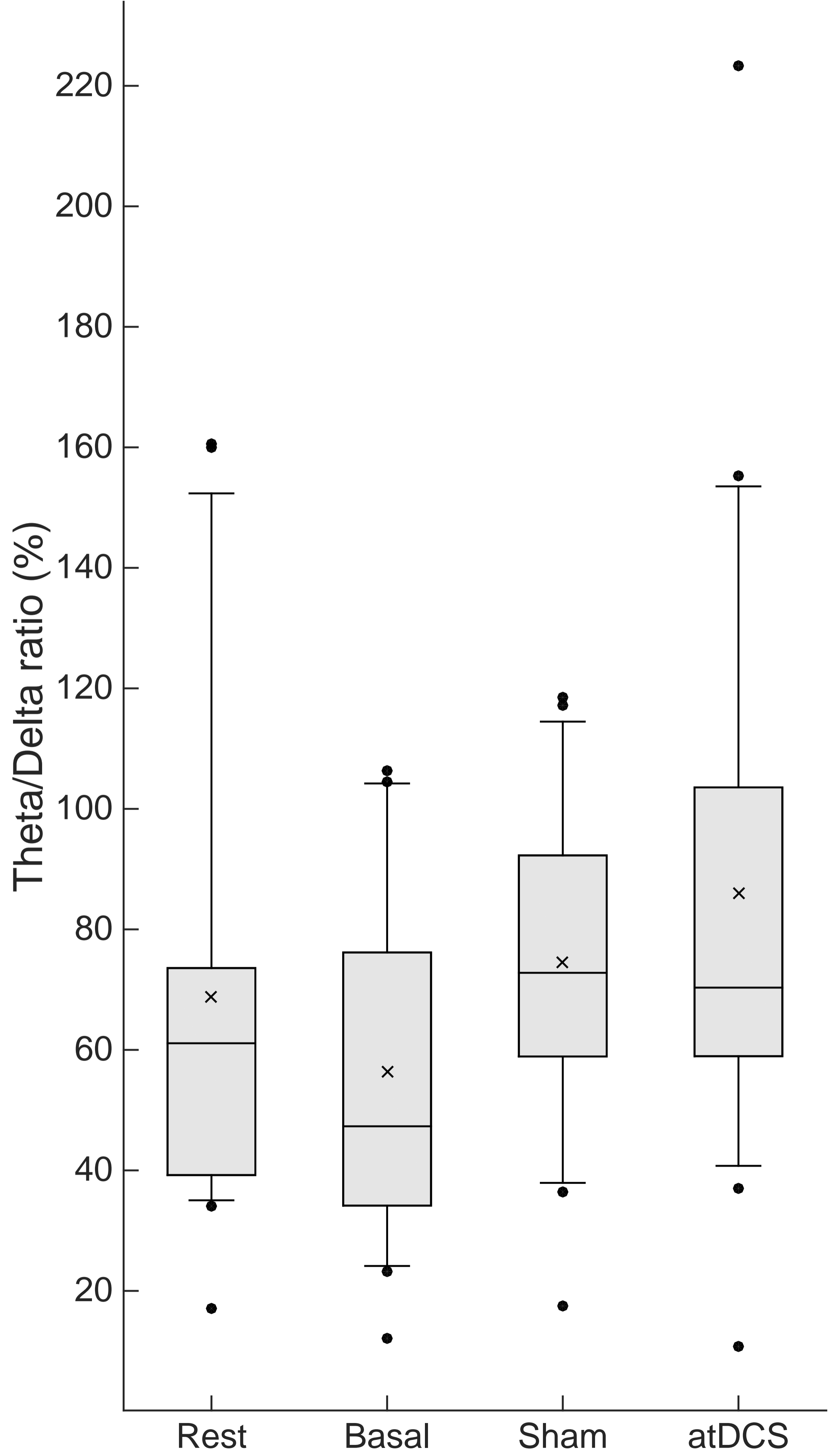

Supplement: Supplementary file 1 [file Data_Sheet_1.zip › Complementary_results/Band_ratios_average_PSD_windows/Theta_Delta/Theta-Delta_mean-win_F4.pdf]

**Theta/Delta ratio on average  
PSD windows for electrode: F7**

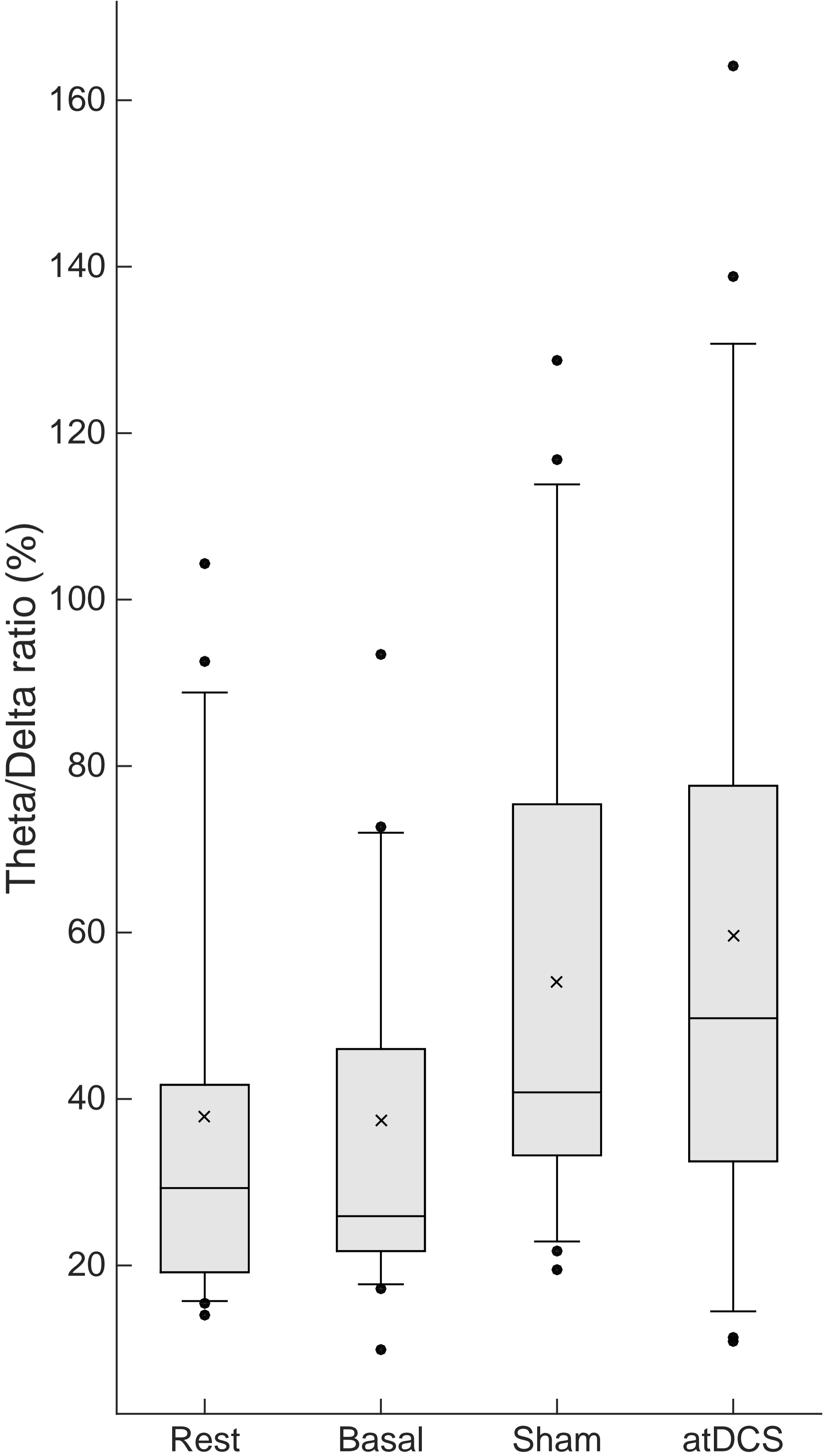

Supplement: Supplementary file 1 [file Data_Sheet_1.zip › Complementary_results/Band_ratios_average_PSD_windows/Theta_Delta/Theta-Delta_mean-win_F7.pdf]

**Theta/Delta ratio on average  
PSD windows for electrode: F8**

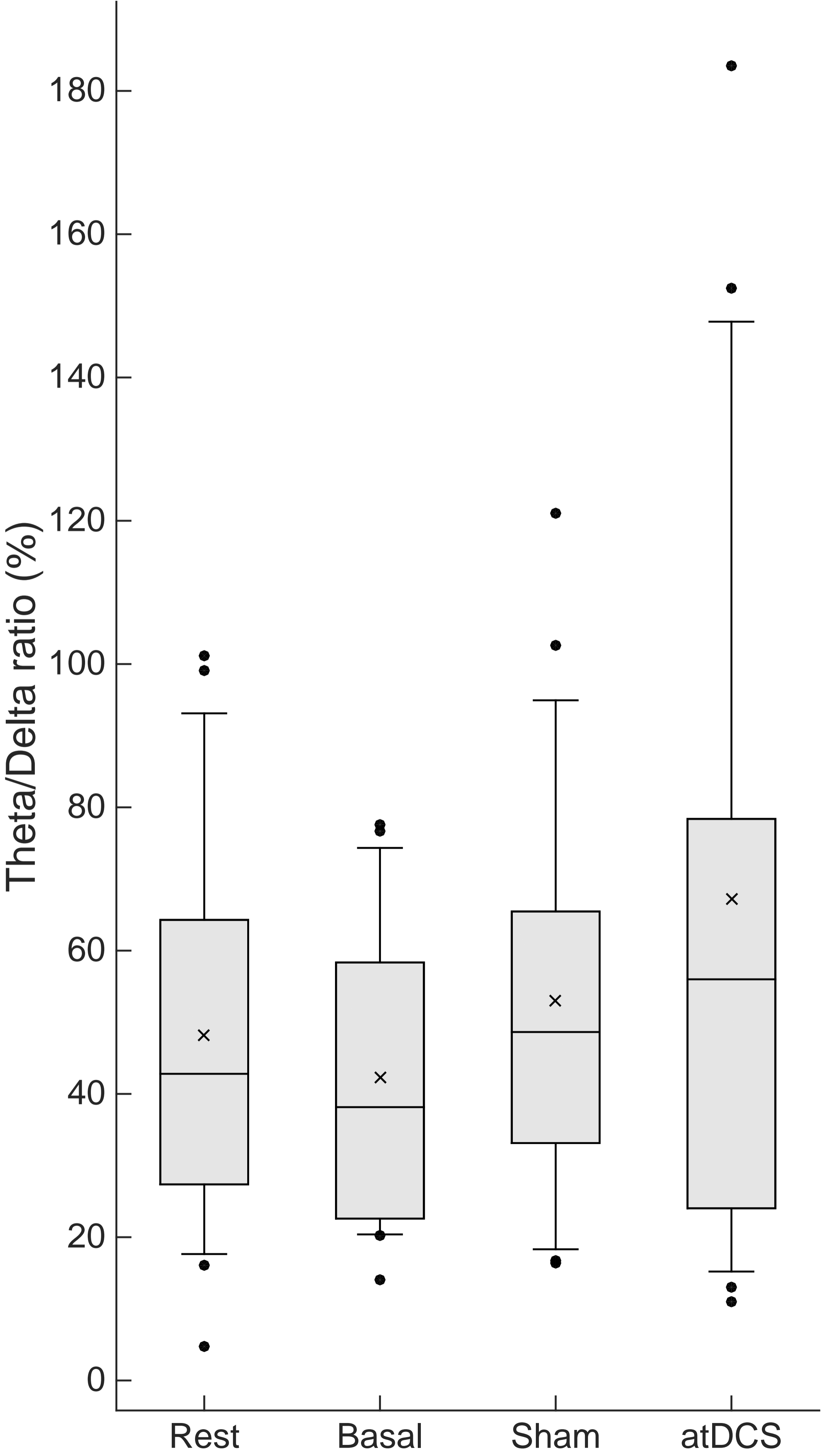

Supplement: Supplementary file 1 [file Data_Sheet_1.zip › Complementary_results/Band_ratios_average_PSD_windows/Theta_Delta/Theta-Delta_mean-win_F8.pdf]

**Theta/Delta ratio on average  
PSD windows for electrode: FC5**

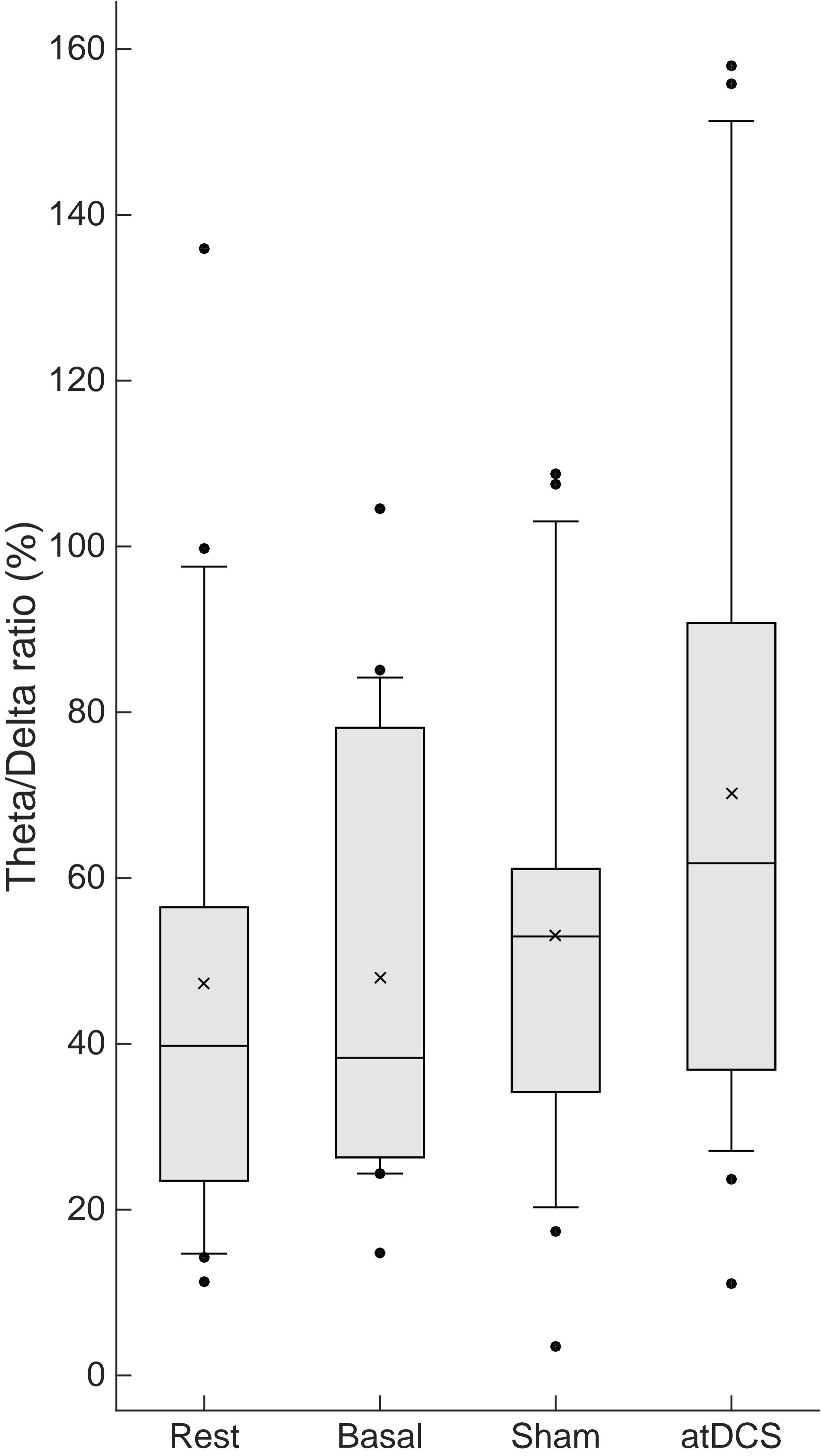

Supplement: Supplementary file 1 [file Data_Sheet_1.zip › Complementary_results/Band_ratios_average_PSD_windows/Theta_Delta/Theta-Delta_mean-win_FC5.pdf]

**Theta/Delta ratio on average  
PSD windows for electrode: FC6**

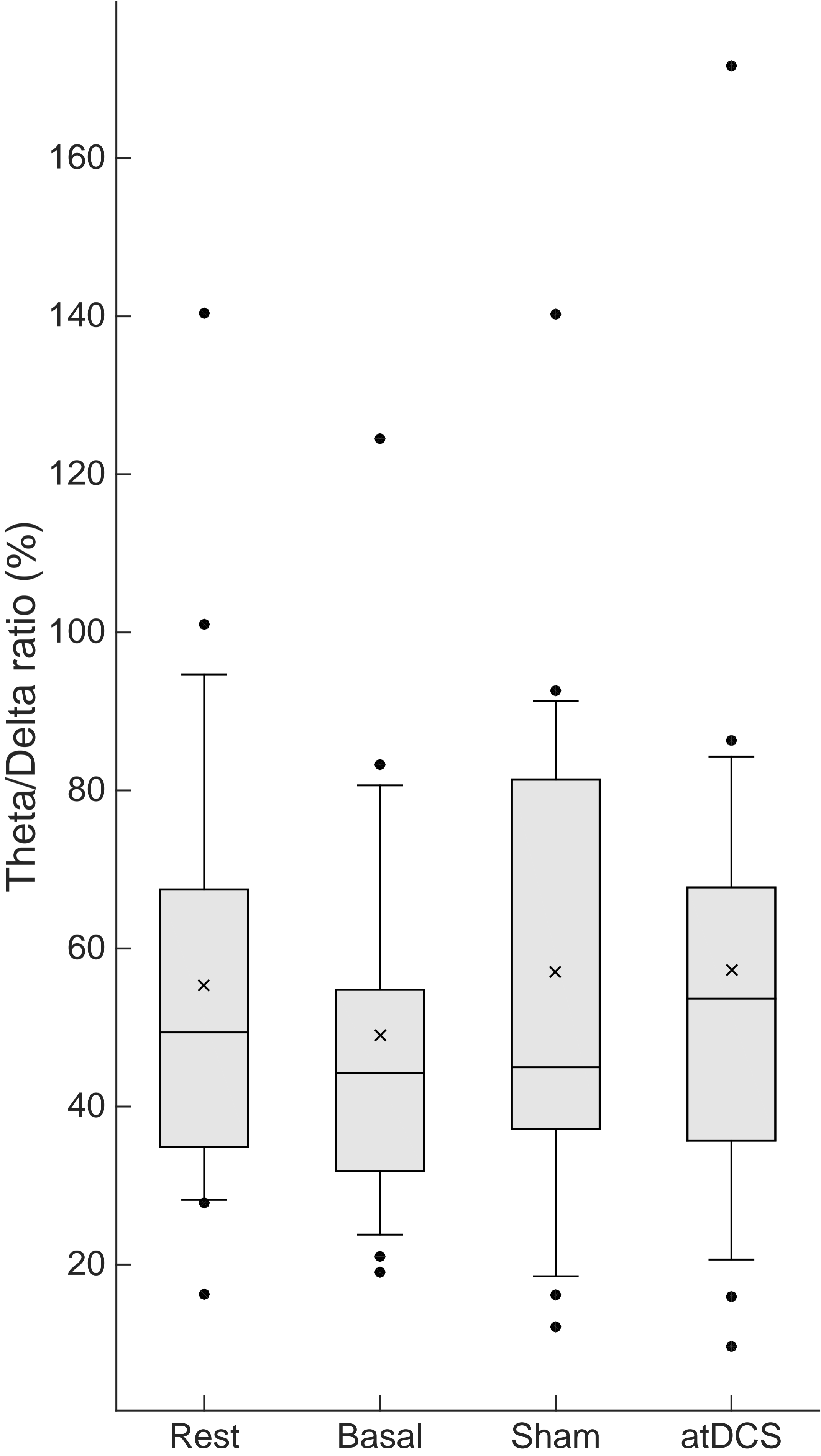

Supplement: Supplementary file 1 [file Data_Sheet_1.zip › Complementary_results/Band_ratios_average_PSD_windows/Theta_Delta/Theta-Delta_mean-win_FC6.pdf]

# Theta/Delta ratio on average PSD windows for electrode: O1

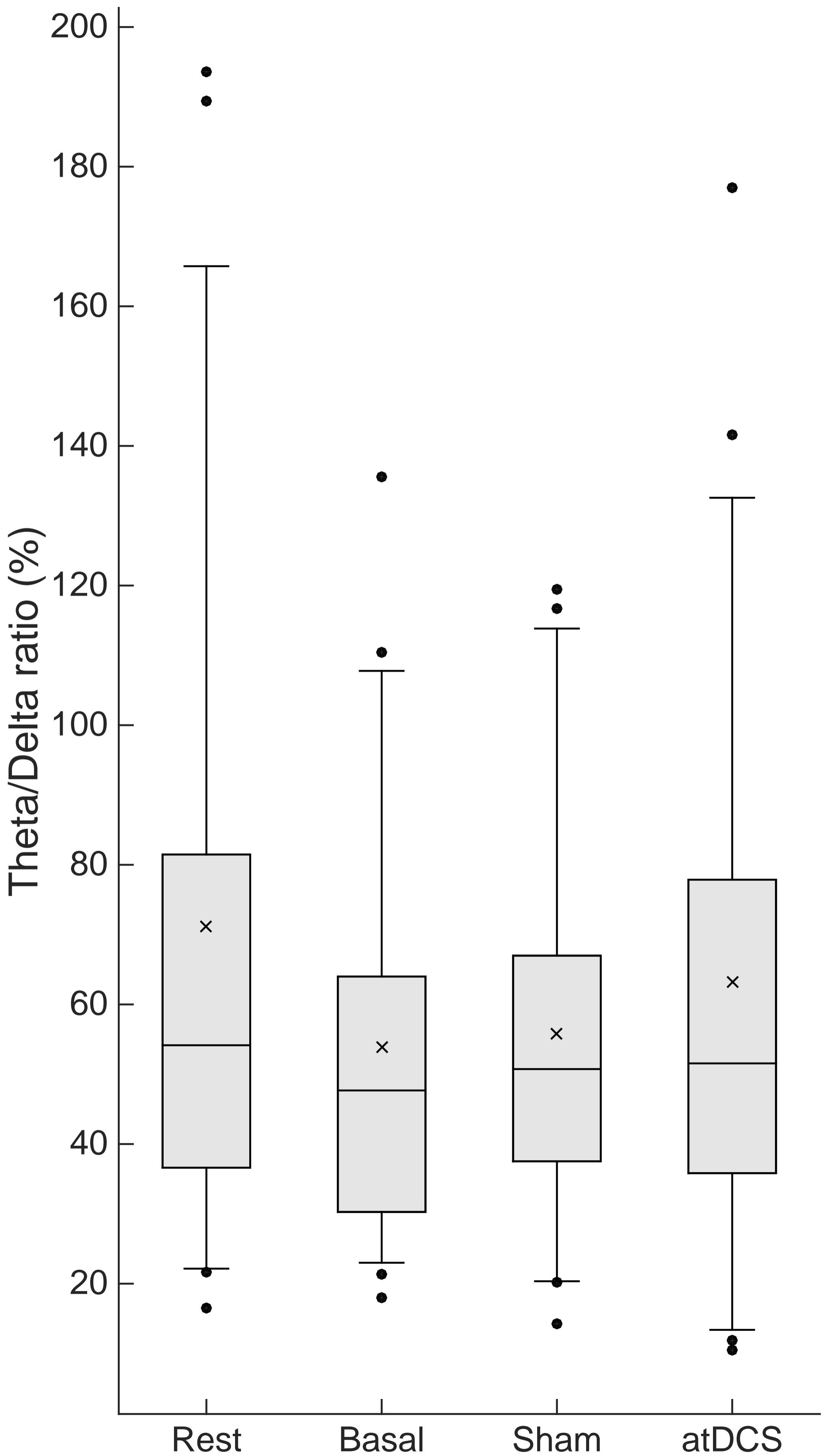

Supplement: Supplementary file 1 [file Data_Sheet_1.zip › Complementary_results/Band_ratios_average_PSD_windows/Theta_Delta/Theta-Delta_mean-win_O1.pdf]

# Theta/Delta ratio on average PSD windows for electrode: O2

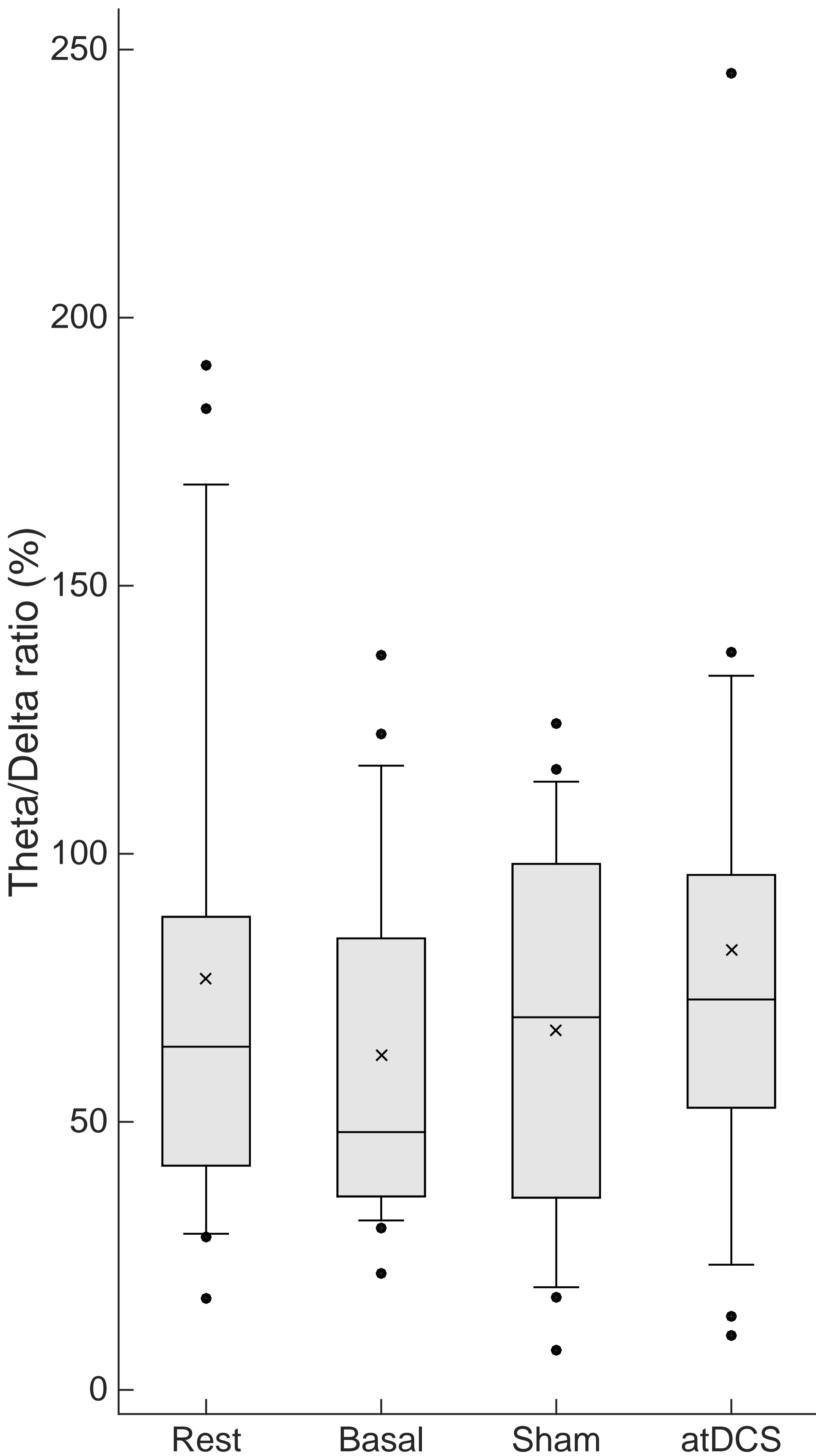

Supplement: Supplementary file 1 [file Data_Sheet_1.zip › Complementary_results/Band_ratios_average_PSD_windows/Theta_Delta/Theta-Delta_mean-win_O2.pdf]

**Theta/Delta ratio on average  
PSD windows for electrode: P7**

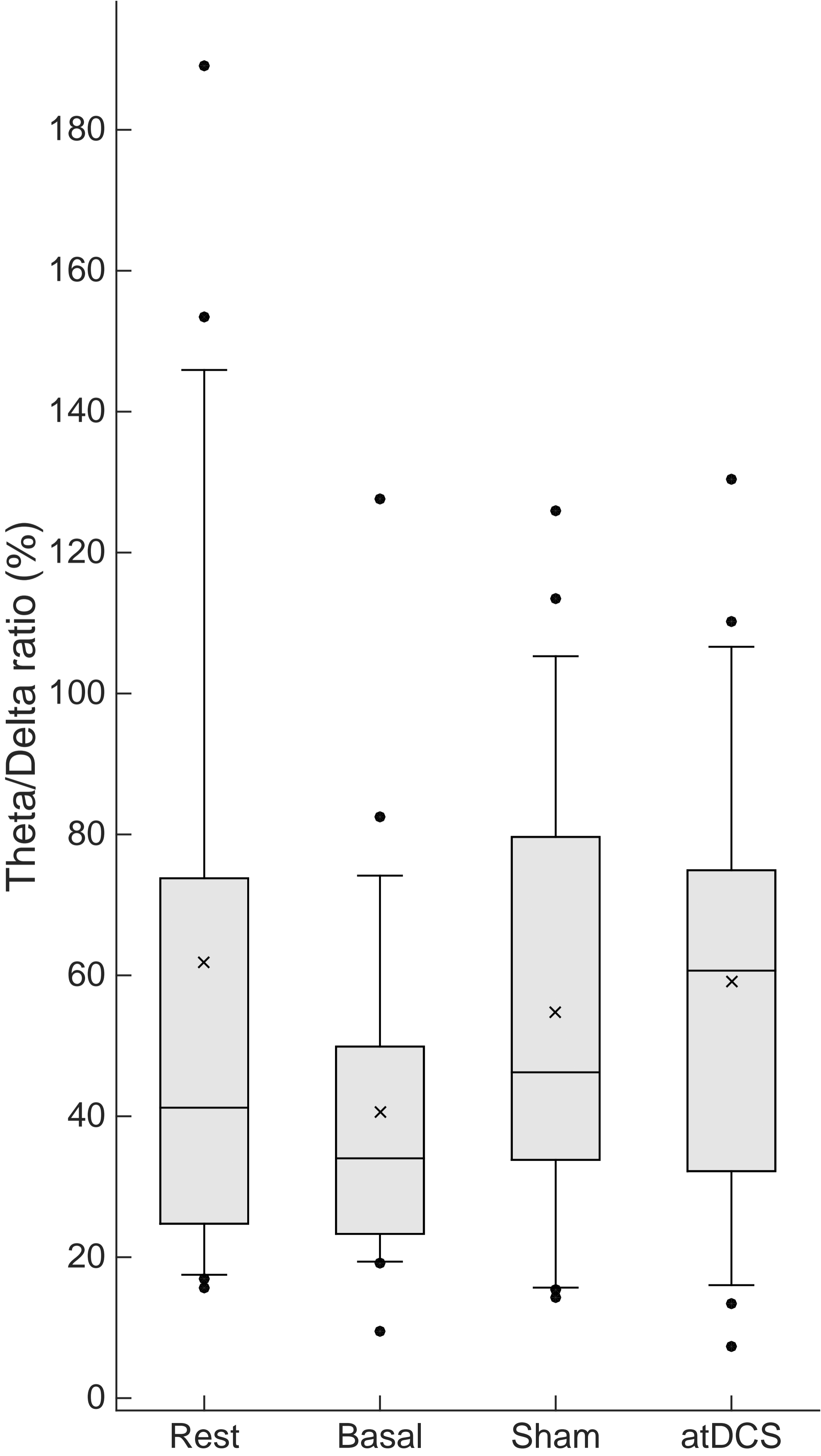

Supplement: Supplementary file 1 [file Data_Sheet_1.zip › Complementary_results/Band_ratios_average_PSD_windows/Theta_Delta/Theta-Delta_mean-win_P7.pdf]

**Theta/Delta ratio on average  
PSD windows for electrode: P8**

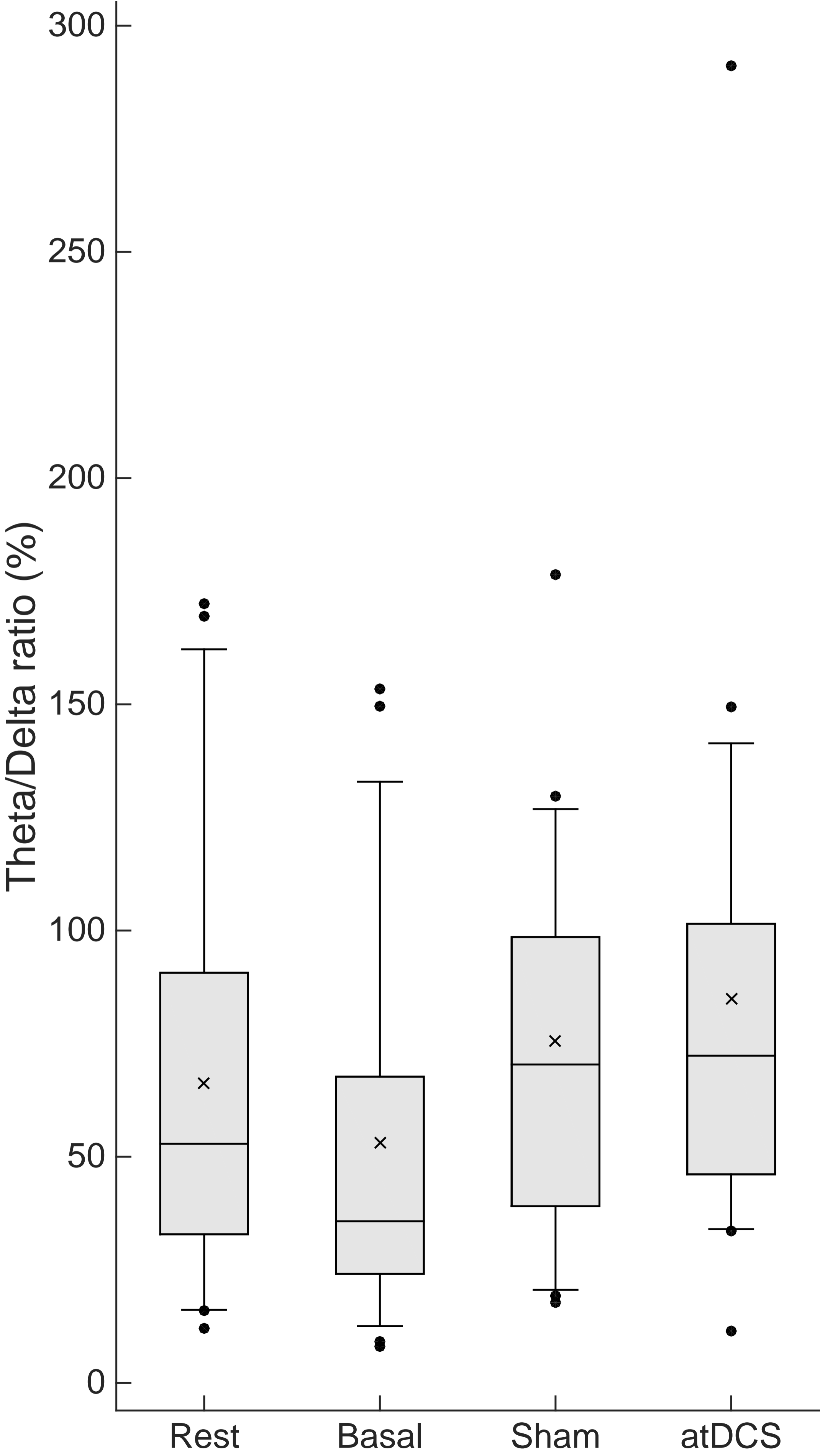

Supplement: Supplementary file 1 [file Data_Sheet_1.zip › Complementary_results/Band_ratios_average_PSD_windows/Theta_Delta/Theta-Delta_mean-win_P8.pdf]

Theta/Delta ratio on average  
PSD windows for electrode: T7

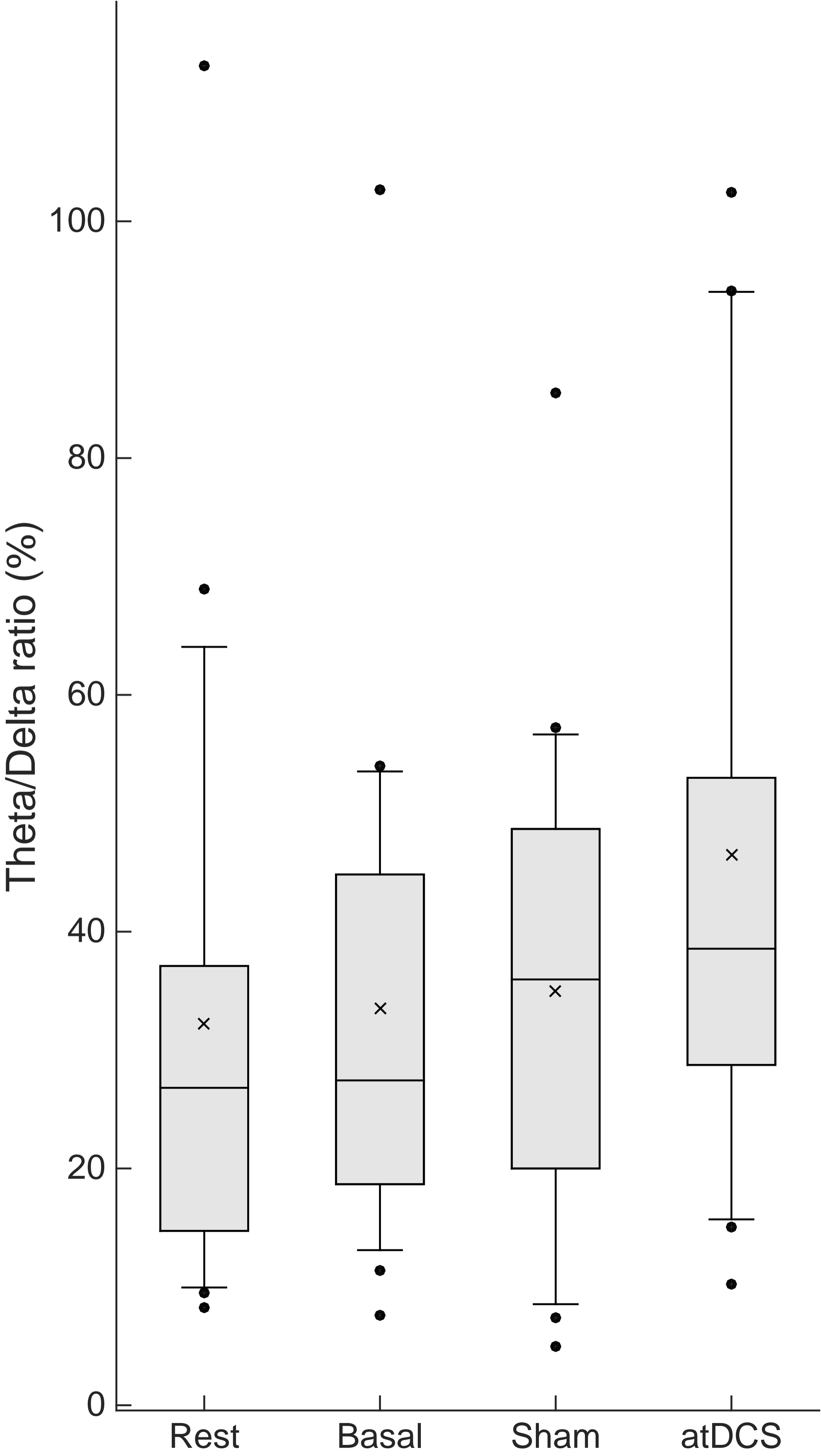

Supplement: Supplementary file 1 [file Data_Sheet_1.zip › Complementary_results/Band_ratios_average_PSD_windows/Theta_Delta/Theta-Delta_mean-win_T7.pdf]

Theta/Delta ratio on average  
PSD windows for electrode: T8

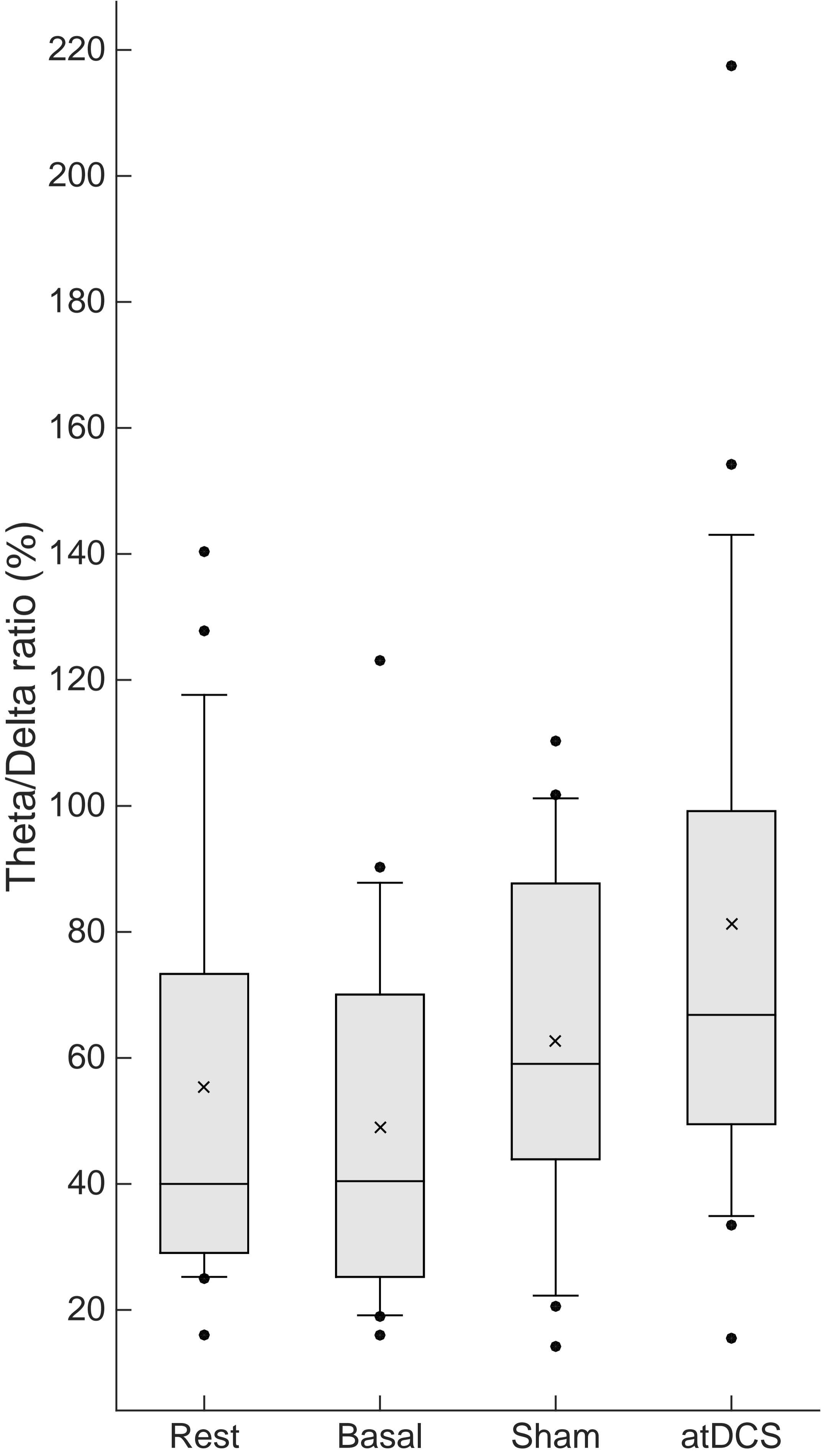

Supplement: Supplementary file 1 [file Data_Sheet_1.zip › Complementary_results/Band_ratios_average_PSD_windows/Theta_Delta/Theta-Delta_mean-win_T8.pdf]

Alpha/Delta ratio on complete EEG signal for electrode: AF3

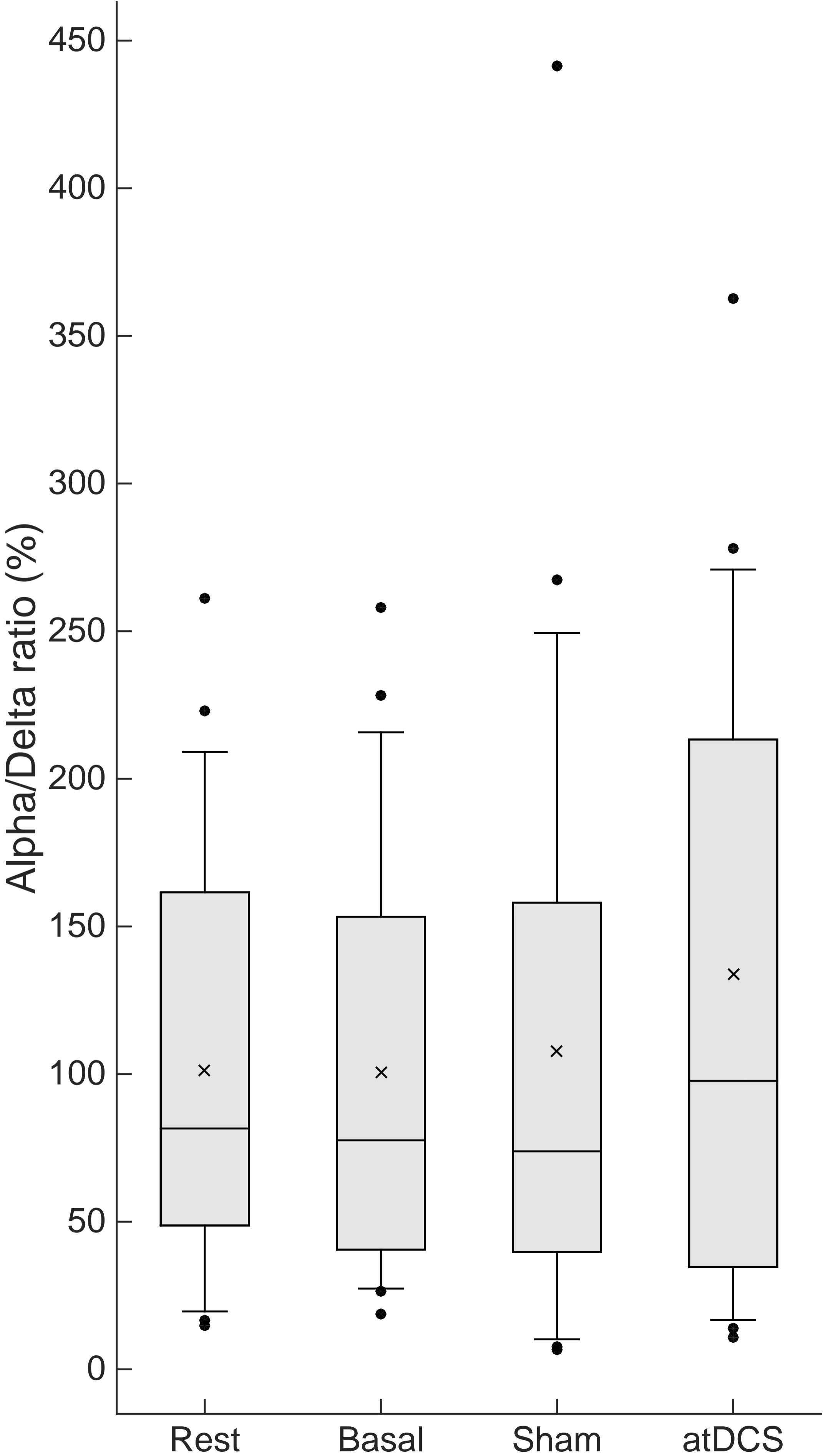

Supplement: Supplementary file 1 [file Data_Sheet_1.zip › Complementary_results/Band_ratios_Complete_EEG/Alpha_Delta/Alpha-Delta_complete-EEG_AF3.pdf]

Alpha/Delta ratio on complete EEG signal for electrode: AF4

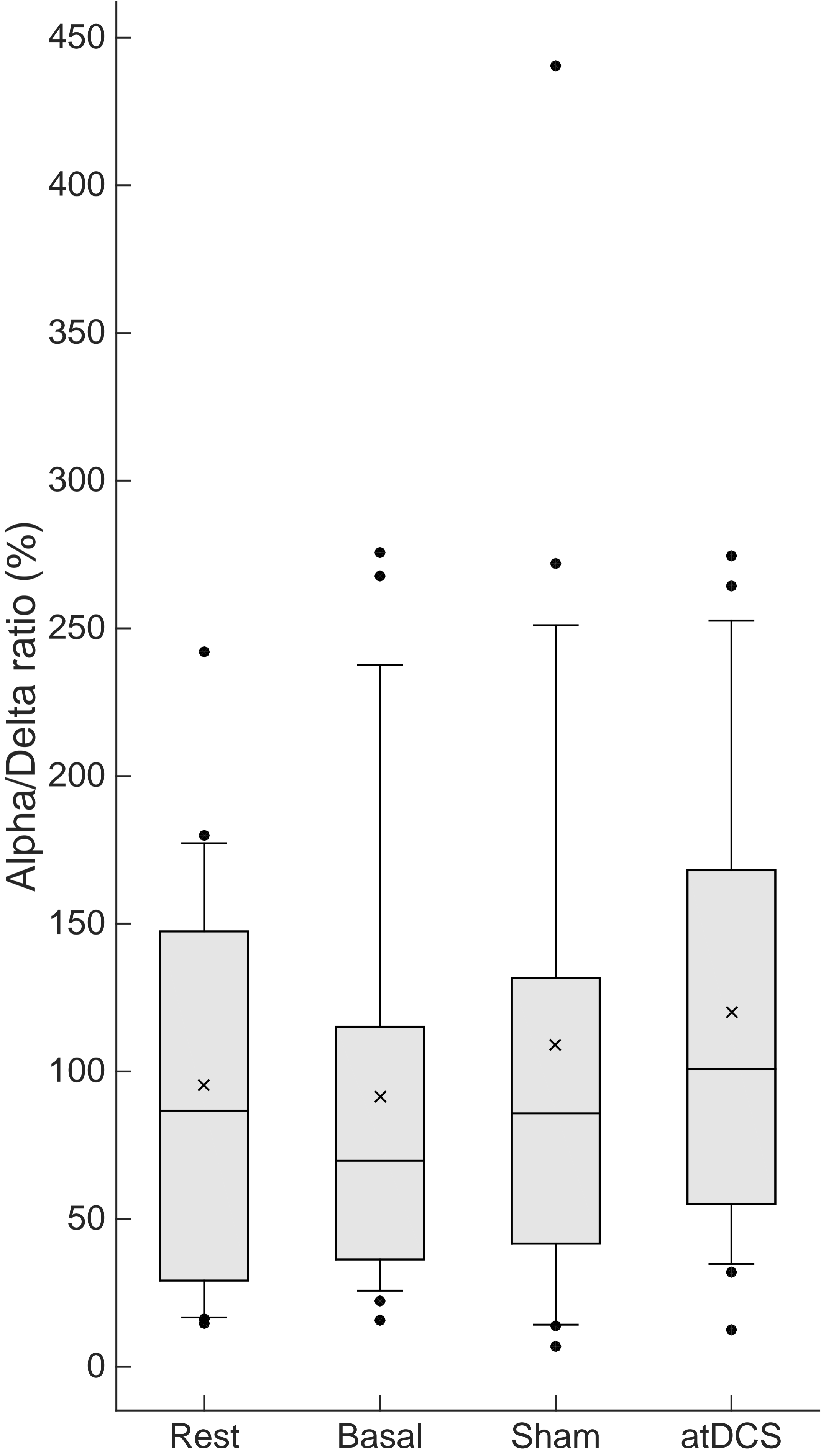

Supplement: Supplementary file 1 [file Data_Sheet_1.zip › Complementary_results/Band_ratios_Complete_EEG/Alpha_Delta/Alpha-Delta_complete-EEG_AF4.pdf]

**Alpha/Delta ratio on complete EEG signal for electrode: Avg AF3-F3-F7**

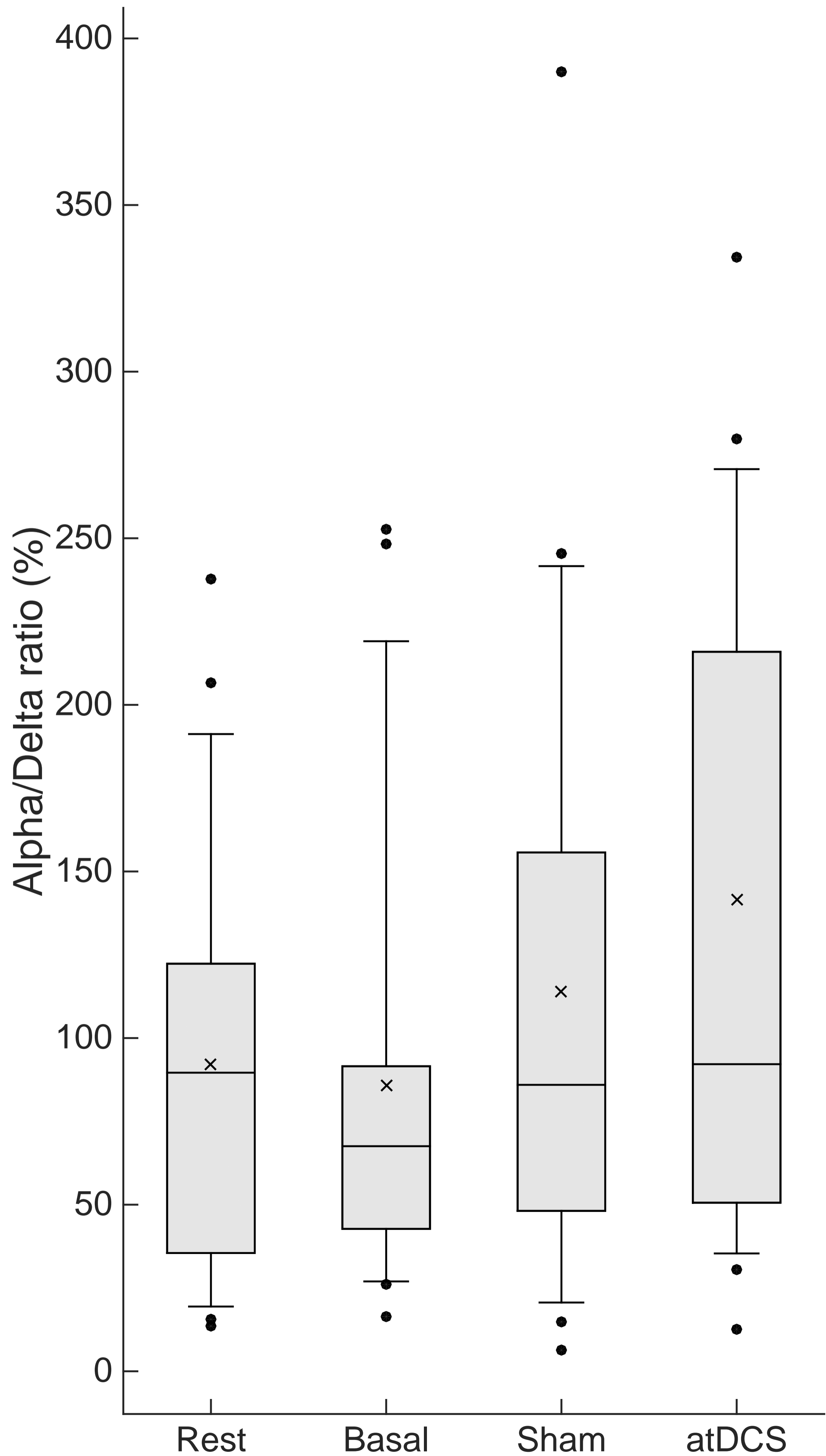

Supplement: Supplementary file 1 [file Data_Sheet_1.zip › Complementary_results/Band_ratios_Complete_EEG/Alpha_Delta/Alpha-Delta_complete-EEG_Avg AF3-F3-F7.pdf]

**Alpha/Delta ratio on complete EEG signal for electrode: Avg AF4-F4-F8**

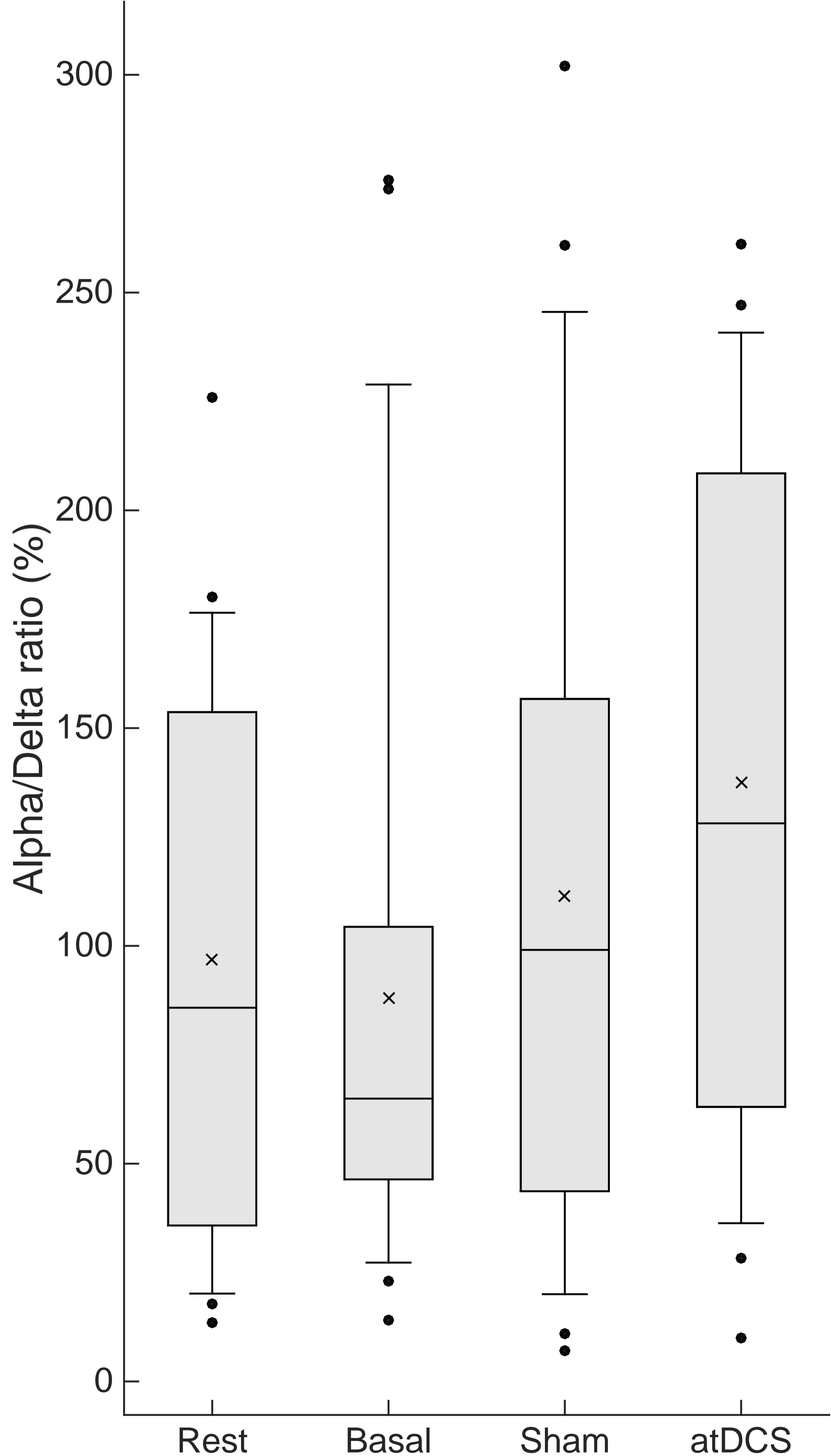

Supplement: Supplementary file 1 [file Data_Sheet_1.zip › Complementary_results/Band_ratios_Complete_EEG/Alpha_Delta/Alpha-Delta_complete-EEG_Avg AF4-F4-F8.pdf]

**Alpha/Delta ratio on complete EEG signal for electrode: Avg F3-F7-FC5**

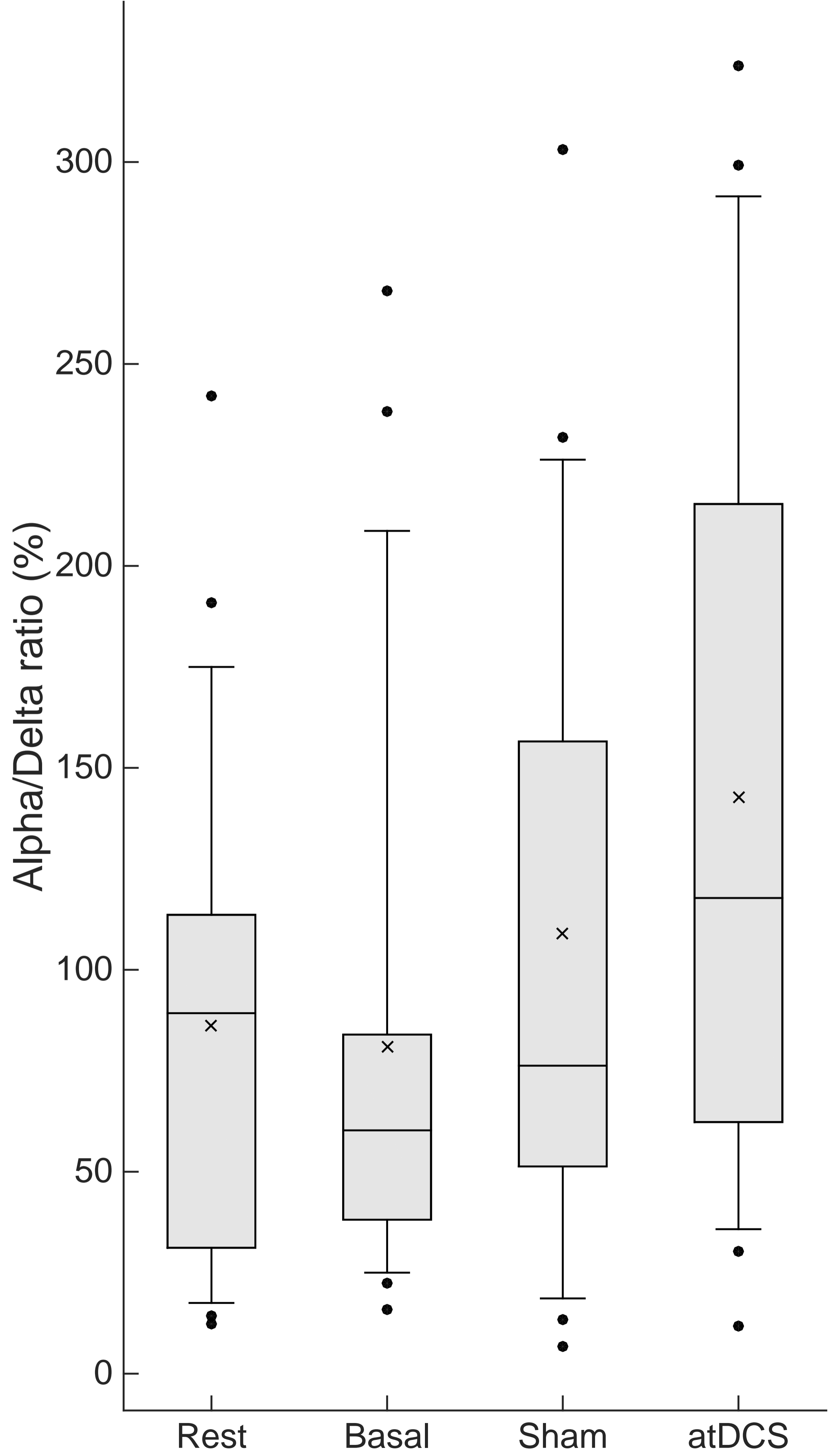

Supplement: Supplementary file 1 [file Data_Sheet_1.zip › Complementary_results/Band_ratios_Complete_EEG/Alpha_Delta/Alpha-Delta_complete-EEG_Avg F3-F7-FC5.pdf]

Alpha/Delta ratio on complete EEG signal for electrode: Avg F4-F8-FC6

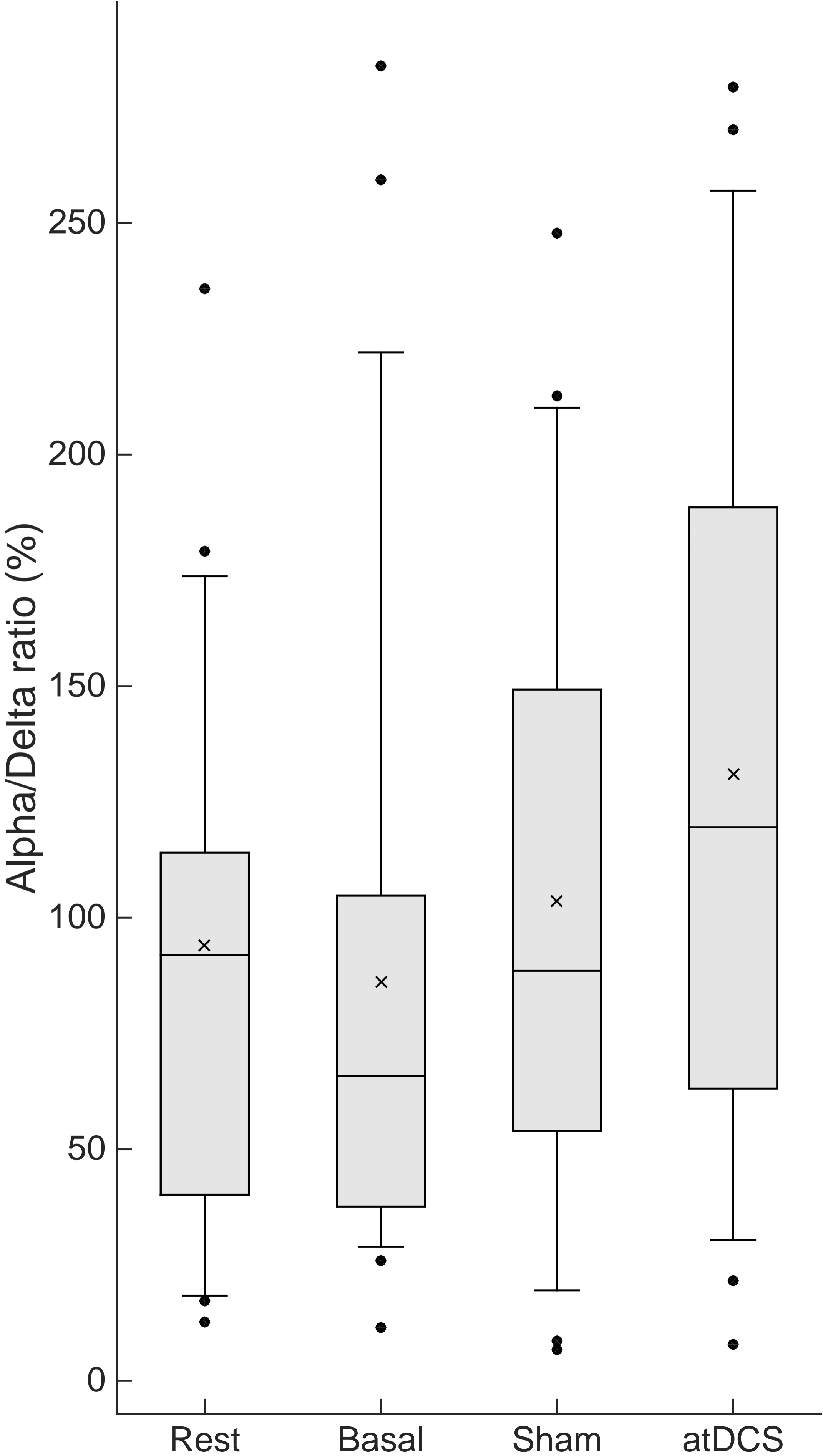

Supplement: Supplementary file 1 [file Data_Sheet_1.zip › Complementary_results/Band_ratios_Complete_EEG/Alpha_Delta/Alpha-Delta_complete-EEG_Avg F4-F8-FC6.pdf]

Alpha/Delta ratio on complete EEG signal for electrode: F3

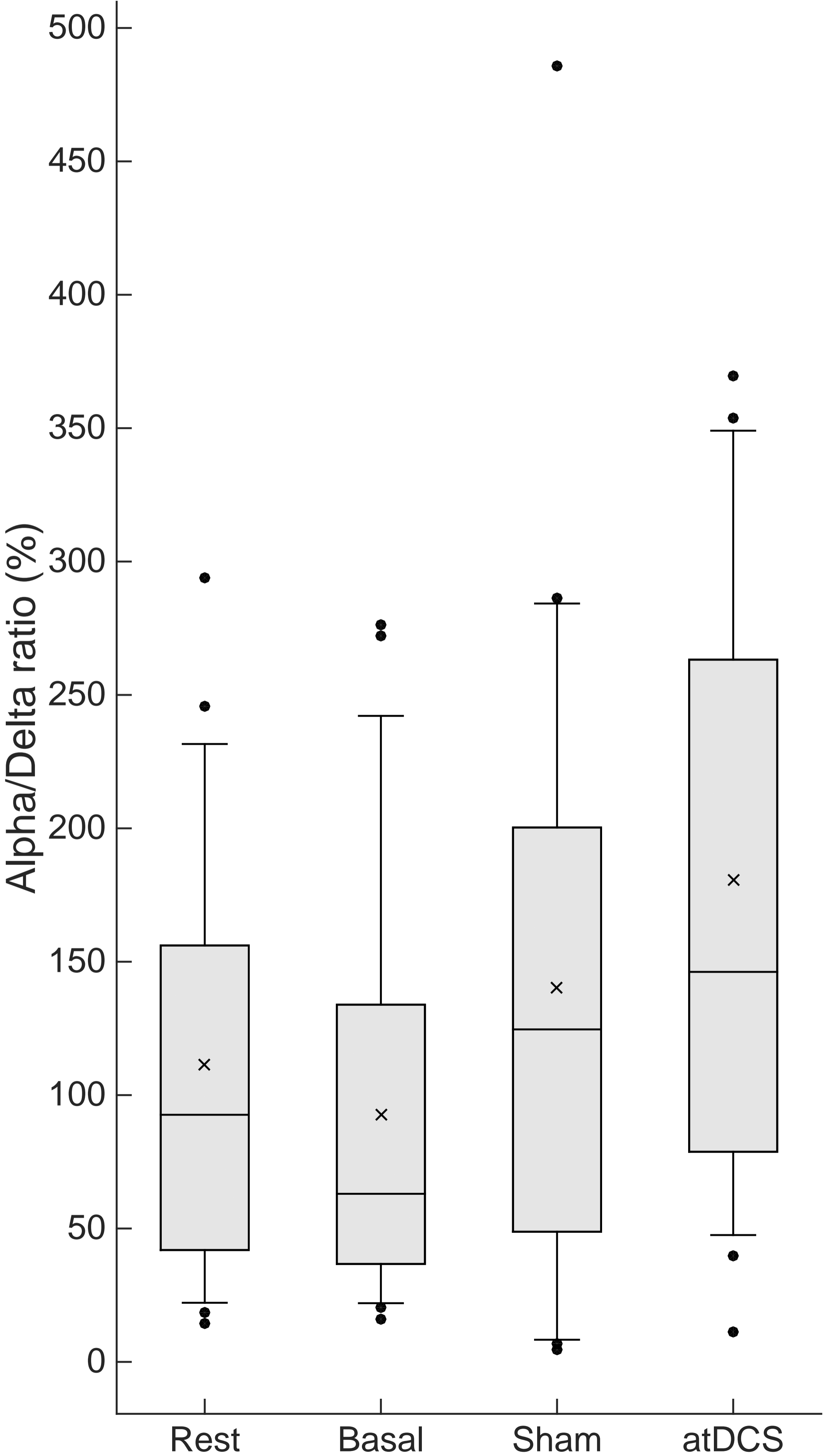

Supplement: Supplementary file 1 [file Data_Sheet_1.zip › Complementary_results/Band_ratios_Complete_EEG/Alpha_Delta/Alpha-Delta_complete-EEG_F3.pdf]

Alpha/Delta ratio on complete EEG signal for electrode: F4

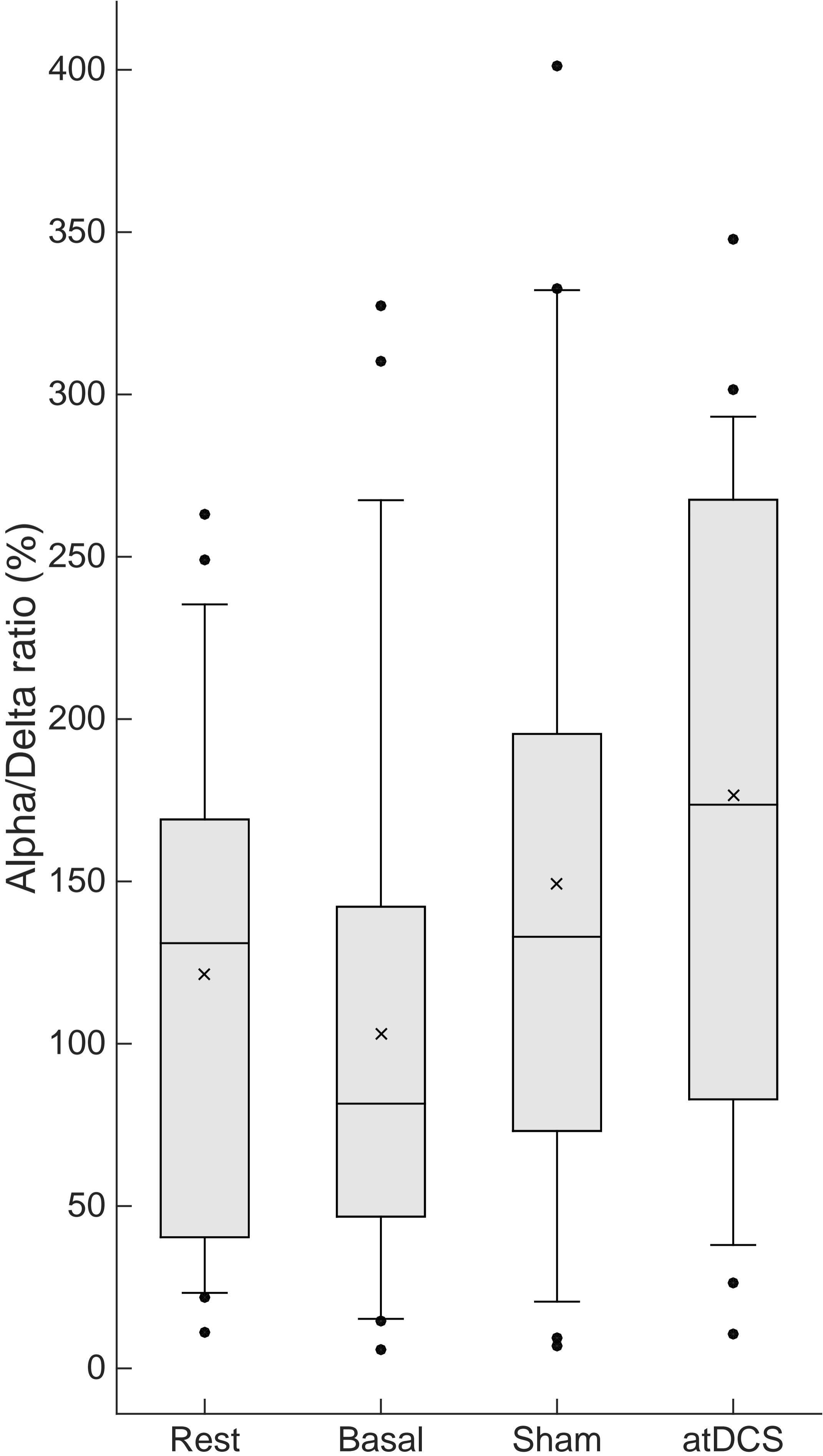

Supplement: Supplementary file 1 [file Data_Sheet_1.zip › Complementary_results/Band_ratios_Complete_EEG/Alpha_Delta/Alpha-Delta_complete-EEG_F4.pdf]

Alpha/Delta ratio on complete EEG signal for electrode: F7

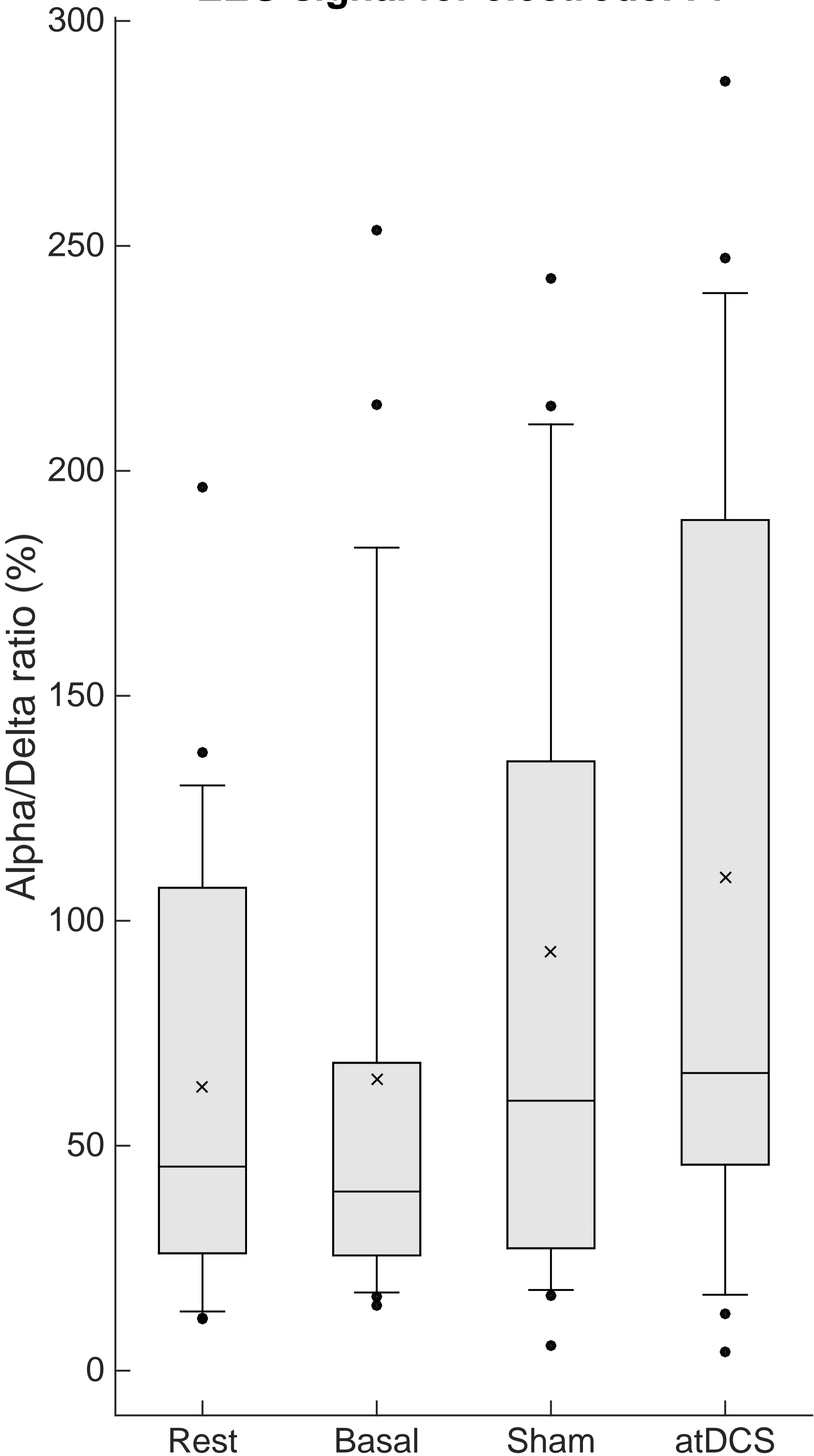

Supplement: Supplementary file 1 [file Data_Sheet_1.zip › Complementary_results/Band_ratios_Complete_EEG/Alpha_Delta/Alpha-Delta_complete-EEG_F7.pdf]

Alpha/Delta ratio on complete EEG signal for electrode: F8

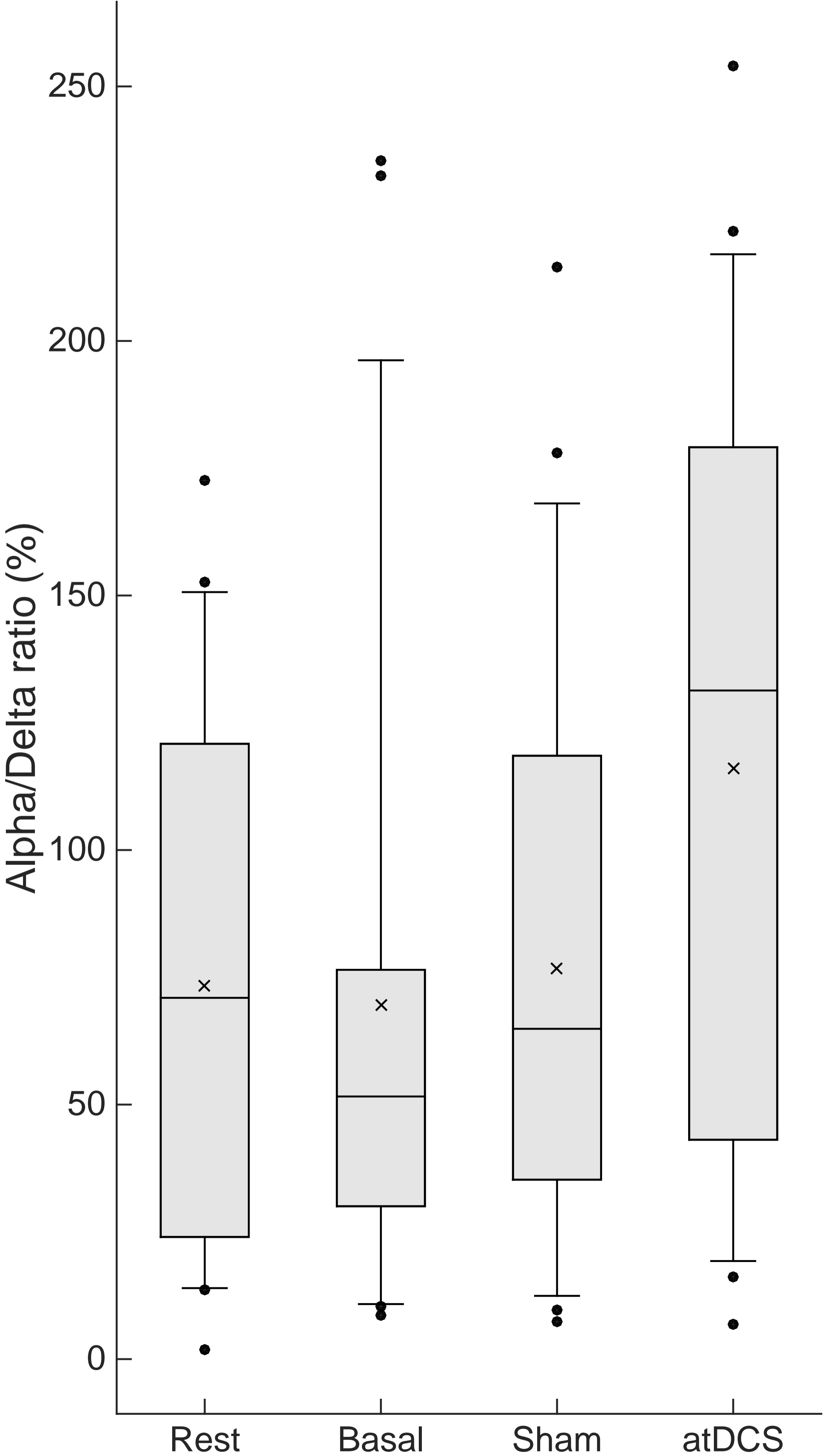

Supplement: Supplementary file 1 [file Data_Sheet_1.zip › Complementary_results/Band_ratios_Complete_EEG/Alpha_Delta/Alpha-Delta_complete-EEG_F8.pdf]

Alpha/Delta ratio on complete EEG signal for electrode: FC5

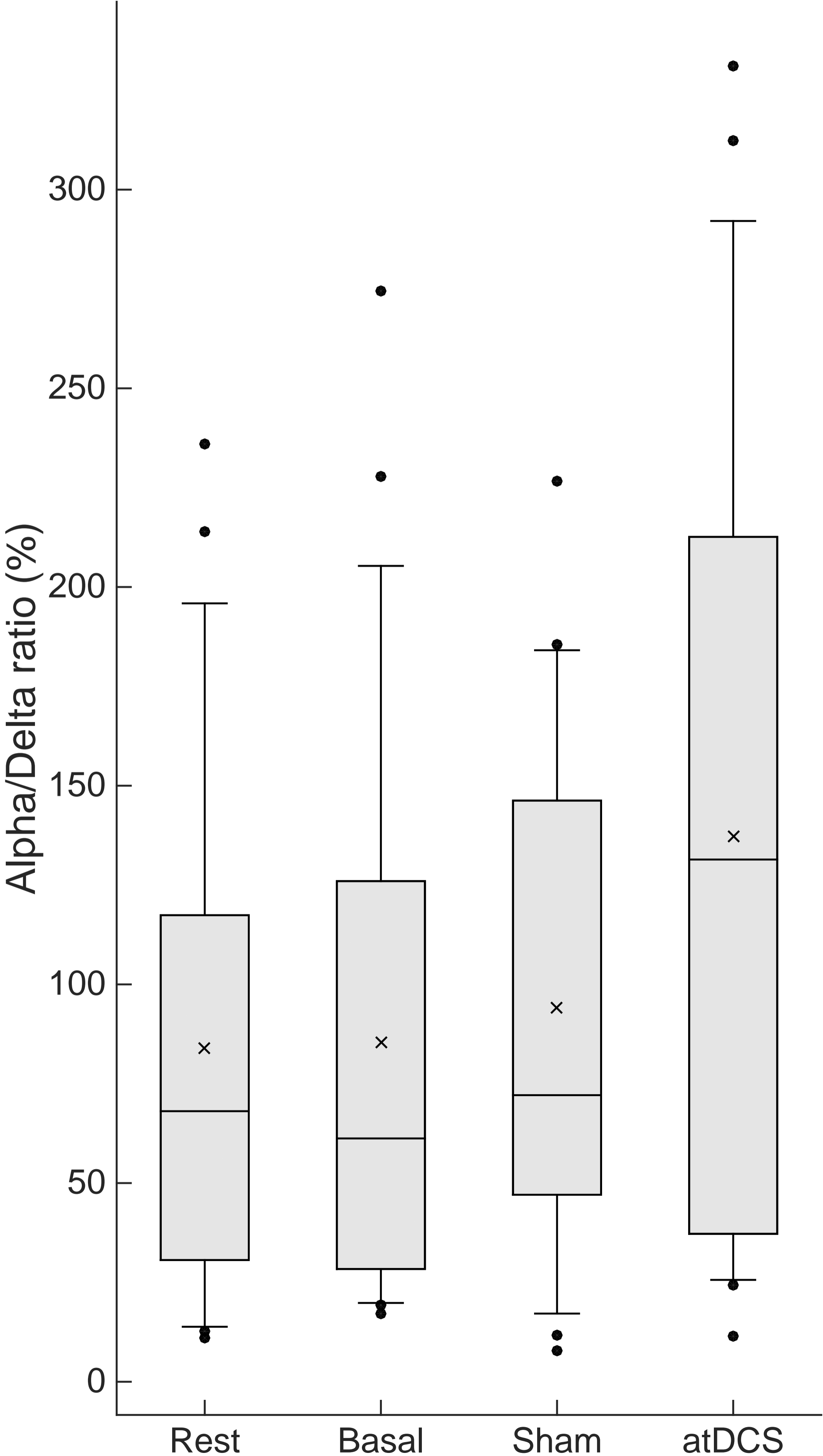

Supplement: Supplementary file 1 [file Data_Sheet_1.zip › Complementary_results/Band_ratios_Complete_EEG/Alpha_Delta/Alpha-Delta_complete-EEG_FC5.pdf]

Alpha/Delta ratio on complete EEG signal for electrode: FC6

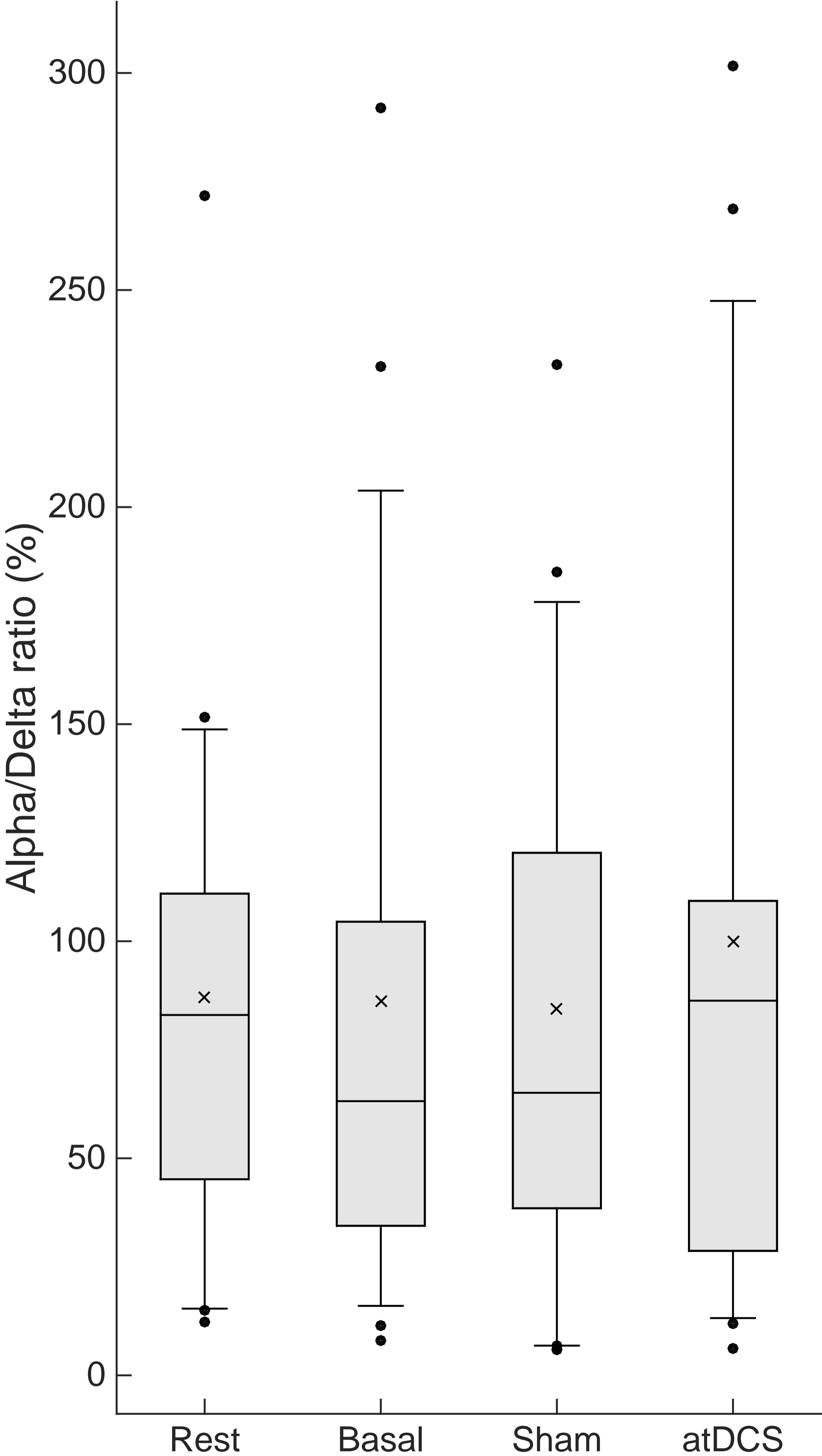

Supplement: Supplementary file 1 [file Data_Sheet_1.zip › Complementary_results/Band_ratios_Complete_EEG/Alpha_Delta/Alpha-Delta_complete-EEG_FC6.pdf]

Alpha/Delta ratio on complete EEG signal for electrode: O1

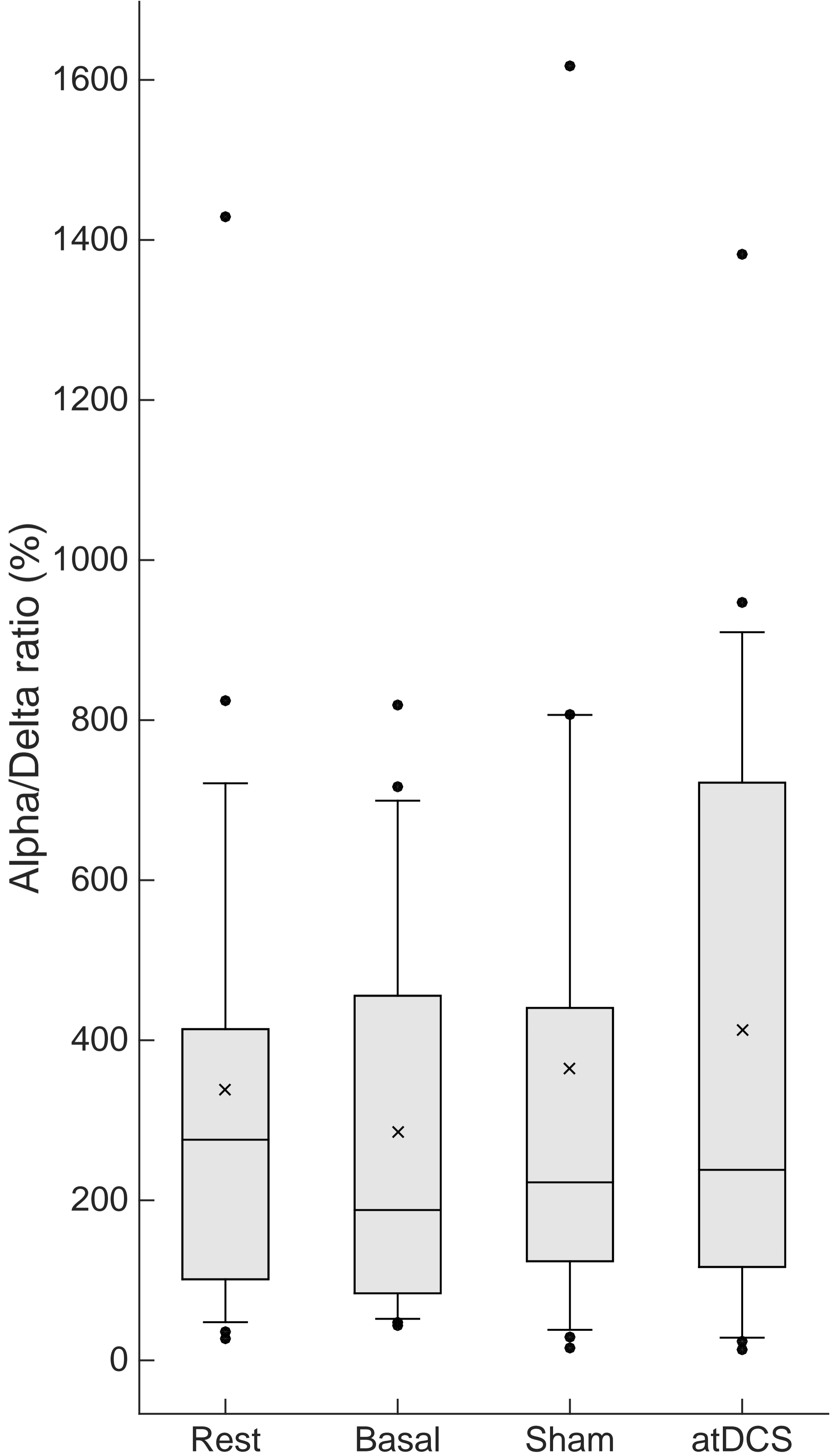

Supplement: Supplementary file 1 [file Data_Sheet_1.zip › Complementary_results/Band_ratios_Complete_EEG/Alpha_Delta/Alpha-Delta_complete-EEG_O1.pdf]

Alpha/Delta ratio on complete EEG signal for electrode: O2

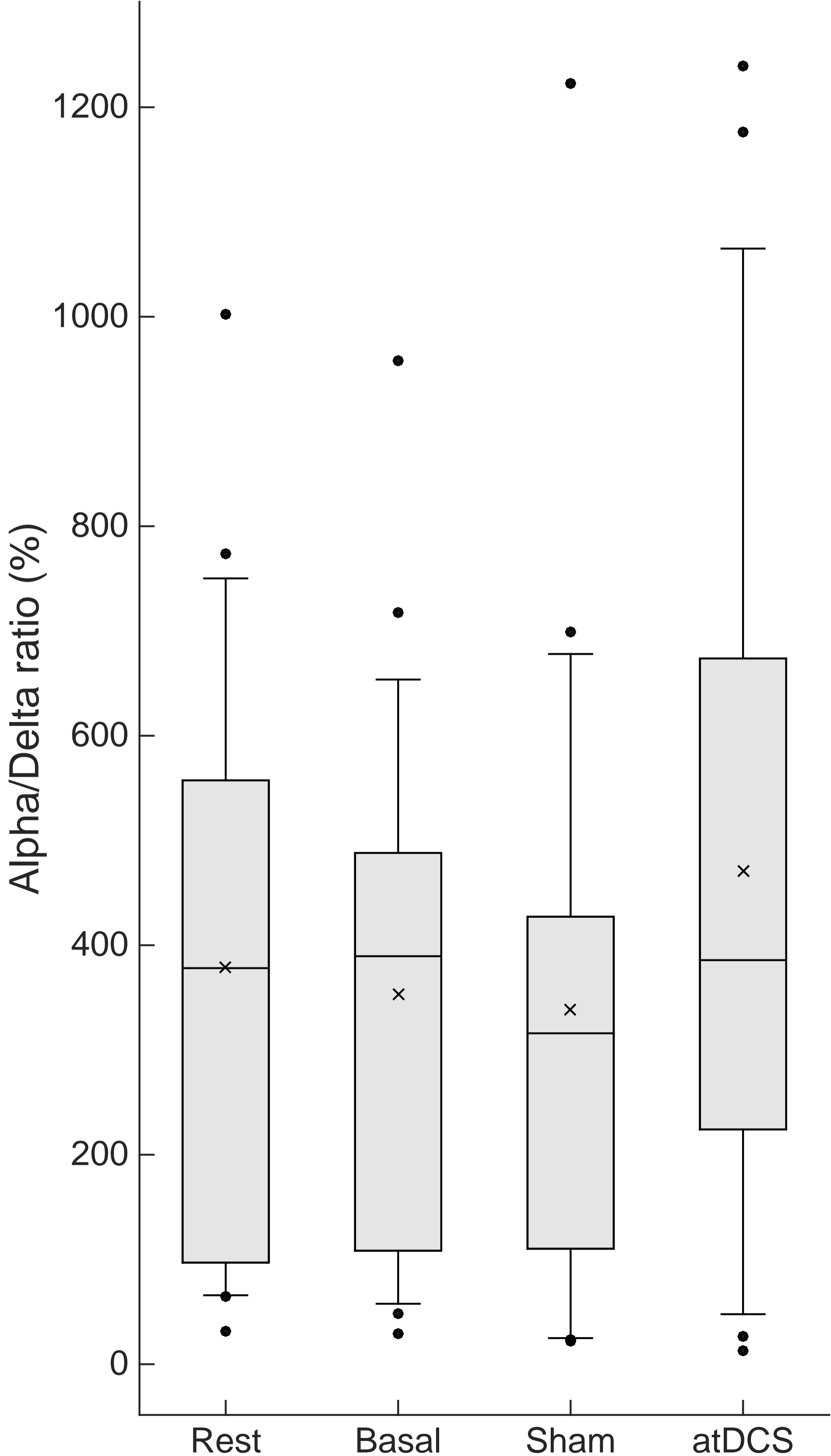

Supplement: Supplementary file 1 [file Data_Sheet_1.zip › Complementary_results/Band_ratios_Complete_EEG/Alpha_Delta/Alpha-Delta_complete-EEG_O2.pdf]

Alpha/Delta ratio on complete EEG signal for electrode: P7

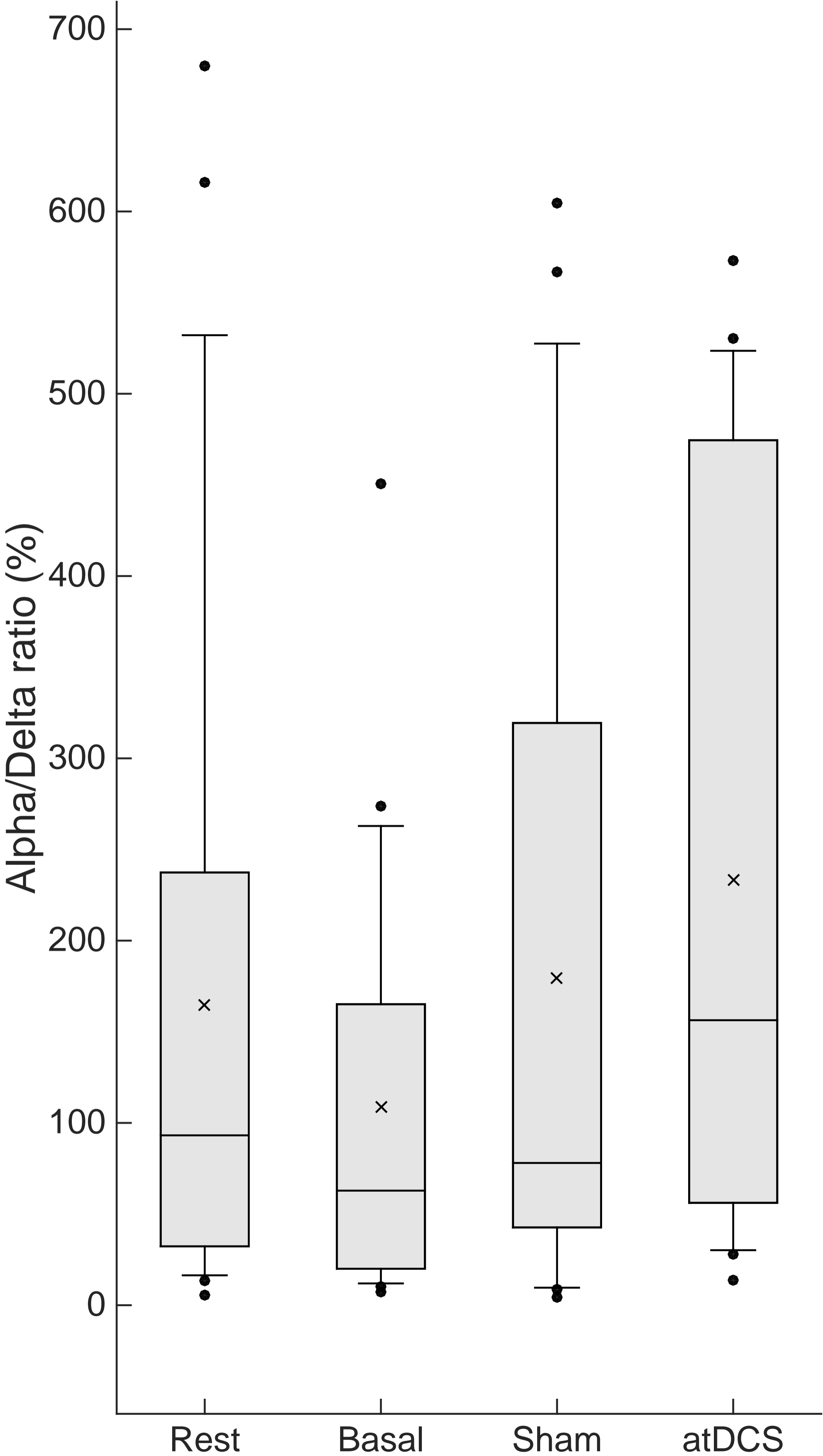

Supplement: Supplementary file 1 [file Data_Sheet_1.zip › Complementary_results/Band_ratios_Complete_EEG/Alpha_Delta/Alpha-Delta_complete-EEG_P7.pdf]

Alpha/Delta ratio on complete EEG signal for electrode: P8

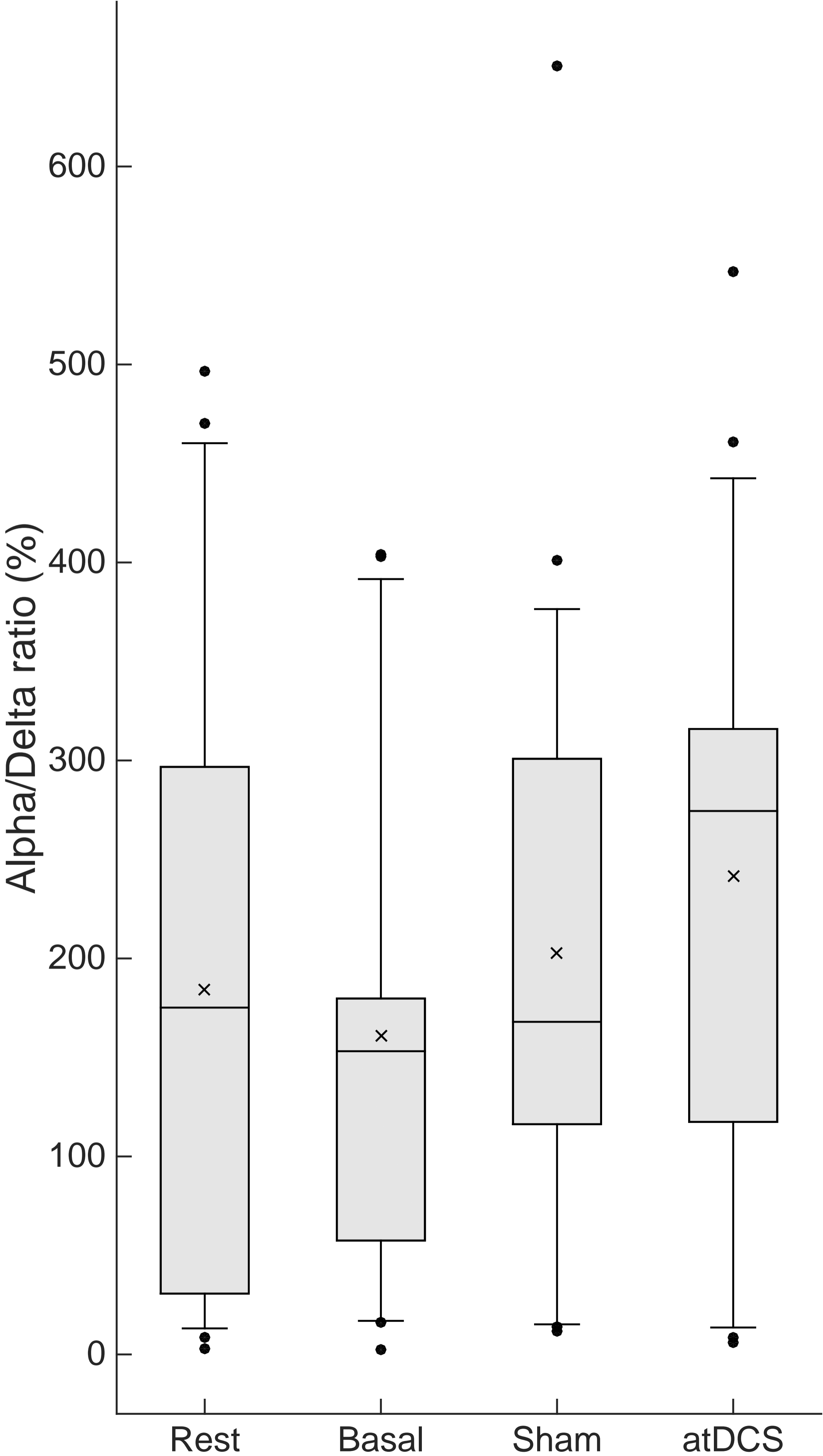

Supplement: Supplementary file 1 [file Data_Sheet_1.zip › Complementary_results/Band_ratios_Complete_EEG/Alpha_Delta/Alpha-Delta_complete-EEG_P8.pdf]

Alpha/Delta ratio on complete EEG signal for electrode: T7

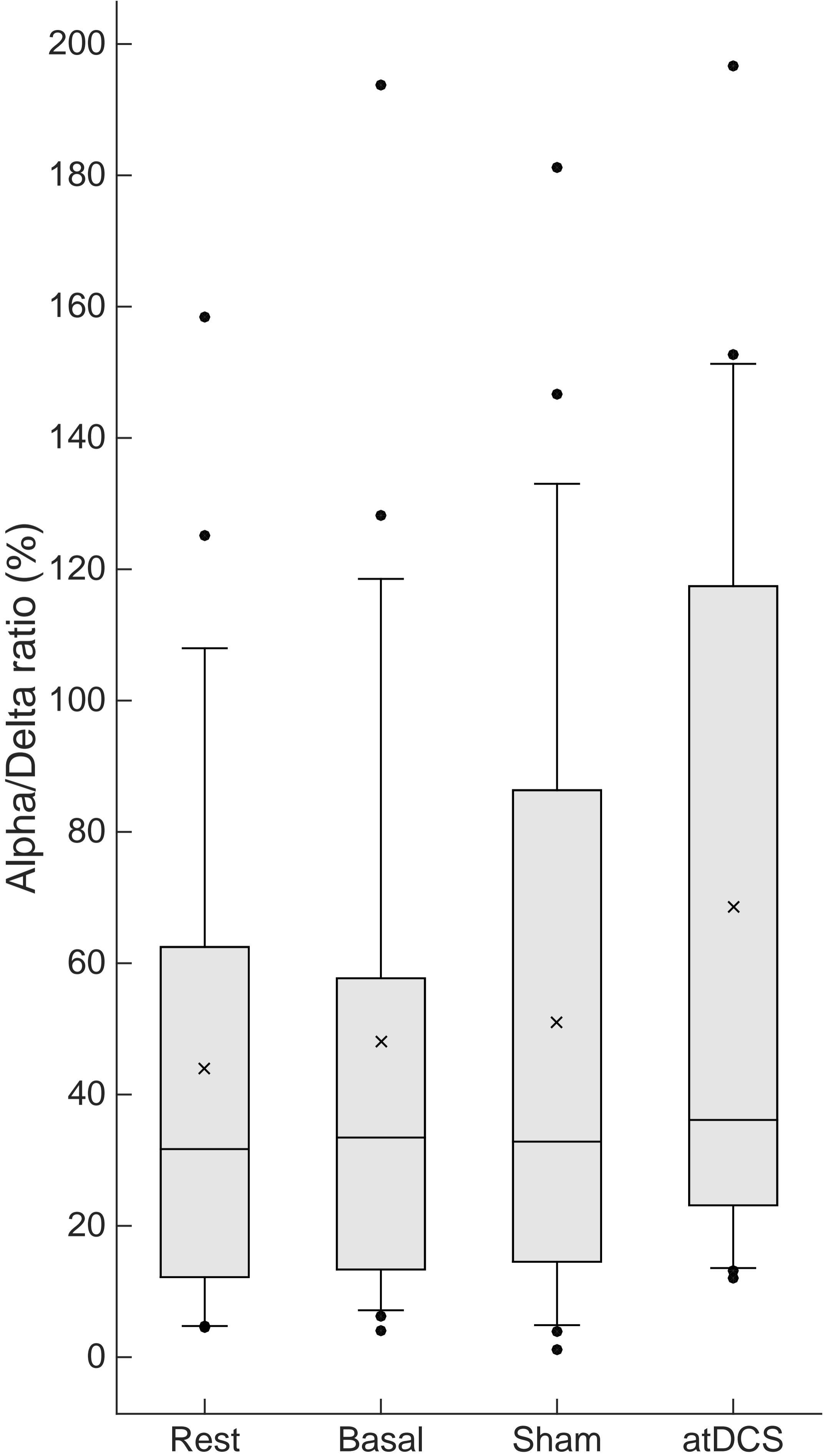

Supplement: Supplementary file 1 [file Data_Sheet_1.zip › Complementary_results/Band_ratios_Complete_EEG/Alpha_Delta/Alpha-Delta_complete-EEG_T7.pdf]

Alpha/Delta ratio on complete EEG signal for electrode: T8

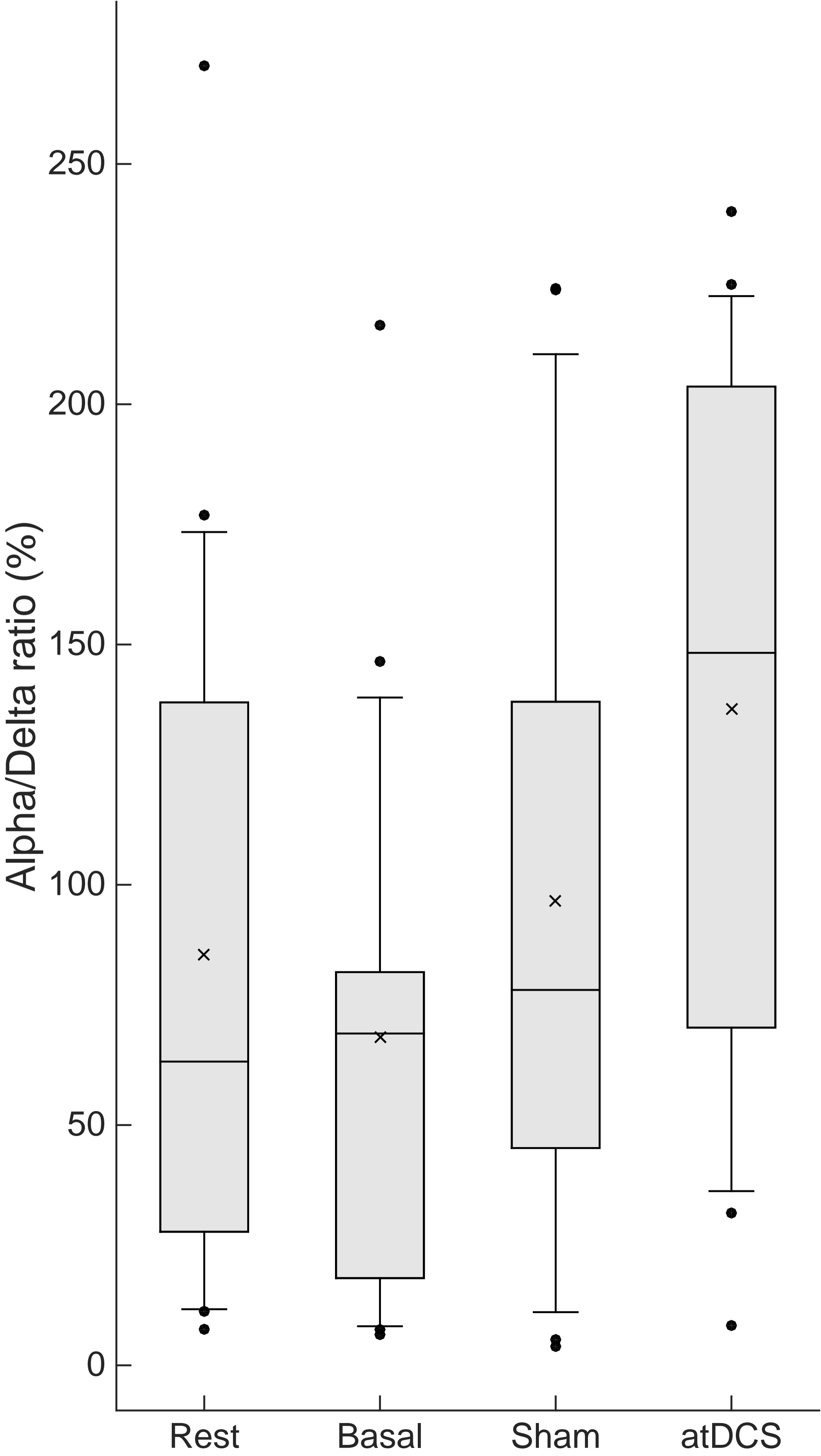

Supplement: Supplementary file 1 [file Data_Sheet_1.zip › Complementary_results/Band_ratios_Complete_EEG/Alpha_Delta/Alpha-Delta_complete-EEG_T8.pdf]

**Beta/Alpha ratio on complete EEG signal for electrode: AF3**

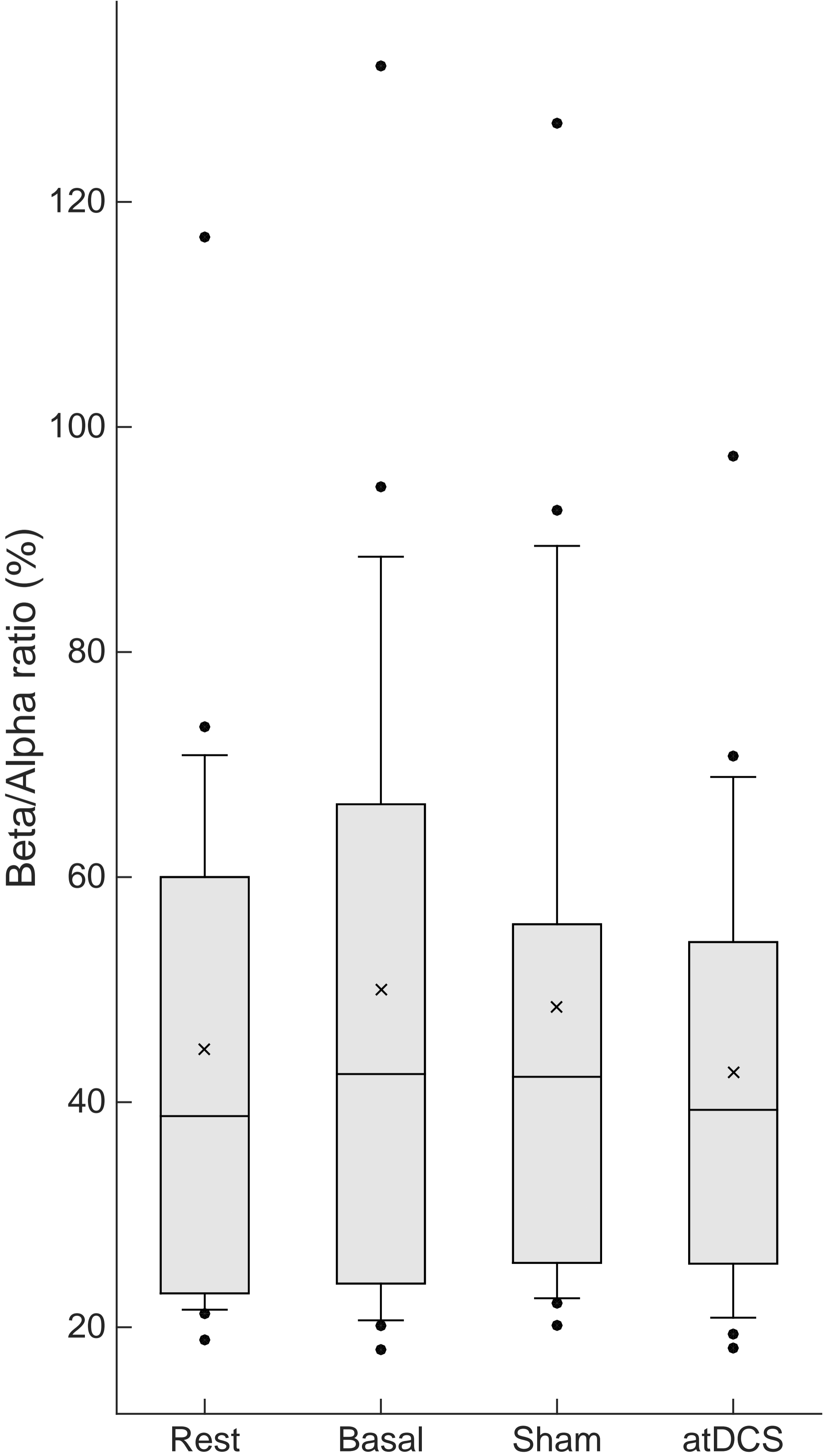

Supplement: Supplementary file 1 [file Data_Sheet_1.zip › Complementary_results/Band_ratios_Complete_EEG/Beta_Alpha/Beta-Alpha_complete-EEG_AF3.pdf]

**Beta/Alpha ratio on complete EEG signal for electrode: AF4**

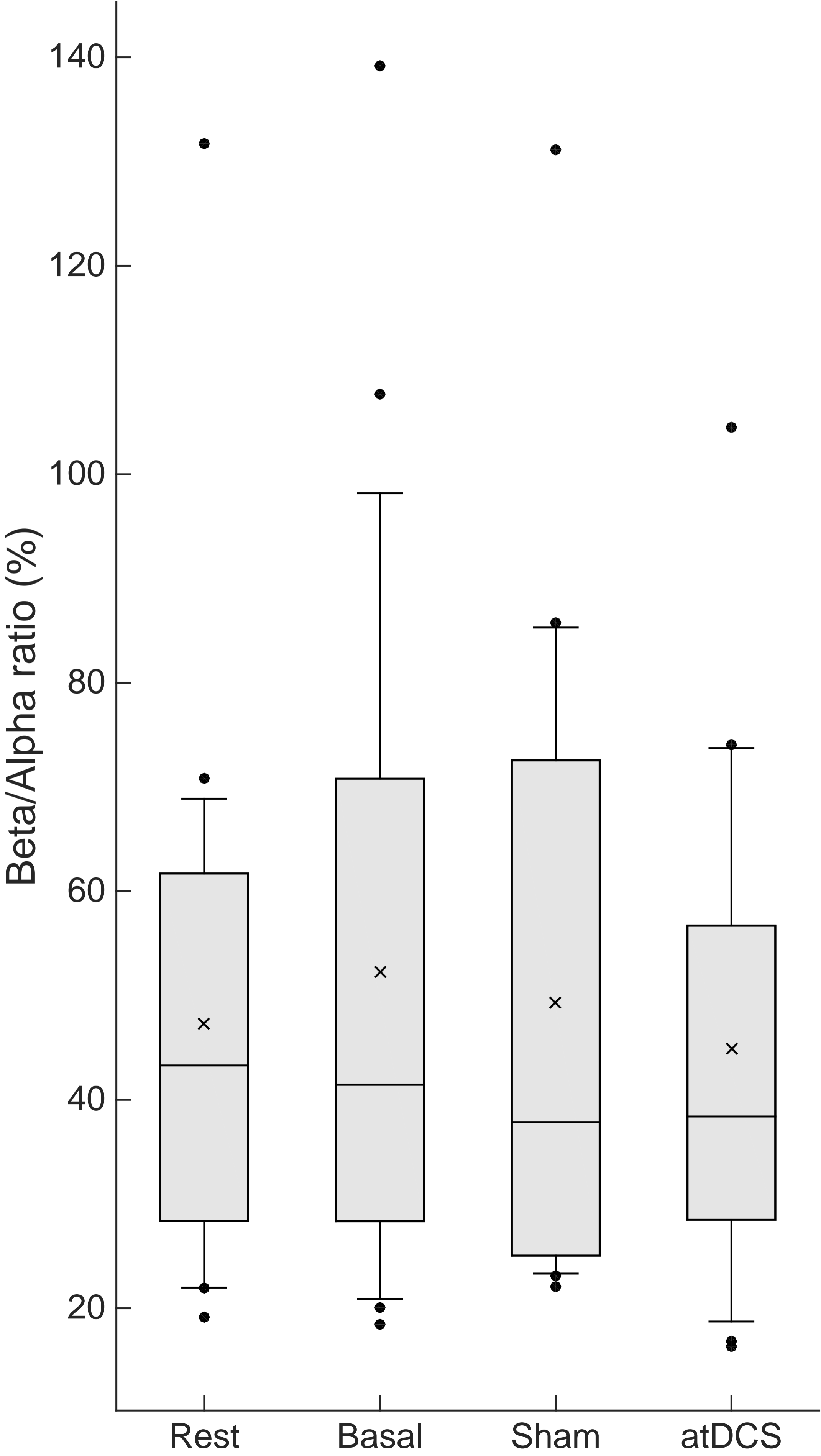

Supplement: Supplementary file 1 [file Data_Sheet_1.zip › Complementary_results/Band_ratios_Complete_EEG/Beta_Alpha/Beta-Alpha_complete-EEG_AF4.pdf]

**Beta/Alpha ratio on complete EEG signal for electrode: Avg AF3-F3-F7**

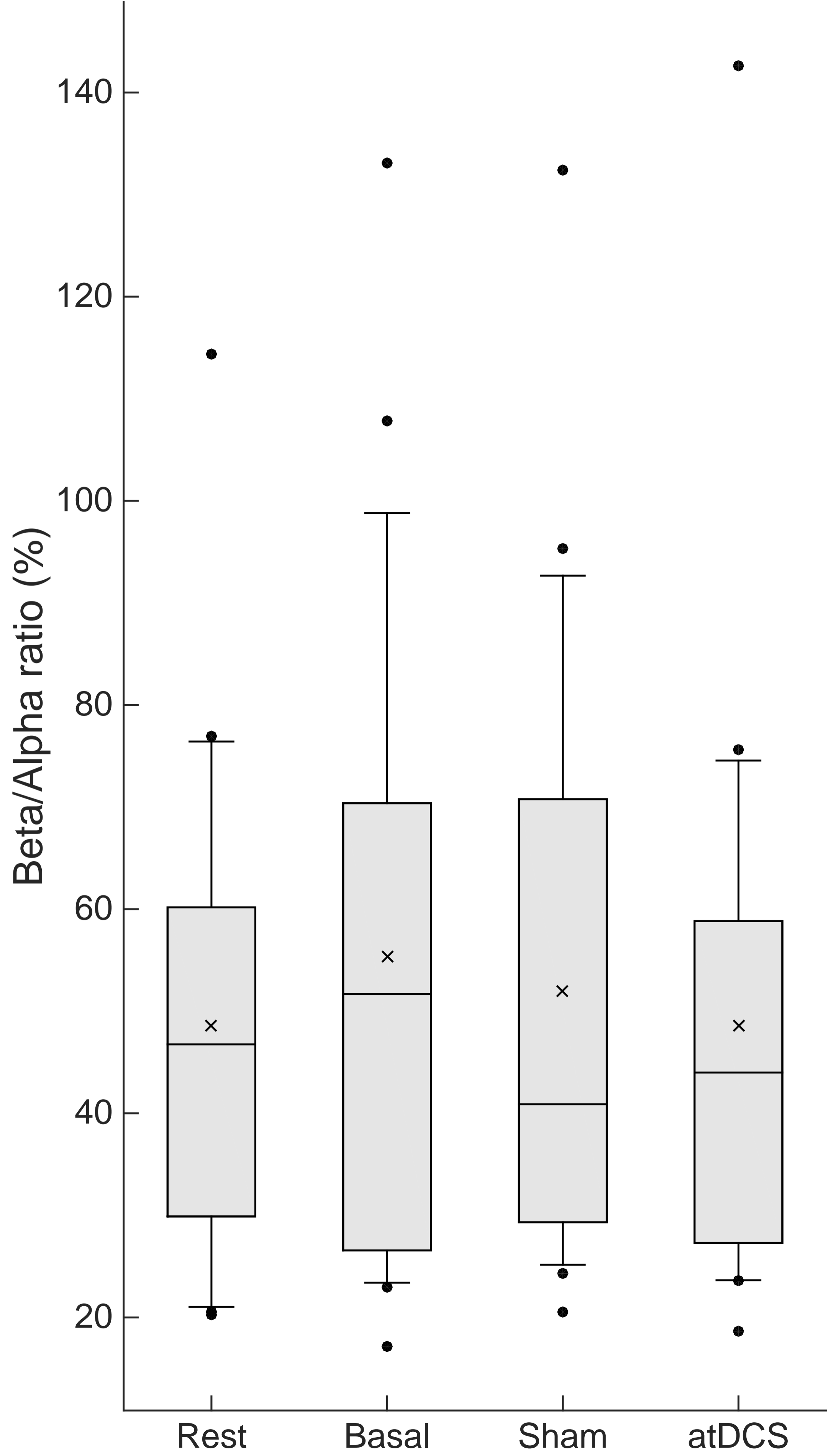

Supplement: Supplementary file 1 [file Data_Sheet_1.zip › Complementary_results/Band_ratios_Complete_EEG/Beta_Alpha/Beta-Alpha_complete-EEG_Avg AF3-F3-F7.pdf]

**Beta/Alpha ratio on complete EEG signal for electrode: Avg AF4-F4-F8**

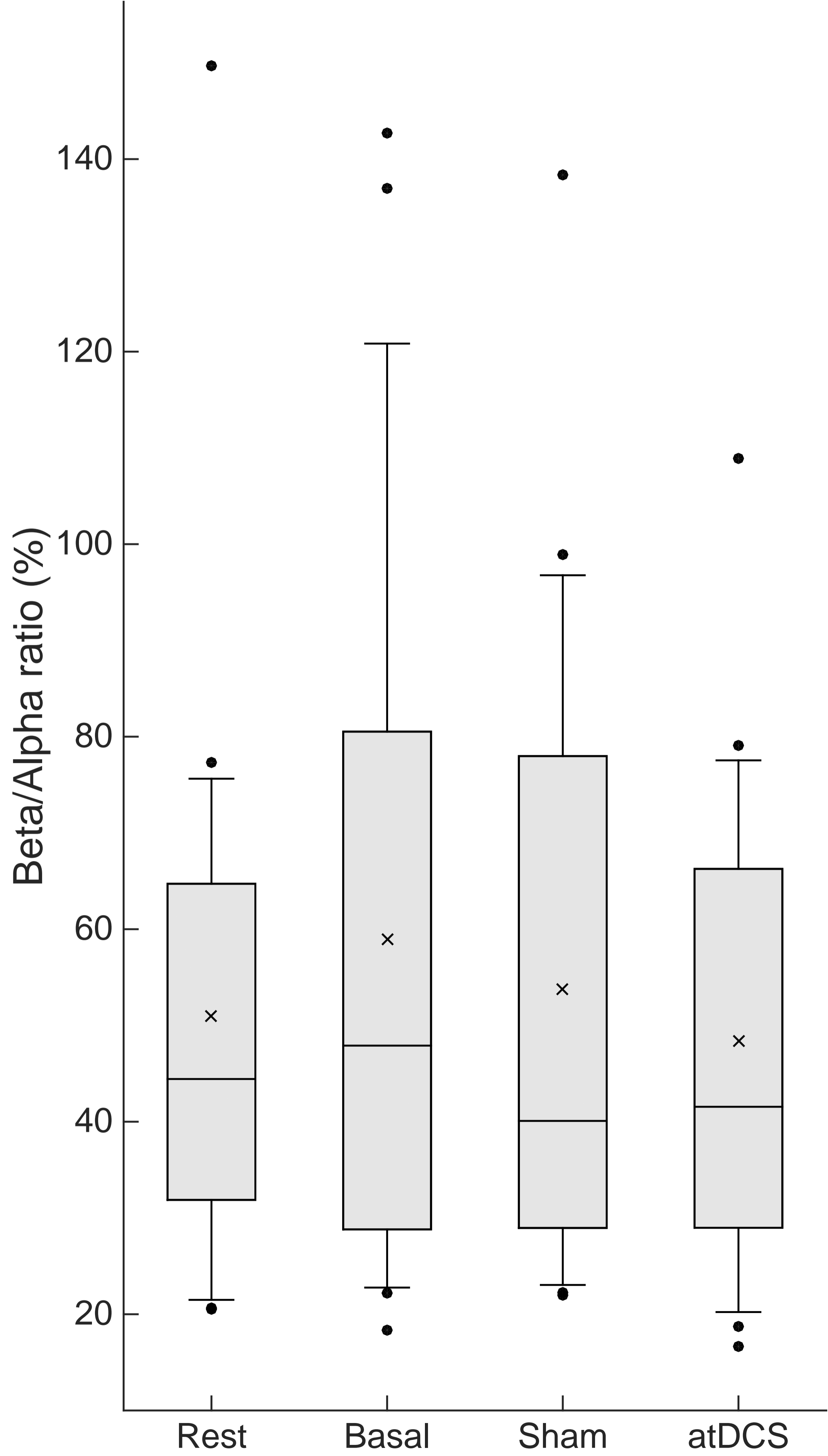

Supplement: Supplementary file 1 [file Data_Sheet_1.zip › Complementary_results/Band_ratios_Complete_EEG/Beta_Alpha/Beta-Alpha_complete-EEG_Avg AF4-F4-F8.pdf]

**Beta/Alpha ratio on complete EEG signal for electrode: Avg F3-F7-FC5**

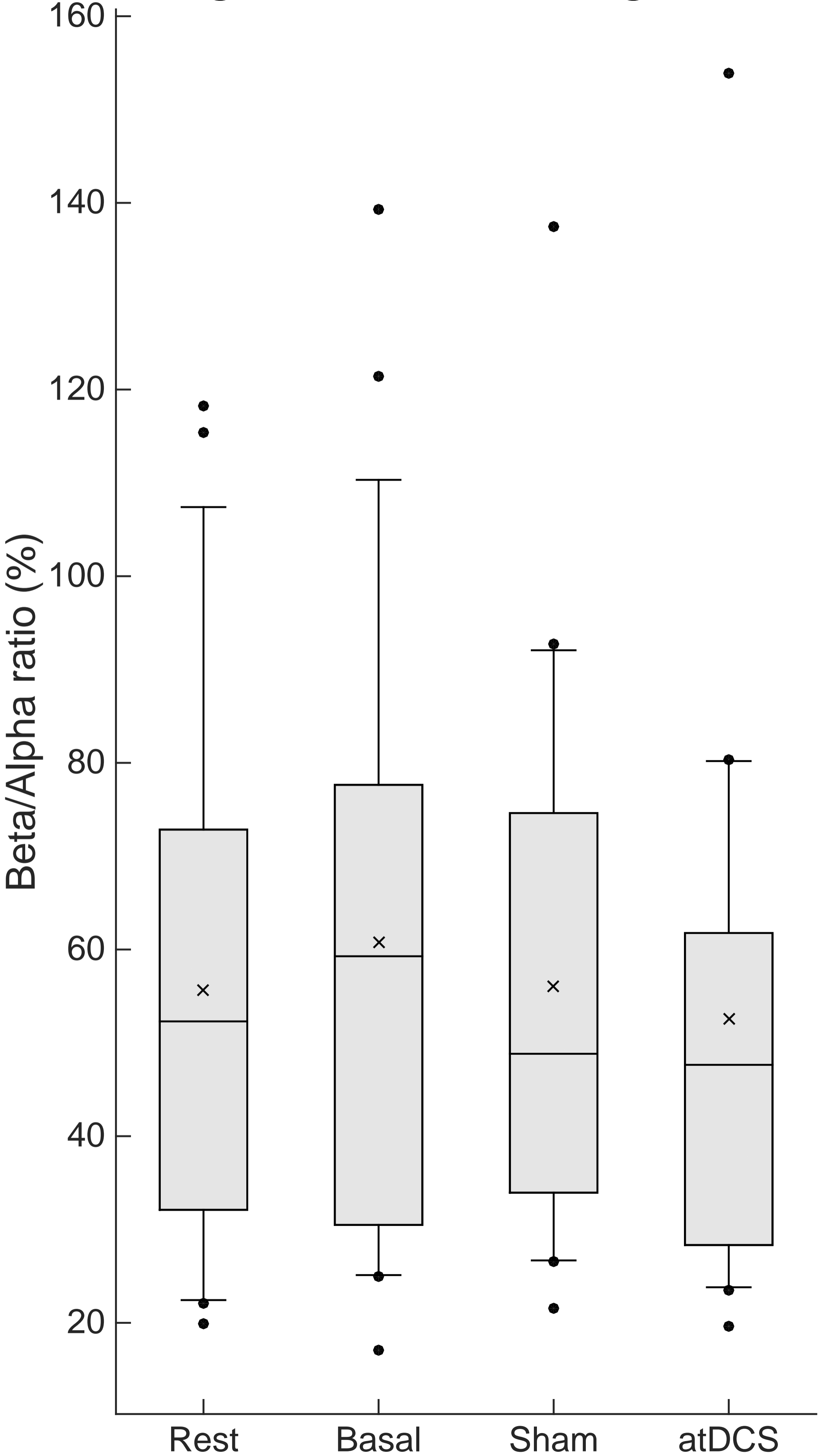

Supplement: Supplementary file 1 [file Data_Sheet_1.zip › Complementary_results/Band_ratios_Complete_EEG/Beta_Alpha/Beta-Alpha_complete-EEG_Avg F3-F7-FC5.pdf]

**Beta/Alpha ratio on complete EEG signal for electrode: Avg F4-F8-FC6**

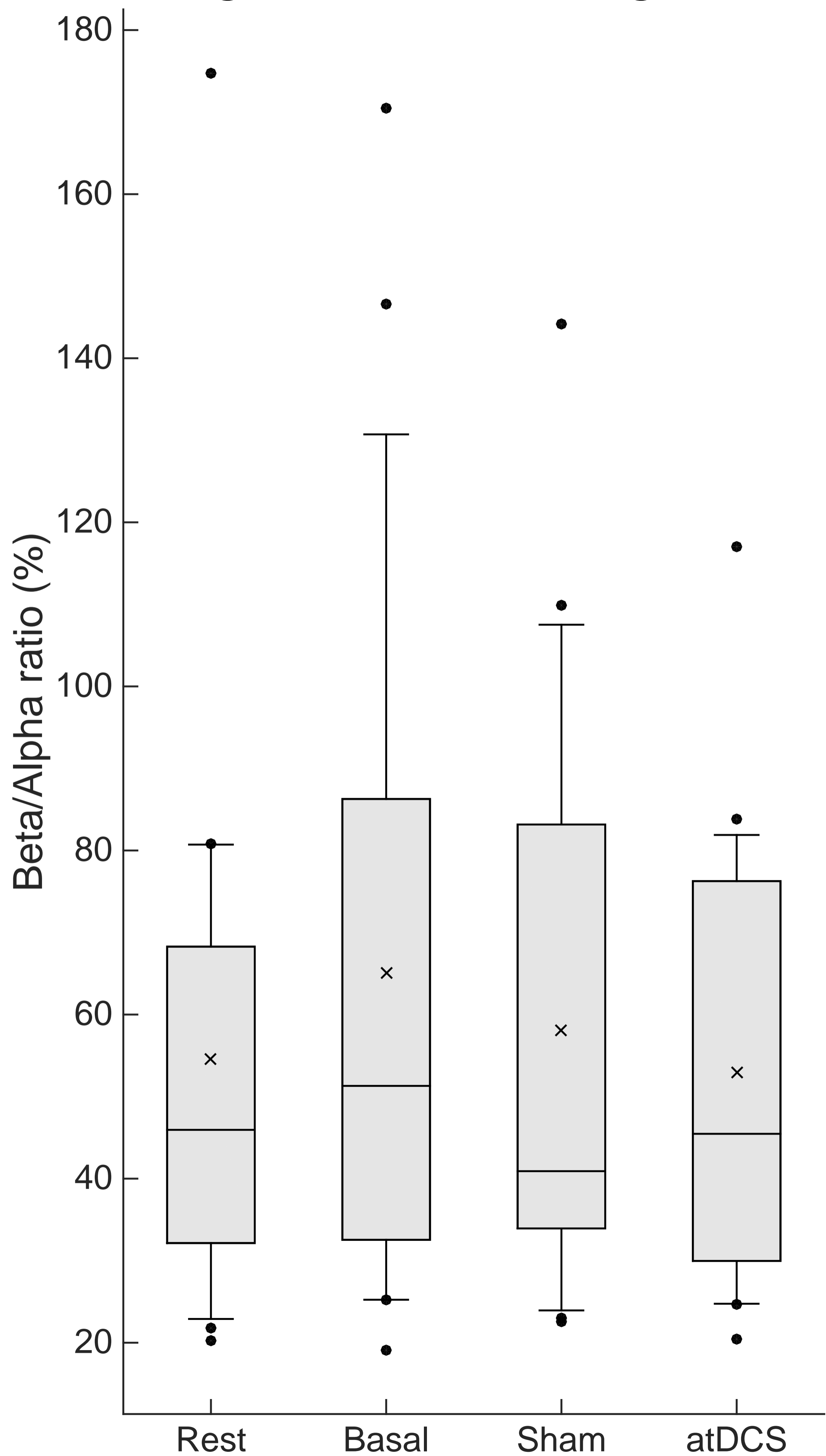

Supplement: Supplementary file 1 [file Data_Sheet_1.zip › Complementary_results/Band_ratios_Complete_EEG/Beta_Alpha/Beta-Alpha_complete-EEG_Avg F4-F8-FC6.pdf]

**Beta/Alpha ratio on complete EEG signal for electrode: F3**

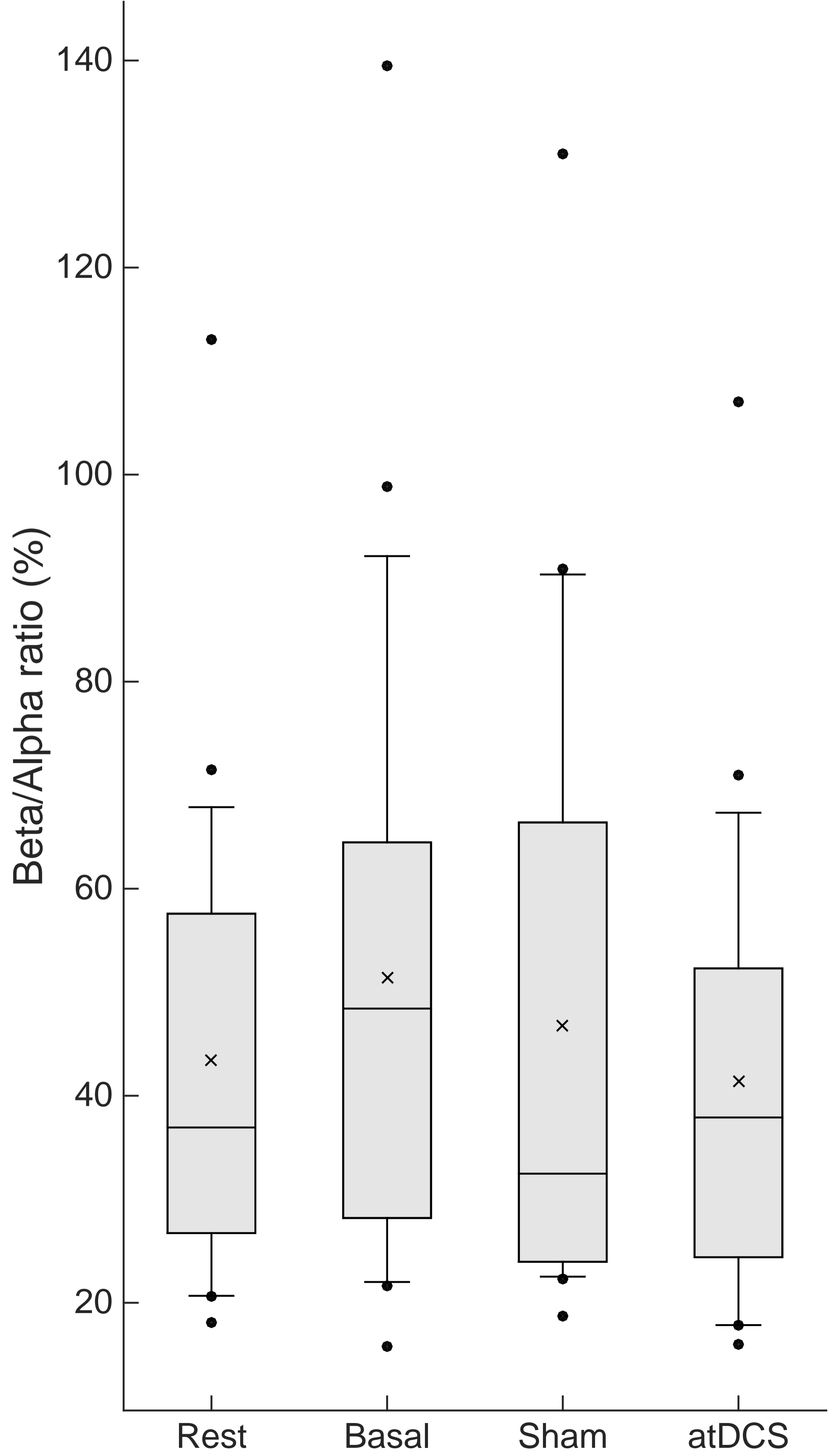

Supplement: Supplementary file 1 [file Data_Sheet_1.zip › Complementary_results/Band_ratios_Complete_EEG/Beta_Alpha/Beta-Alpha_complete-EEG_F3.pdf]

**Beta/Alpha ratio on complete EEG signal for electrode: F4**

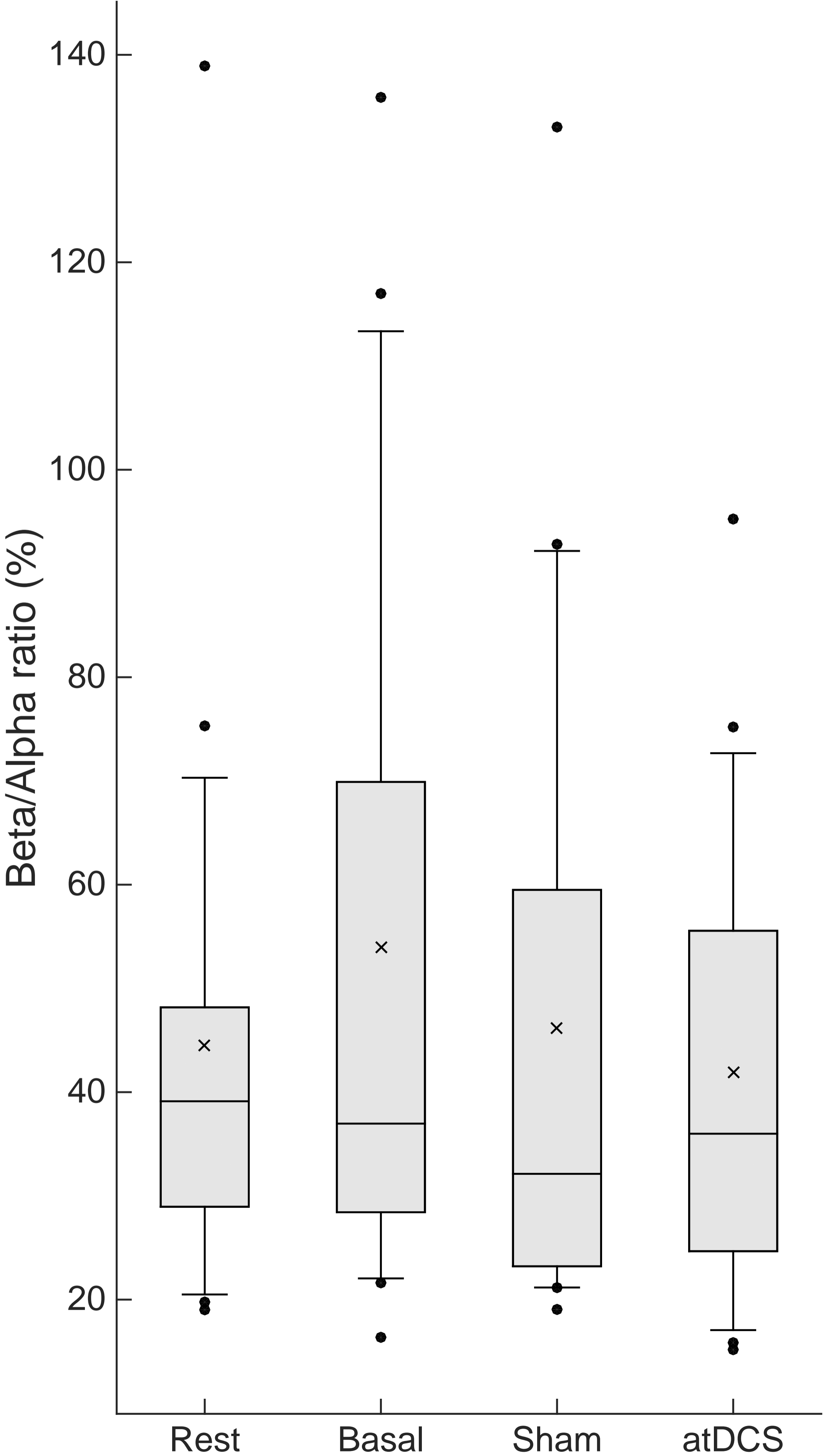

Supplement: Supplementary file 1 [file Data_Sheet_1.zip › Complementary_results/Band_ratios_Complete_EEG/Beta_Alpha/Beta-Alpha_complete-EEG_F4.pdf]

**Beta/Alpha ratio on complete EEG signal for electrode: F7**

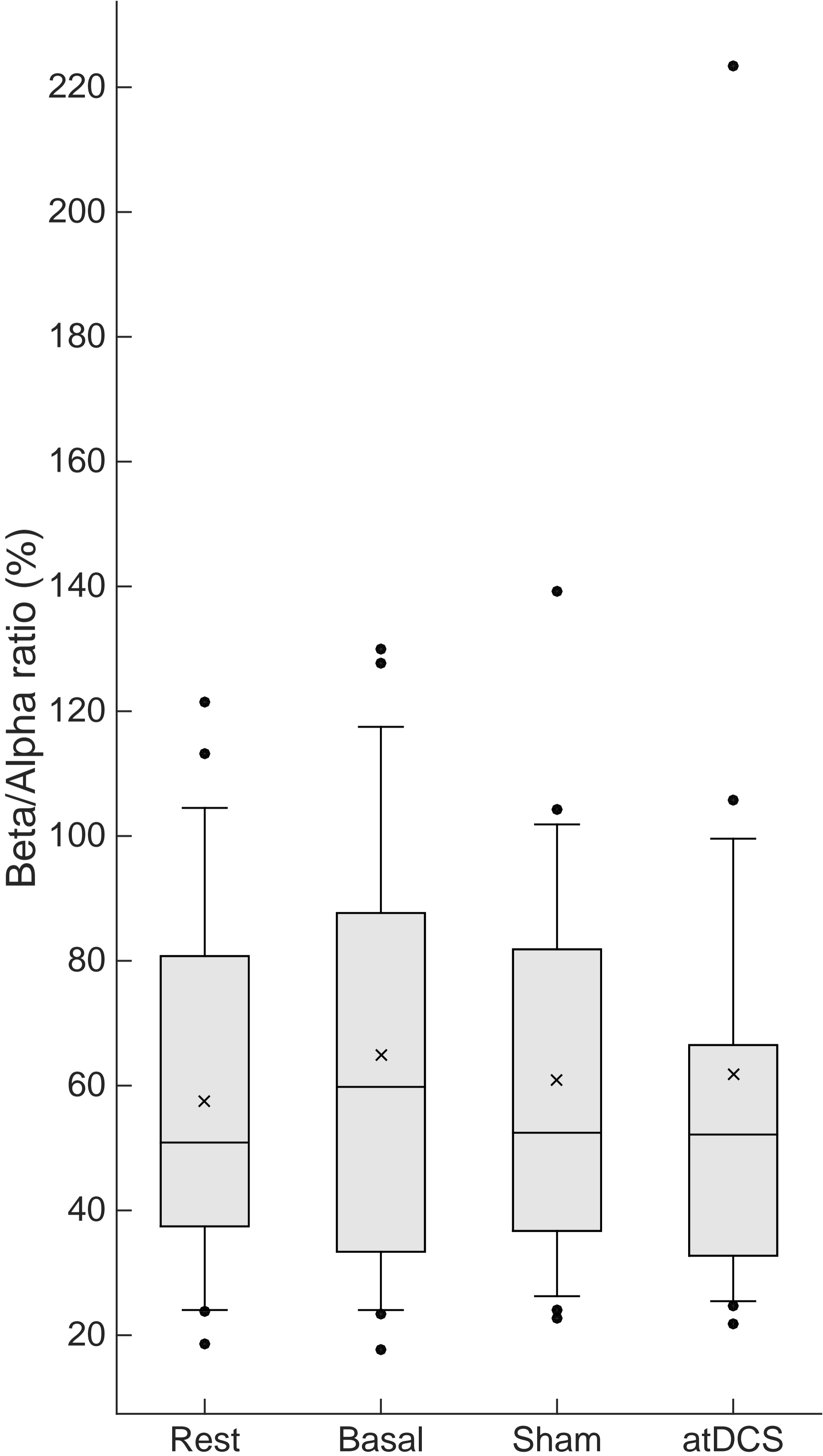

Supplement: Supplementary file 1 [file Data_Sheet_1.zip › Complementary_results/Band_ratios_Complete_EEG/Beta_Alpha/Beta-Alpha_complete-EEG_F7.pdf]

**Beta/Alpha ratio on complete EEG signal for electrode: F8**

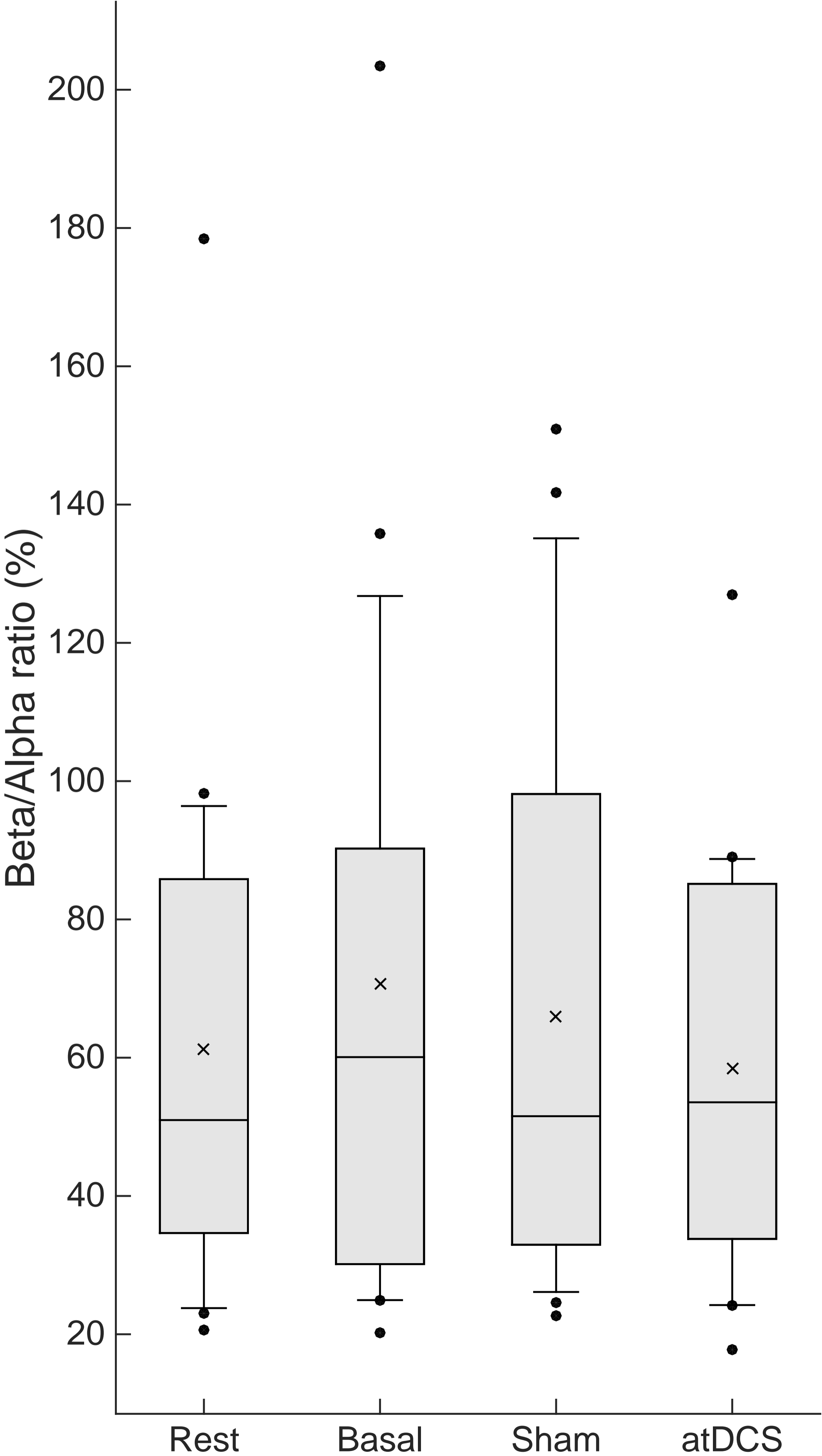

Supplement: Supplementary file 1 [file Data_Sheet_1.zip › Complementary_results/Band_ratios_Complete_EEG/Beta_Alpha/Beta-Alpha_complete-EEG_F8.pdf]

**Beta/Alpha ratio on complete EEG signal for electrode: FC5**

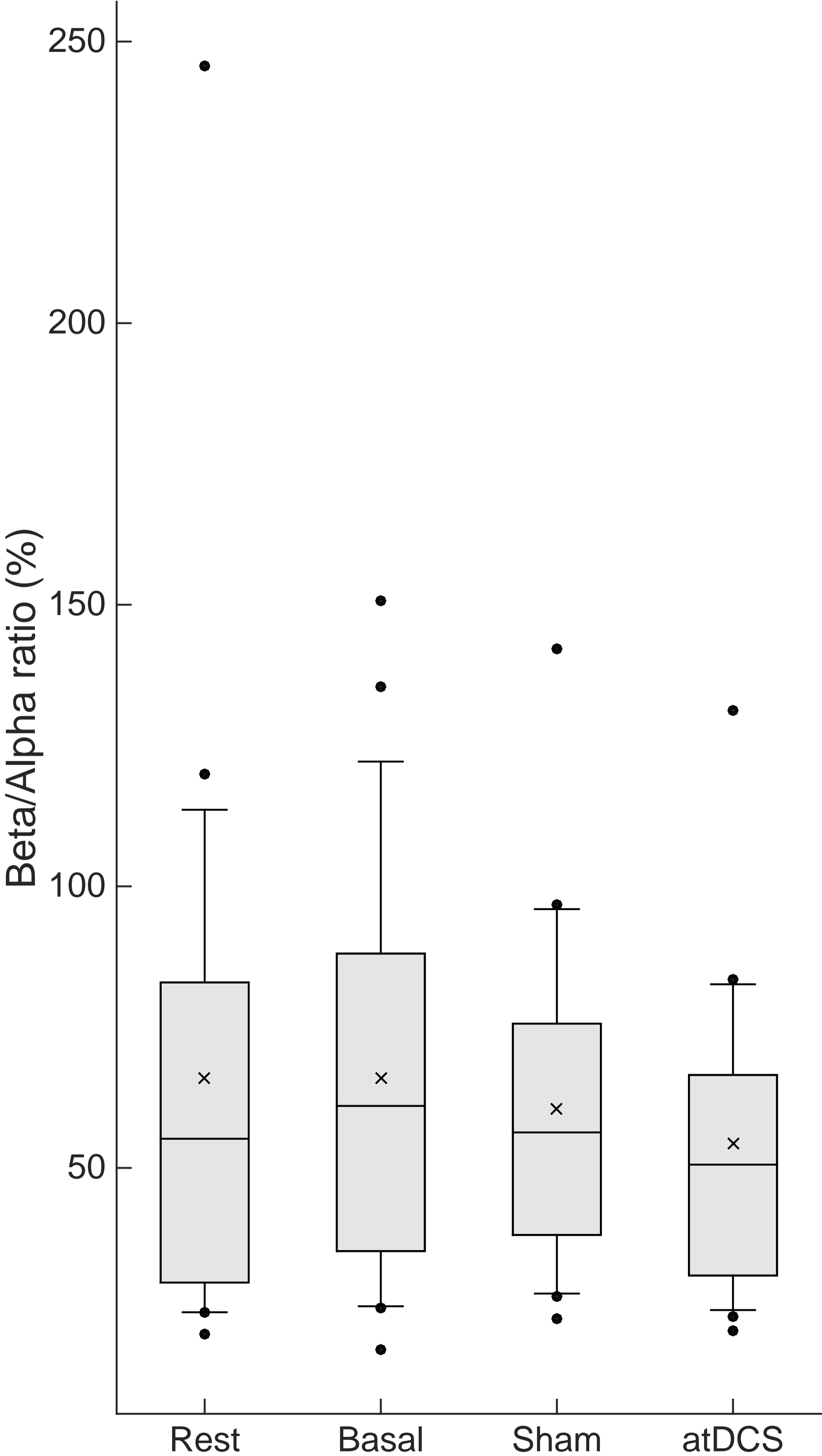

Supplement: Supplementary file 1 [file Data_Sheet_1.zip › Complementary_results/Band_ratios_Complete_EEG/Beta_Alpha/Beta-Alpha_complete-EEG_FC5.pdf]

**Beta/Alpha ratio on complete EEG signal for electrode: FC6**

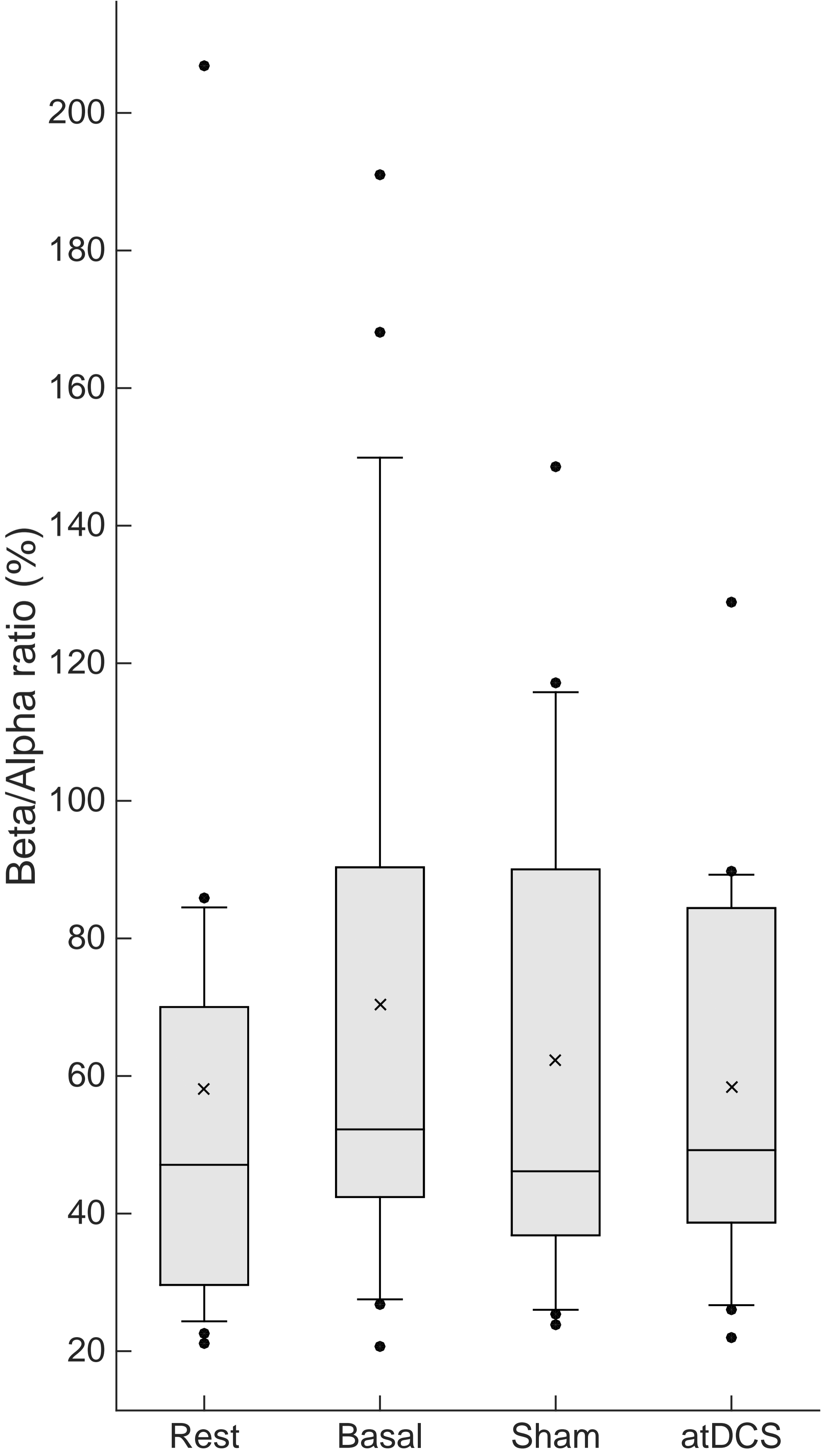

Supplement: Supplementary file 1 [file Data_Sheet_1.zip › Complementary_results/Band_ratios_Complete_EEG/Beta_Alpha/Beta-Alpha_complete-EEG_FC6.pdf]

**Beta/Alpha ratio on complete EEG signal for electrode: O1**

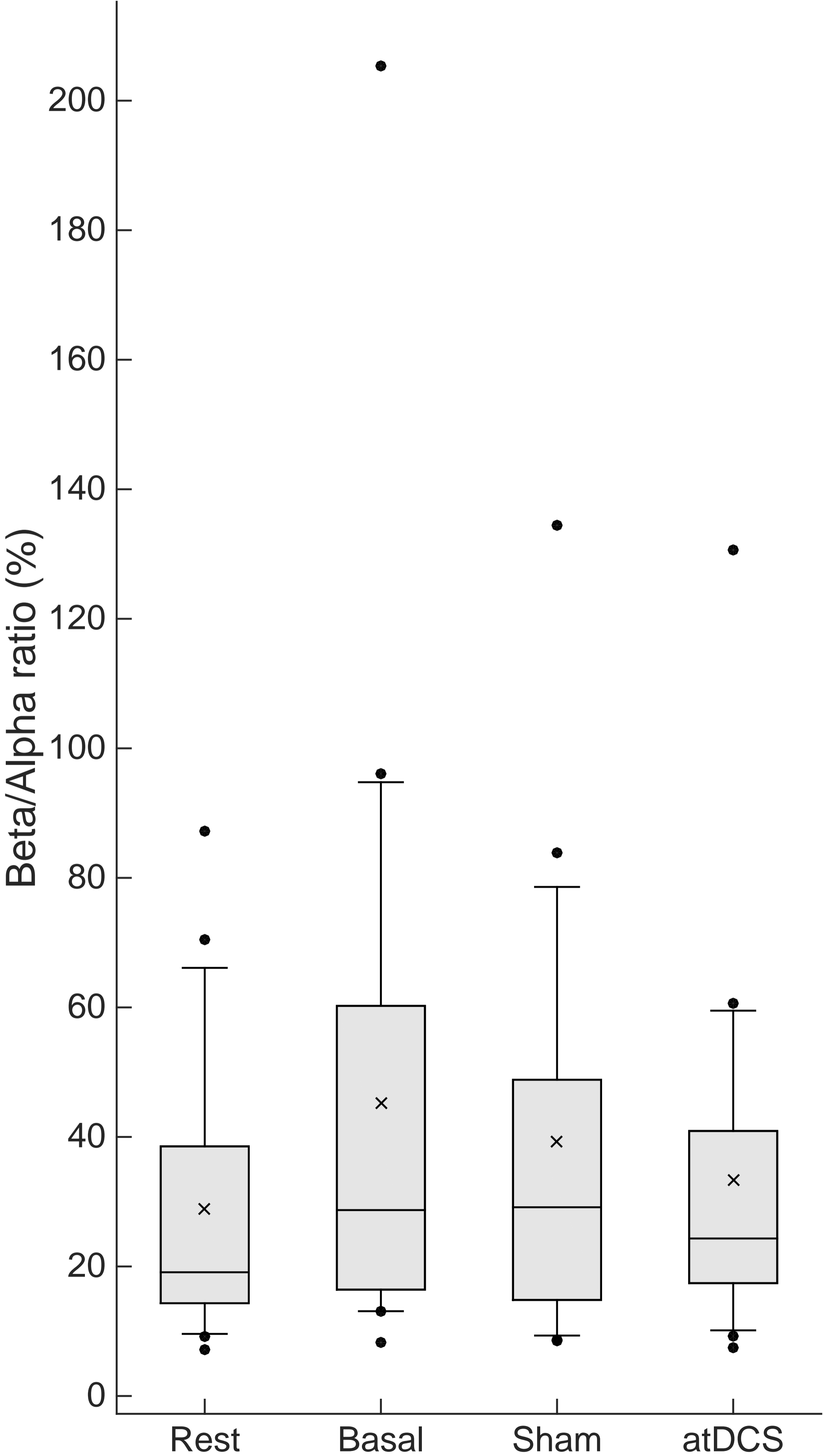

Supplement: Supplementary file 1 [file Data_Sheet_1.zip › Complementary_results/Band_ratios_Complete_EEG/Beta_Alpha/Beta-Alpha_complete-EEG_O1.pdf]

**Beta/Alpha ratio on complete EEG signal for electrode: O2**

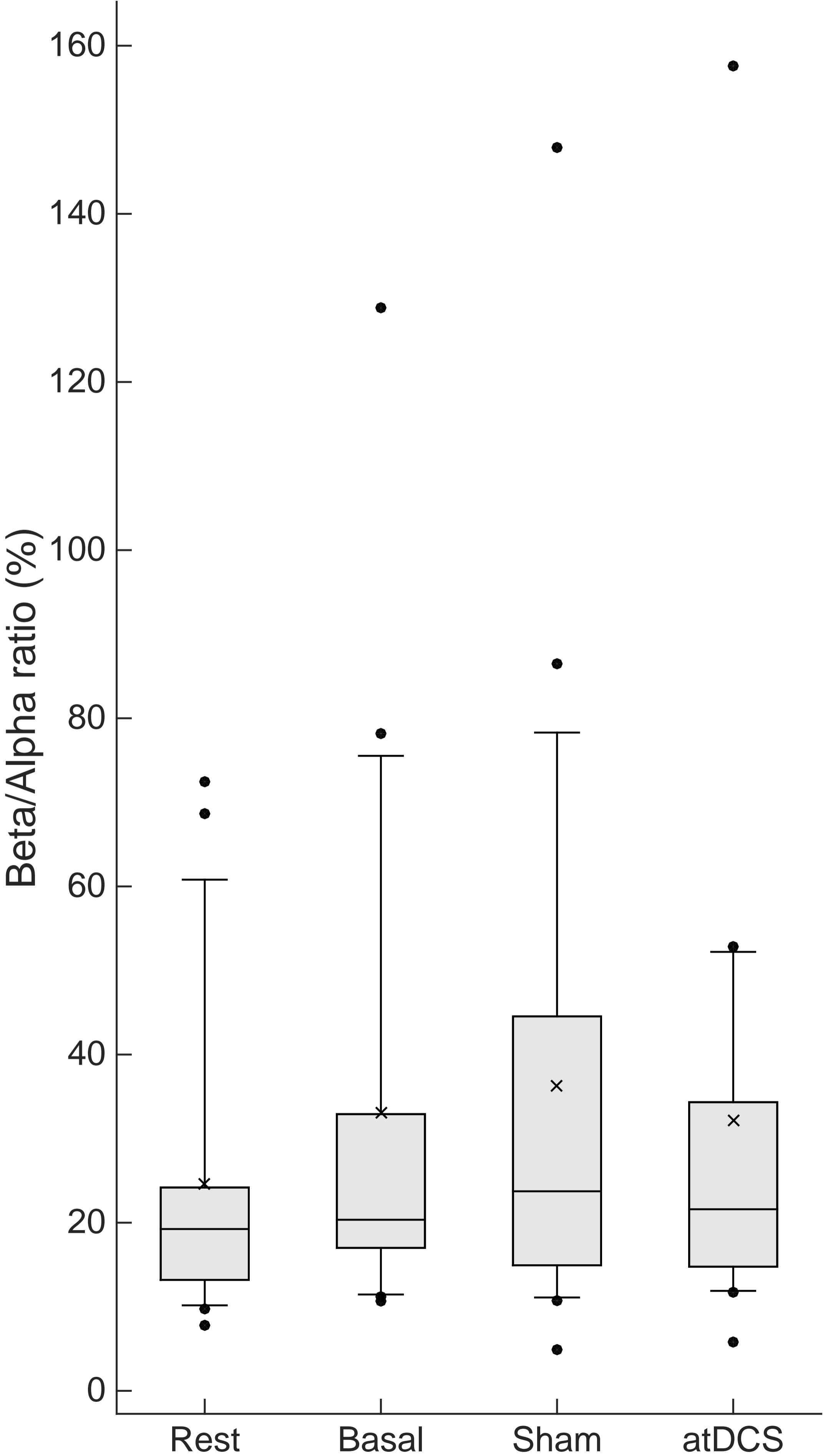

Supplement: Supplementary file 1 [file Data_Sheet_1.zip › Complementary_results/Band_ratios_Complete_EEG/Beta_Alpha/Beta-Alpha_complete-EEG_O2.pdf]

**Beta/Alpha ratio on complete EEG signal for electrode: P7**

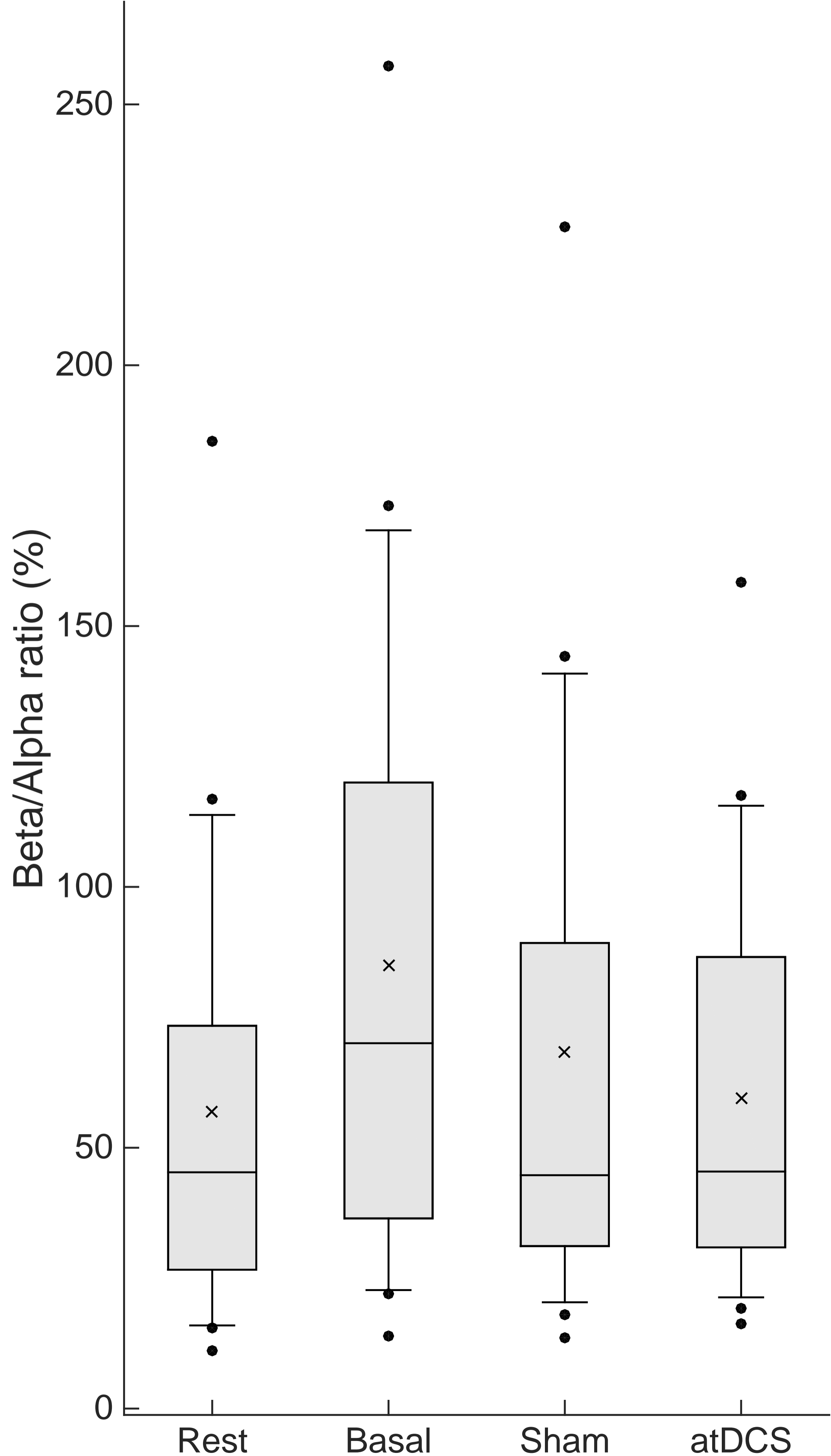

Supplement: Supplementary file 1 [file Data_Sheet_1.zip › Complementary_results/Band_ratios_Complete_EEG/Beta_Alpha/Beta-Alpha_complete-EEG_P7.pdf]

**Beta/Alpha ratio on complete EEG signal for electrode: P8**

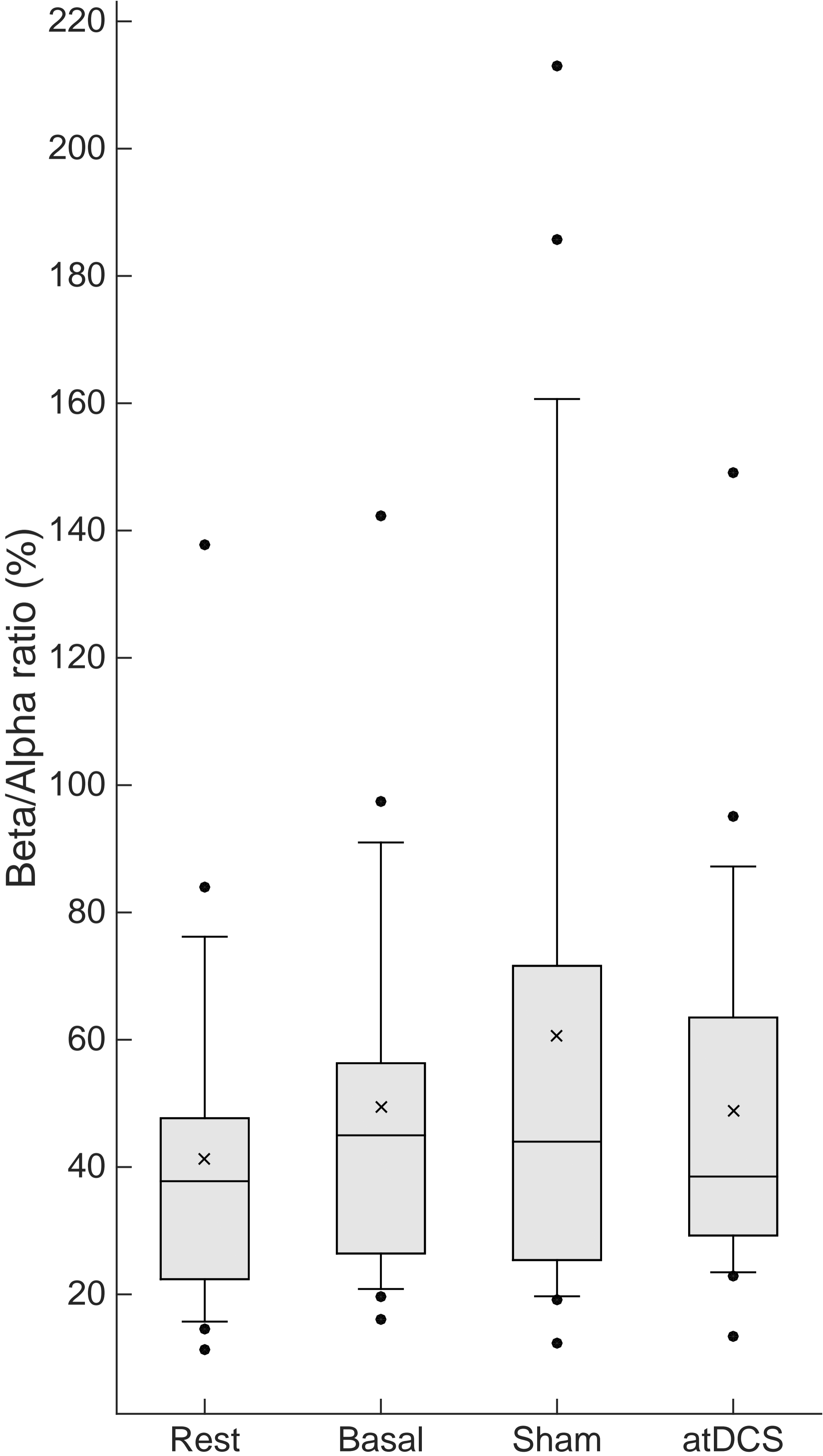

Supplement: Supplementary file 1 [file Data_Sheet_1.zip › Complementary_results/Band_ratios_Complete_EEG/Beta_Alpha/Beta-Alpha_complete-EEG_P8.pdf]

**Beta/Alpha ratio on complete EEG signal for electrode: T7**

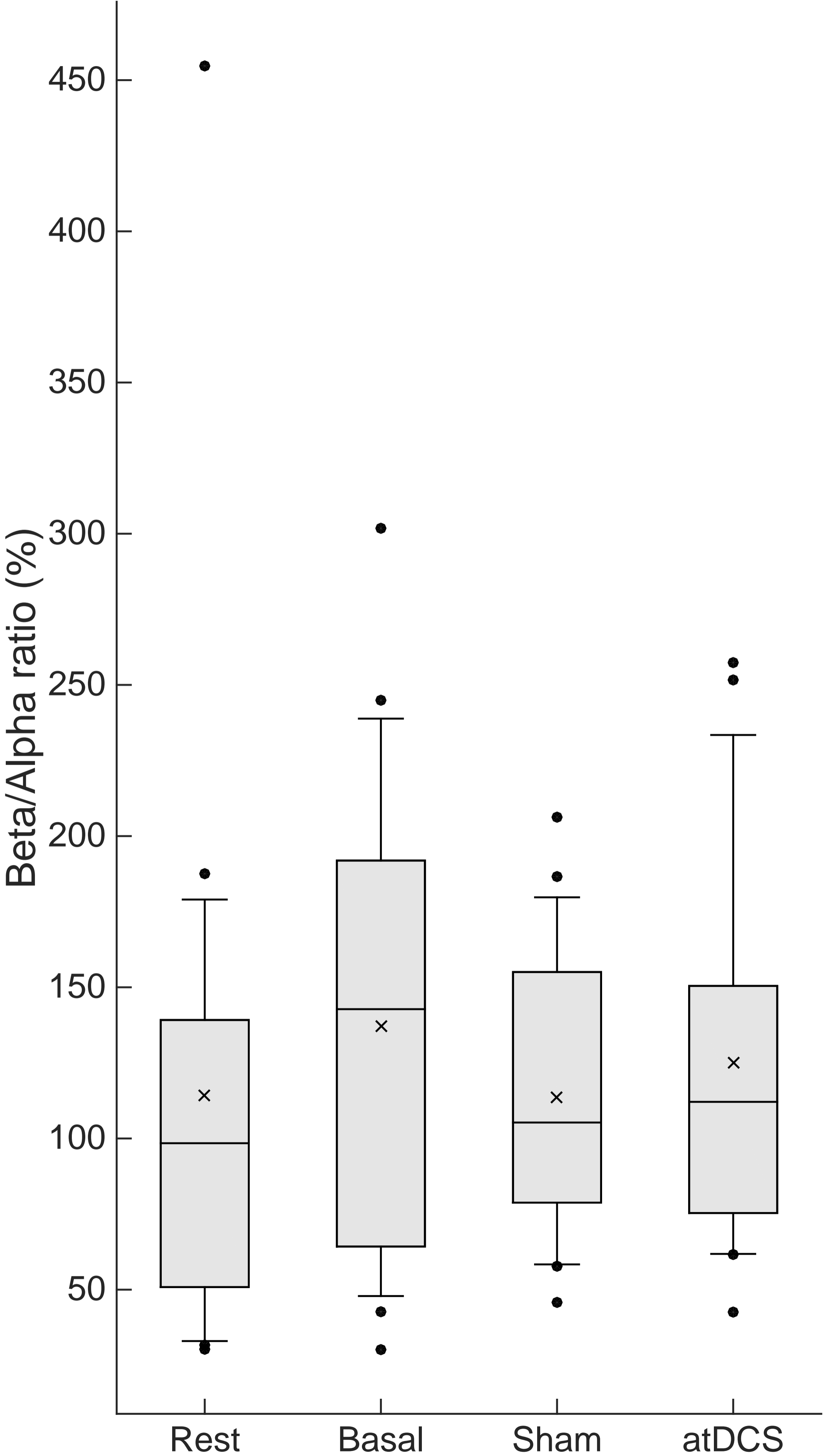

Supplement: Supplementary file 1 [file Data_Sheet_1.zip › Complementary_results/Band_ratios_Complete_EEG/Beta_Alpha/Beta-Alpha_complete-EEG_T7.pdf]

**Beta/Alpha ratio on complete EEG signal for electrode: T8**

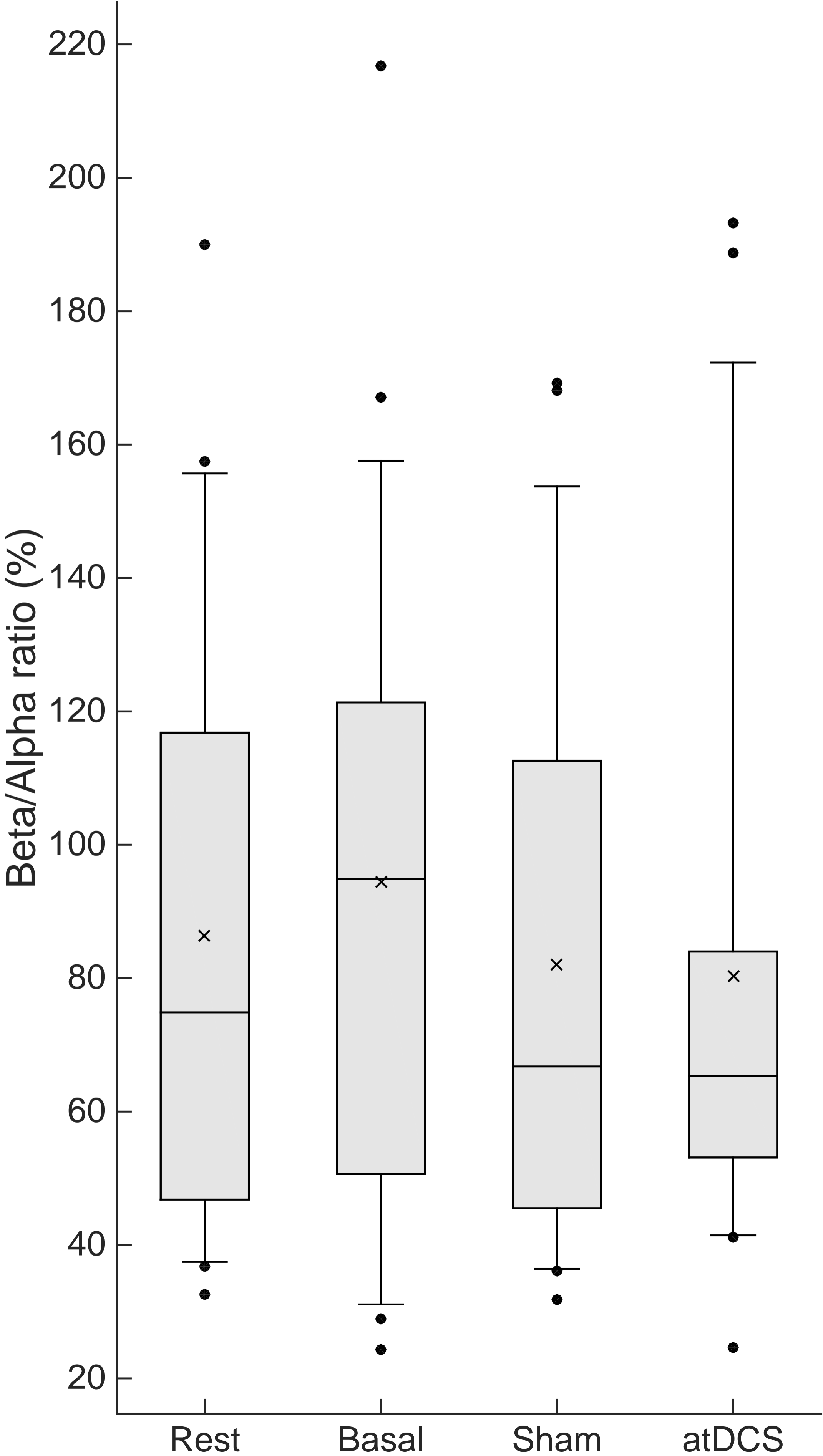

Supplement: Supplementary file 1 [file Data_Sheet_1.zip › Complementary_results/Band_ratios_Complete_EEG/Beta_Alpha/Beta-Alpha_complete-EEG_T8.pdf]

**Beta/Delta ratio on complete EEG signal for electrode: AF3**

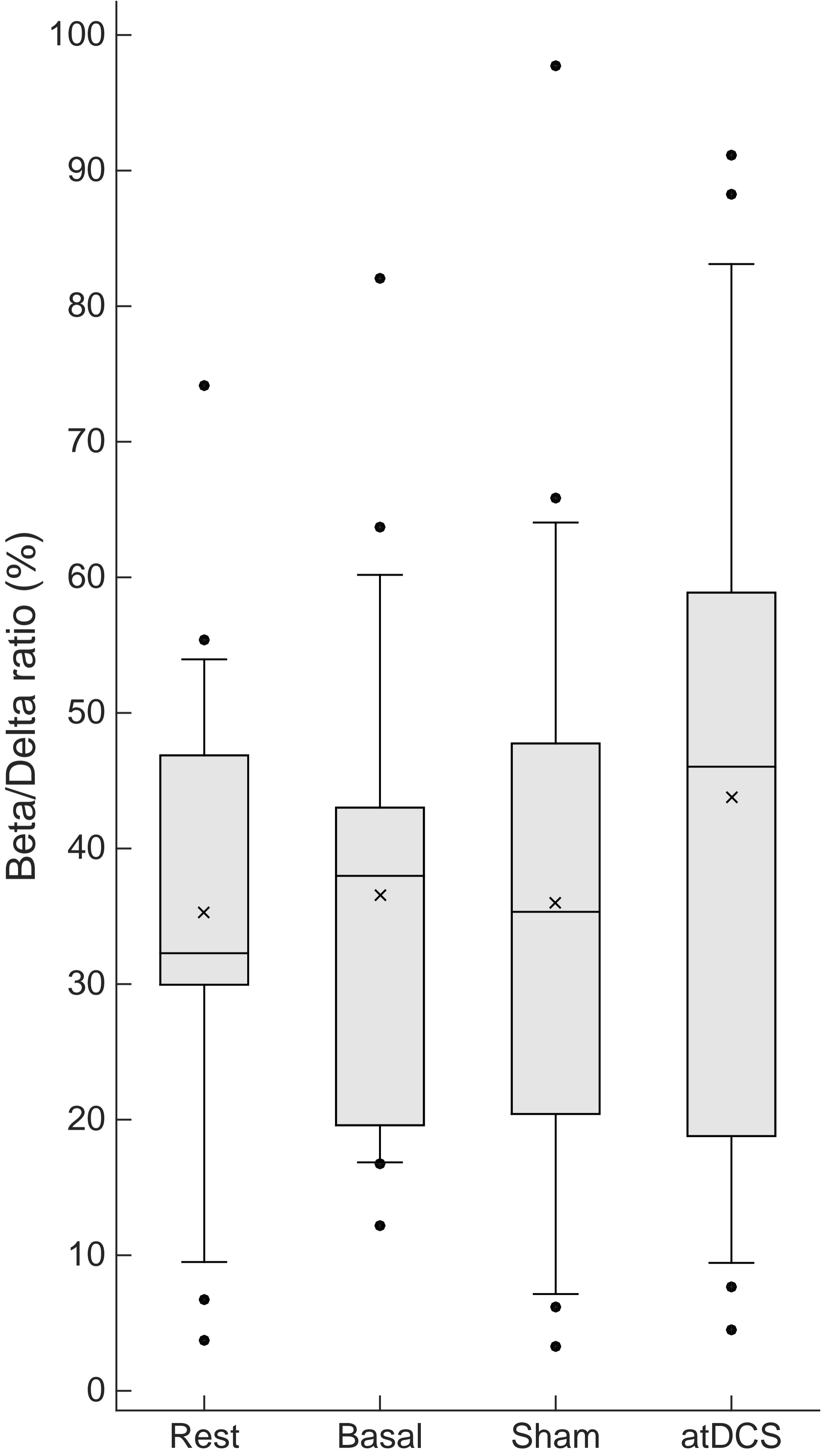

Supplement: Supplementary file 1 [file Data_Sheet_1.zip › Complementary_results/Band_ratios_Complete_EEG/Beta_Delta/Beta-Delta_complete-EEG_AF3.pdf]

**Beta/Delta ratio on complete EEG signal for electrode: AF4**

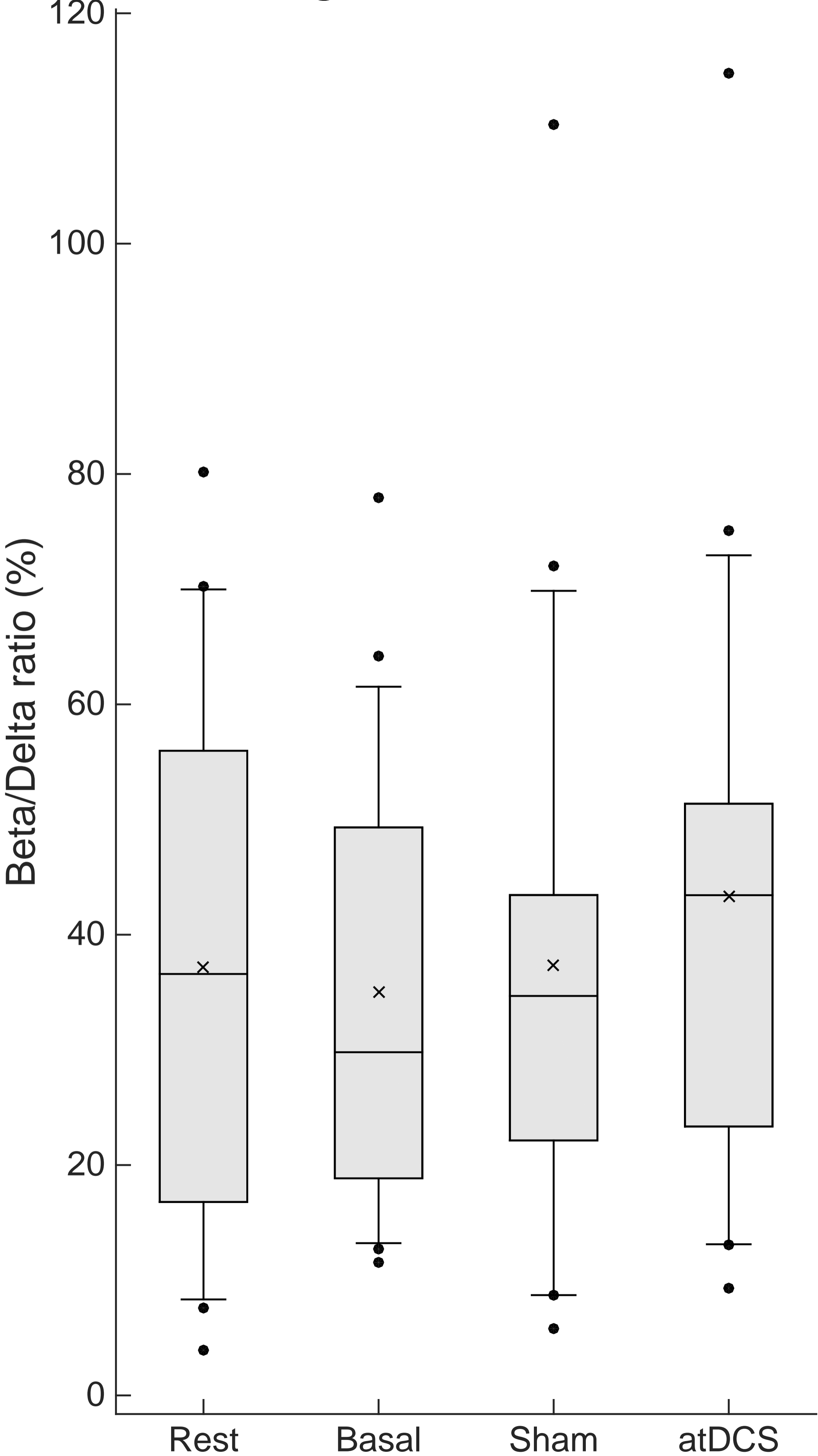

Supplement: Supplementary file 1 [file Data_Sheet_1.zip › Complementary_results/Band_ratios_Complete_EEG/Beta_Delta/Beta-Delta_complete-EEG_AF4.pdf]

**Beta/Delta ratio on complete EEG signal for electrode: Avg AF3-F3-F7**

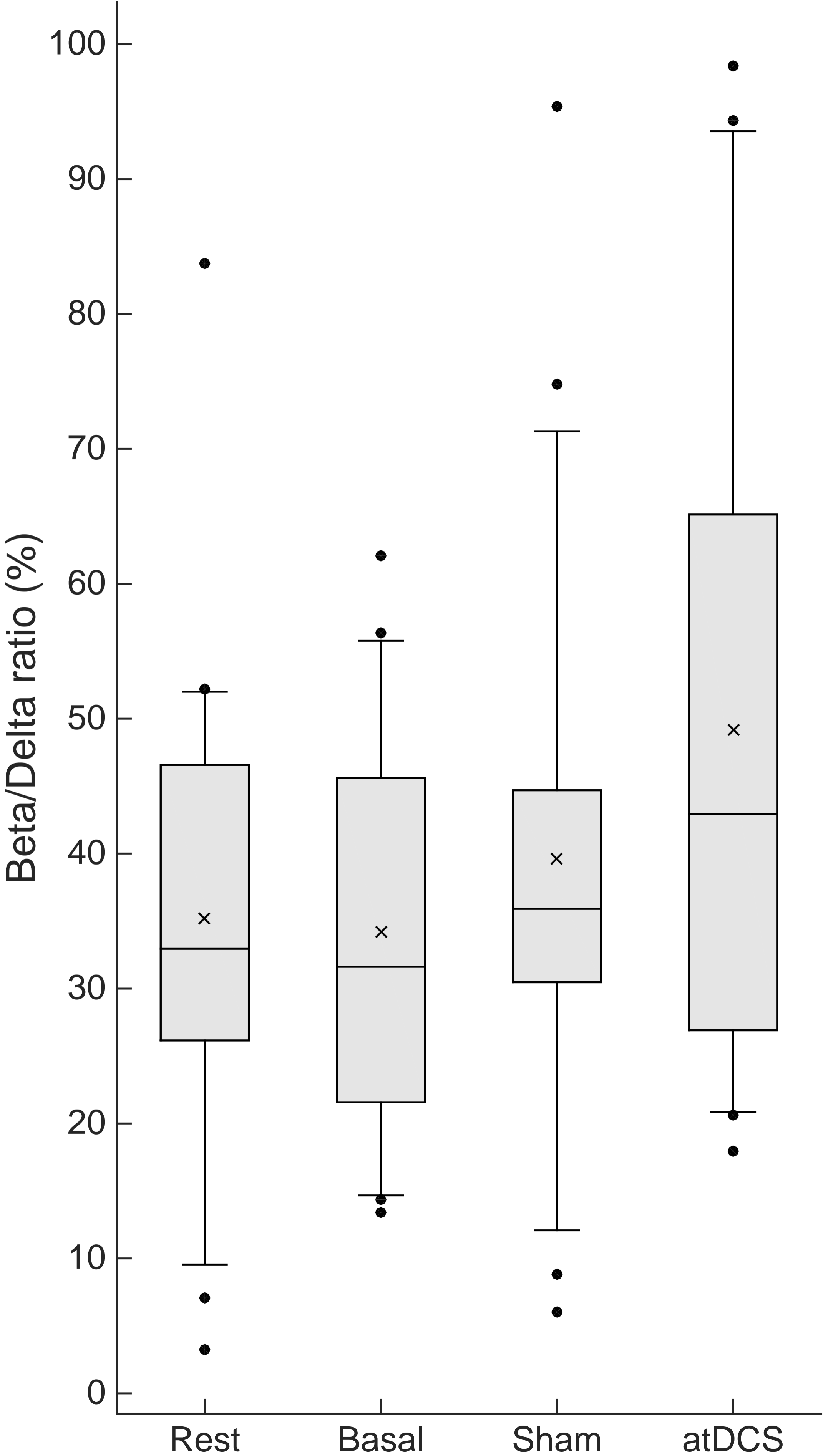

Supplement: Supplementary file 1 [file Data_Sheet_1.zip › Complementary_results/Band_ratios_Complete_EEG/Beta_Delta/Beta-Delta_complete-EEG_Avg AF3-F3-F7.pdf]

**Beta/Delta ratio on complete EEG signal for electrode: Avg AF4-F4-F8**

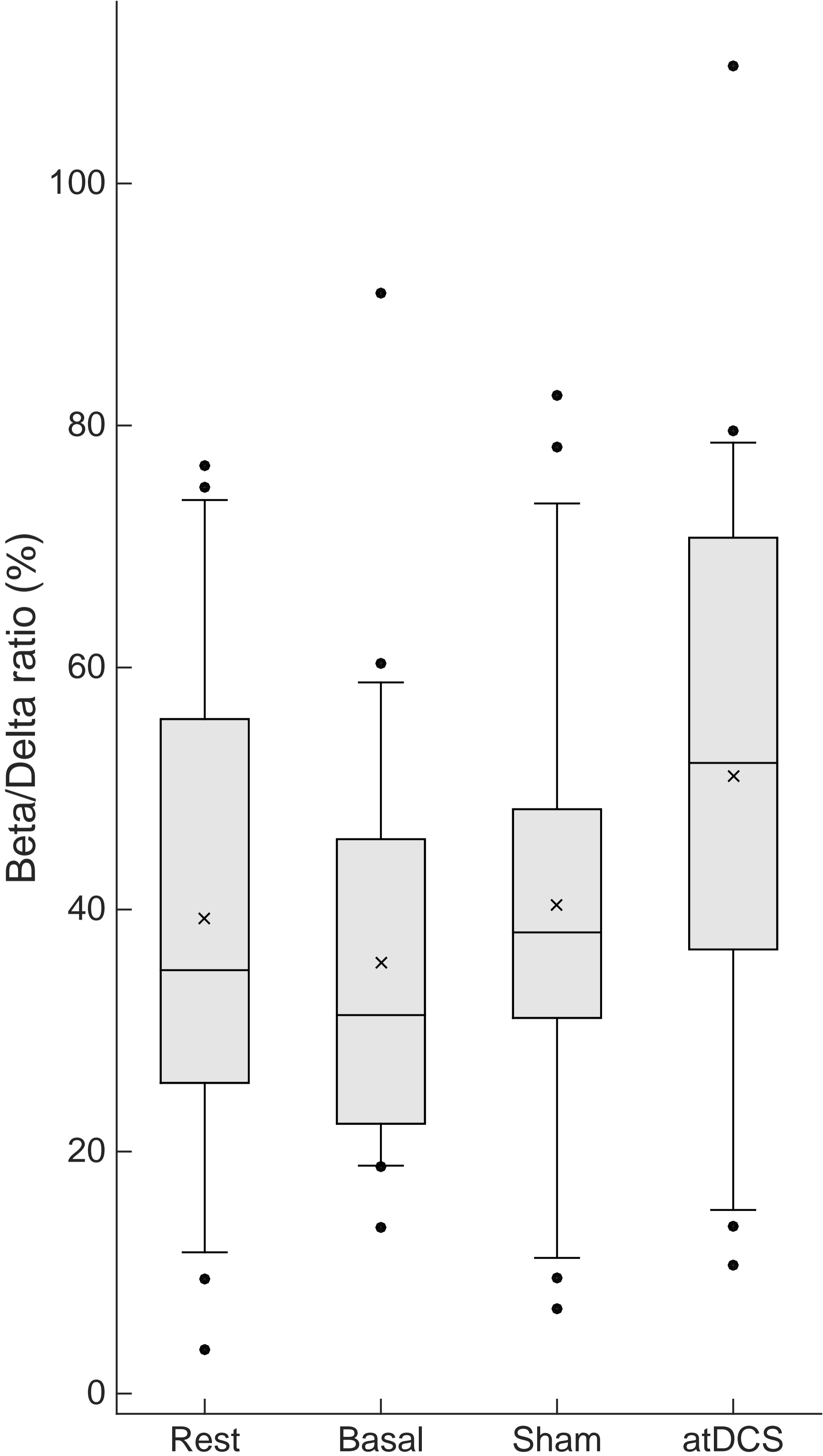

Supplement: Supplementary file 1 [file Data_Sheet_1.zip › Complementary_results/Band_ratios_Complete_EEG/Beta_Delta/Beta-Delta_complete-EEG_Avg AF4-F4-F8.pdf]

**Beta/Delta ratio on complete EEG signal for electrode: Avg F3-F7-FC5**

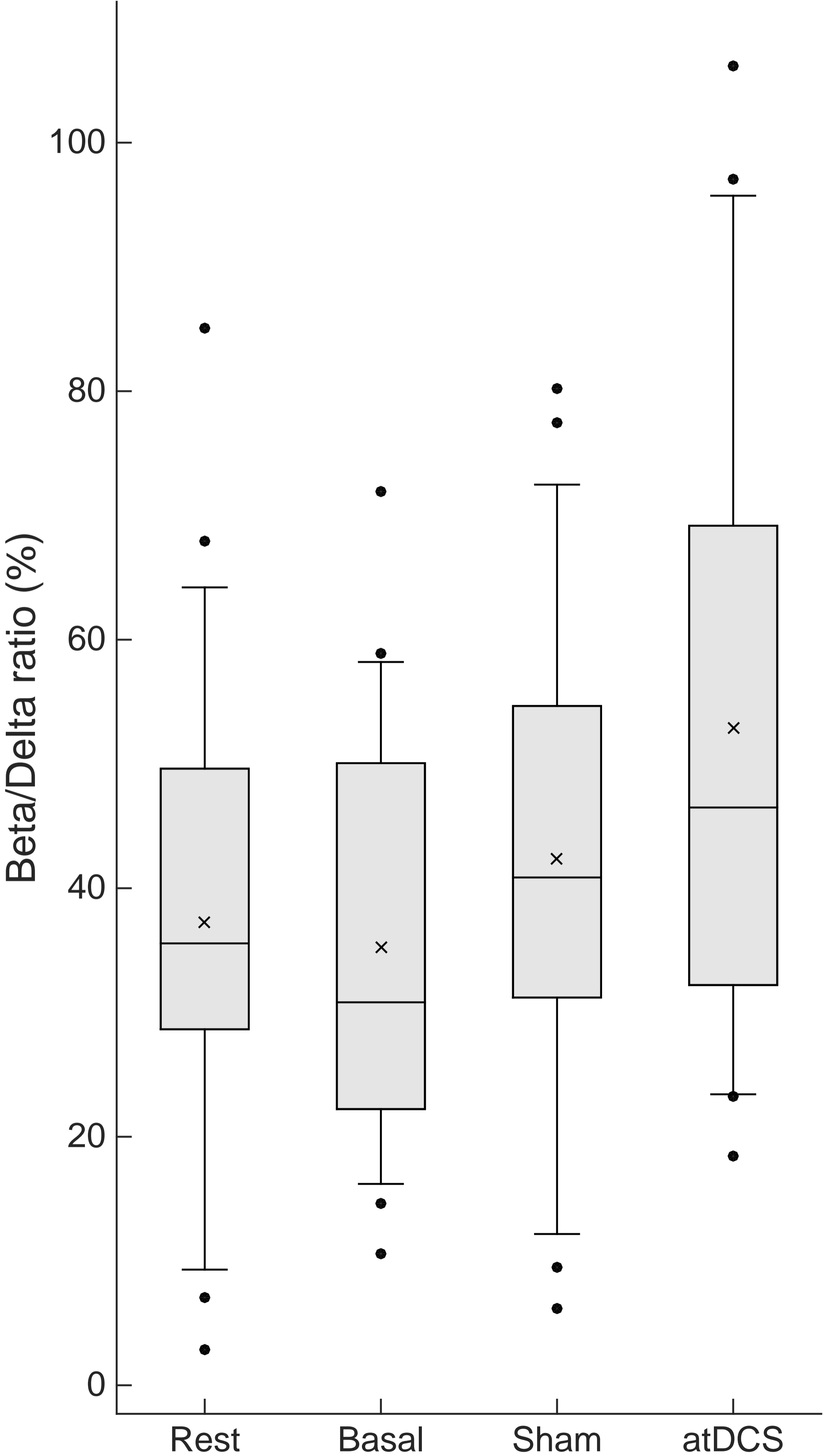

Supplement: Supplementary file 1 [file Data_Sheet_1.zip › Complementary_results/Band_ratios_Complete_EEG/Beta_Delta/Beta-Delta_complete-EEG_Avg F3-F7-FC5.pdf]

**Beta/Delta ratio on complete EEG signal for electrode: Avg F4-F8-FC6**

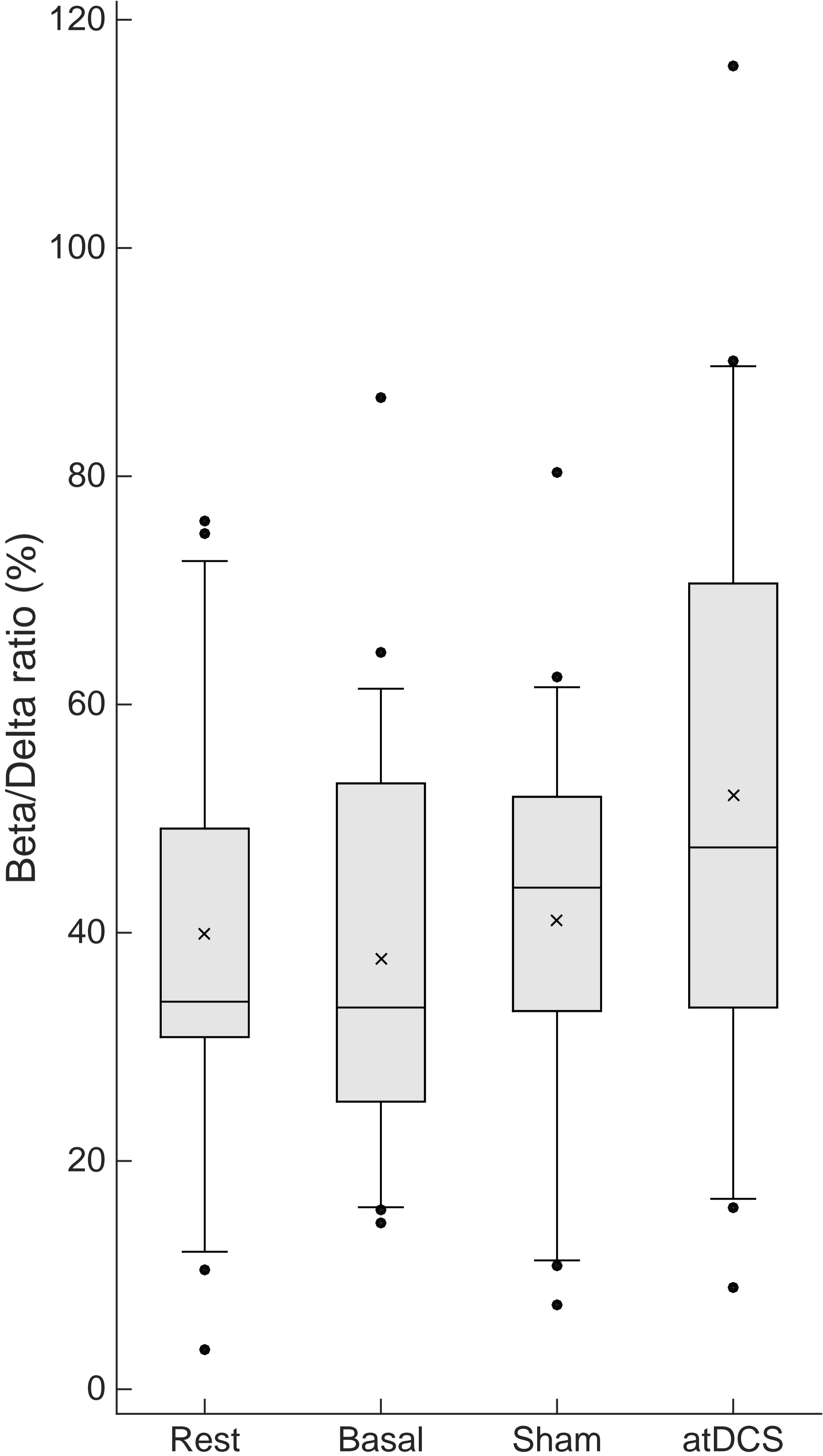

Supplement: Supplementary file 1 [file Data_Sheet_1.zip › Complementary_results/Band_ratios_Complete_EEG/Beta_Delta/Beta-Delta_complete-EEG_Avg F4-F8-FC6.pdf]

**Beta/Delta ratio on complete EEG signal for electrode: F3**

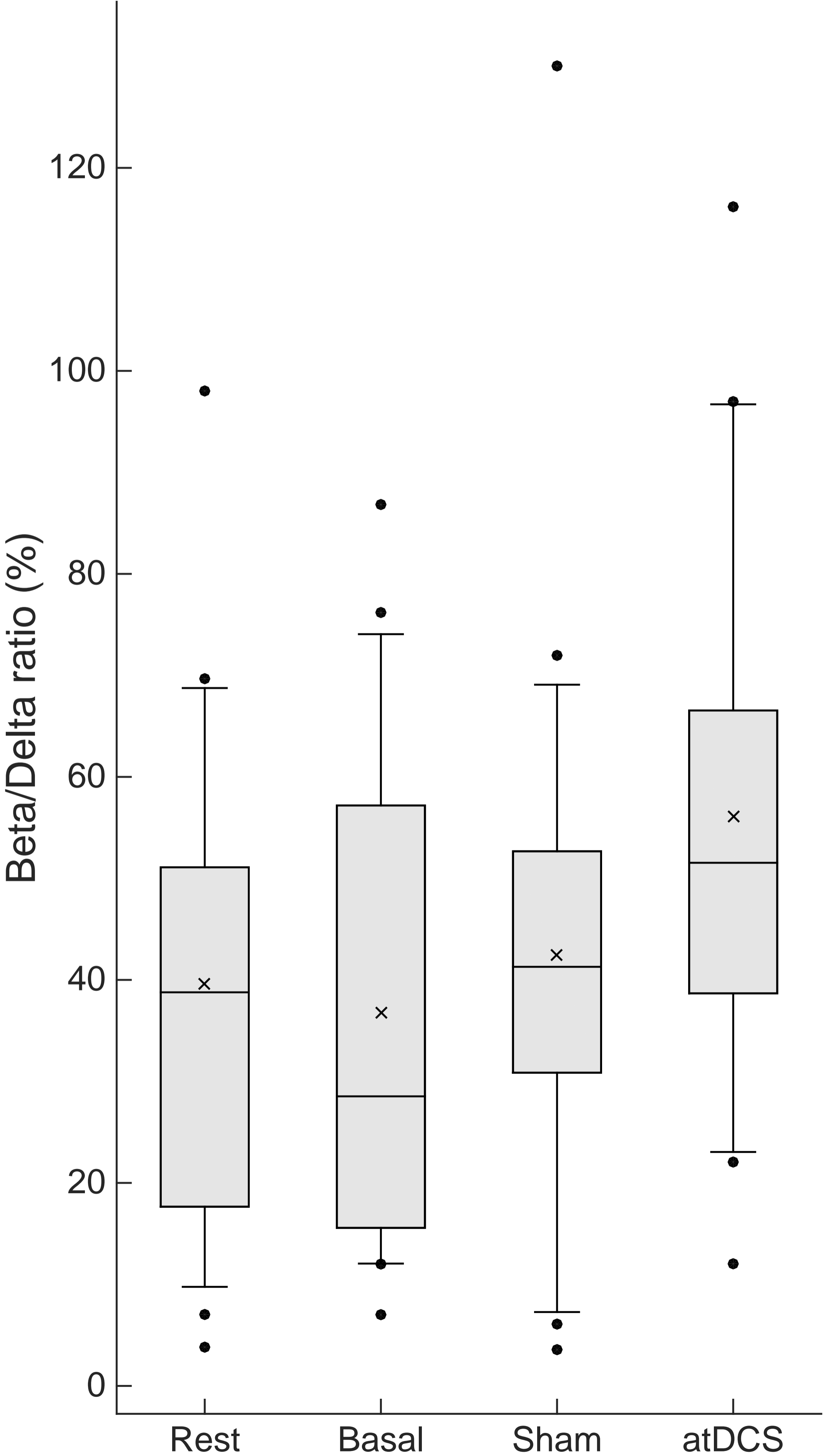

Supplement: Supplementary file 1 [file Data_Sheet_1.zip › Complementary_results/Band_ratios_Complete_EEG/Beta_Delta/Beta-Delta_complete-EEG_F3.pdf]

**Beta/Delta ratio on complete EEG signal for electrode: F4**

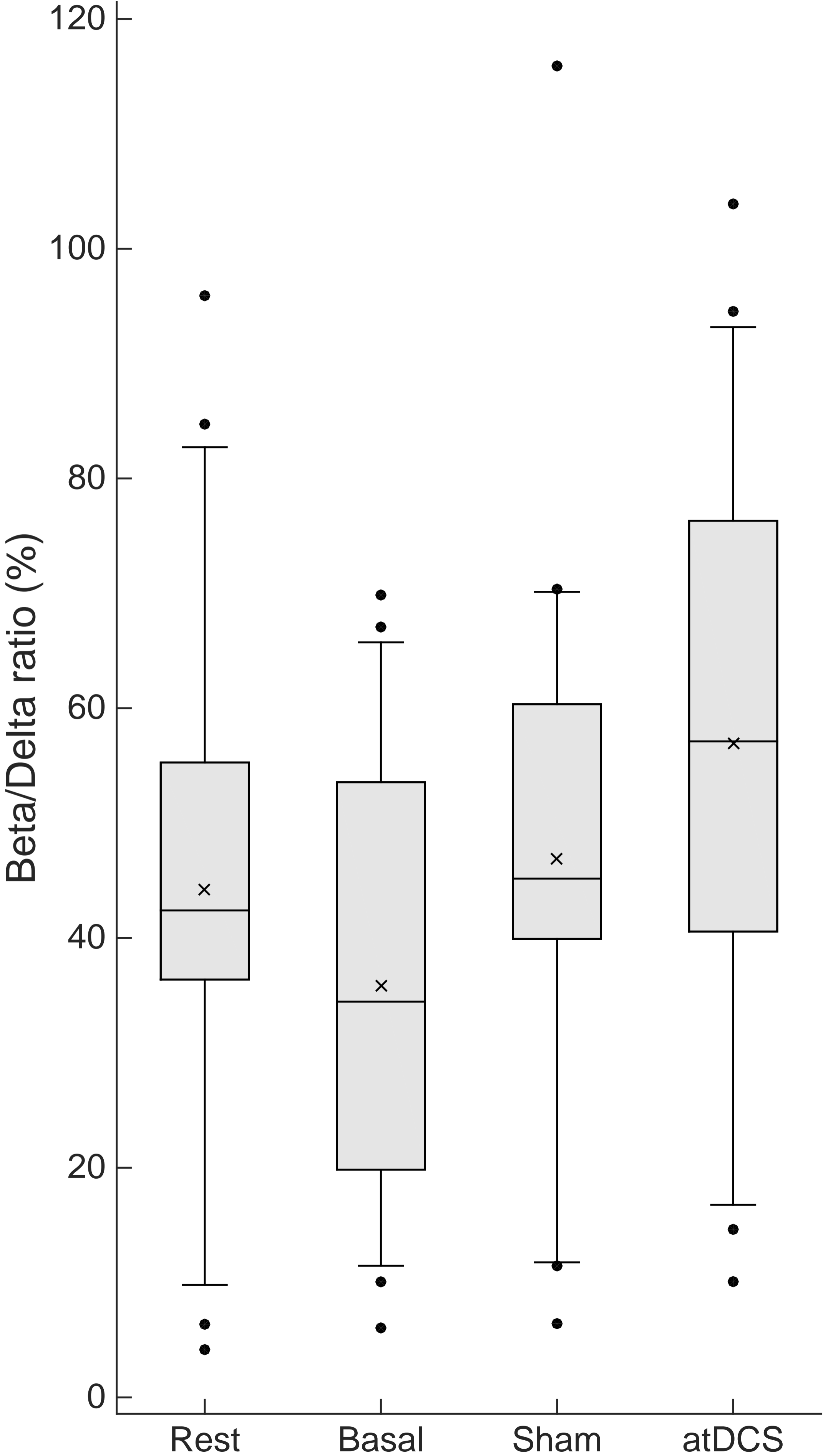

Supplement: Supplementary file 1 [file Data_Sheet_1.zip › Complementary_results/Band_ratios_Complete_EEG/Beta_Delta/Beta-Delta_complete-EEG_F4.pdf]

**Beta/Delta ratio on complete EEG signal for electrode: F7**

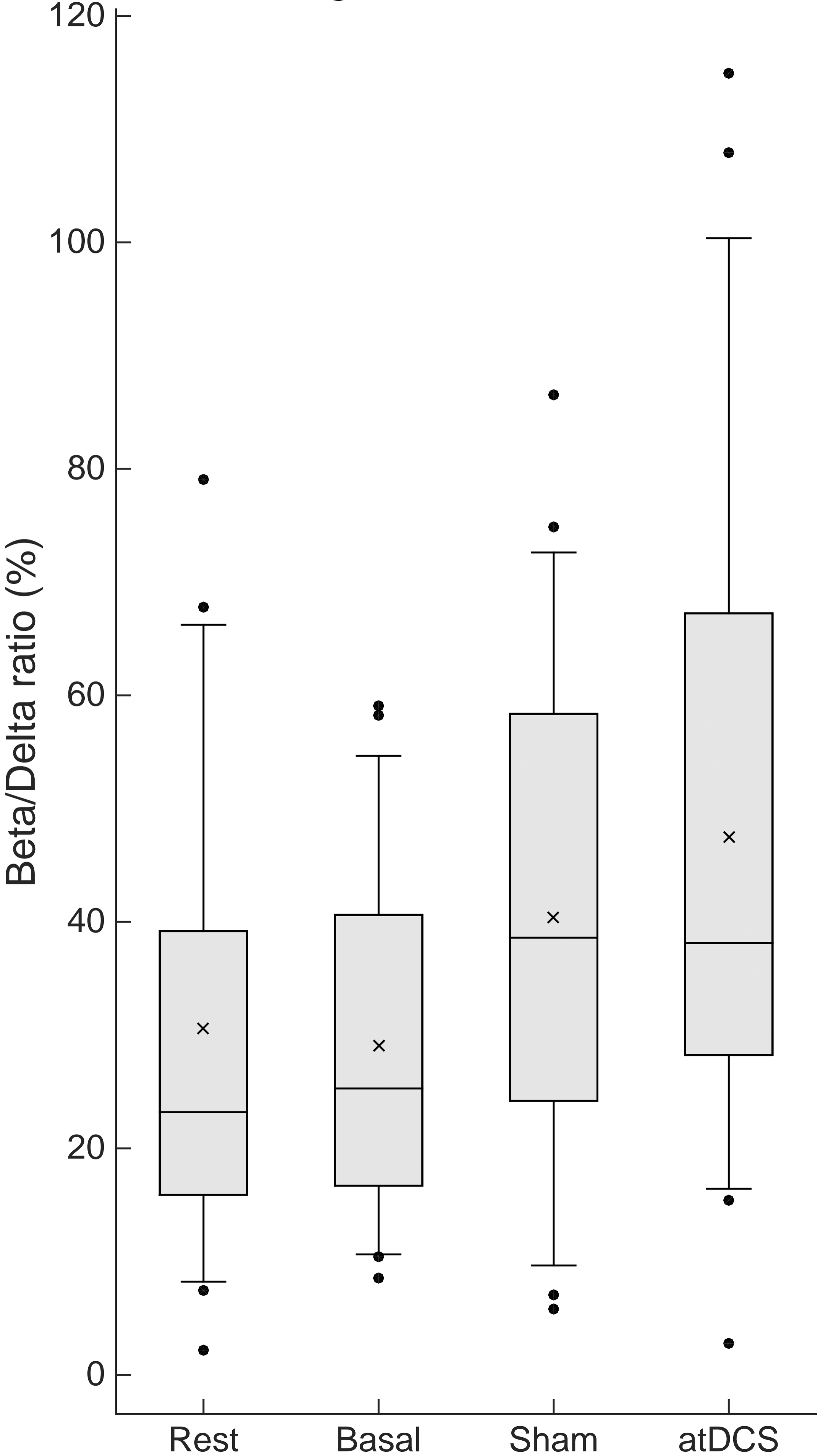

Supplement: Supplementary file 1 [file Data_Sheet_1.zip › Complementary_results/Band_ratios_Complete_EEG/Beta_Delta/Beta-Delta_complete-EEG_F7.pdf]

**Beta/Delta ratio on complete EEG signal for electrode: F8**

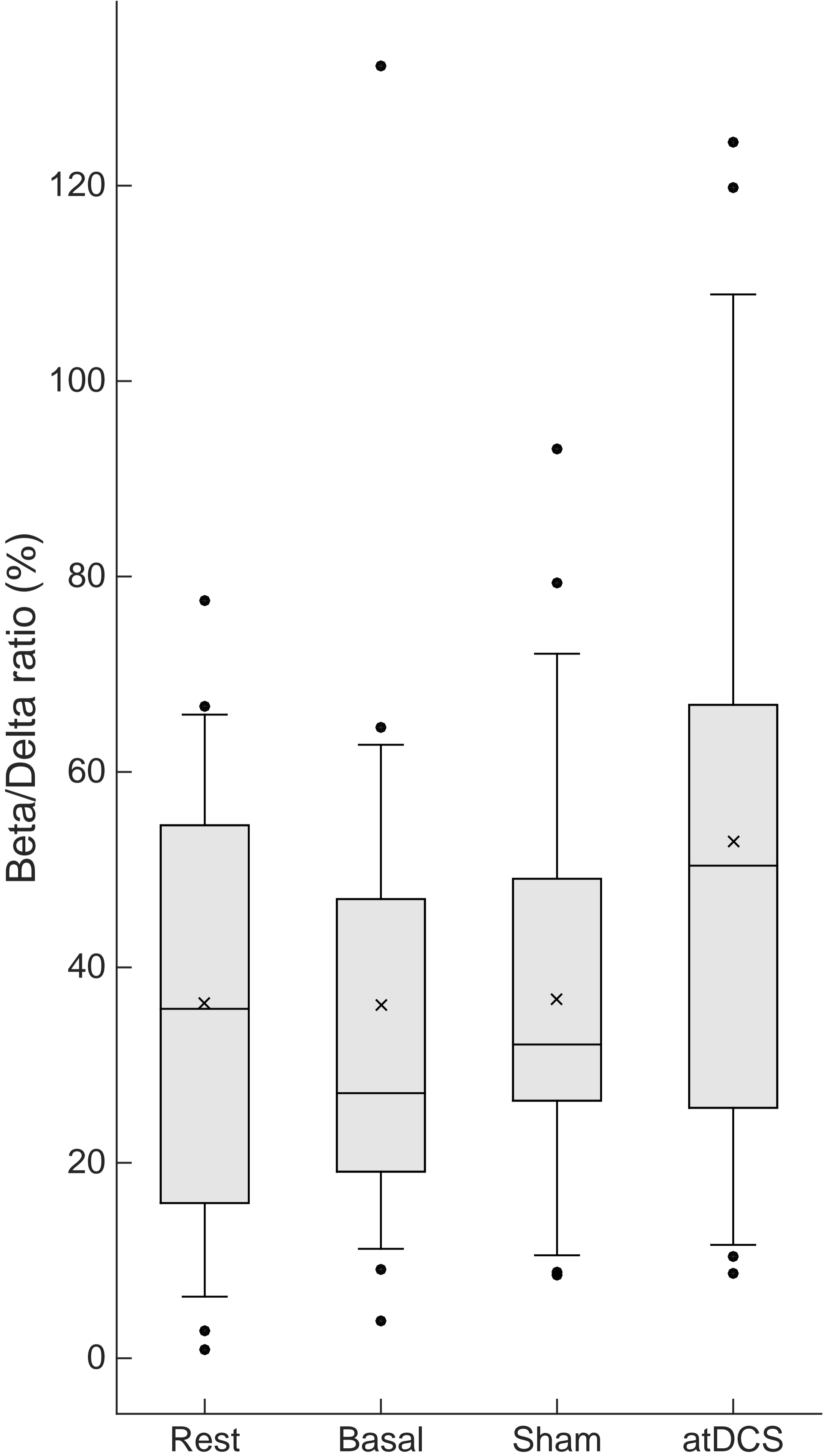

Supplement: Supplementary file 1 [file Data_Sheet_1.zip › Complementary_results/Band_ratios_Complete_EEG/Beta_Delta/Beta-Delta_complete-EEG_F8.pdf]

**Beta/Delta ratio on complete EEG signal for electrode: FC5**

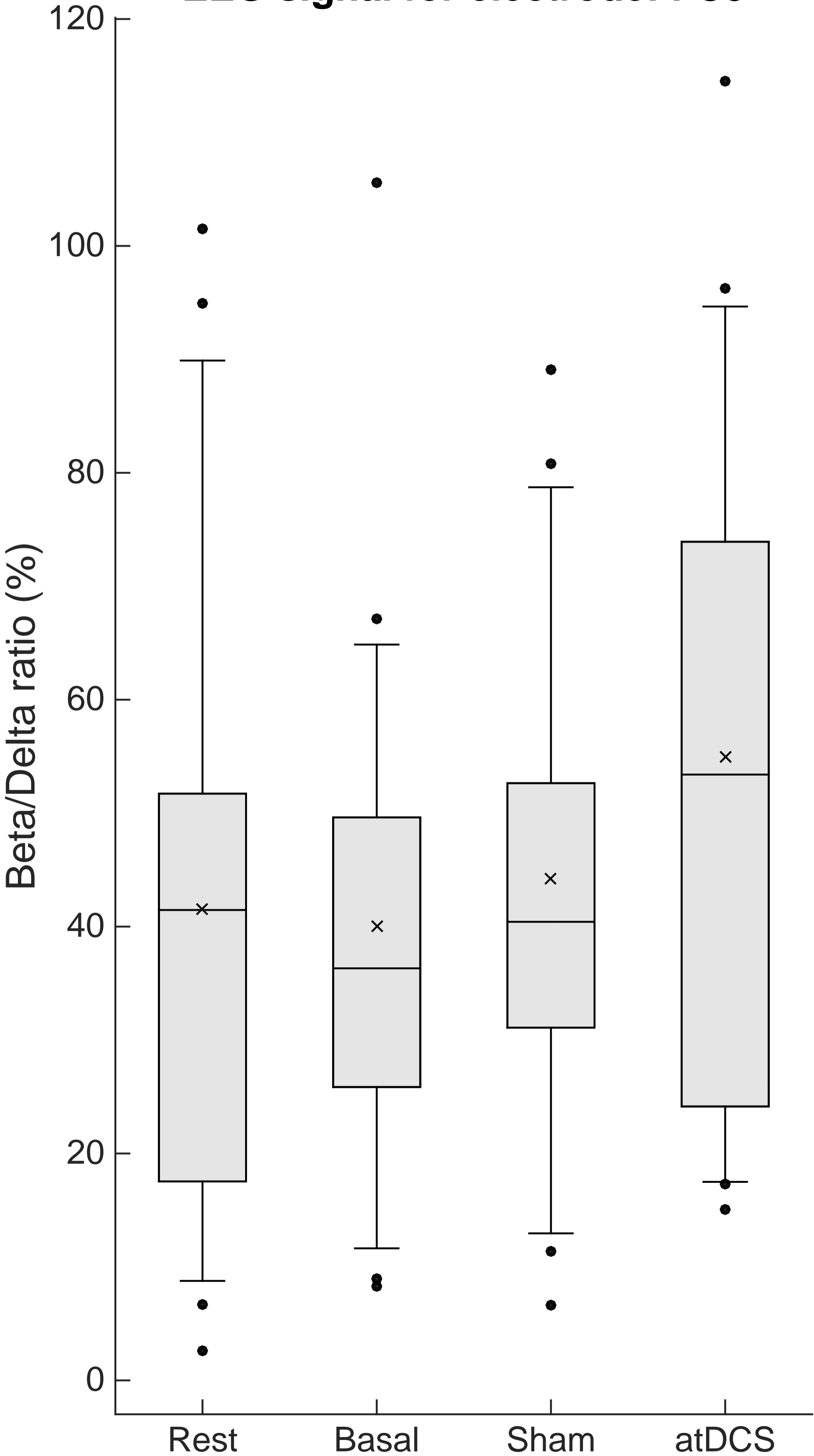

Supplement: Supplementary file 1 [file Data_Sheet_1.zip › Complementary_results/Band_ratios_Complete_EEG/Beta_Delta/Beta-Delta_complete-EEG_FC5.pdf]

**Beta/Delta ratio on complete EEG signal for electrode: FC6**

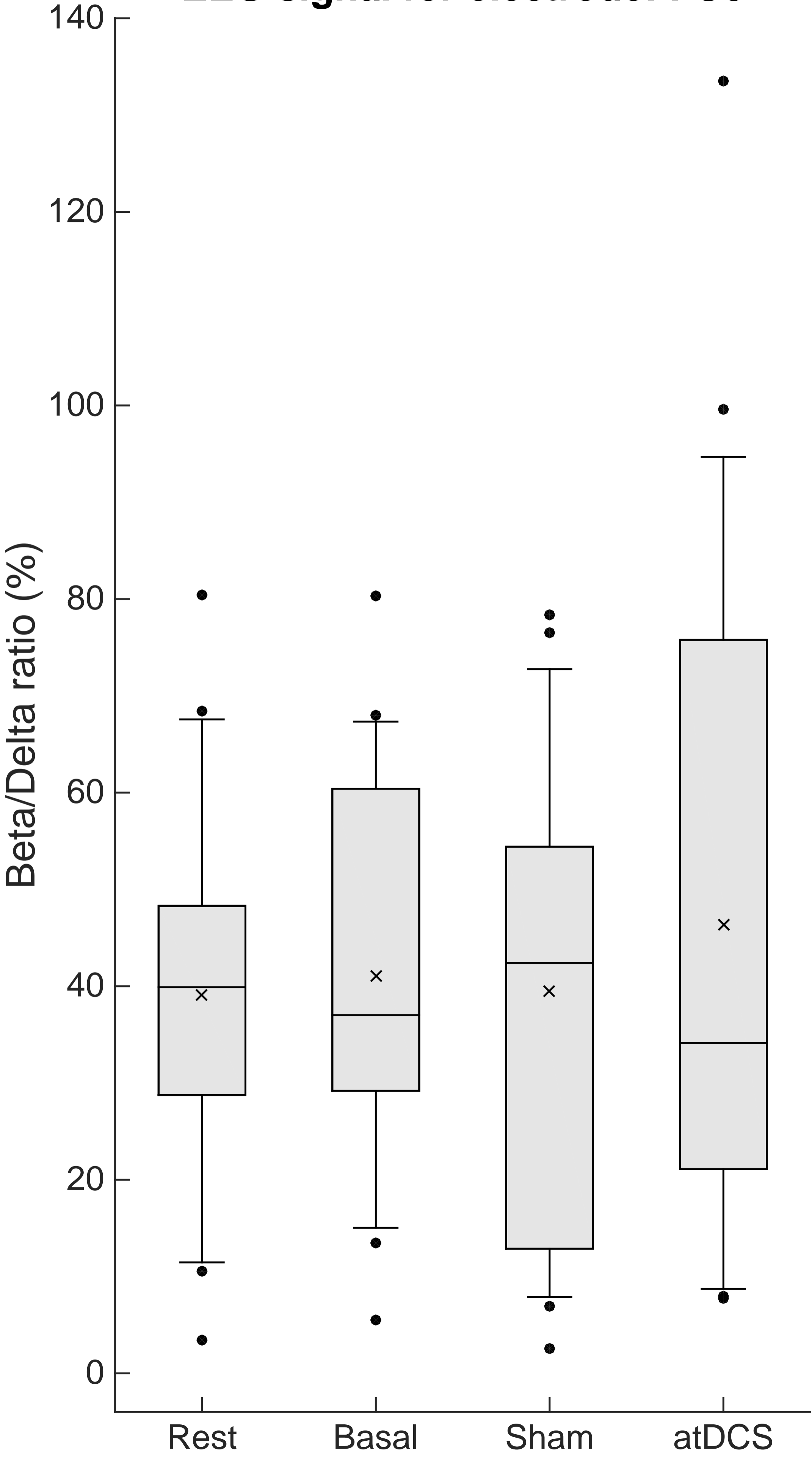

Supplement: Supplementary file 1 [file Data_Sheet_1.zip › Complementary_results/Band_ratios_Complete_EEG/Beta_Delta/Beta-Delta_complete-EEG_FC6.pdf]

**Beta/Delta ratio on complete EEG signal for electrode: O1**

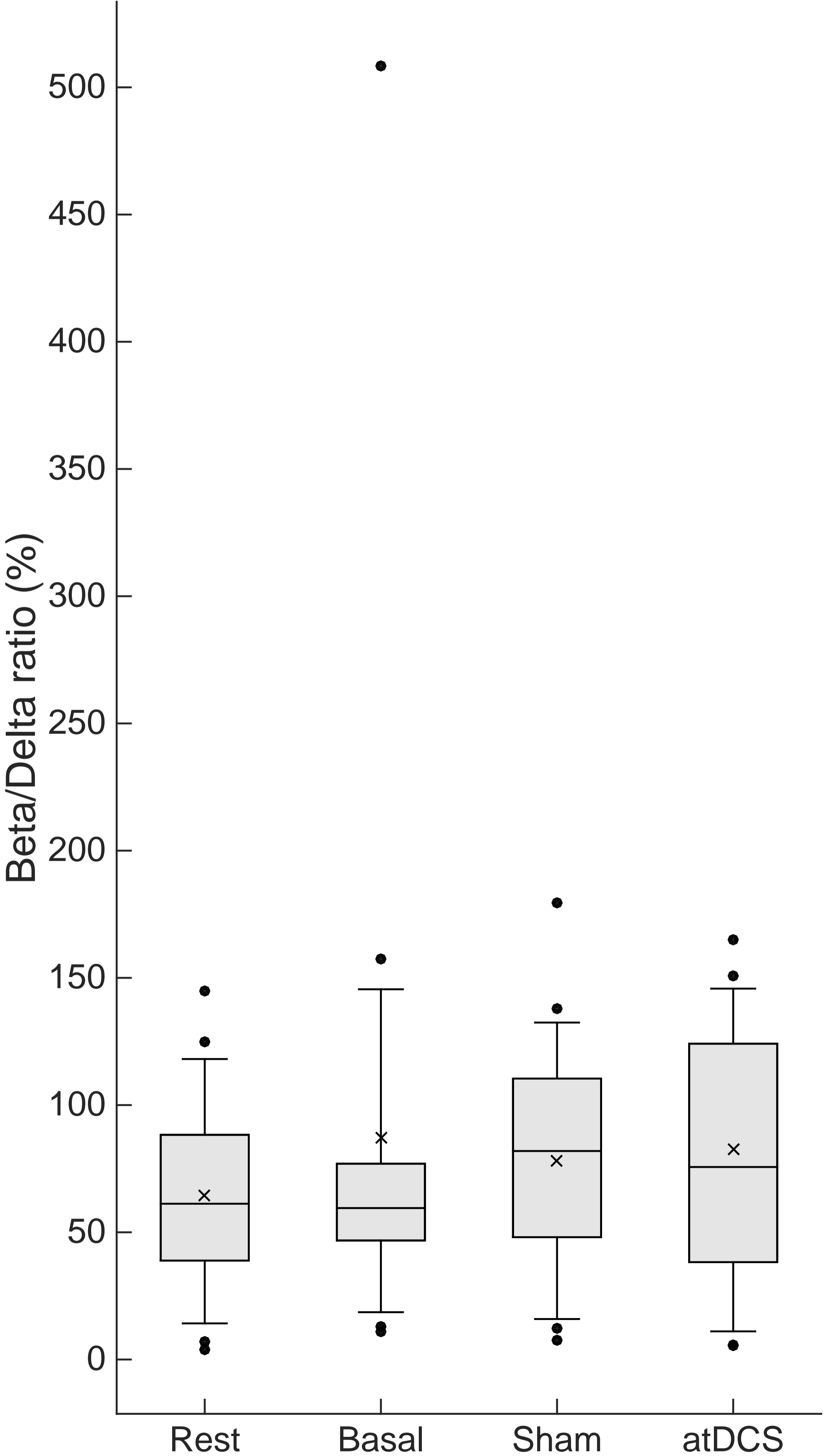

Supplement: Supplementary file 1 [file Data_Sheet_1.zip › Complementary_results/Band_ratios_Complete_EEG/Beta_Delta/Beta-Delta_complete-EEG_O1.pdf]

**Beta/Delta ratio on complete EEG signal for electrode: O2**

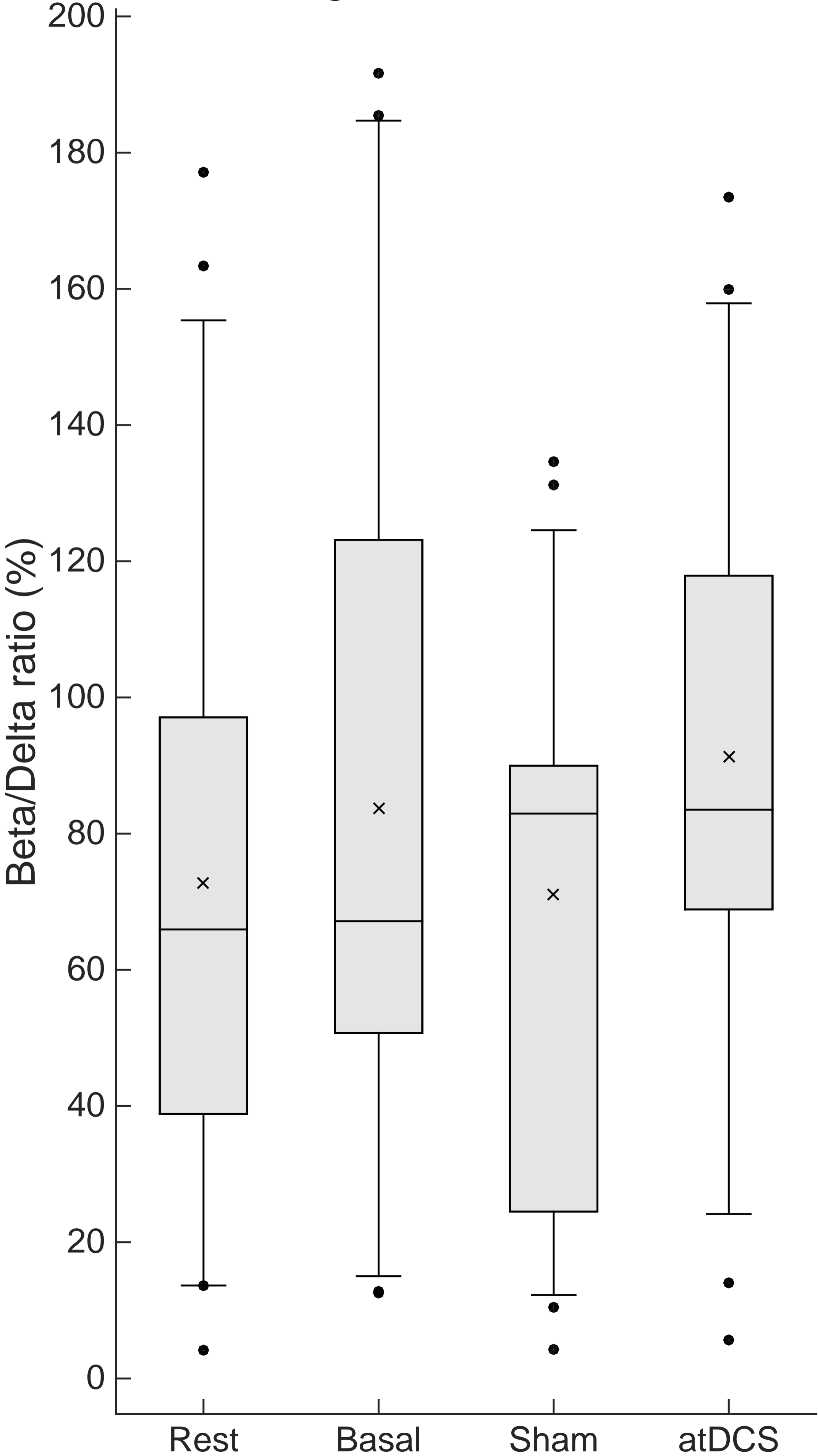

Supplement: Supplementary file 1 [file Data_Sheet_1.zip › Complementary_results/Band_ratios_Complete_EEG/Beta_Delta/Beta-Delta_complete-EEG_O2.pdf]

**Beta/Delta ratio on complete EEG signal for electrode: P7**

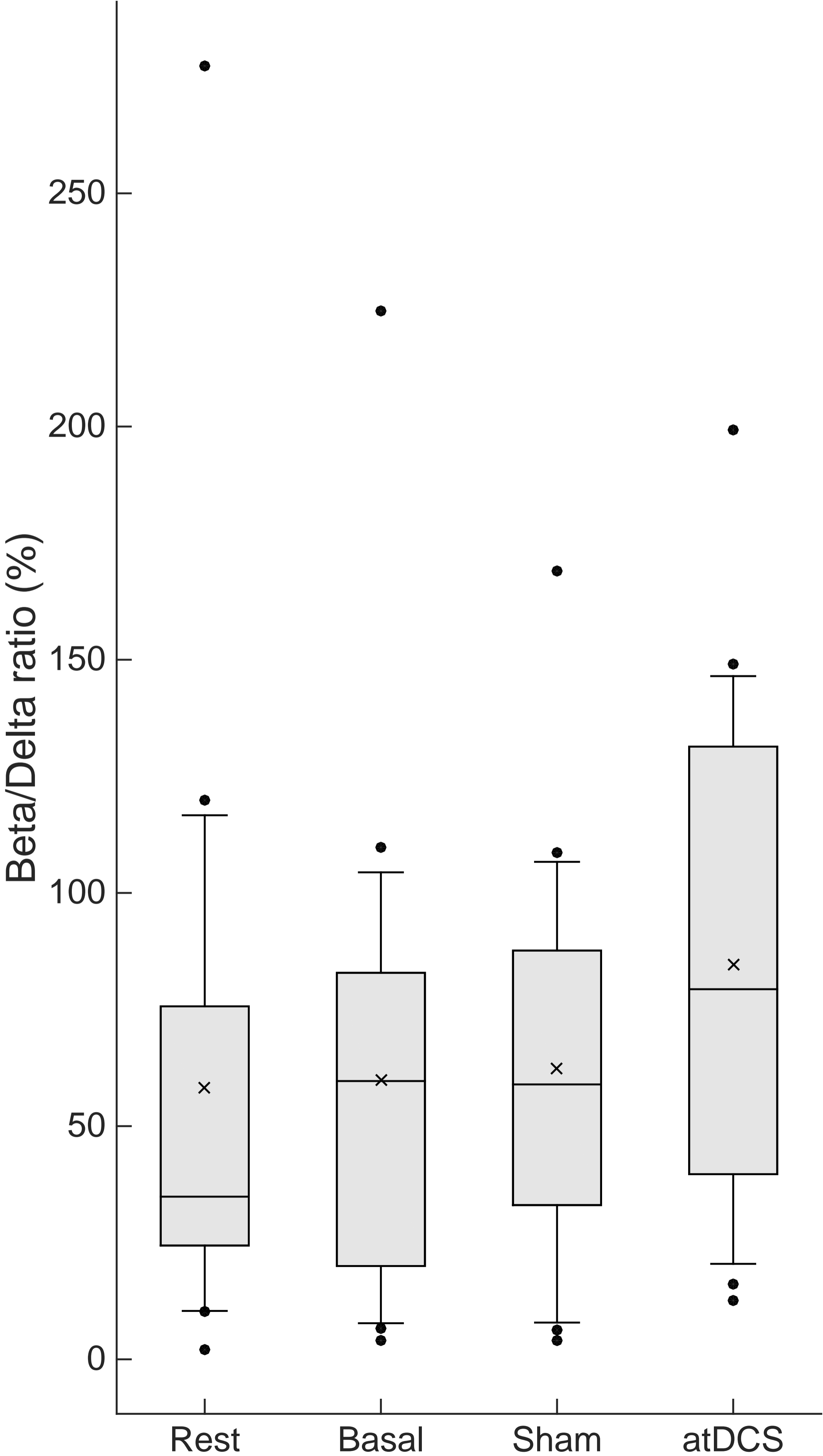

Supplement: Supplementary file 1 [file Data_Sheet_1.zip › Complementary_results/Band_ratios_Complete_EEG/Beta_Delta/Beta-Delta_complete-EEG_P7.pdf]

**Beta/Delta ratio on complete EEG signal for electrode: P8**

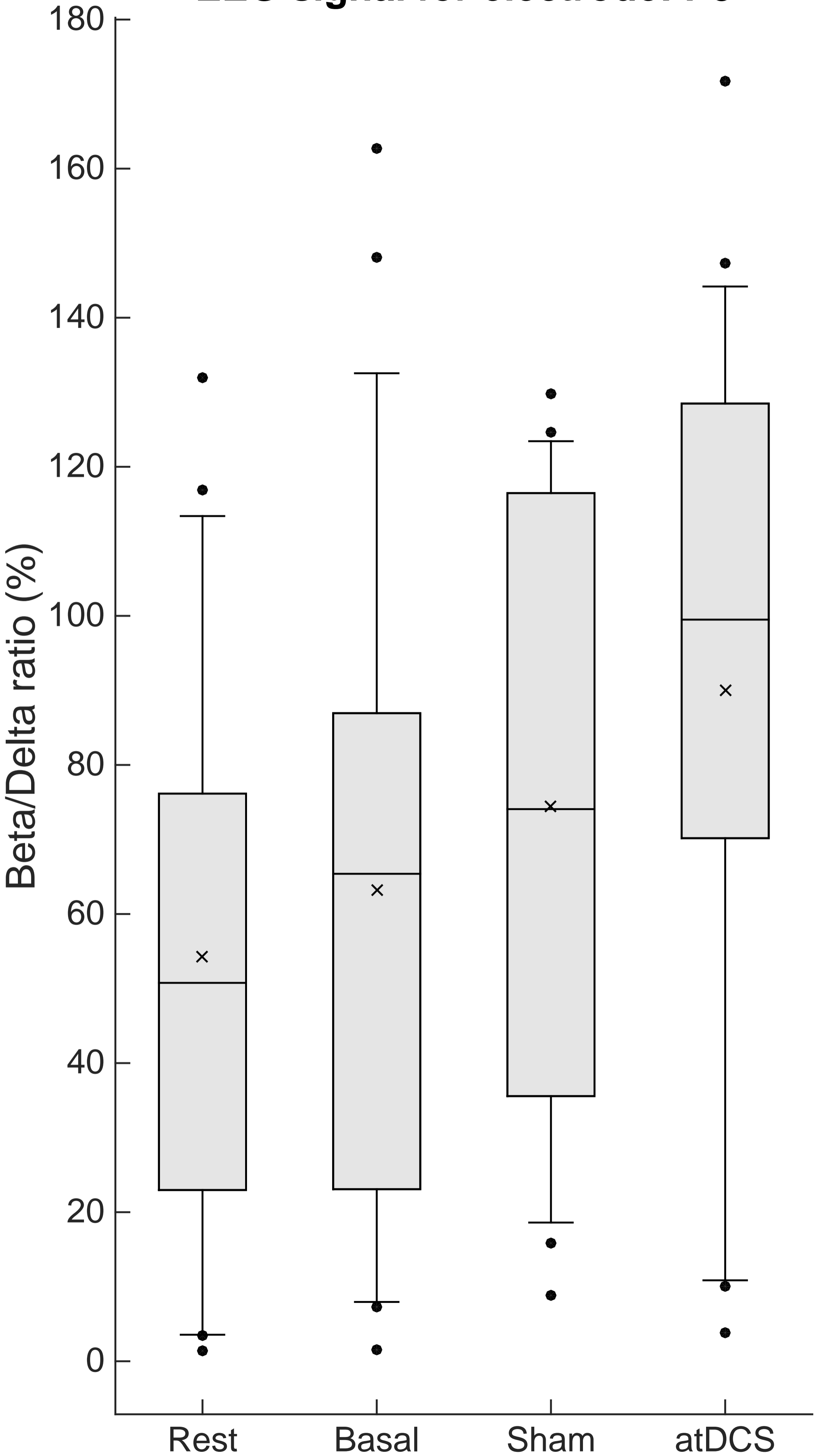

Supplement: Supplementary file 1 [file Data_Sheet_1.zip › Complementary_results/Band_ratios_Complete_EEG/Beta_Delta/Beta-Delta_complete-EEG_P8.pdf]

**Beta/Delta ratio on complete EEG signal for electrode: T7**

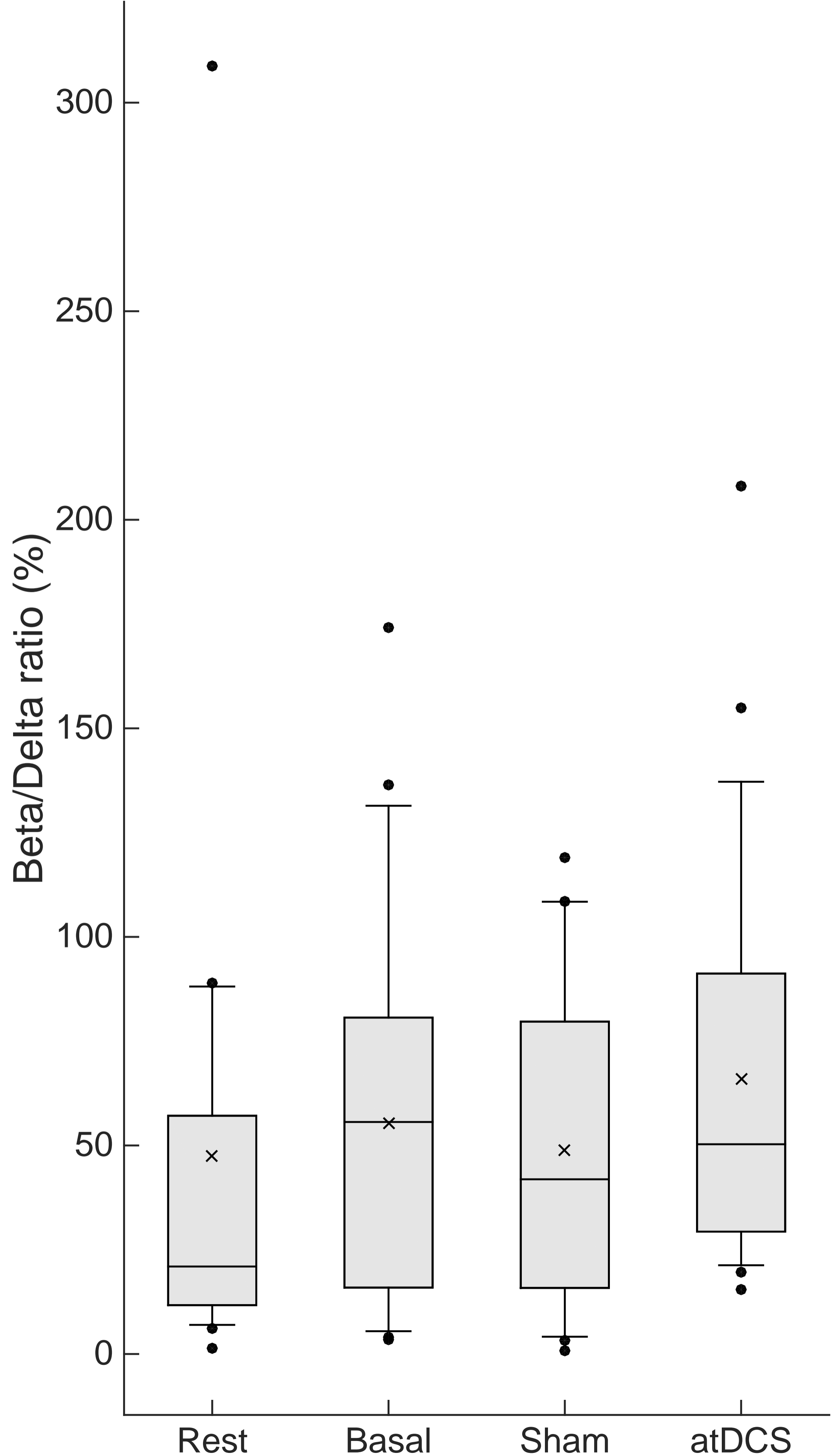

Supplement: Supplementary file 1 [file Data_Sheet_1.zip › Complementary_results/Band_ratios_Complete_EEG/Beta_Delta/Beta-Delta_complete-EEG_T7.pdf]

**Beta/Delta ratio on complete EEG signal for electrode: T8**

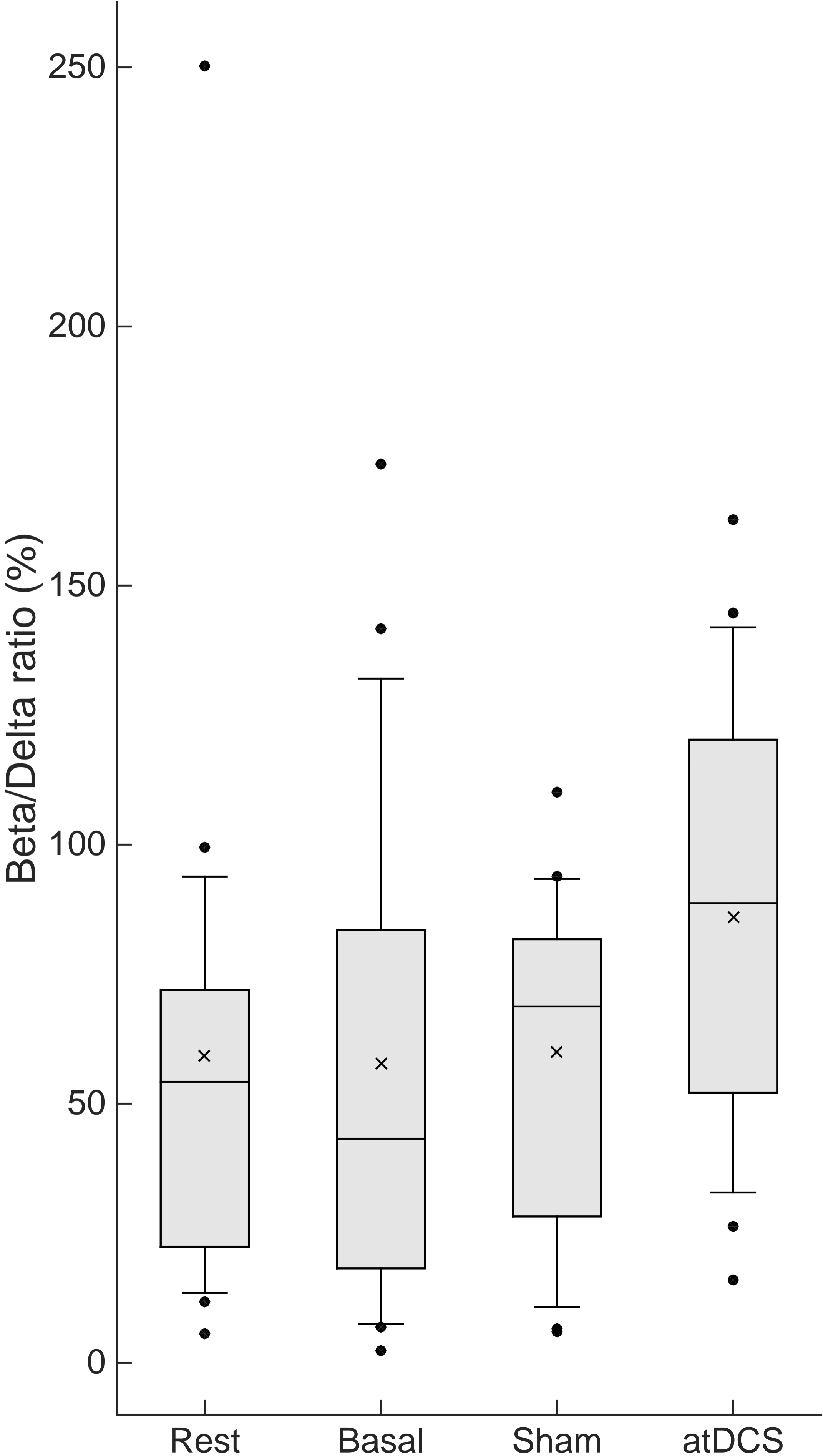

Supplement: Supplementary file 1 [file Data_Sheet_1.zip › Complementary_results/Band_ratios_Complete_EEG/Beta_Delta/Beta-Delta_complete-EEG_T8.pdf]

# Gamma/Alpha ratio on complete EEG signal for electrode: AF3

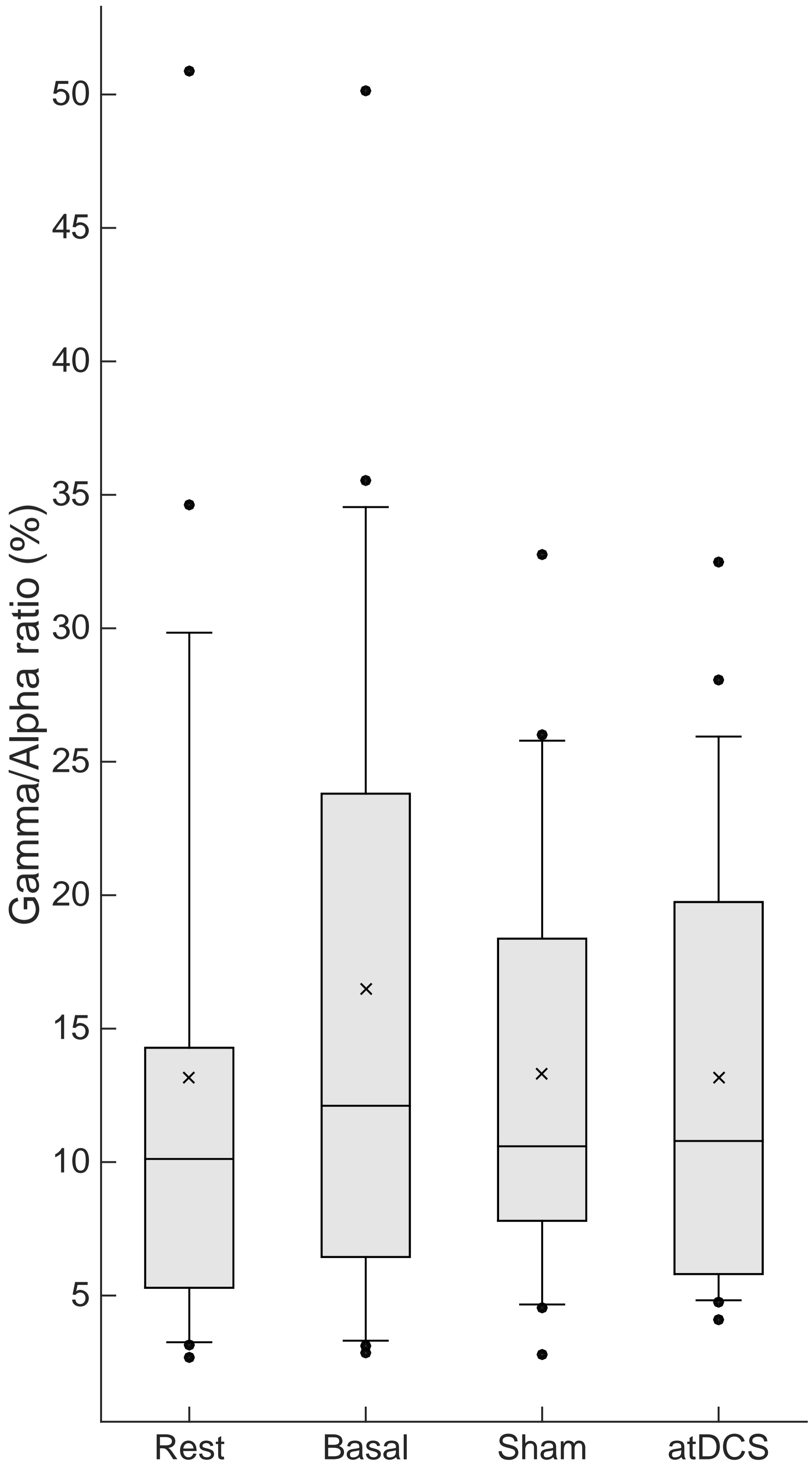

Supplement: Supplementary file 1 [file Data_Sheet_1.zip › Complementary_results/Band_ratios_Complete_EEG/Gamma_Alpha/Gamma-Alpha_complete-EEG_AF3.pdf]

# Gamma/Alpha ratio on complete EEG signal for electrode: AF4

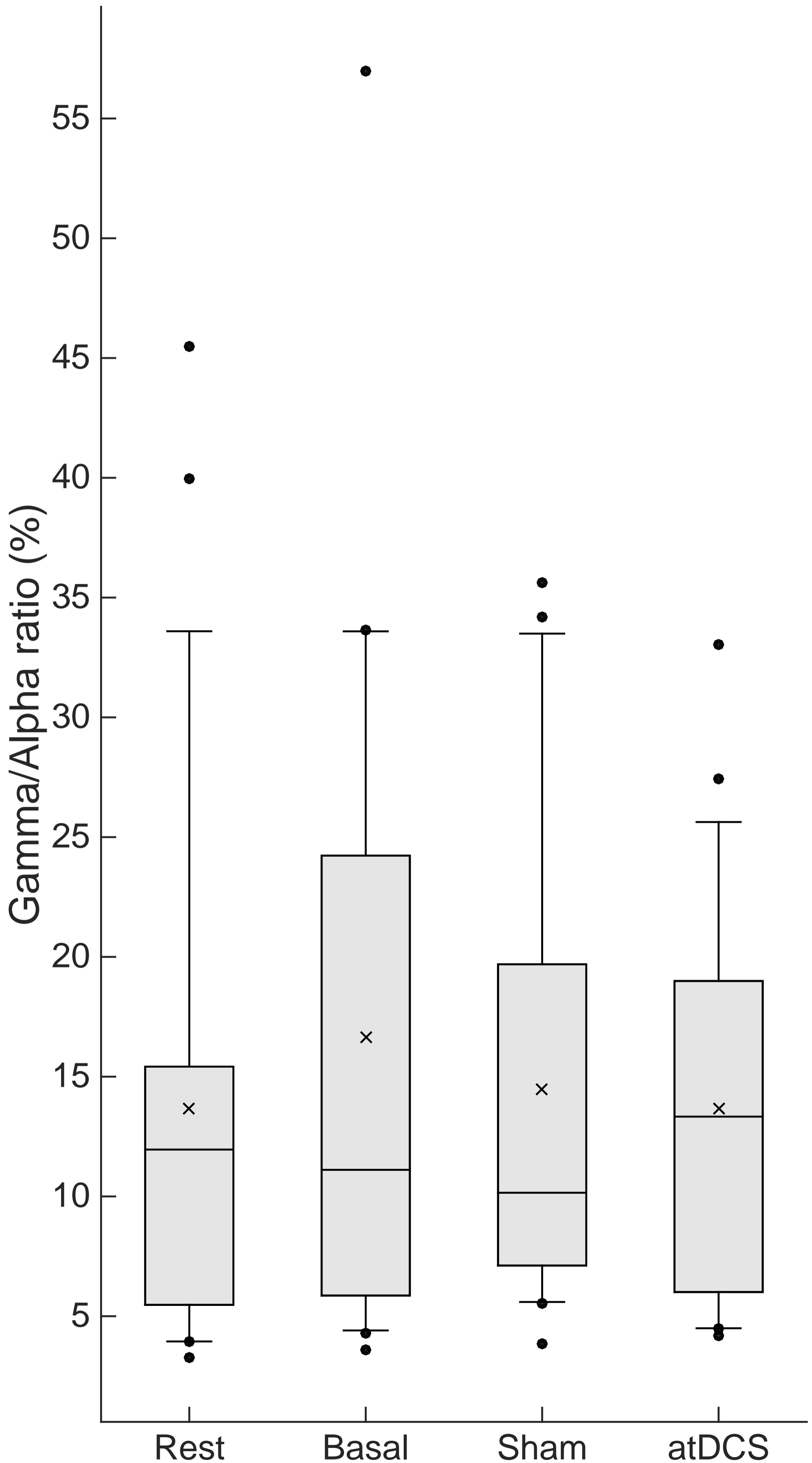

Supplement: Supplementary file 1 [file Data_Sheet_1.zip › Complementary_results/Band_ratios_Complete_EEG/Gamma_Alpha/Gamma-Alpha_complete-EEG_AF4.pdf]

**Gamma/Alpha ratio on complete EEG signal for electrode: Avg AF3-F3-F7**

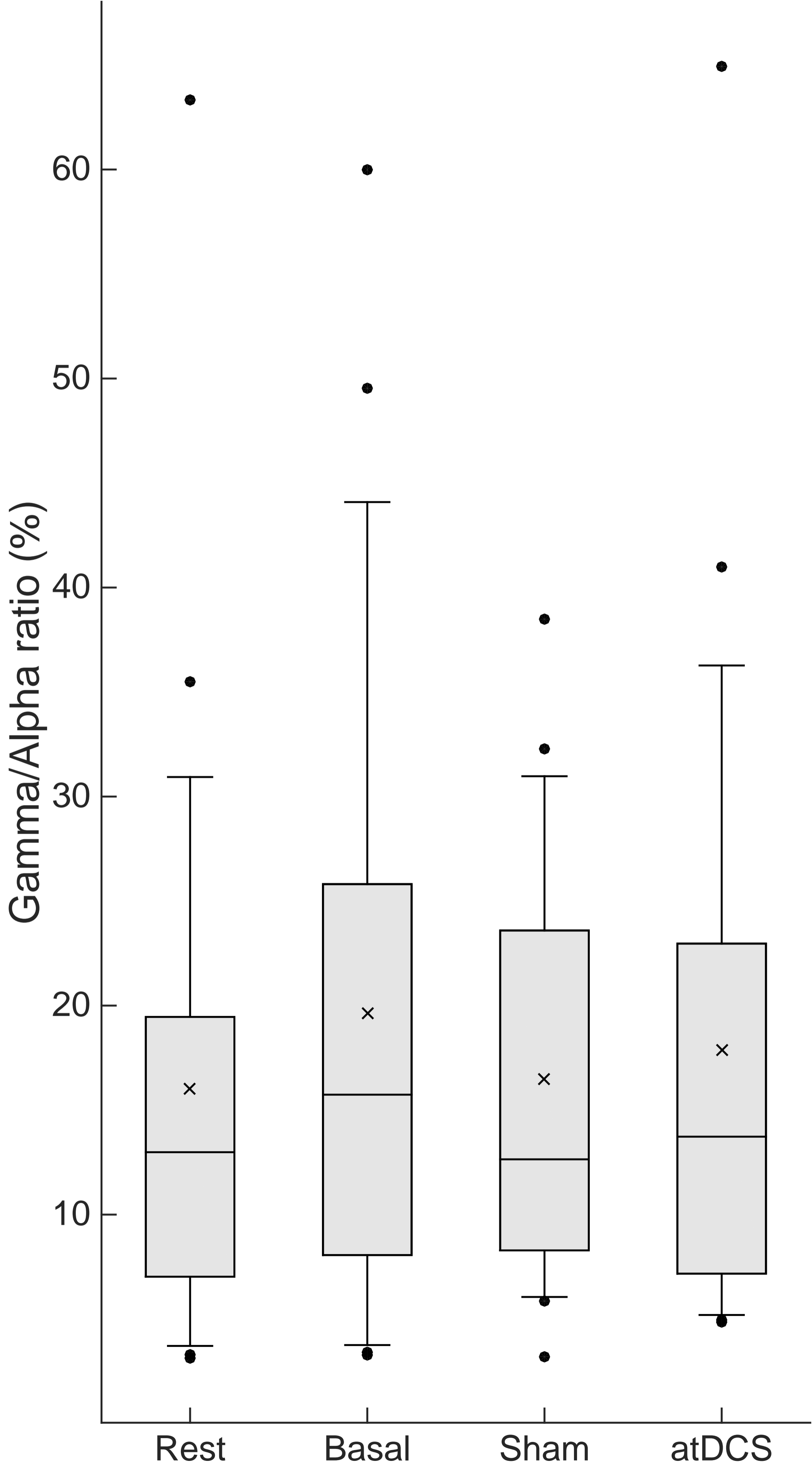

Supplement: Supplementary file 1 [file Data_Sheet_1.zip › Complementary_results/Band_ratios_Complete_EEG/Gamma_Alpha/Gamma-Alpha_complete-EEG_Avg AF3-F3-F7.pdf]

**Gamma/Alpha ratio on complete EEG signal for electrode: Avg AF4-F4-F8**

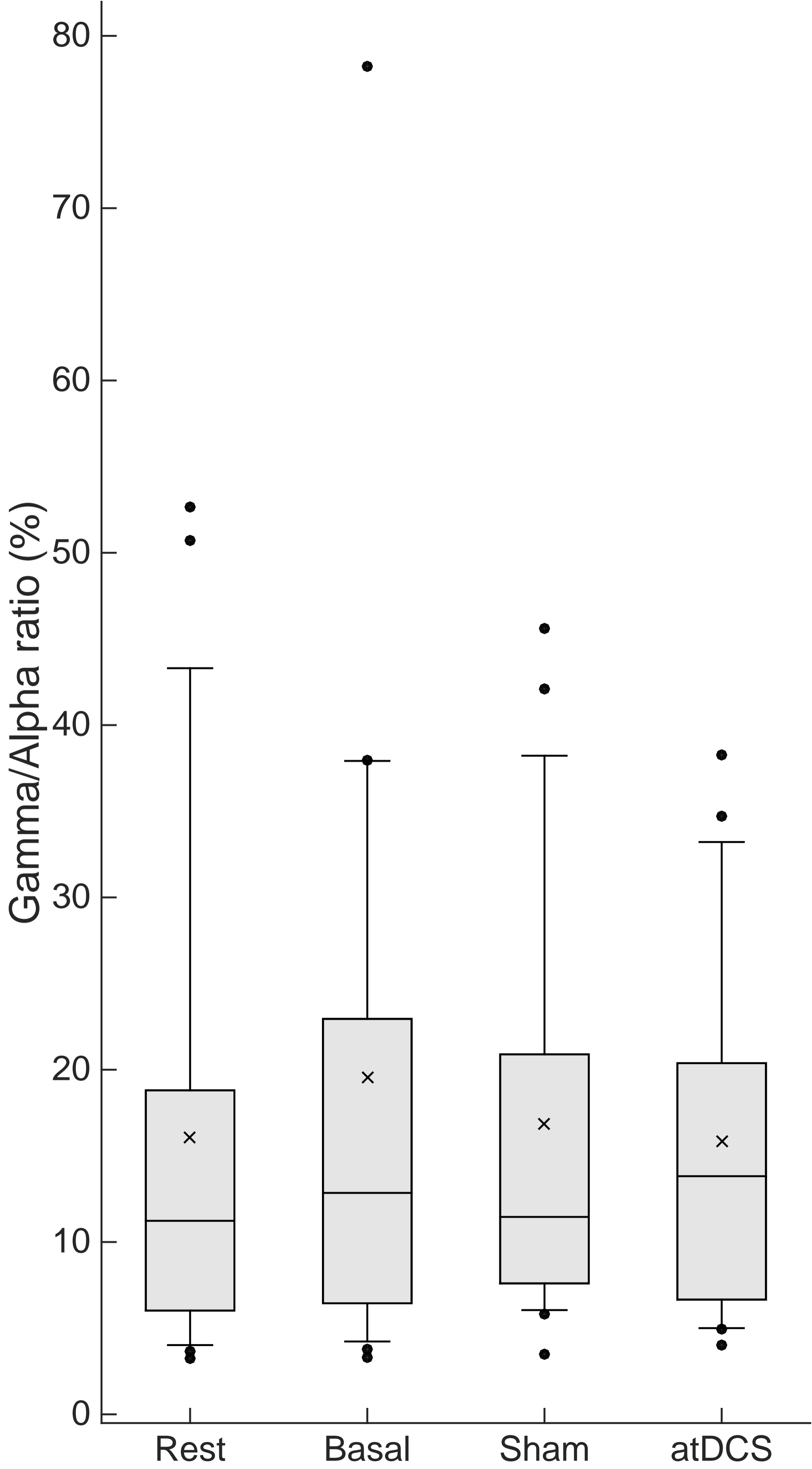

Supplement: Supplementary file 1 [file Data_Sheet_1.zip › Complementary_results/Band_ratios_Complete_EEG/Gamma_Alpha/Gamma-Alpha_complete-EEG_Avg AF4-F4-F8.pdf]

**Gamma/Alpha ratio on complete EEG signal for electrode: Avg F3-F7-FC5**

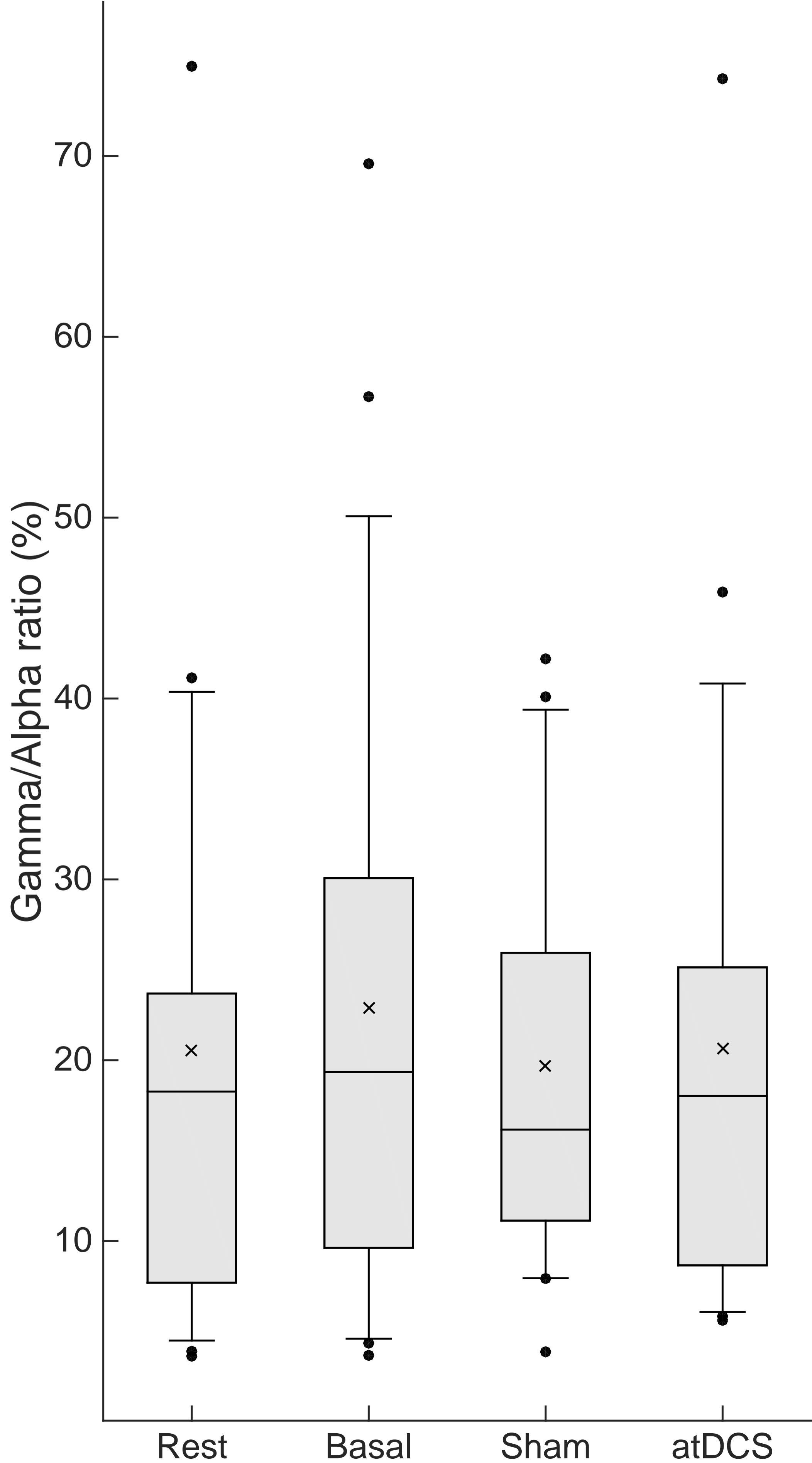

Supplement: Supplementary file 1 [file Data_Sheet_1.zip › Complementary_results/Band_ratios_Complete_EEG/Gamma_Alpha/Gamma-Alpha_complete-EEG_Avg F3-F7-FC5.pdf]

**Gamma/Alpha ratio on complete EEG signal for electrode: Avg F4-F8-FC6**

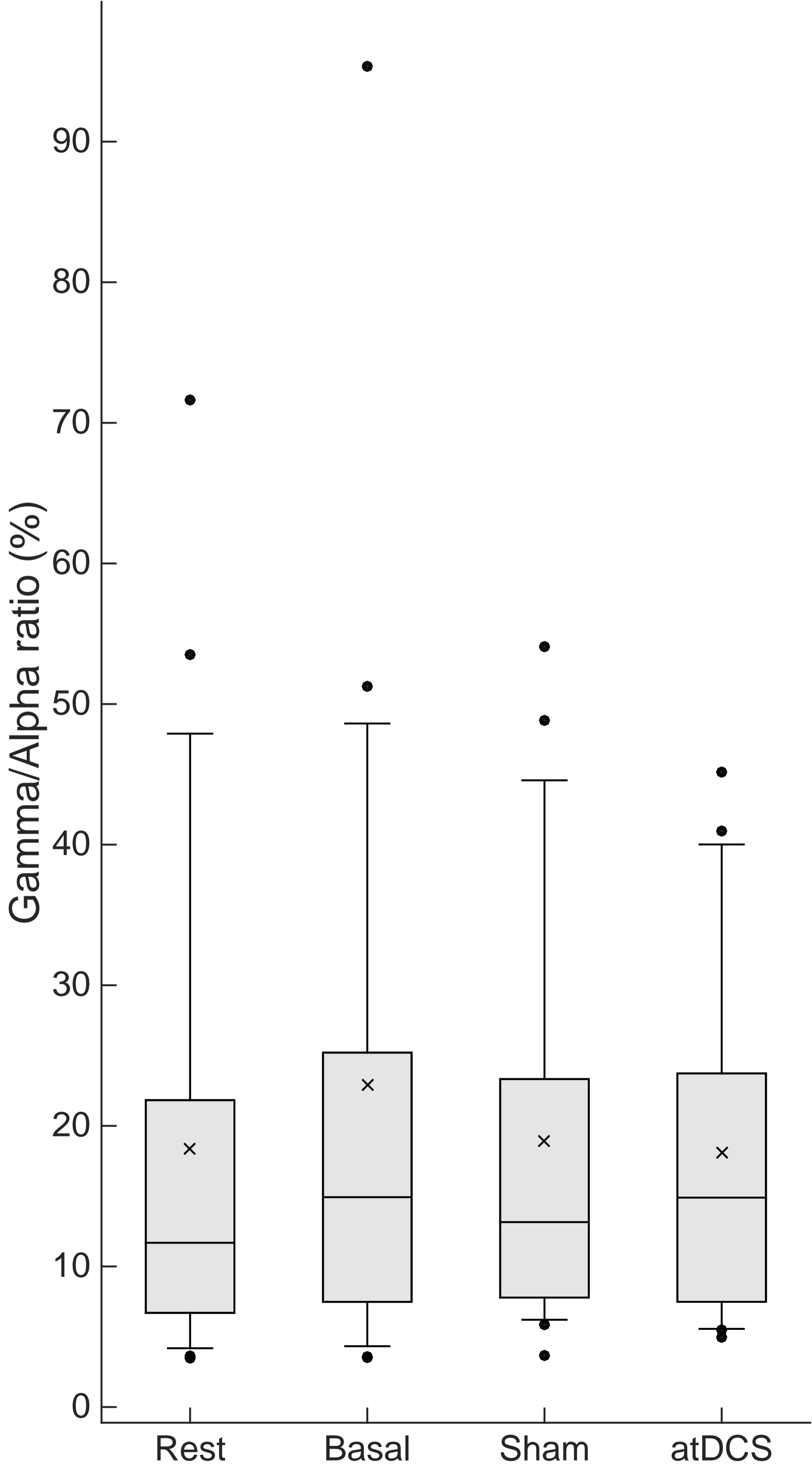

Supplement: Supplementary file 1 [file Data_Sheet_1.zip › Complementary_results/Band_ratios_Complete_EEG/Gamma_Alpha/Gamma-Alpha_complete-EEG_Avg F4-F8-FC6.pdf]

# Gamma/Alpha ratio on complete EEG signal for electrode: F3

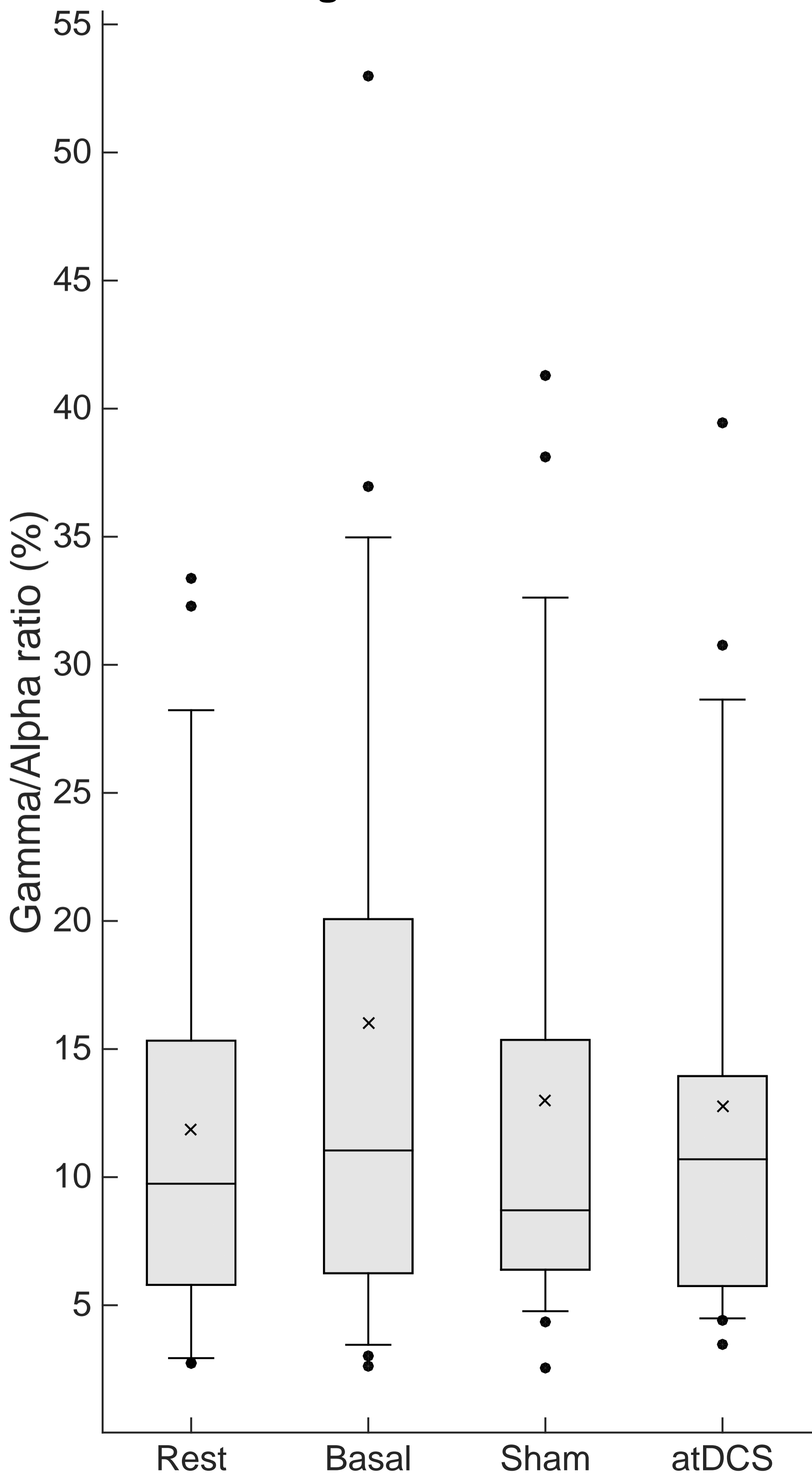

Supplement: Supplementary file 1 [file Data_Sheet_1.zip › Complementary_results/Band_ratios_Complete_EEG/Gamma_Alpha/Gamma-Alpha_complete-EEG_F3.pdf]

# Gamma/Alpha ratio on complete EEG signal for electrode: F4

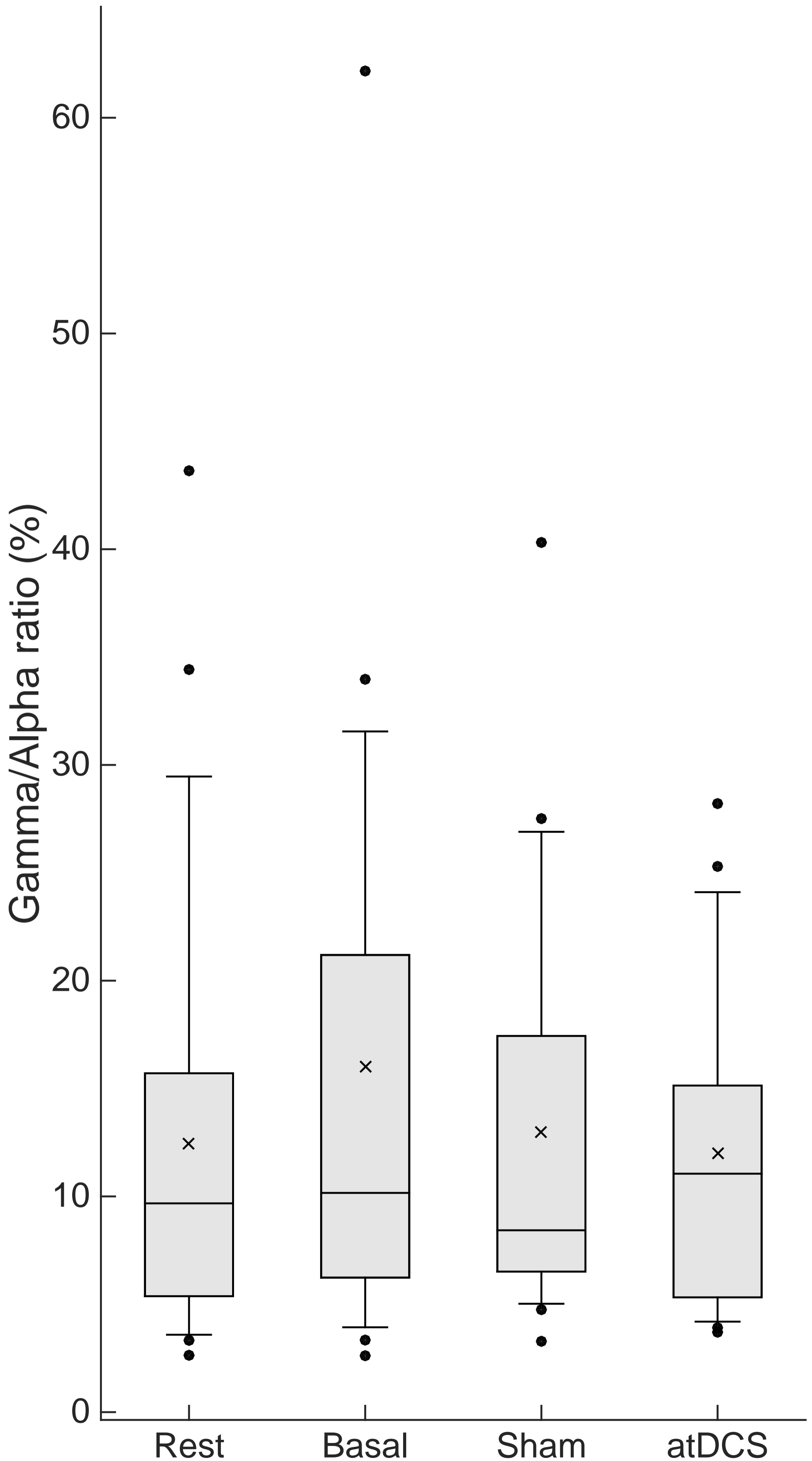

Supplement: Supplementary file 1 [file Data_Sheet_1.zip › Complementary_results/Band_ratios_Complete_EEG/Gamma_Alpha/Gamma-Alpha_complete-EEG_F4.pdf]

**Gamma/Alpha ratio on complete EEG signal for electrode: F7**

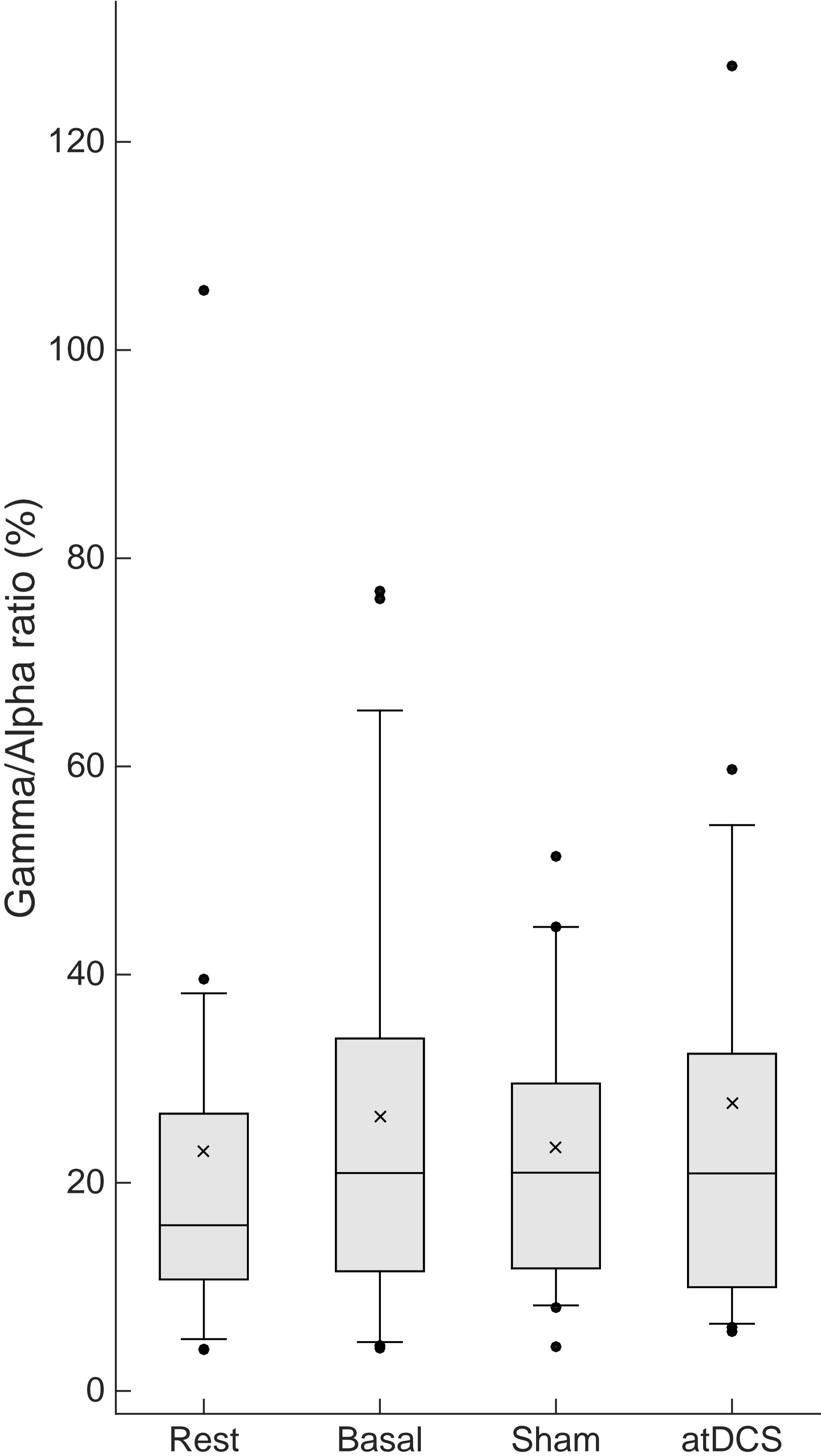

Supplement: Supplementary file 1 [file Data_Sheet_1.zip › Complementary_results/Band_ratios_Complete_EEG/Gamma_Alpha/Gamma-Alpha_complete-EEG_F7.pdf]

# Gamma/Alpha ratio on complete EEG signal for electrode: F8

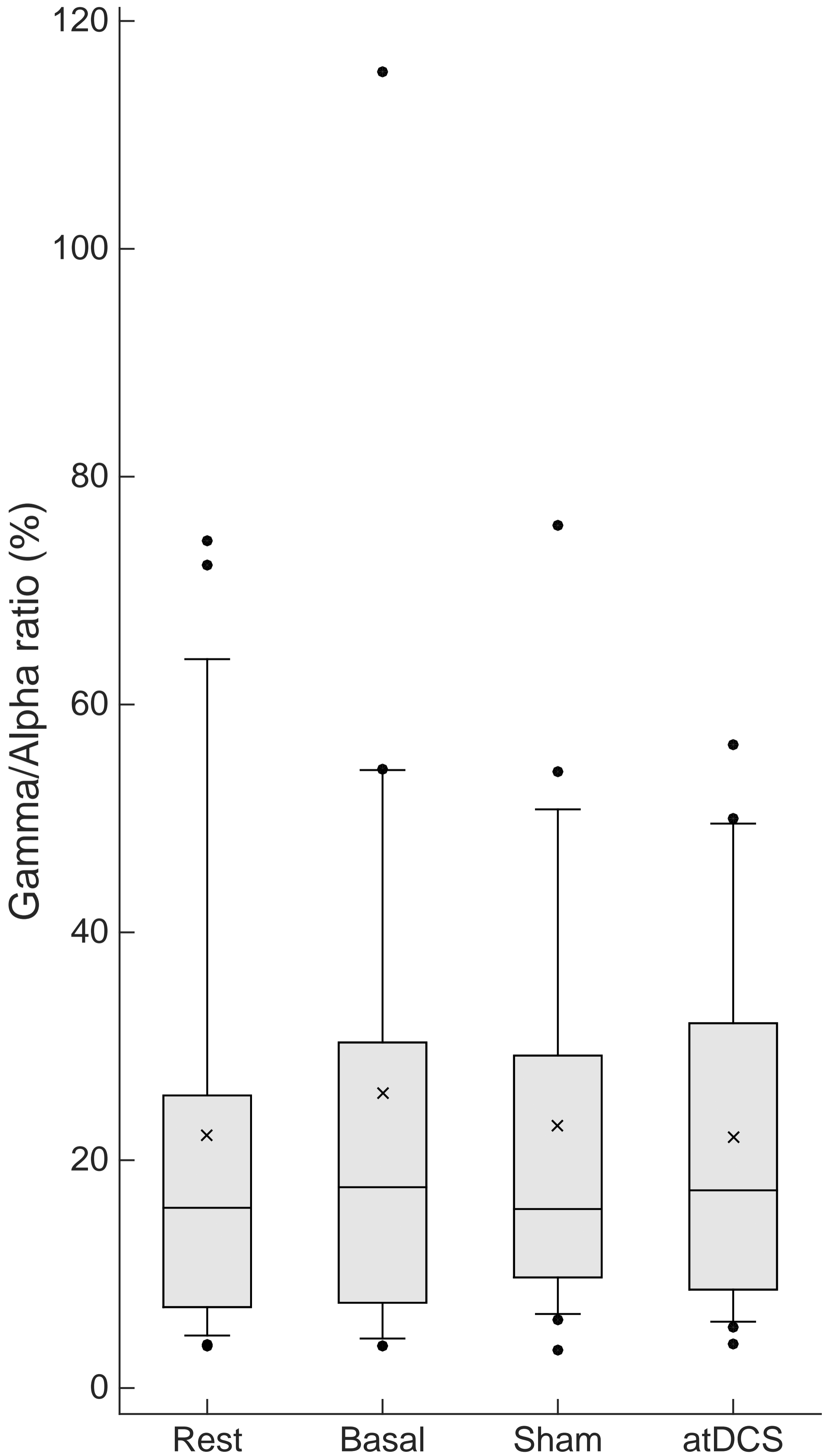

Supplement: Supplementary file 1 [file Data_Sheet_1.zip › Complementary_results/Band_ratios_Complete_EEG/Gamma_Alpha/Gamma-Alpha_complete-EEG_F8.pdf]

**Gamma/Alpha ratio on complete EEG signal for electrode: FC5**

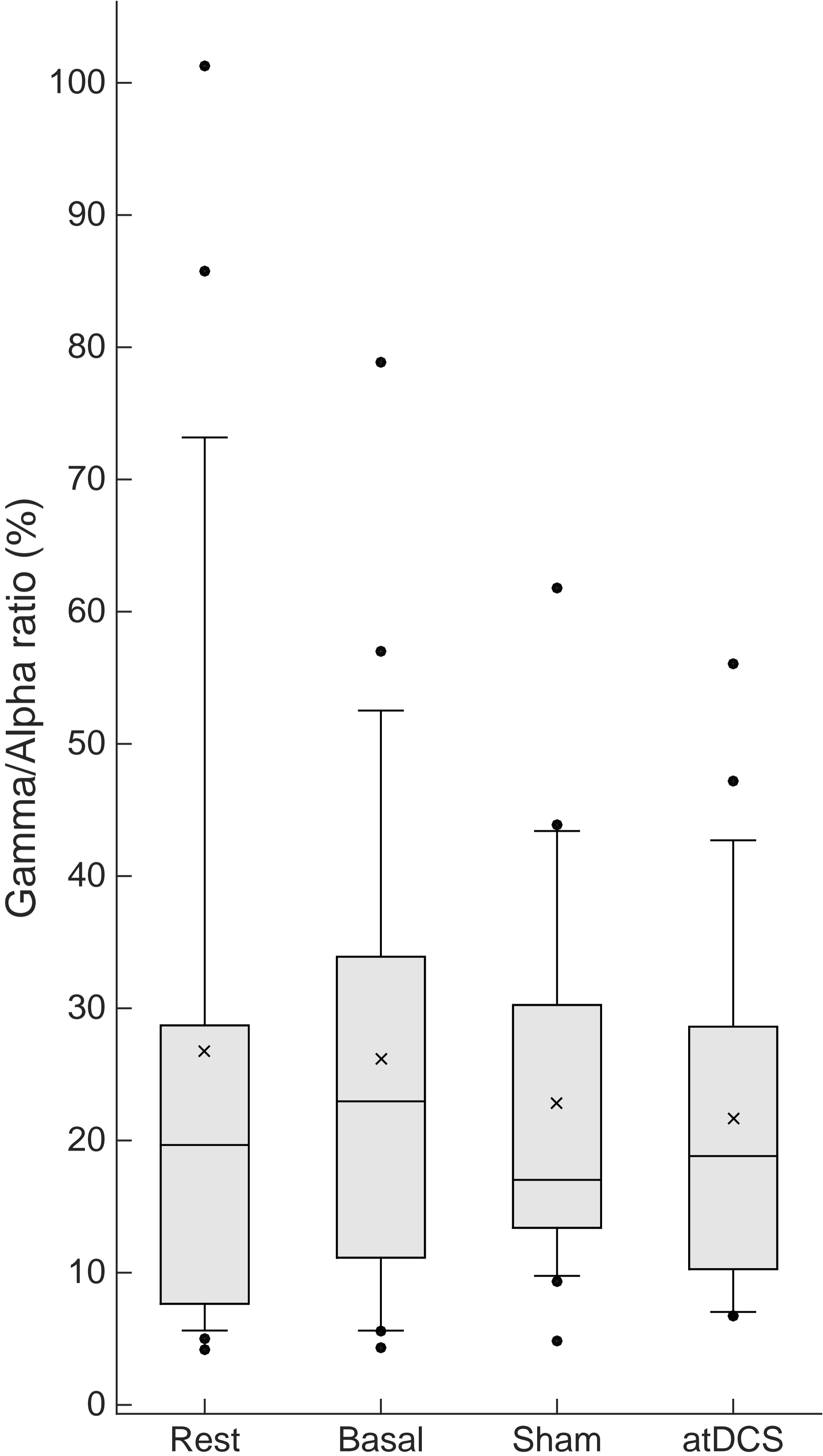

Supplement: Supplementary file 1 [file Data_Sheet_1.zip › Complementary_results/Band_ratios_Complete_EEG/Gamma_Alpha/Gamma-Alpha_complete-EEG_FC5.pdf]

**Gamma/Alpha ratio on complete EEG signal for electrode: FC6**

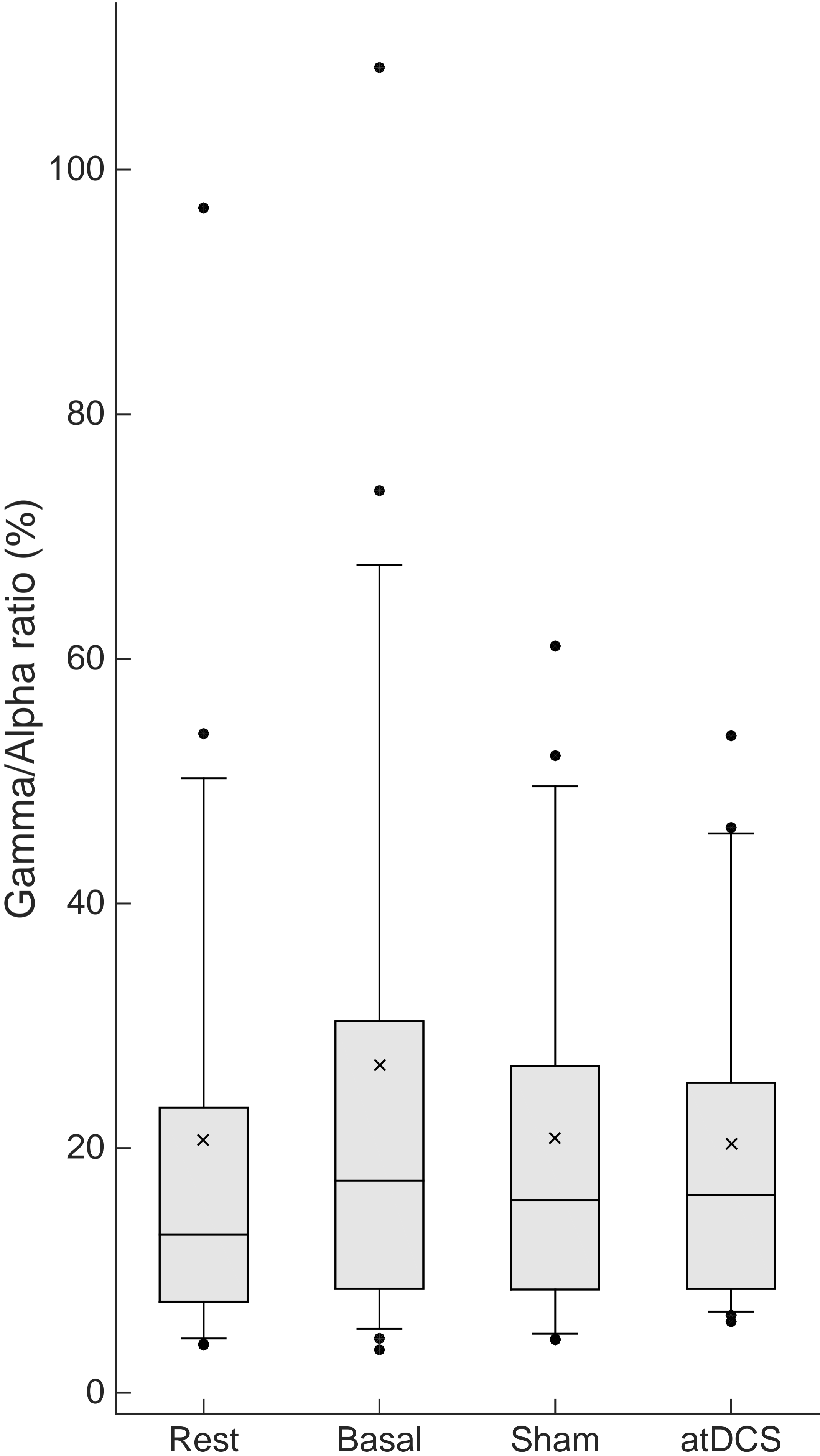

Supplement: Supplementary file 1 [file Data_Sheet_1.zip › Complementary_results/Band_ratios_Complete_EEG/Gamma_Alpha/Gamma-Alpha_complete-EEG_FC6.pdf]

**Gamma/Alpha ratio on complete EEG signal for electrode: O1**

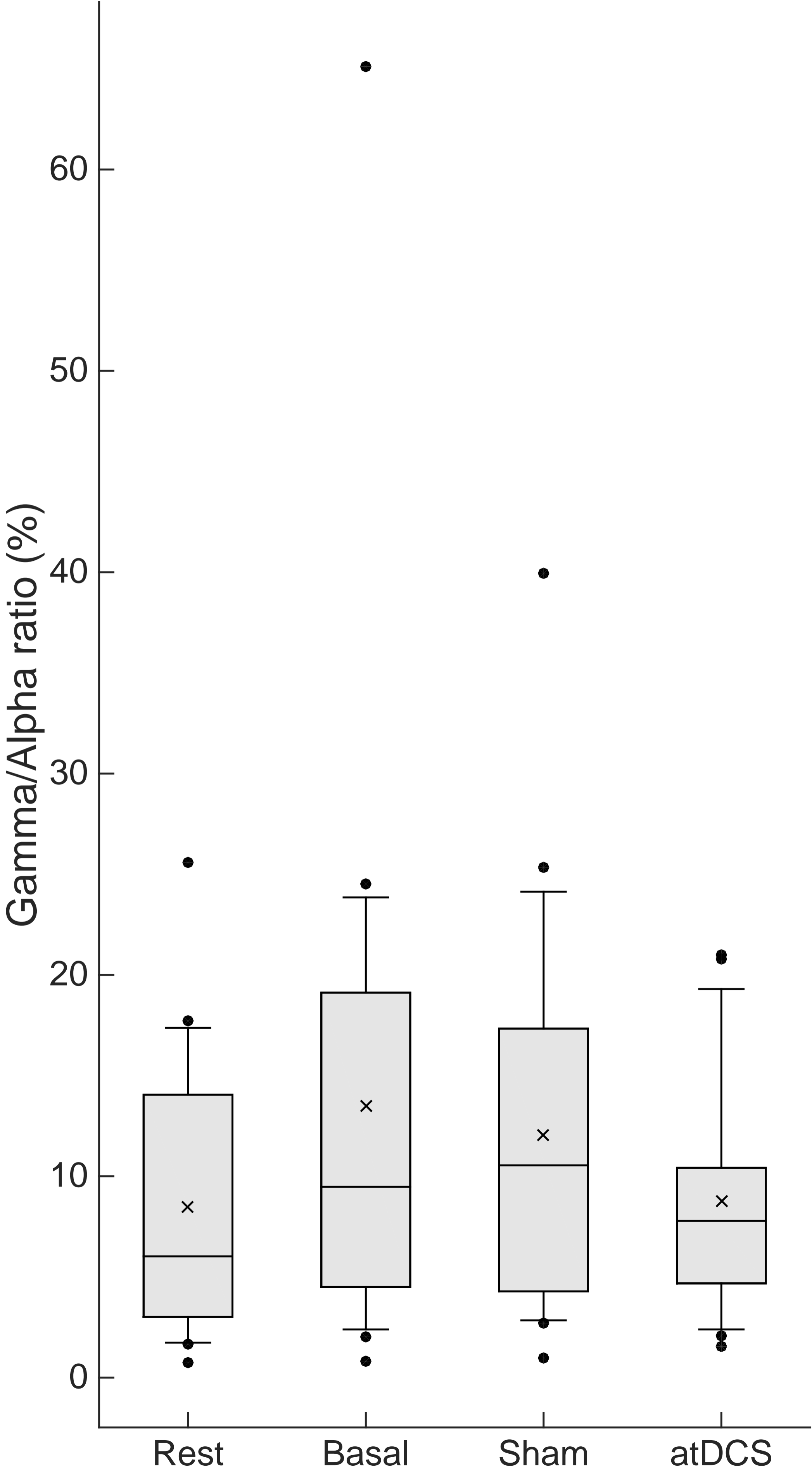

Supplement: Supplementary file 1 [file Data_Sheet_1.zip › Complementary_results/Band_ratios_Complete_EEG/Gamma_Alpha/Gamma-Alpha_complete-EEG_O1.pdf]

**Gamma/Alpha ratio on complete EEG signal for electrode: O2**

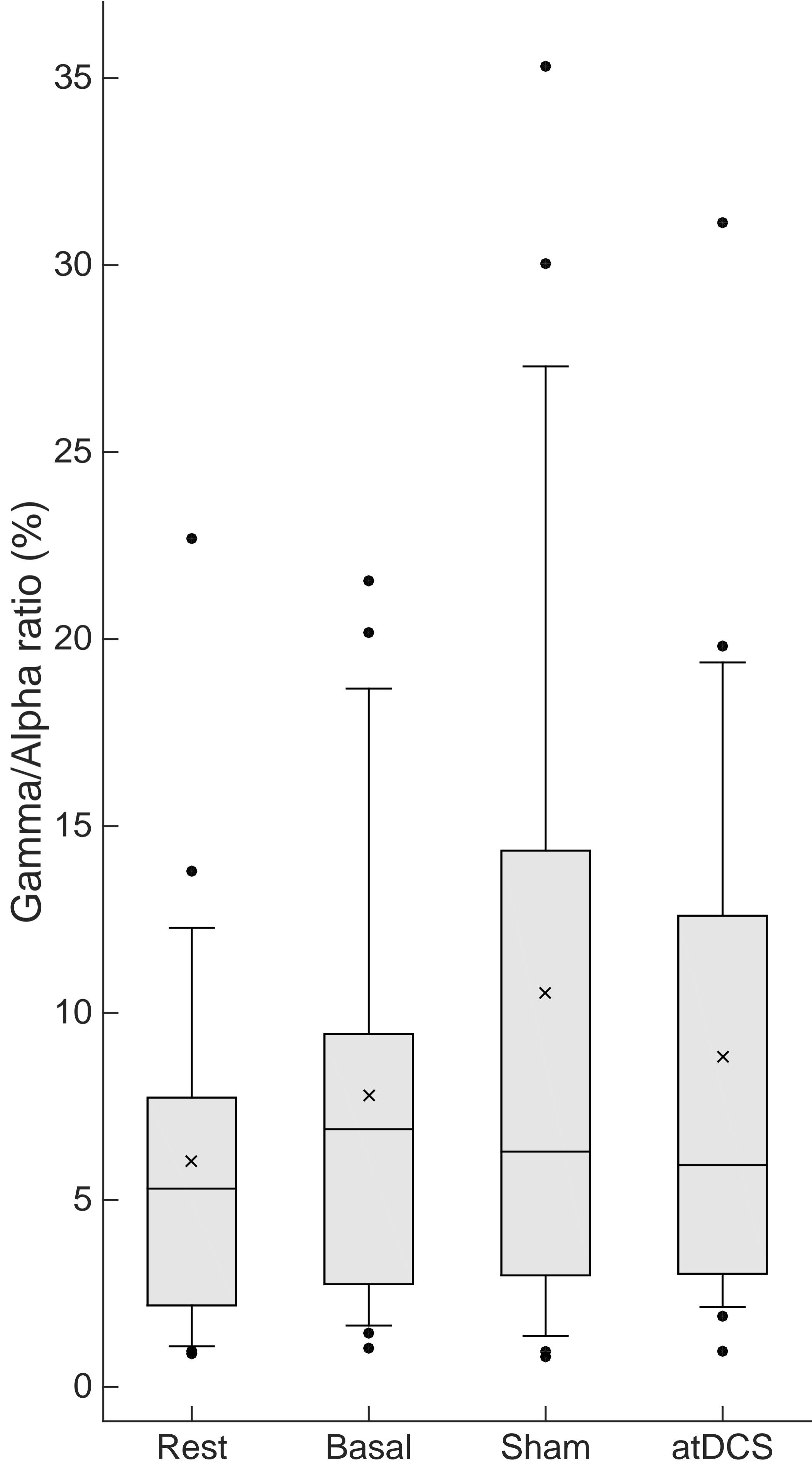

Supplement: Supplementary file 1 [file Data_Sheet_1.zip › Complementary_results/Band_ratios_Complete_EEG/Gamma_Alpha/Gamma-Alpha_complete-EEG_O2.pdf]

# Gamma/Alpha ratio on complete EEG signal for electrode: P7

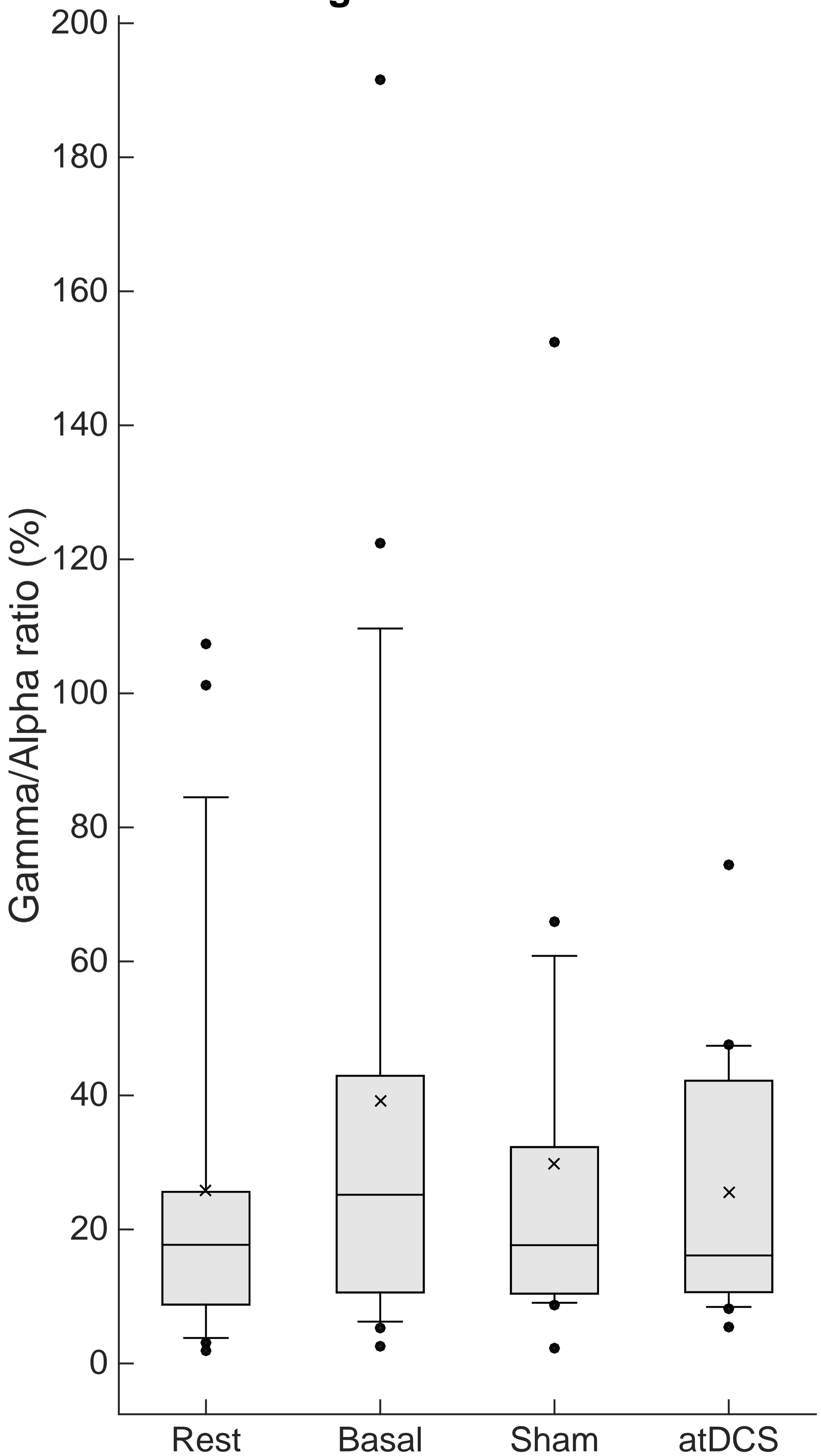

Supplement: Supplementary file 1 [file Data_Sheet_1.zip › Complementary_results/Band_ratios_Complete_EEG/Gamma_Alpha/Gamma-Alpha_complete-EEG_P7.pdf]

**Gamma/Alpha ratio on complete EEG signal for electrode: P8**

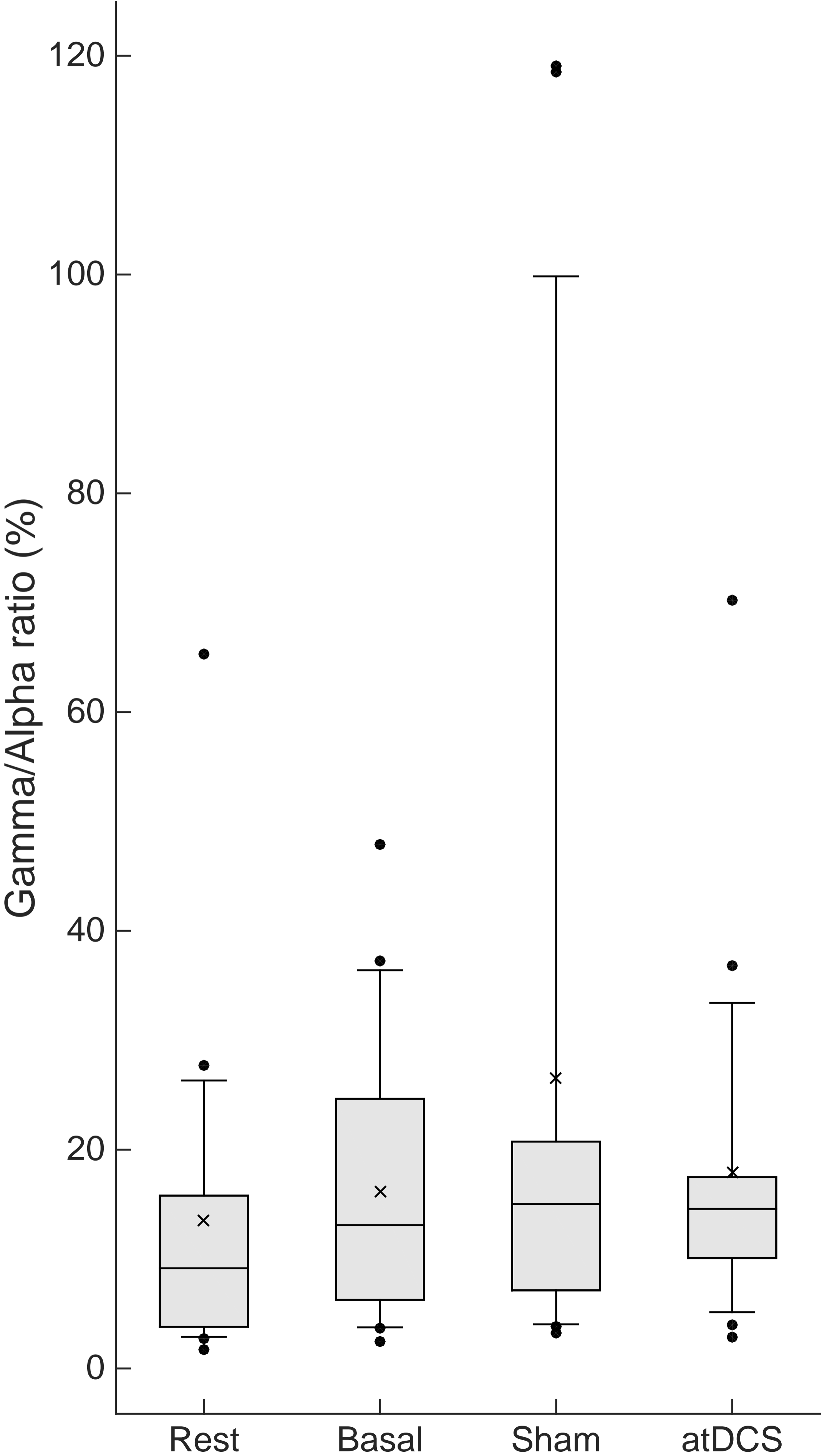

Supplement: Supplementary file 1 [file Data_Sheet_1.zip › Complementary_results/Band_ratios_Complete_EEG/Gamma_Alpha/Gamma-Alpha_complete-EEG_P8.pdf]

**Gamma/Alpha ratio on complete EEG signal for electrode: T7**

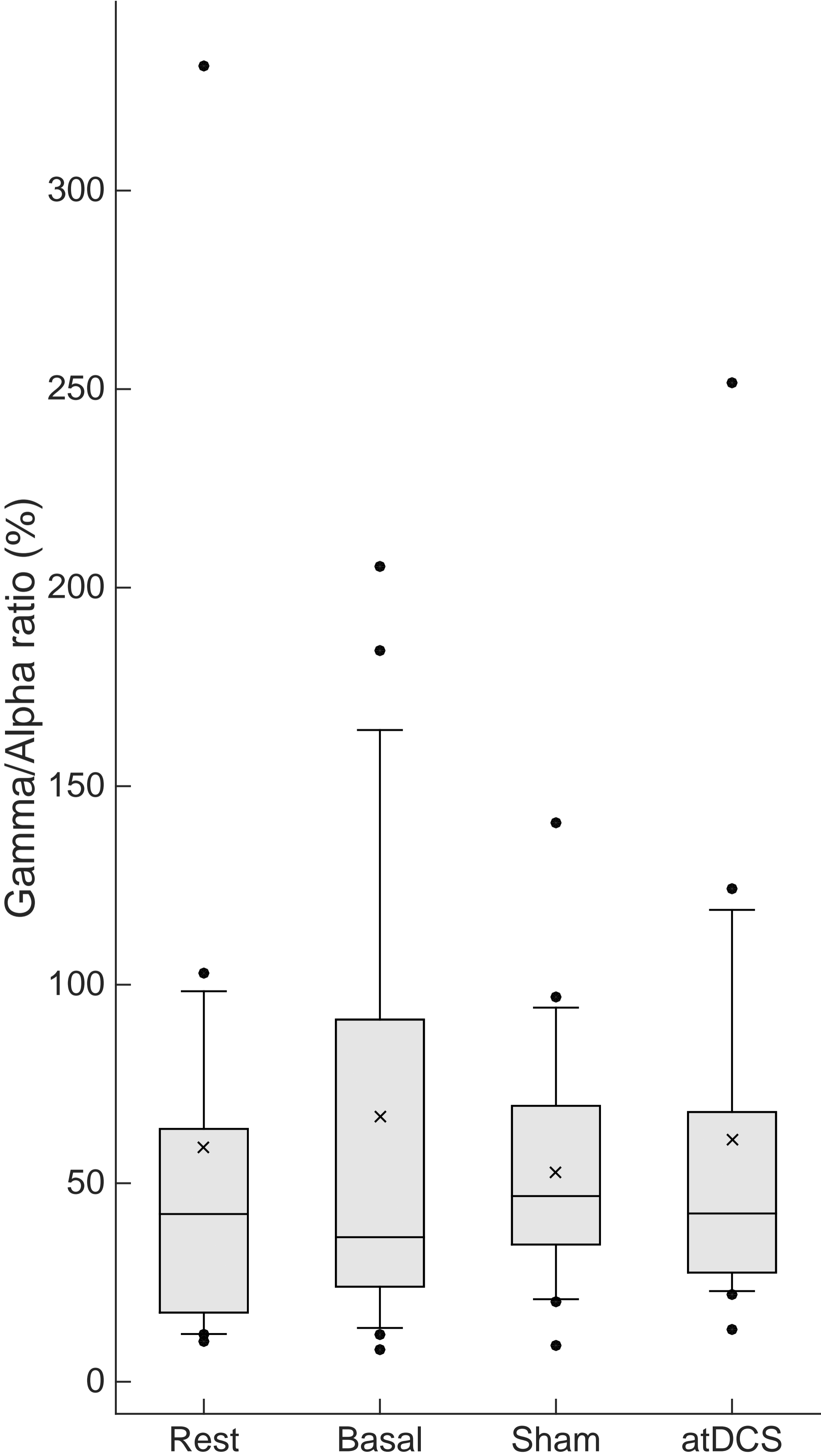

Supplement: Supplementary file 1 [file Data_Sheet_1.zip › Complementary_results/Band_ratios_Complete_EEG/Gamma_Alpha/Gamma-Alpha_complete-EEG_T7.pdf]

**Gamma/Alpha ratio on complete EEG signal for electrode: T8**

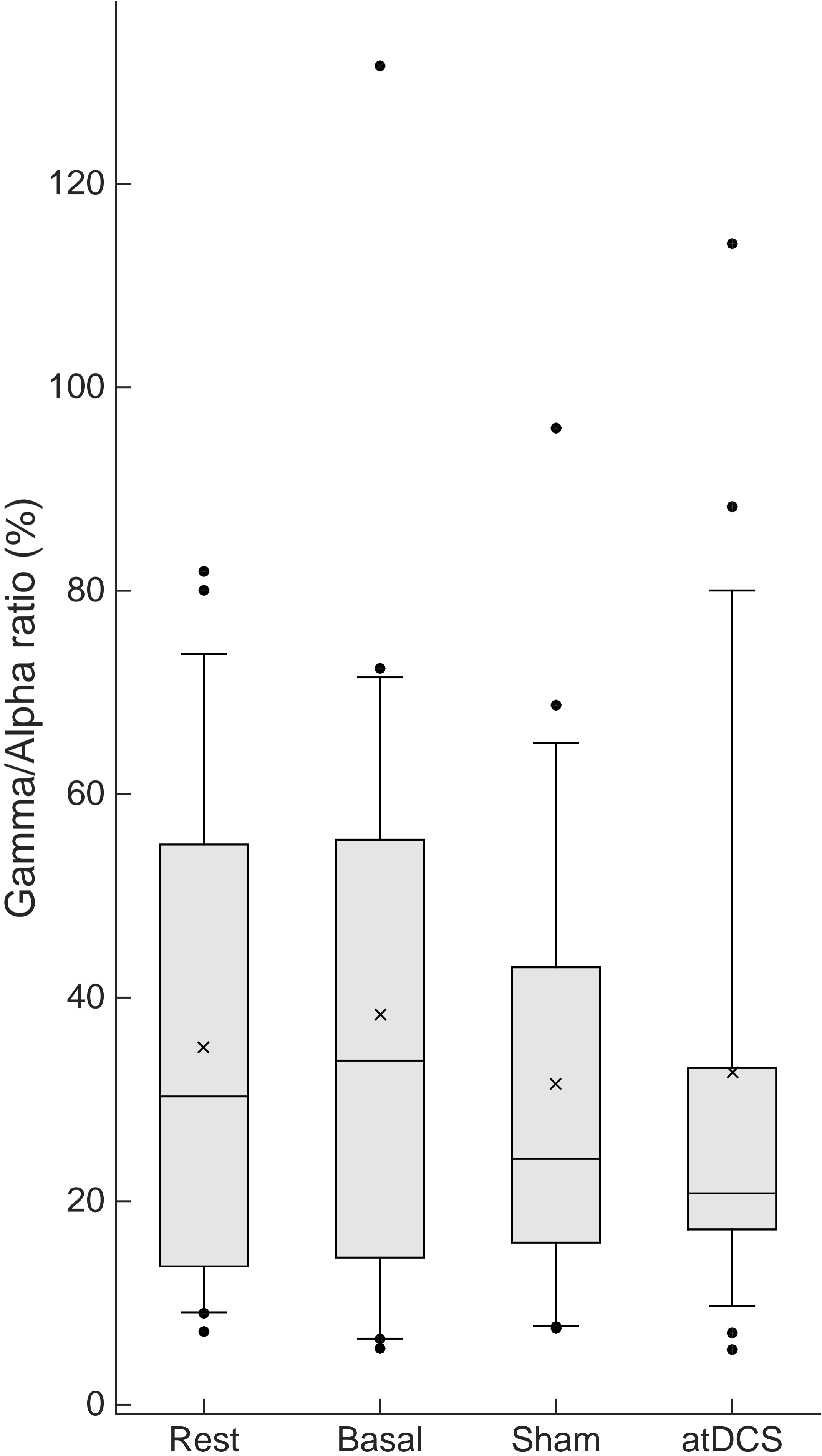

Supplement: Supplementary file 1 [file Data_Sheet_1.zip › Complementary_results/Band_ratios_Complete_EEG/Gamma_Alpha/Gamma-Alpha_complete-EEG_T8.pdf]

**Gamma/Delta ratio on complete EEG signal for electrode: AF3**

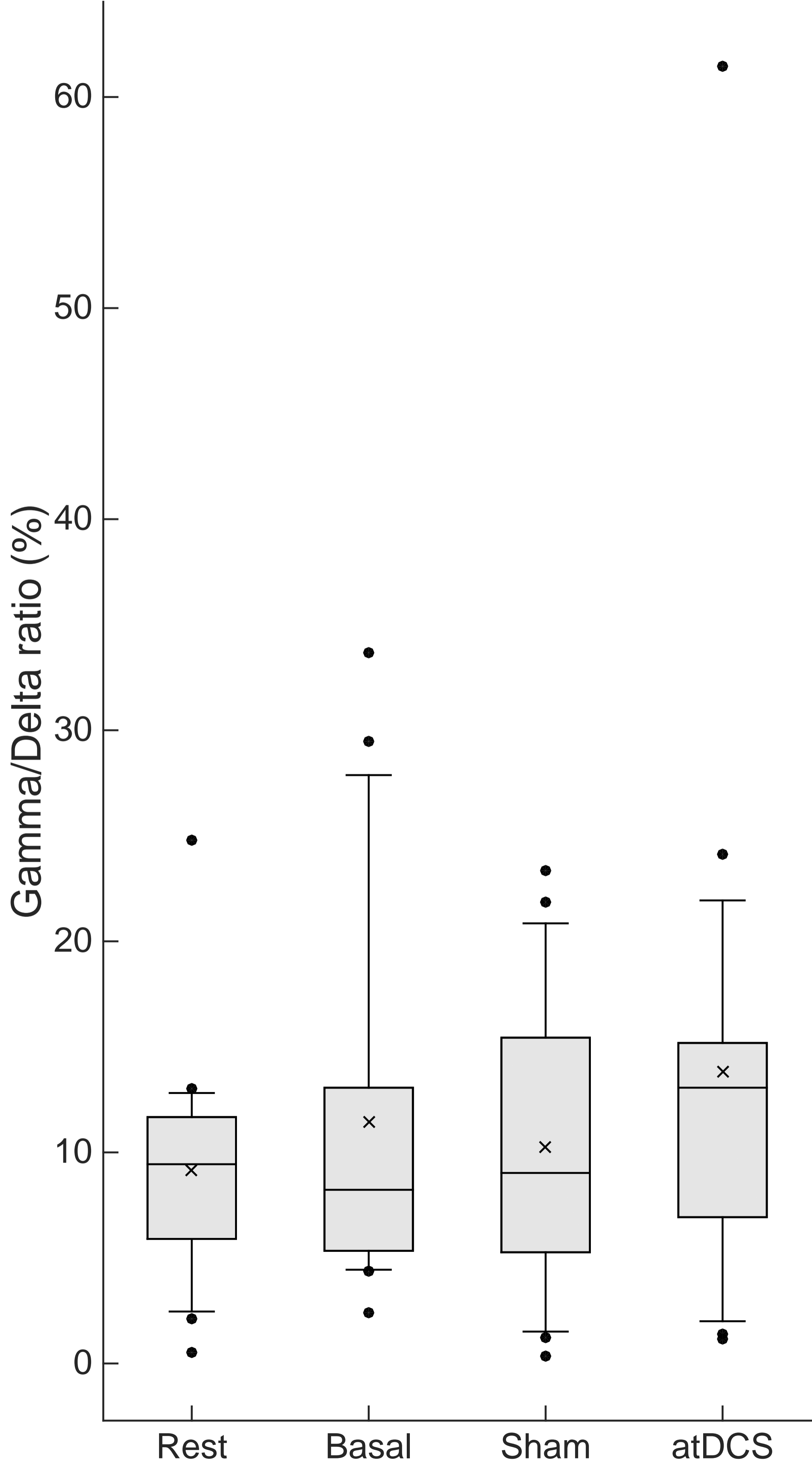

Supplement: Supplementary file 1 [file Data_Sheet_1.zip › Complementary_results/Band_ratios_Complete_EEG/Gamma_Delta/Gamma-Delta_complete-EEG_AF3.pdf]

**Gamma/Delta ratio on complete EEG signal for electrode: AF4**

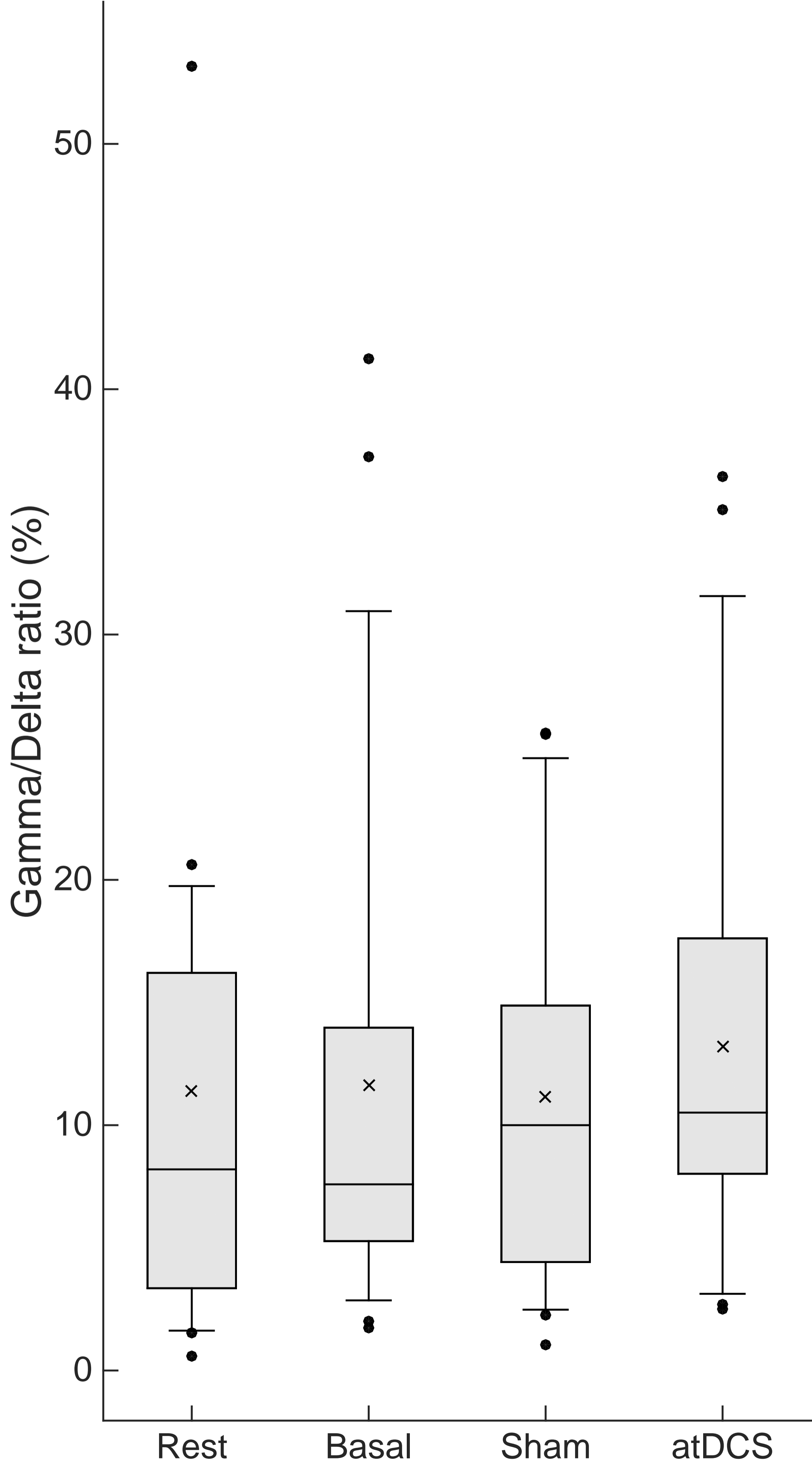

Supplement: Supplementary file 1 [file Data_Sheet_1.zip › Complementary_results/Band_ratios_Complete_EEG/Gamma_Delta/Gamma-Delta_complete-EEG_AF4.pdf]
