# Supplementary figures and images for: A Pilot Study on the Effects of Transcranial Direct Current Stimulation on Brain Rhythms and Entropy during Self-Paced Finger Movement using the Epoc Helmet (part 1 of 2)
Source: Front Hum Neurosci. 2017 Apr 25;11:201. doi: 10.3389/fnhum.2017.00201 (PMC5408787; doi:10.3389/fnhum.2017.00201)

**Alpha/Delta ratio on average  
PSD windows for electrode: AF3**

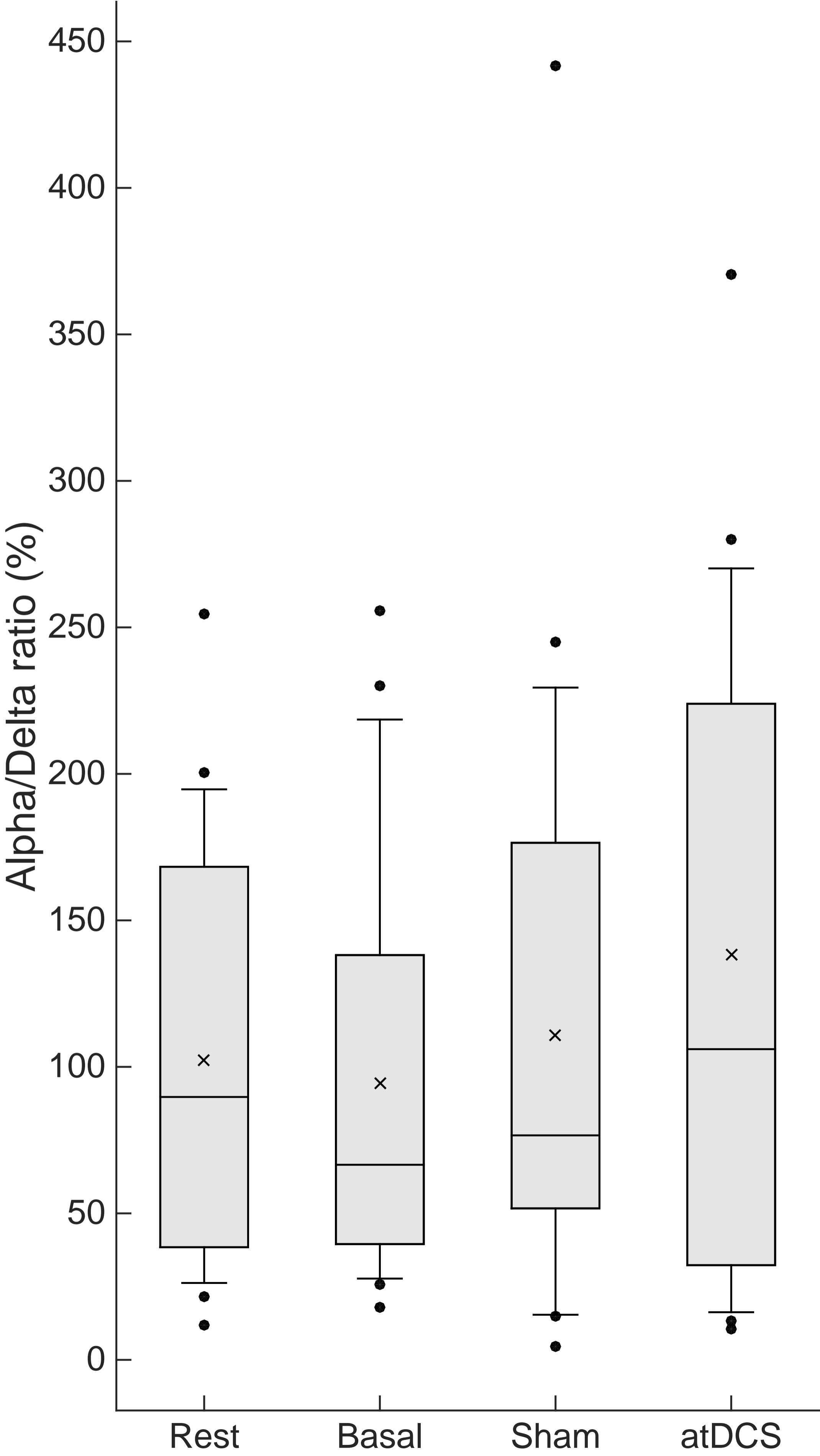

Supplement: Supplementary file 1 [file Data_Sheet_1.zip › Complementary_results/Band_ratios_average_PSD_windows/Alpha_Delta/Alpha-Delta_mean-win_AF3.pdf]

**Alpha/Delta ratio on average  
PSD windows for electrode: AF4**

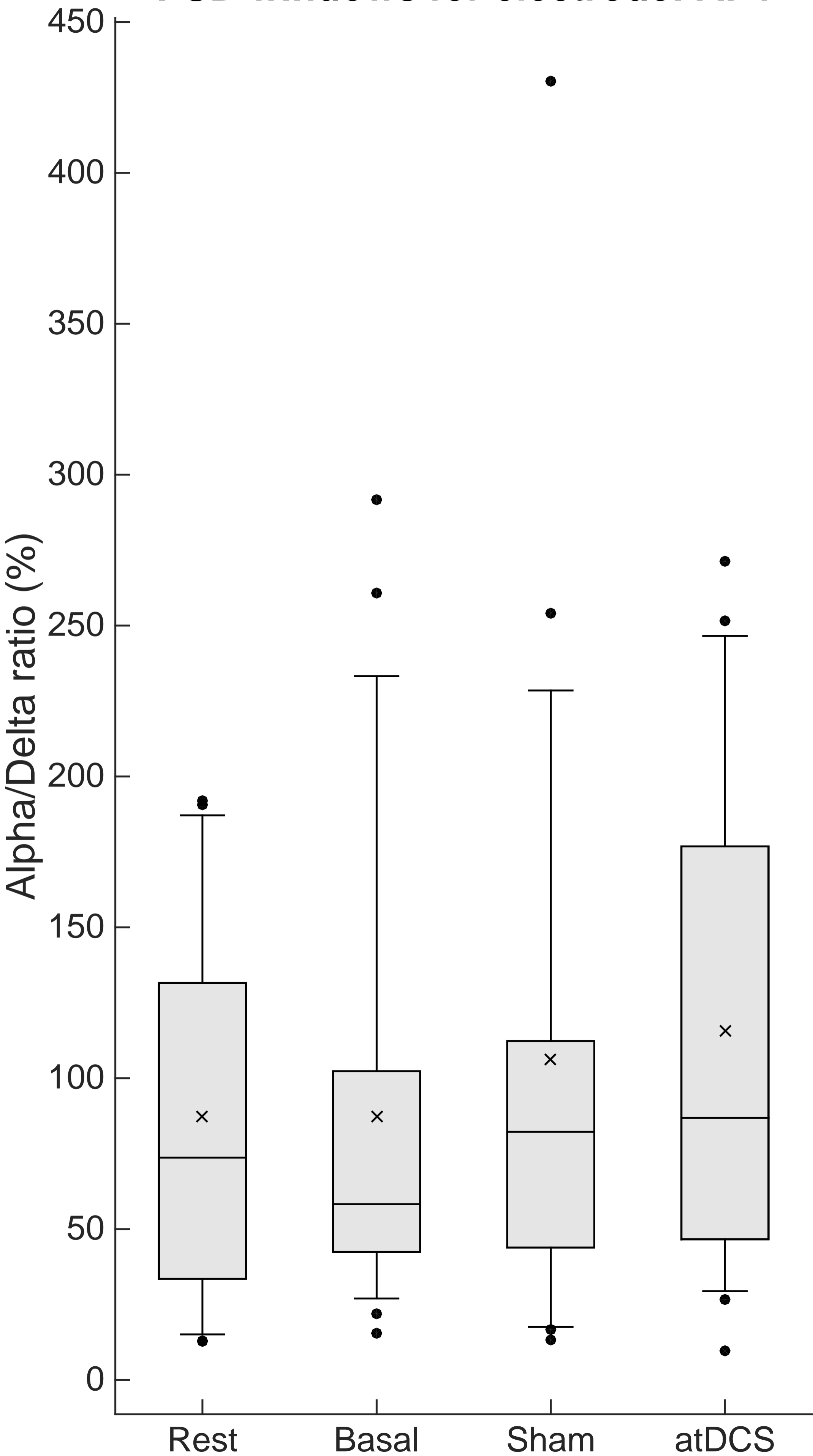

Supplement: Supplementary file 1 [file Data_Sheet_1.zip › Complementary_results/Band_ratios_average_PSD_windows/Alpha_Delta/Alpha-Delta_mean-win_AF4.pdf]

**Alpha/Delta ratio on average**  
**PSD windows for electrode: Avg AF3-F3-F7**

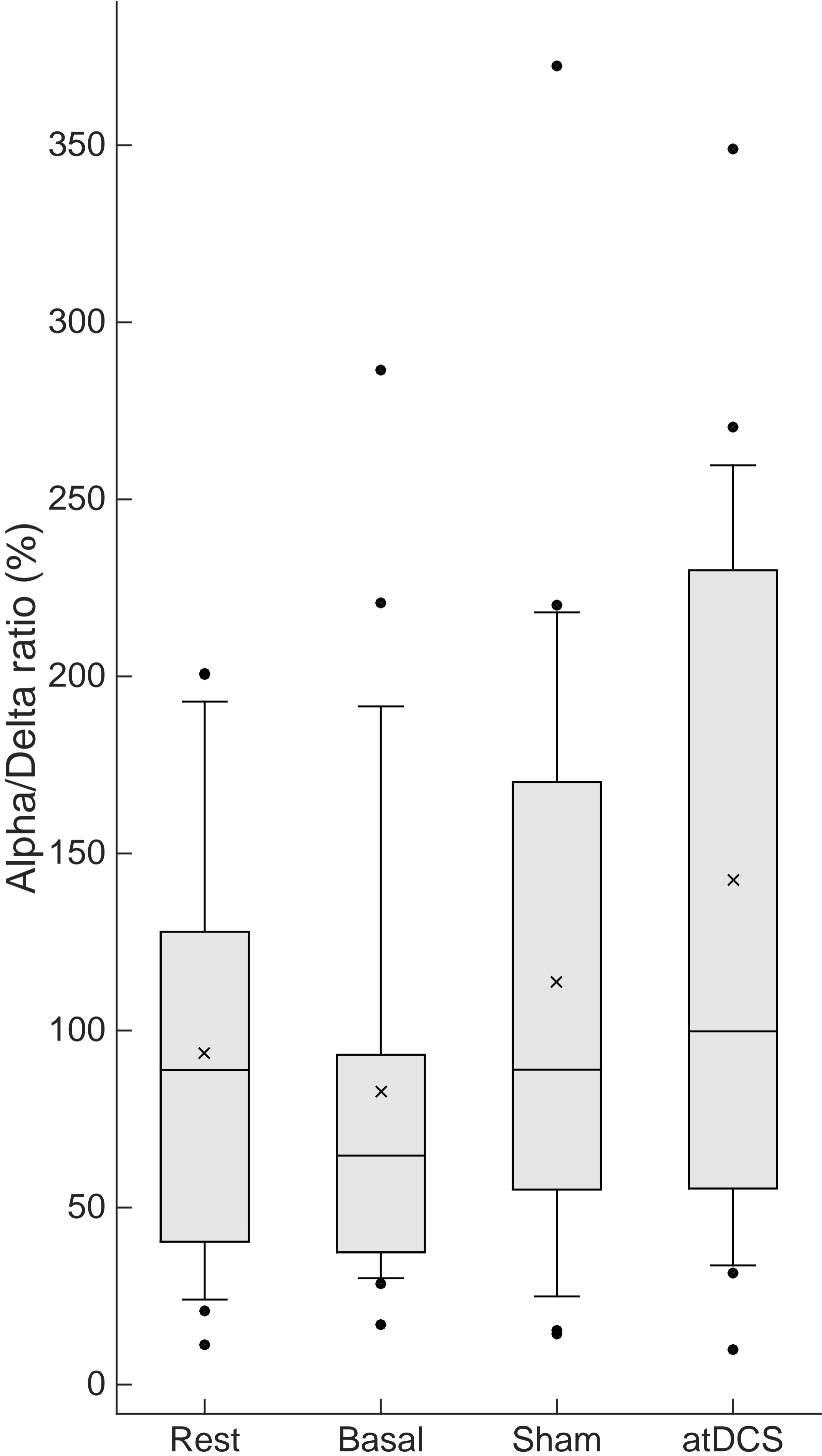

Supplement: Supplementary file 1 [file Data_Sheet_1.zip › Complementary_results/Band_ratios_average_PSD_windows/Alpha_Delta/Alpha-Delta_mean-win_Avg AF3-F3-F7.pdf]

Alpha/Delta ratio on average  
PSD windows for electrode: Avg AF4-F4-F8

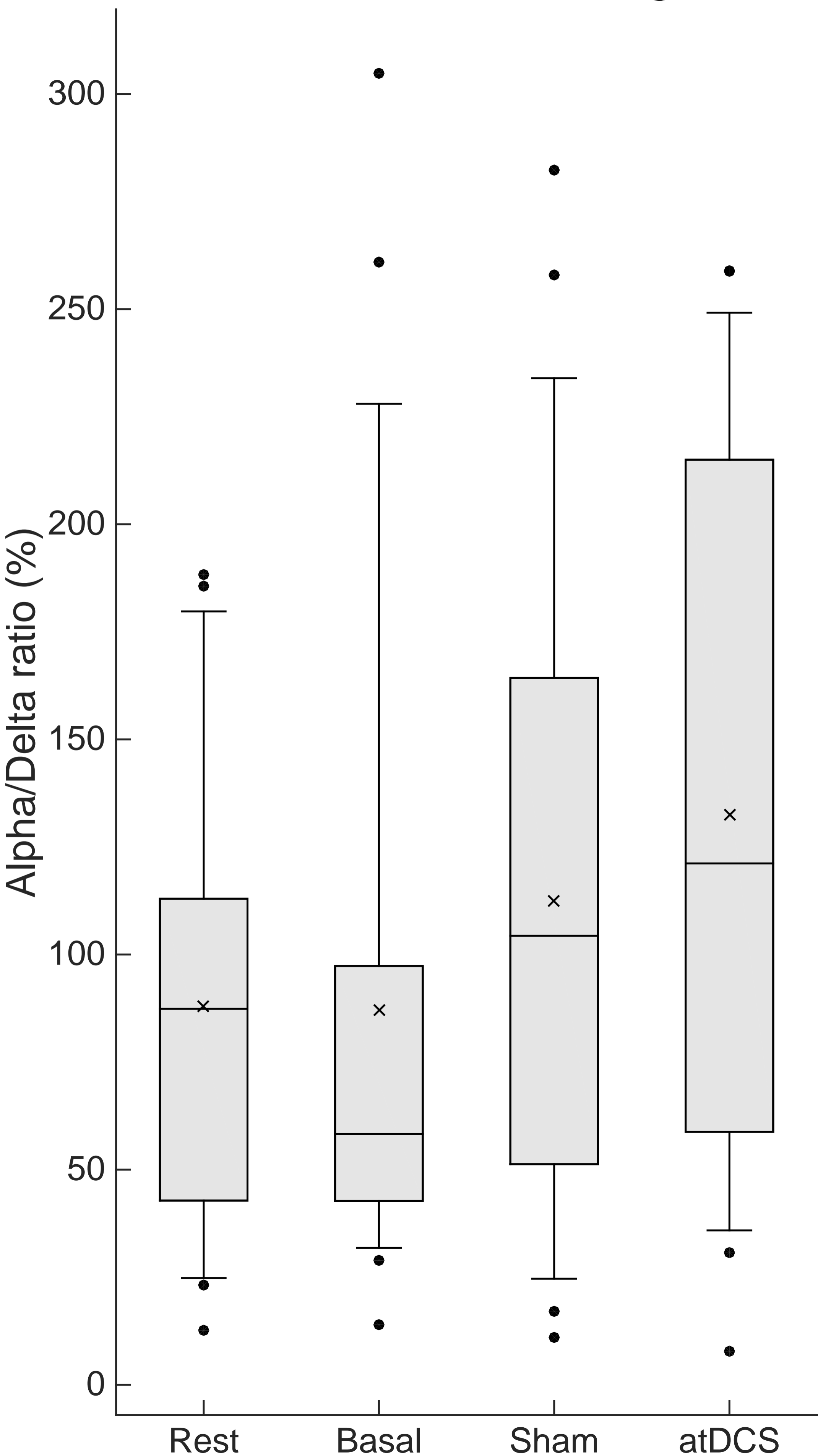

Supplement: Supplementary file 1 [file Data_Sheet_1.zip › Complementary_results/Band_ratios_average_PSD_windows/Alpha_Delta/Alpha-Delta_mean-win_Avg AF4-F4-F8.pdf]

**Alpha/Delta ratio on average**  
**PSD windows for electrode: Avg F3-F7-FC5**

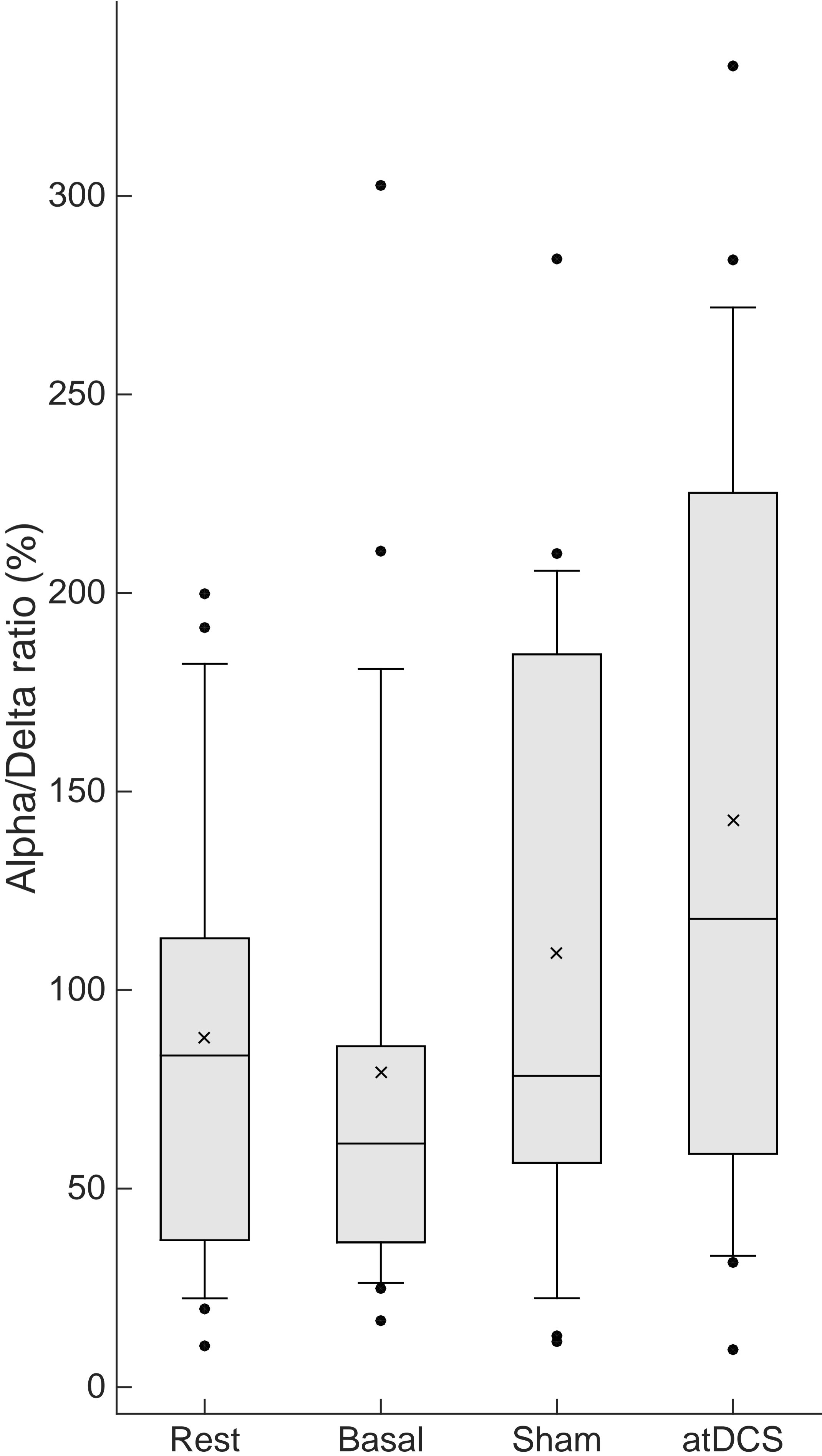

Supplement: Supplementary file 1 [file Data_Sheet_1.zip › Complementary_results/Band_ratios_average_PSD_windows/Alpha_Delta/Alpha-Delta_mean-win_Avg F3-F7-FC5.pdf]

**Alpha/Delta ratio on average**  
**PSD windows for electrode: Avg F4-F8-FC6**

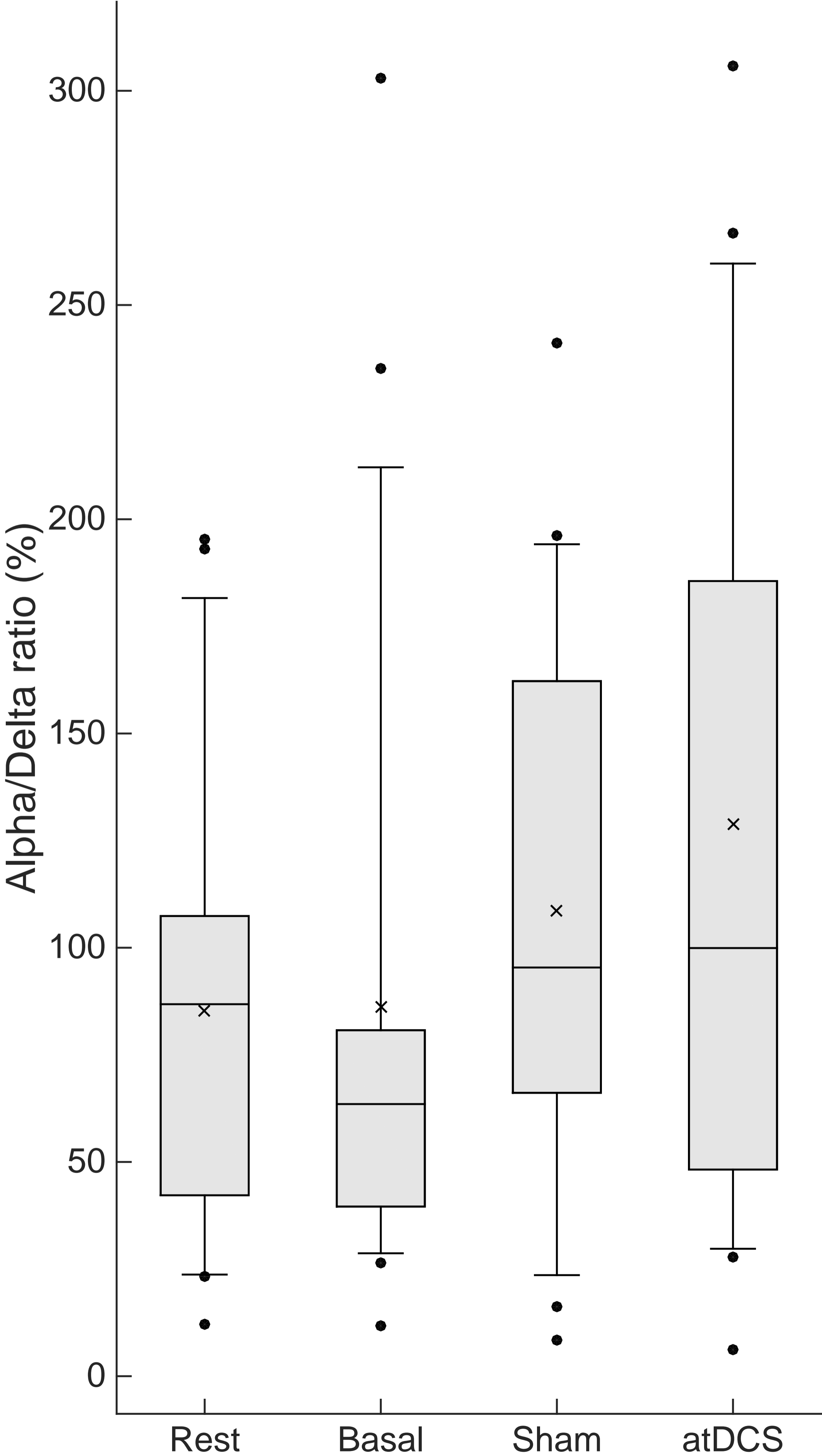

Supplement: Supplementary file 1 [file Data_Sheet_1.zip › Complementary_results/Band_ratios_average_PSD_windows/Alpha_Delta/Alpha-Delta_mean-win_Avg F4-F8-FC6.pdf]

**Alpha/Delta ratio on average  
PSD windows for electrode: F3**

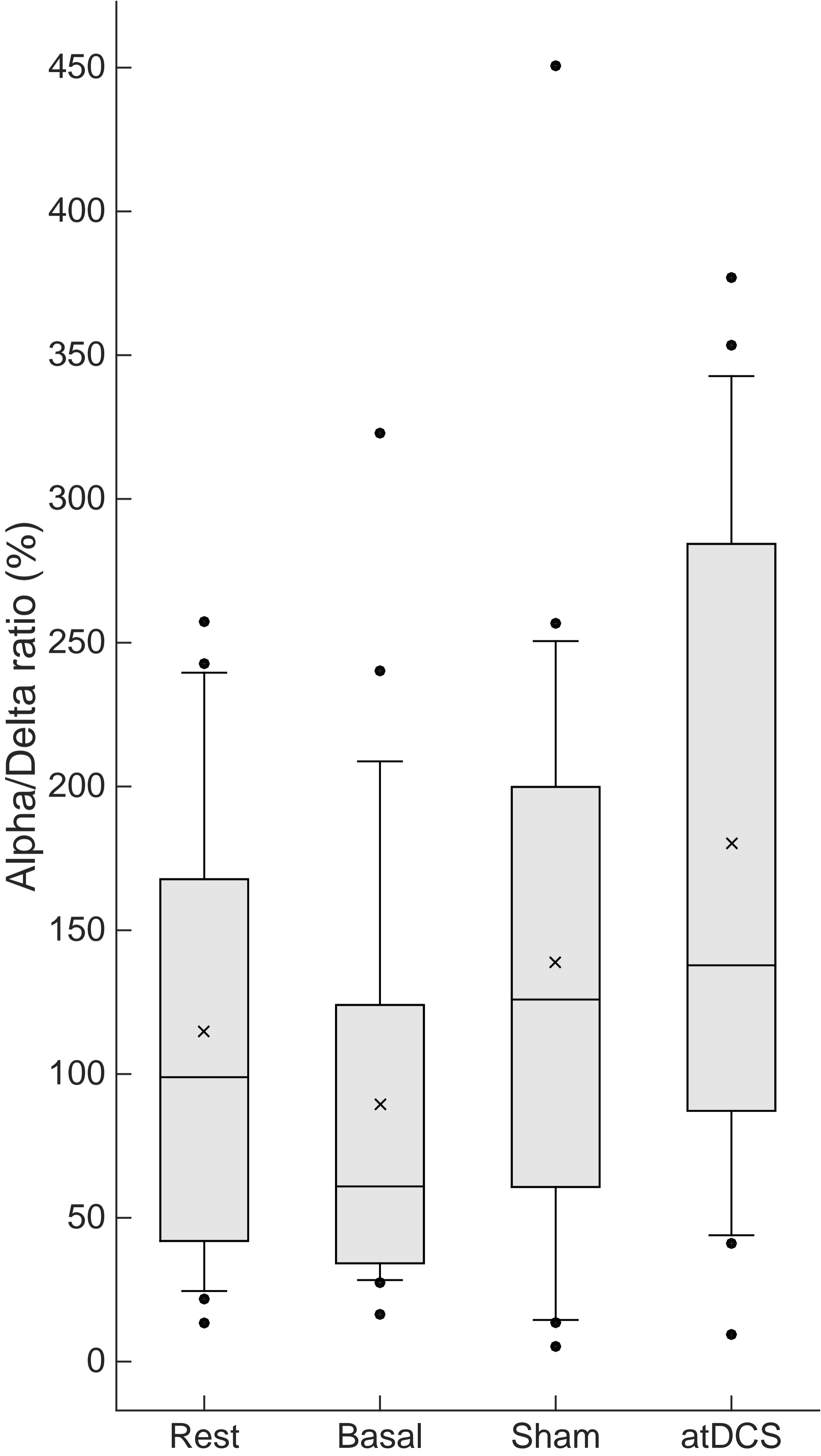

Supplement: Supplementary file 1 [file Data_Sheet_1.zip › Complementary_results/Band_ratios_average_PSD_windows/Alpha_Delta/Alpha-Delta_mean-win_F3.pdf]

**Alpha/Delta ratio on average  
PSD windows for electrode: F4**

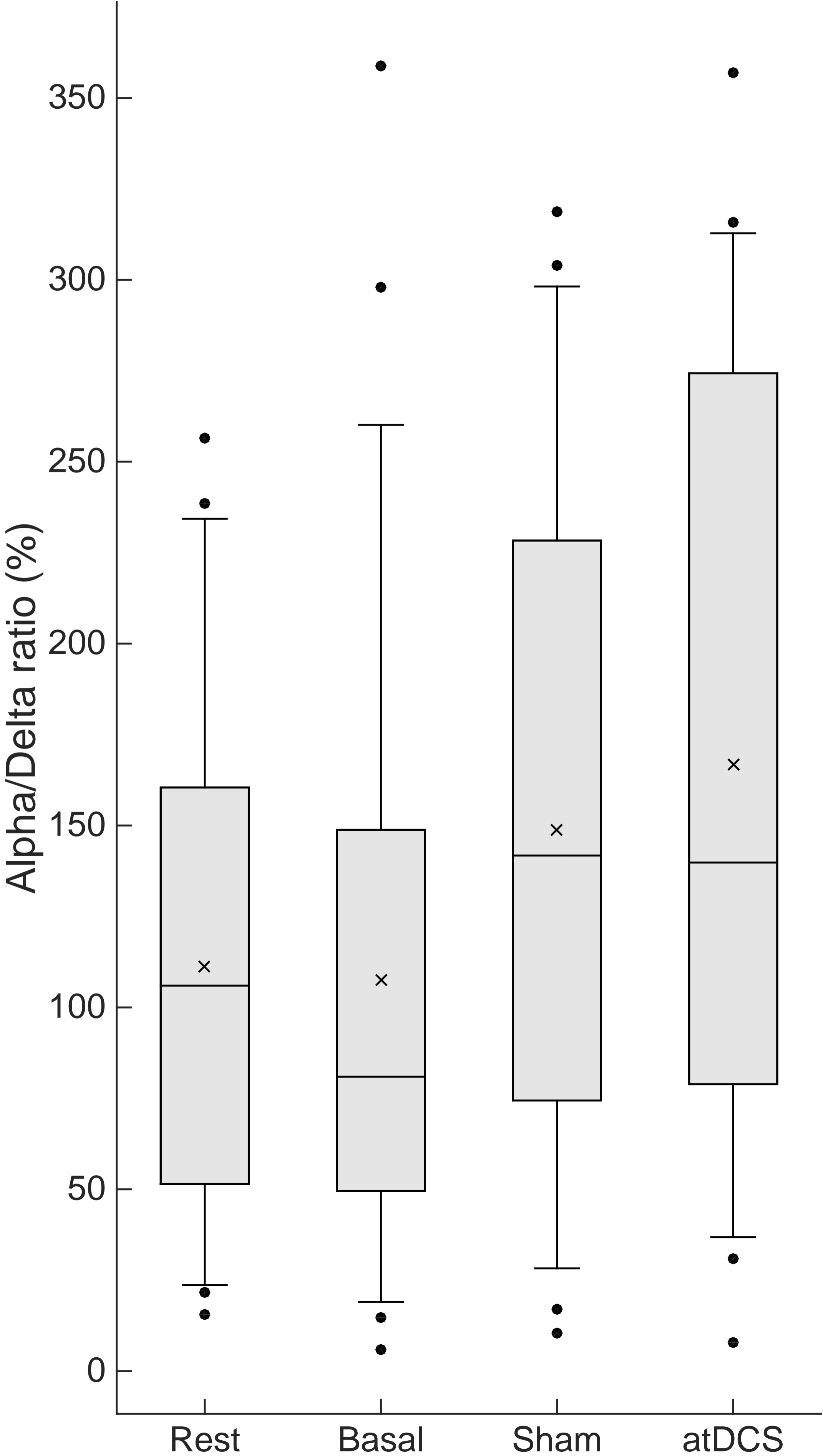

Supplement: Supplementary file 1 [file Data_Sheet_1.zip › Complementary_results/Band_ratios_average_PSD_windows/Alpha_Delta/Alpha-Delta_mean-win_F4.pdf]

**Alpha/Delta ratio on average  
PSD windows for electrode: F7**

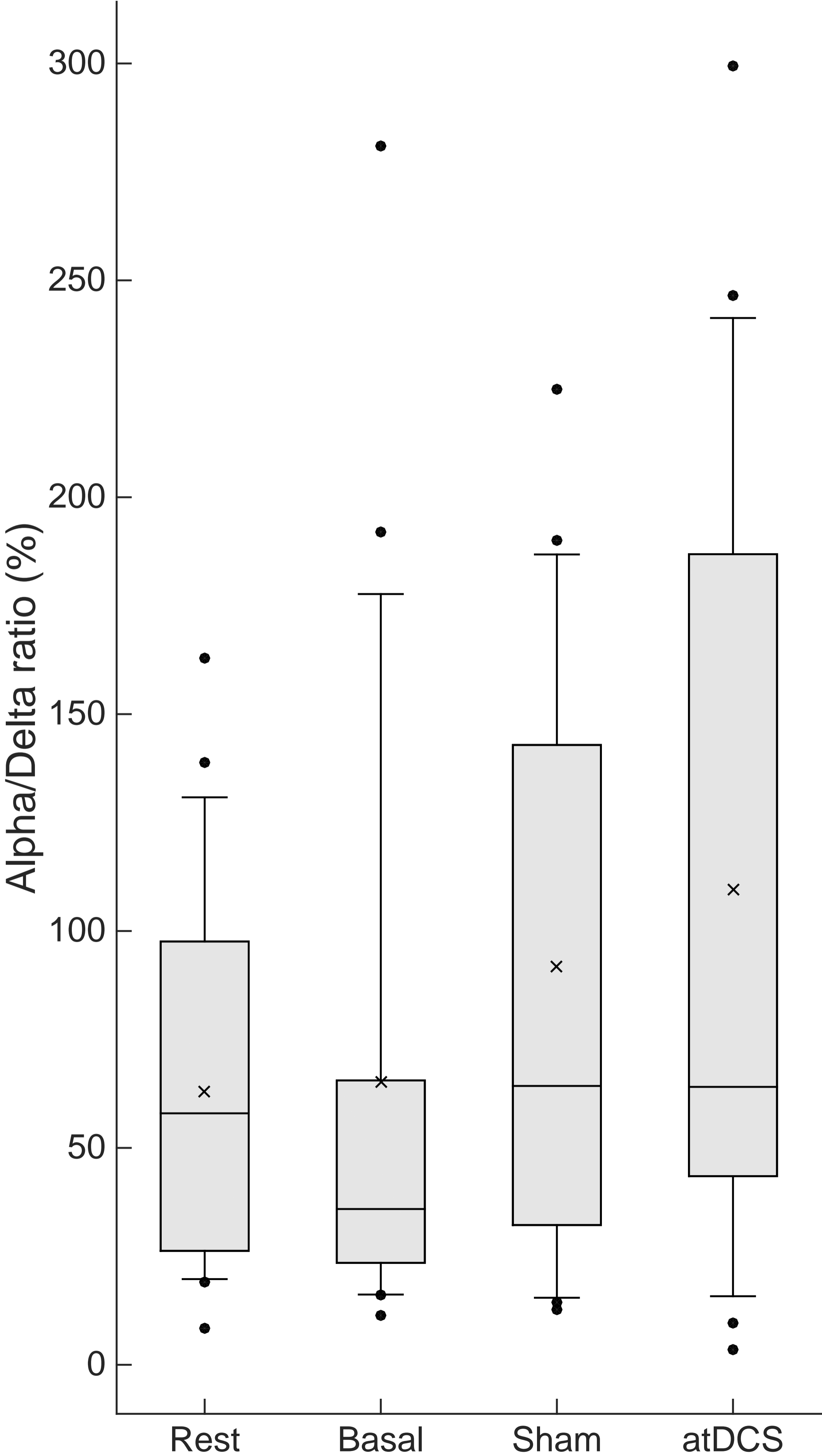

Supplement: Supplementary file 1 [file Data_Sheet_1.zip › Complementary_results/Band_ratios_average_PSD_windows/Alpha_Delta/Alpha-Delta_mean-win_F7.pdf]

**Alpha/Delta ratio on average  
PSD windows for electrode: F8**

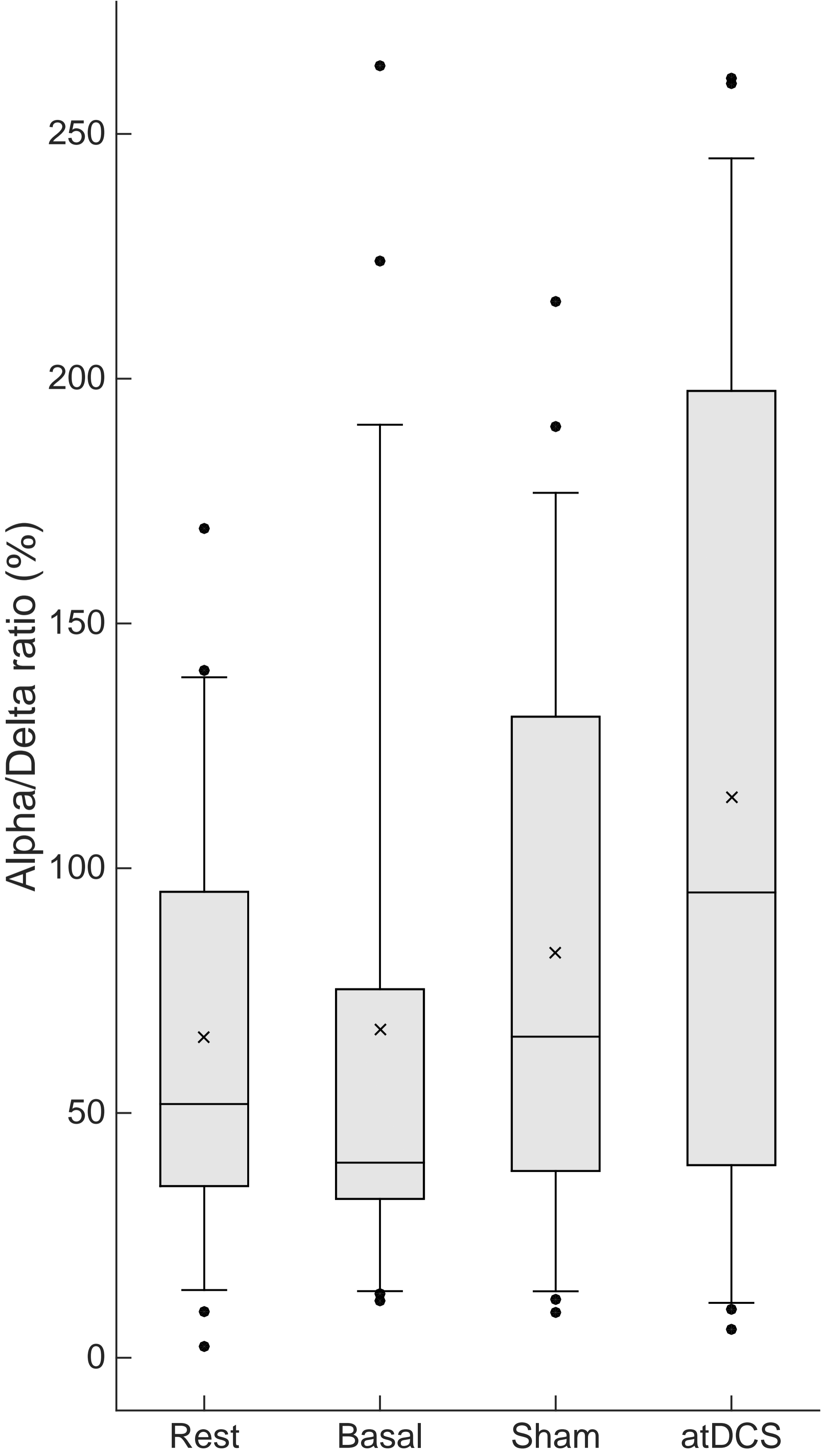

Supplement: Supplementary file 1 [file Data_Sheet_1.zip › Complementary_results/Band_ratios_average_PSD_windows/Alpha_Delta/Alpha-Delta_mean-win_F8.pdf]

**Alpha/Delta ratio on average  
PSD windows for electrode: FC5**

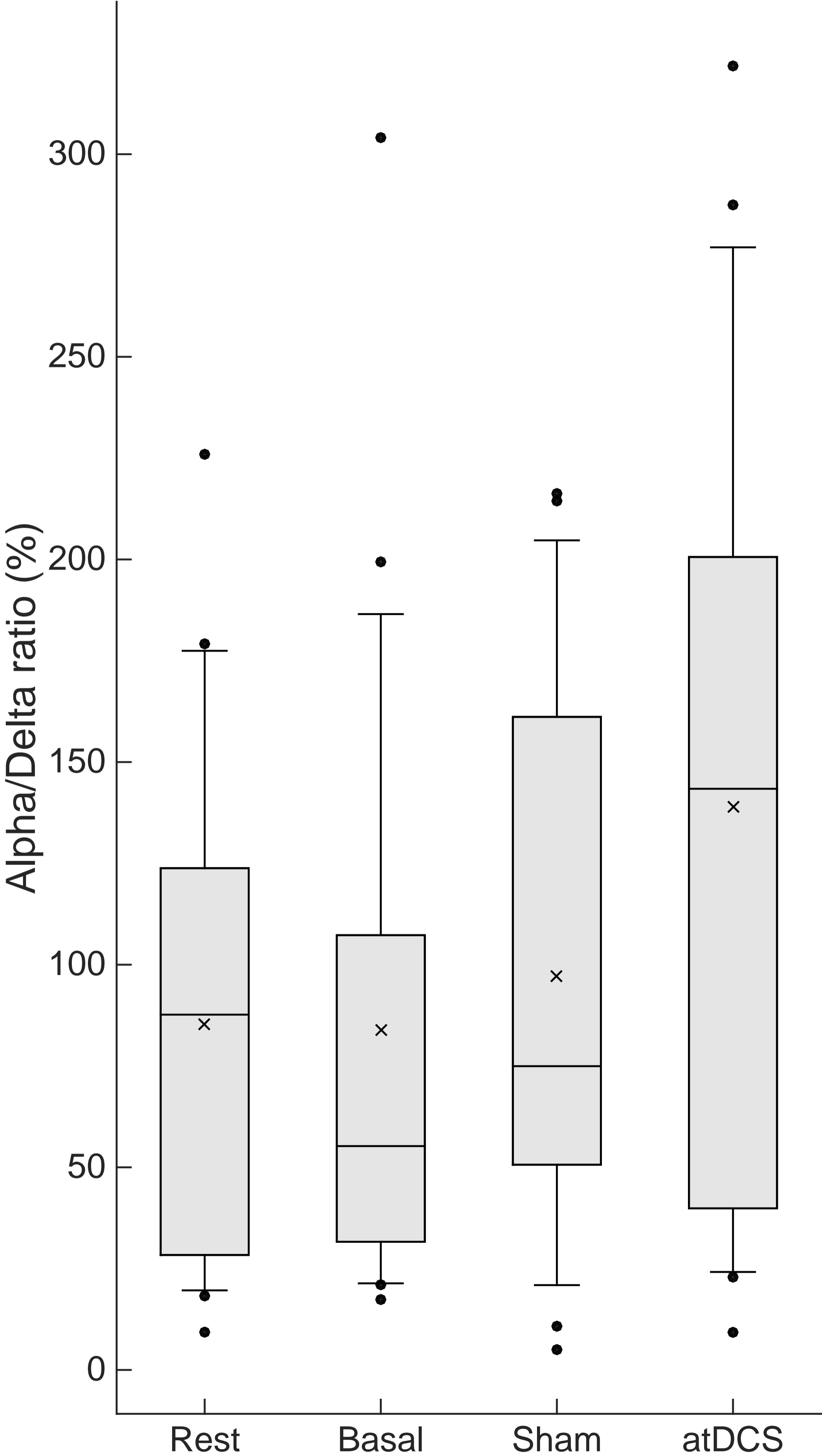

Supplement: Supplementary file 1 [file Data_Sheet_1.zip › Complementary_results/Band_ratios_average_PSD_windows/Alpha_Delta/Alpha-Delta_mean-win_FC5.pdf]

**Alpha/Delta ratio on average  
PSD windows for electrode: FC6**

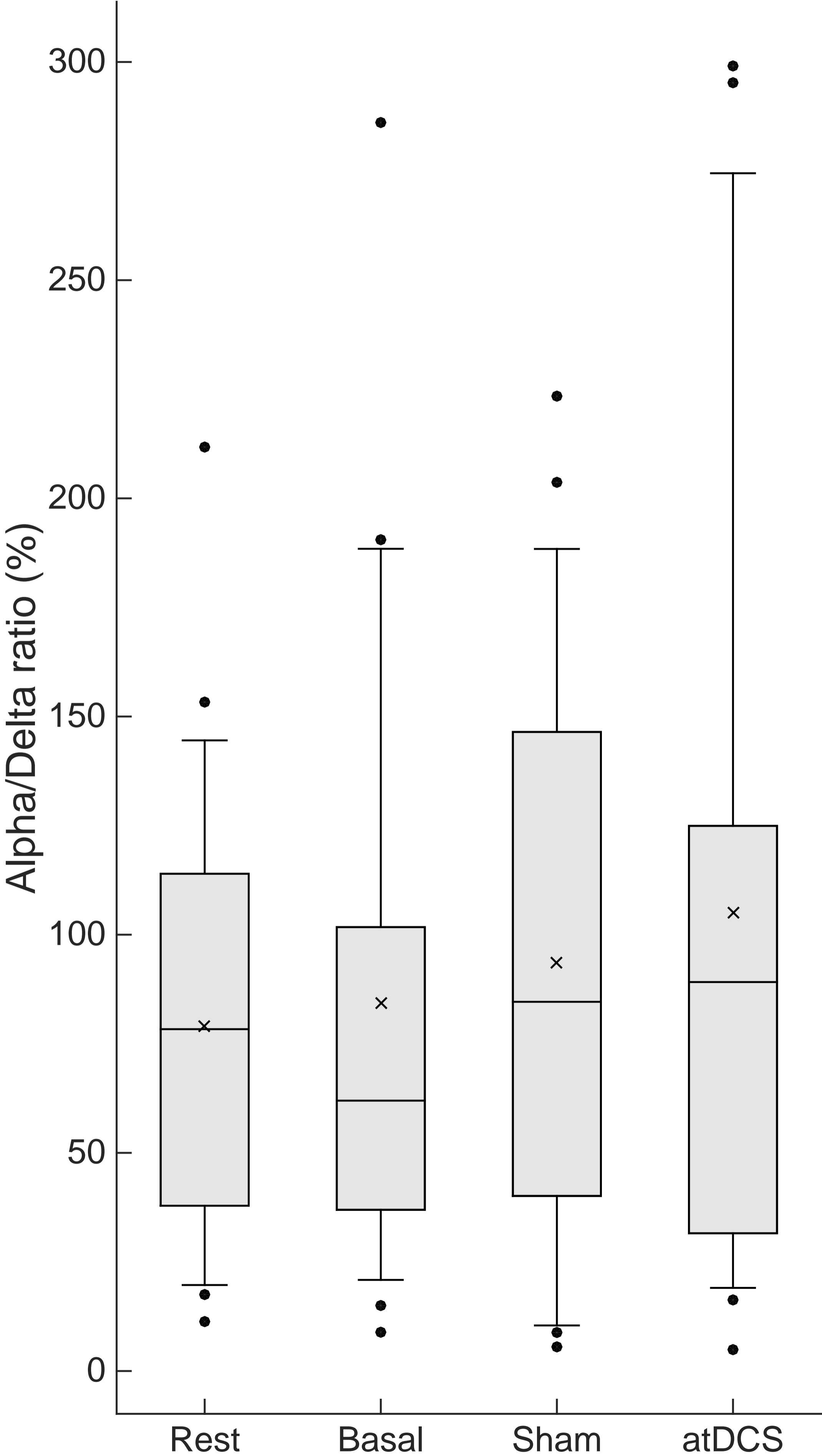

Supplement: Supplementary file 1 [file Data_Sheet_1.zip › Complementary_results/Band_ratios_average_PSD_windows/Alpha_Delta/Alpha-Delta_mean-win_FC6.pdf]

# Alpha/Delta ratio on average PSD windows for electrode: O1

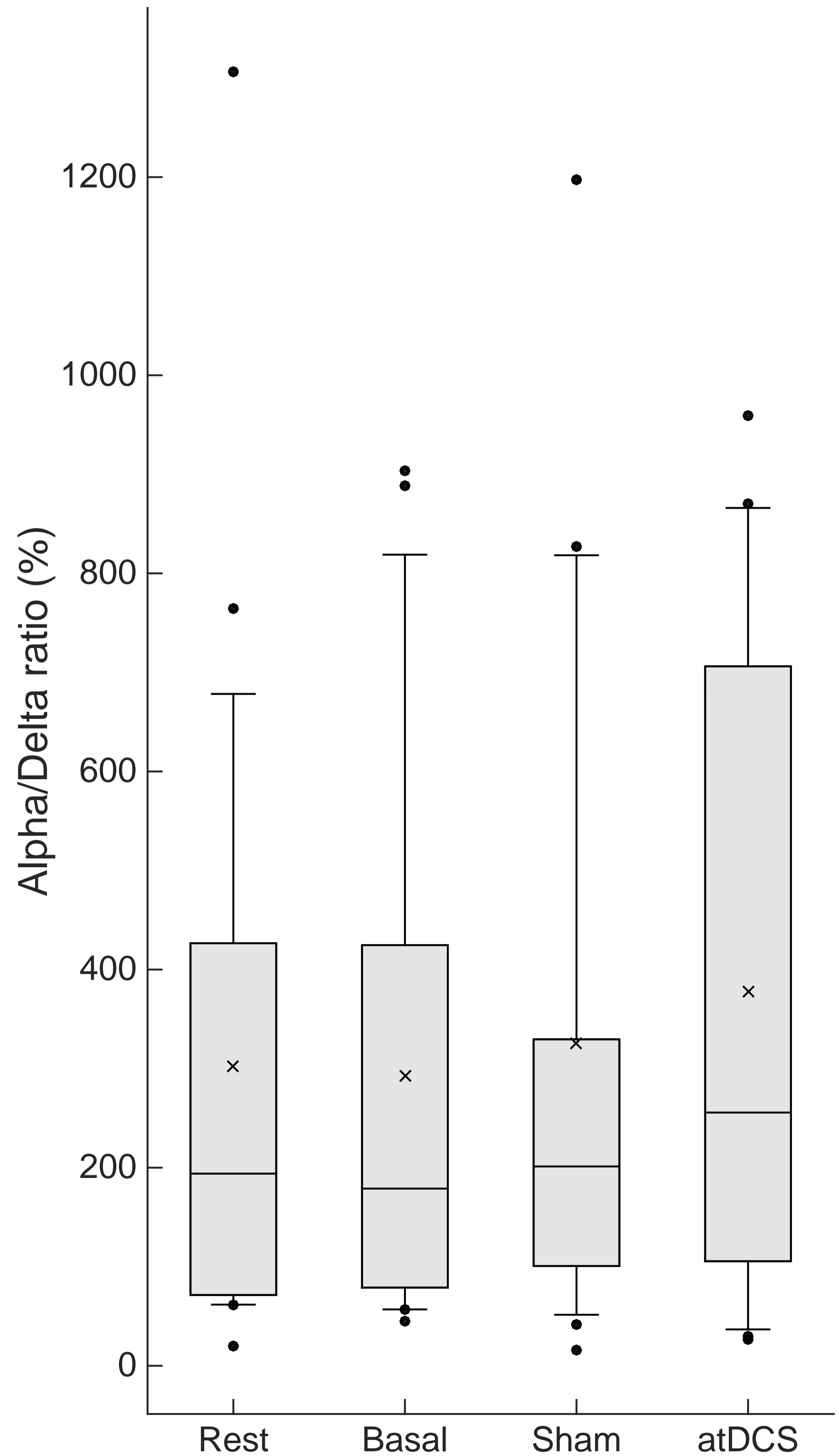

Supplement: Supplementary file 1 [file Data_Sheet_1.zip › Complementary_results/Band_ratios_average_PSD_windows/Alpha_Delta/Alpha-Delta_mean-win_O1.pdf]

# Alpha/Delta ratio on average PSD windows for electrode: O2

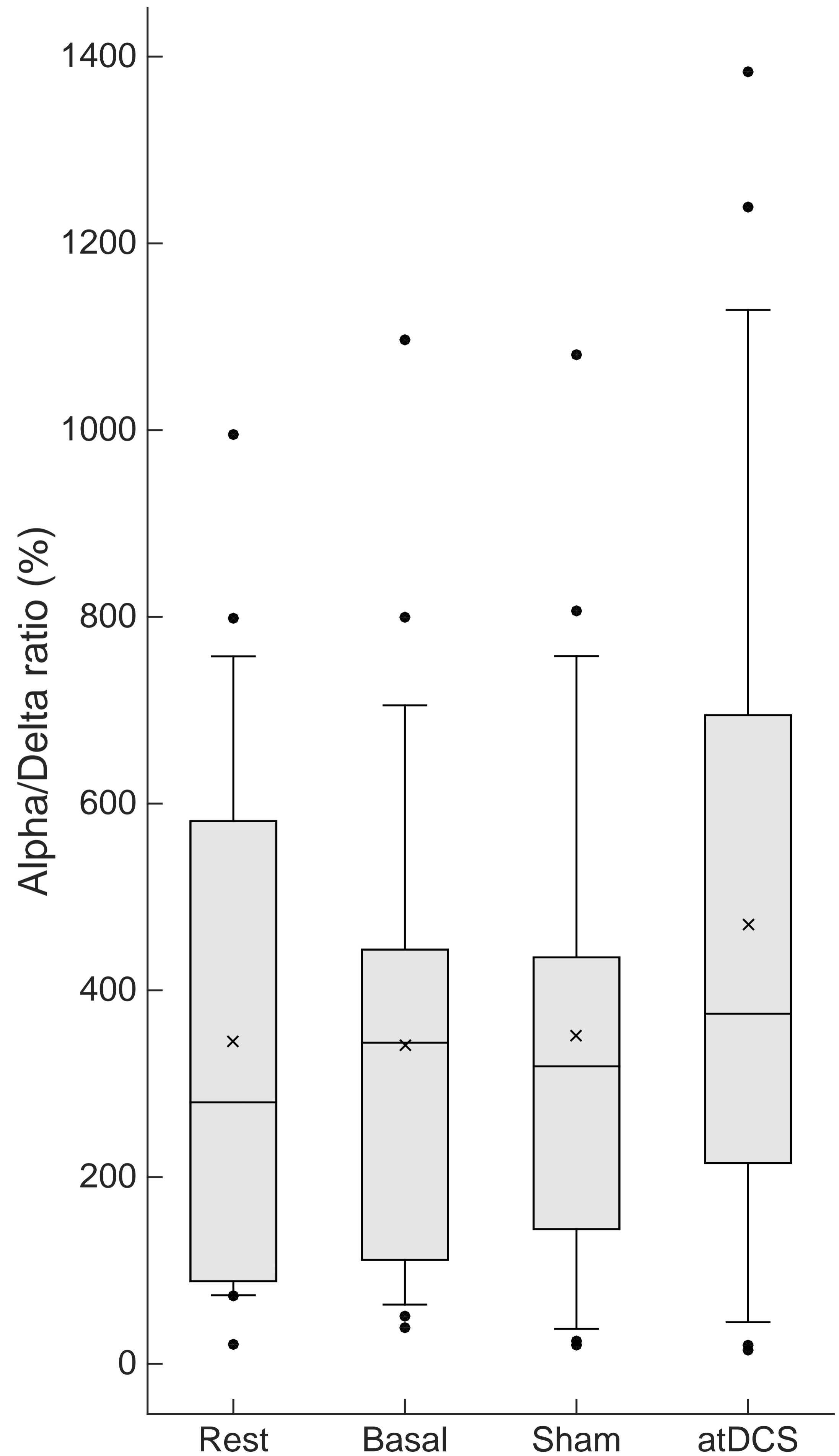

Supplement: Supplementary file 1 [file Data_Sheet_1.zip › Complementary_results/Band_ratios_average_PSD_windows/Alpha_Delta/Alpha-Delta_mean-win_O2.pdf]

**Alpha/Delta ratio on average  
PSD windows for electrode: P7**

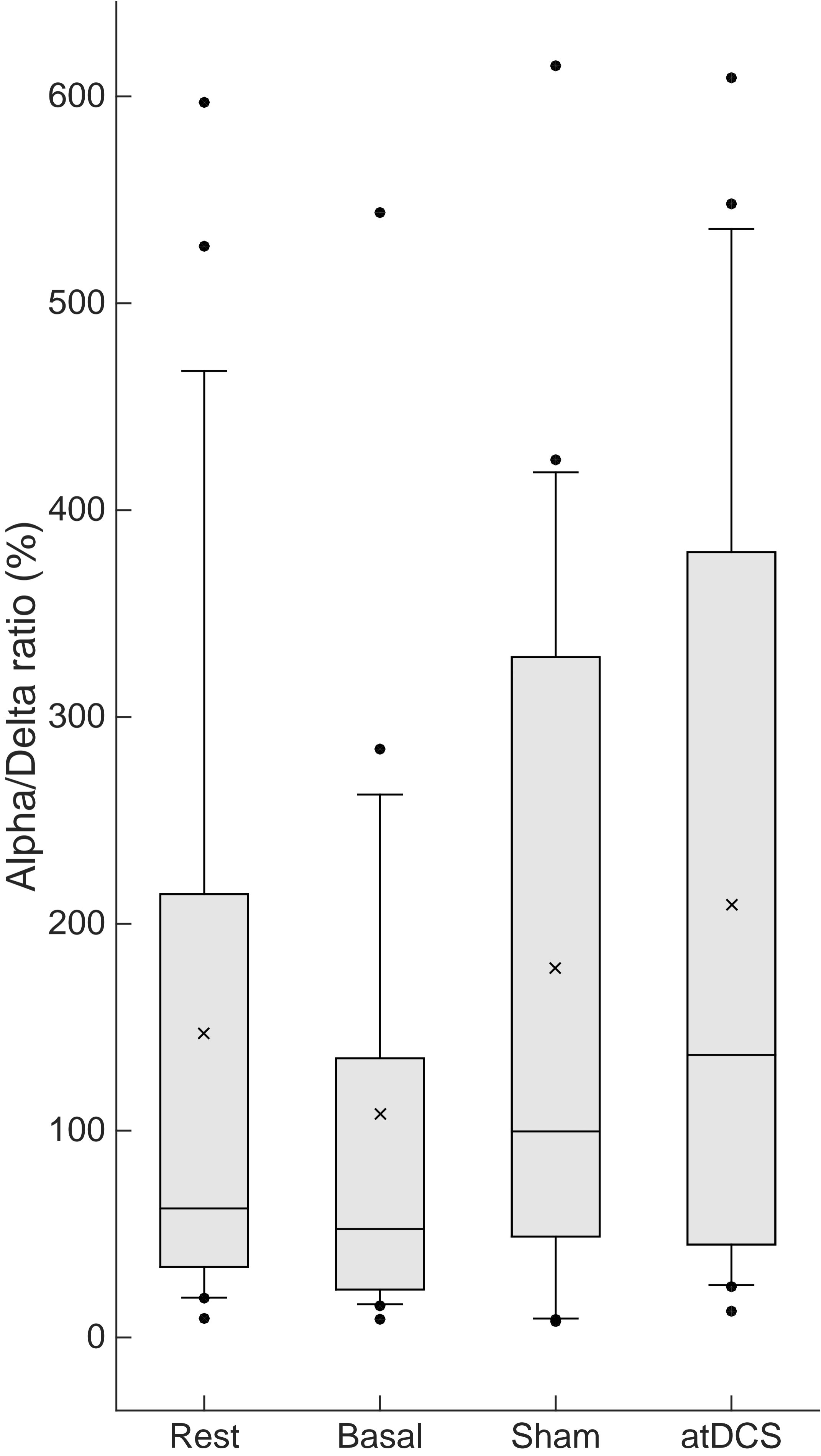

Supplement: Supplementary file 1 [file Data_Sheet_1.zip › Complementary_results/Band_ratios_average_PSD_windows/Alpha_Delta/Alpha-Delta_mean-win_P7.pdf]

**Alpha/Delta ratio on average  
PSD windows for electrode: P8**

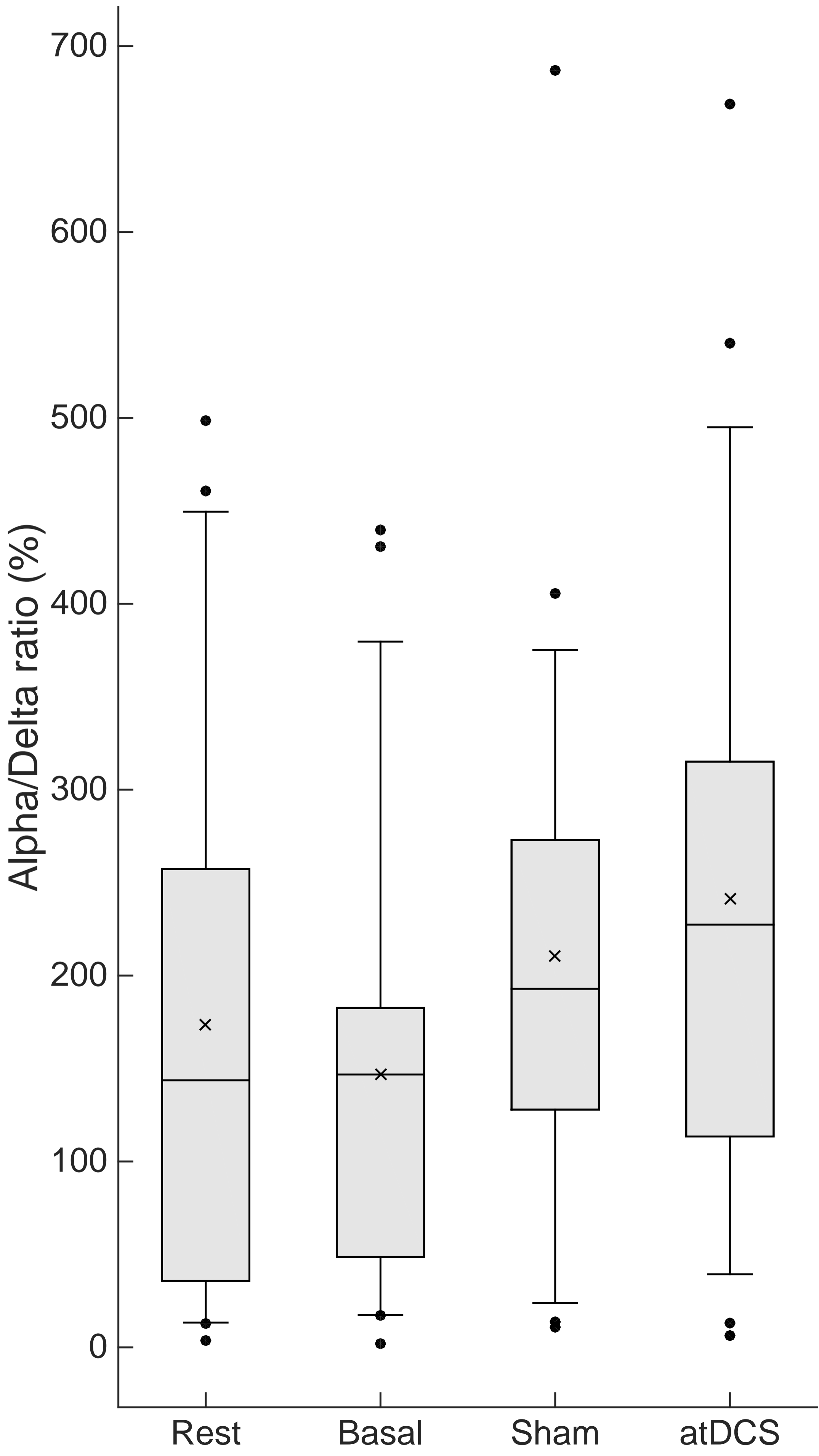

Supplement: Supplementary file 1 [file Data_Sheet_1.zip › Complementary_results/Band_ratios_average_PSD_windows/Alpha_Delta/Alpha-Delta_mean-win_P8.pdf]

**Alpha/Delta ratio on average  
PSD windows for electrode: T7**

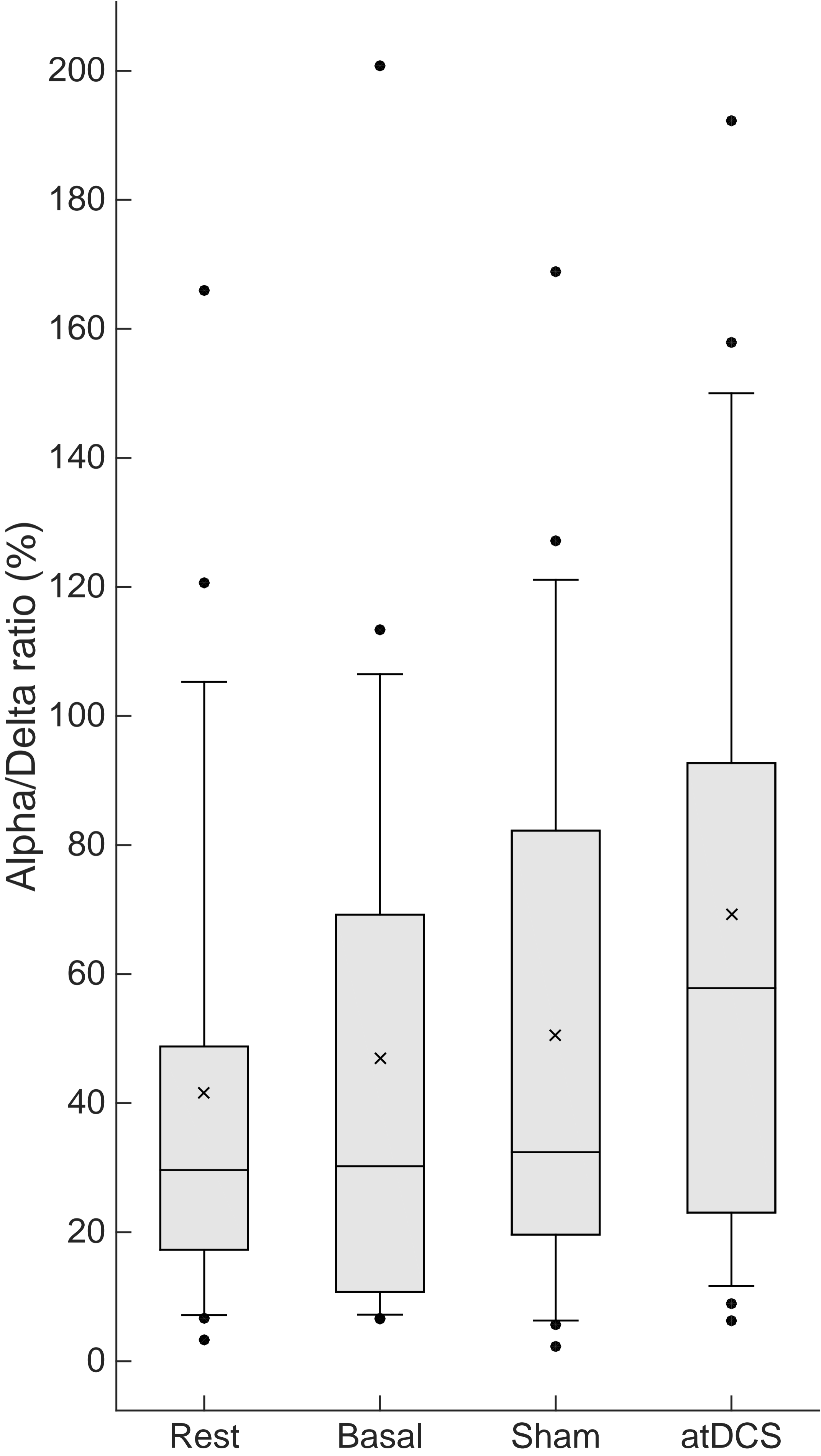

Supplement: Supplementary file 1 [file Data_Sheet_1.zip › Complementary_results/Band_ratios_average_PSD_windows/Alpha_Delta/Alpha-Delta_mean-win_T7.pdf]

**Alpha/Delta ratio on average  
PSD windows for electrode: T8**

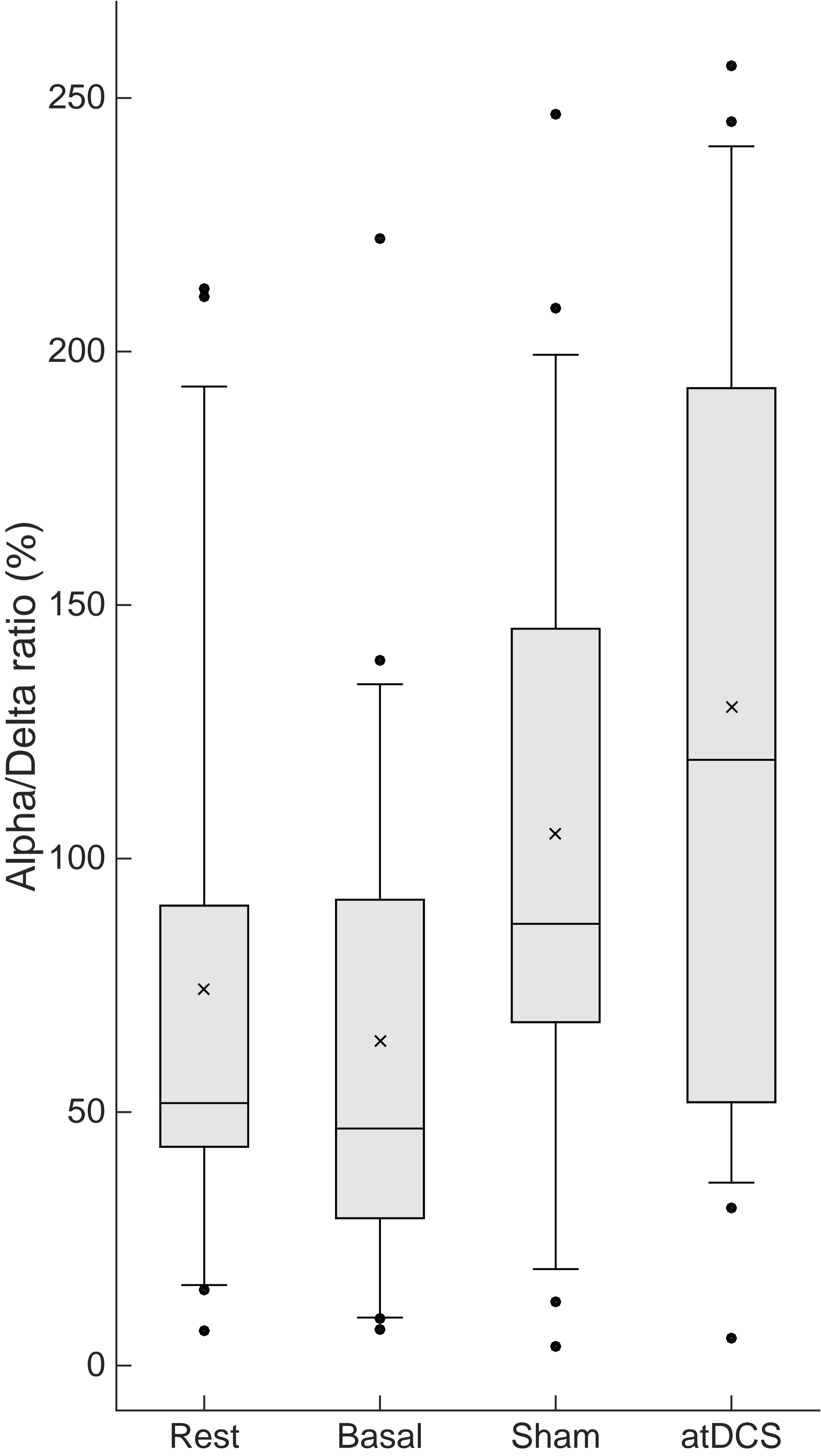

Supplement: Supplementary file 1 [file Data_Sheet_1.zip › Complementary_results/Band_ratios_average_PSD_windows/Alpha_Delta/Alpha-Delta_mean-win_T8.pdf]

**Beta<sup>2</sup>/Alpha ratio on average  
PSD windows for electrode: AF3**

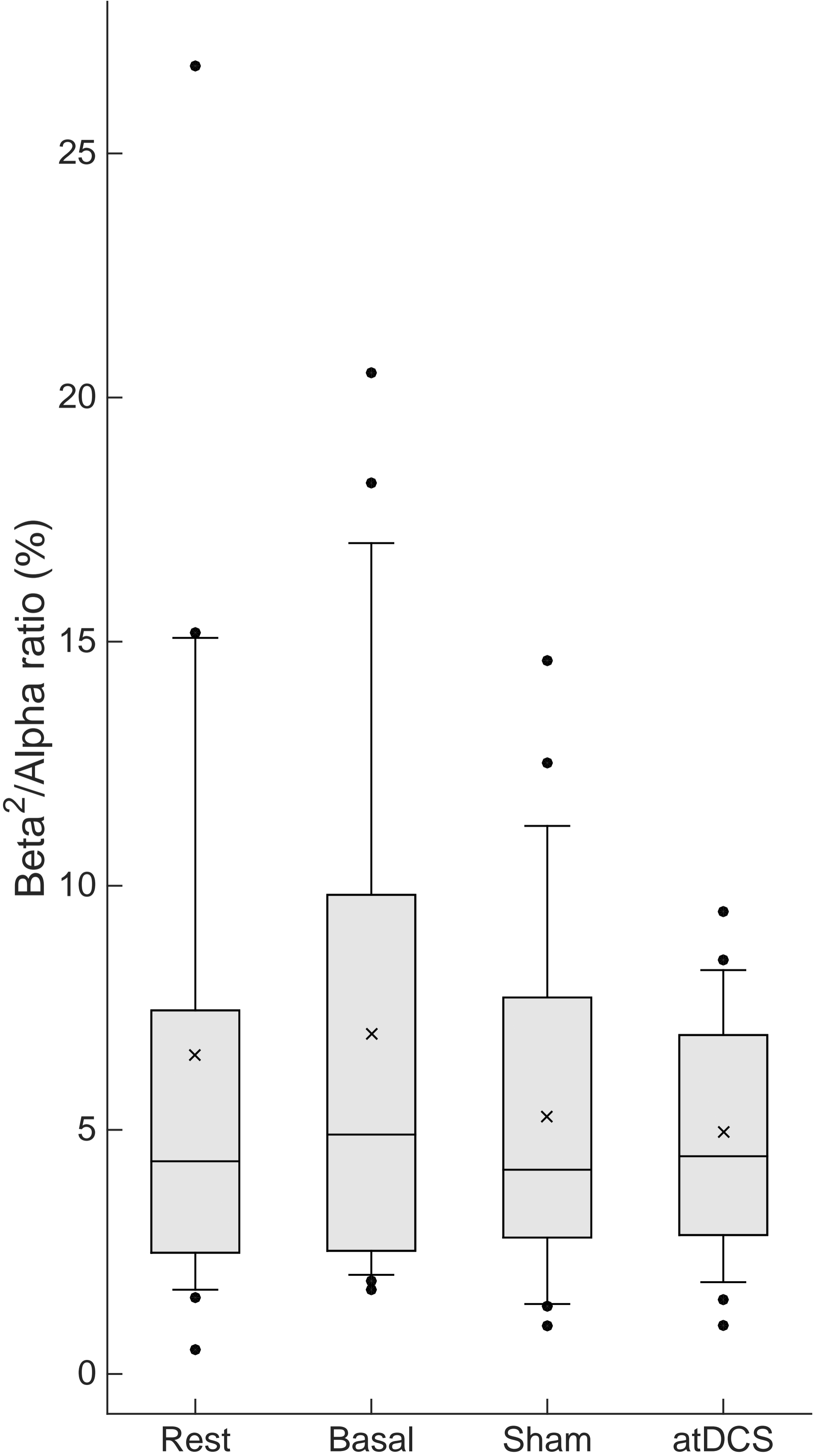

Supplement: Supplementary file 1 [file Data_Sheet_1.zip › Complementary_results/Band_ratios_average_PSD_windows/Beta^2-Alpha/Beta^2-Alpha_mean-win_AF3.pdf]

**Beta<sup>2</sup>/Alpha ratio on average  
PSD windows for electrode: AF4**

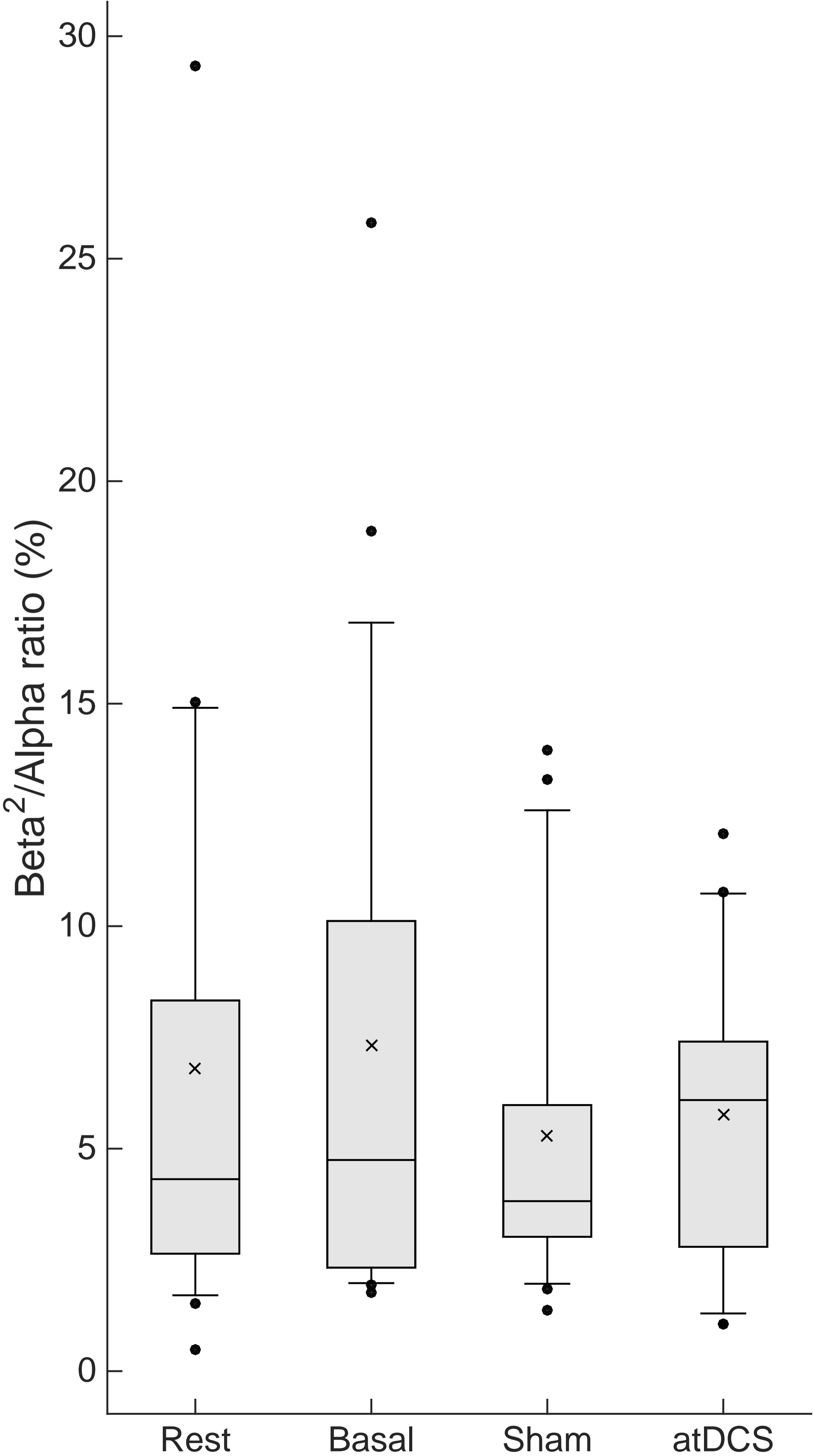

Supplement: Supplementary file 1 [file Data_Sheet_1.zip › Complementary_results/Band_ratios_average_PSD_windows/Beta^2-Alpha/Beta^2-Alpha_mean-win_AF4.pdf]

**Beta<sup>2</sup>/Alpha ratio on average**  
**PSD windows for electrode: Avg AF3-F3-F7**

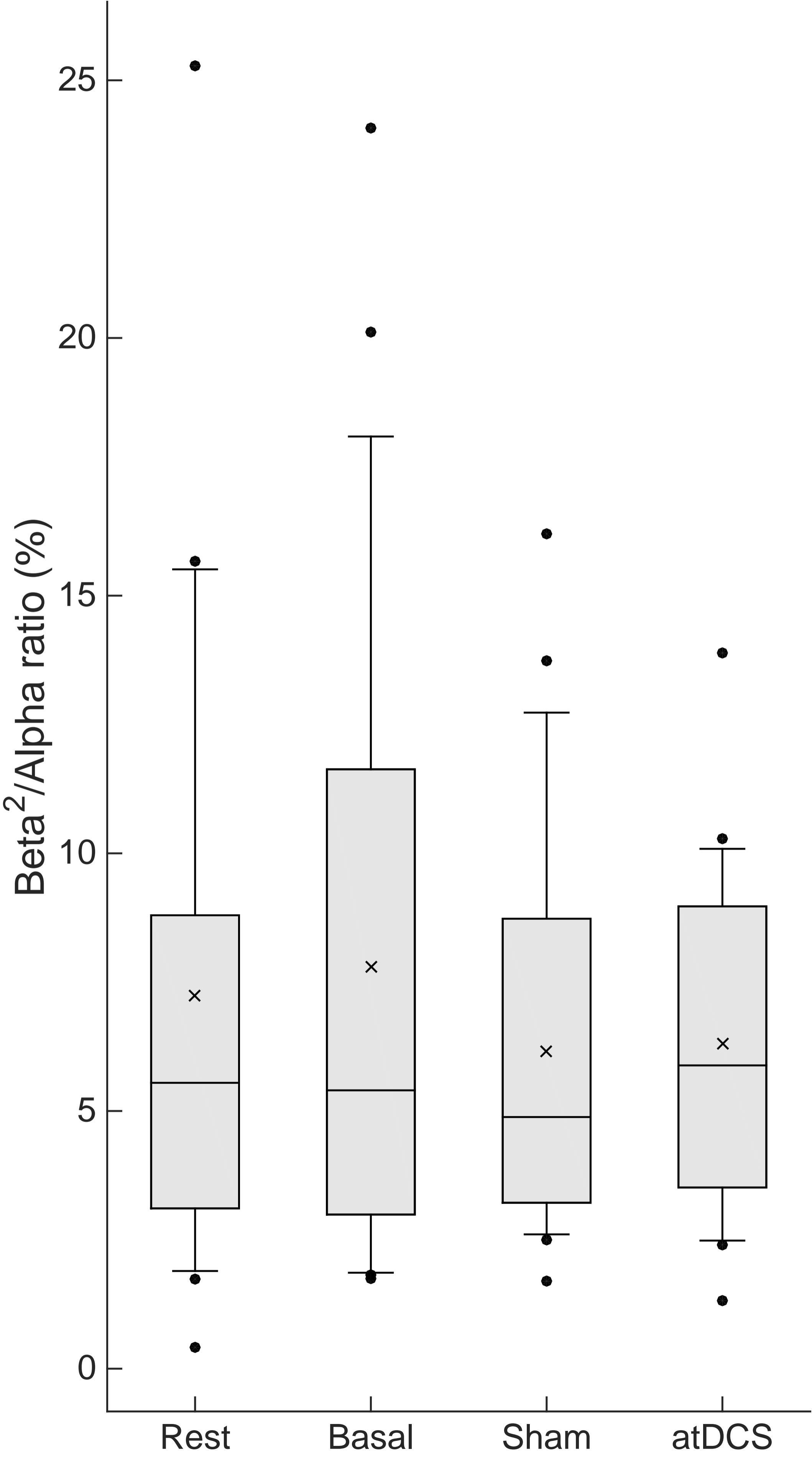

Supplement: Supplementary file 1 [file Data_Sheet_1.zip › Complementary_results/Band_ratios_average_PSD_windows/Beta^2-Alpha/Beta^2-Alpha_mean-win_Avg AF3-F3-F7.pdf]

**Beta<sup>2</sup>/Alpha ratio on average**  
**PSD windows for electrode: Avg AF4-F4-F8**

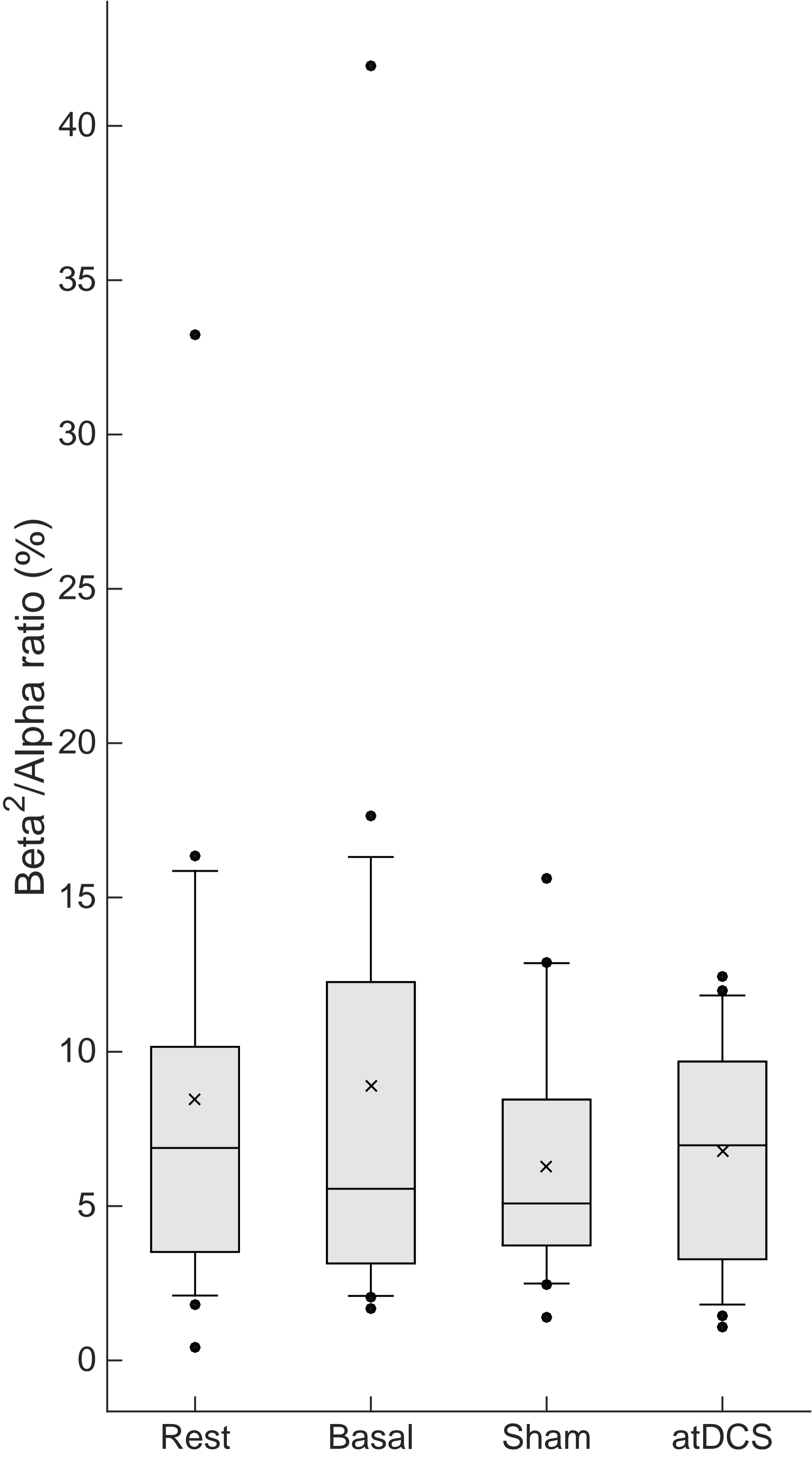

Supplement: Supplementary file 1 [file Data_Sheet_1.zip › Complementary_results/Band_ratios_average_PSD_windows/Beta^2-Alpha/Beta^2-Alpha_mean-win_Avg AF4-F4-F8.pdf]

**Beta<sup>2</sup>/Alpha ratio on average**  
**PSD windows for electrode: Avg F3-F7-FC5**

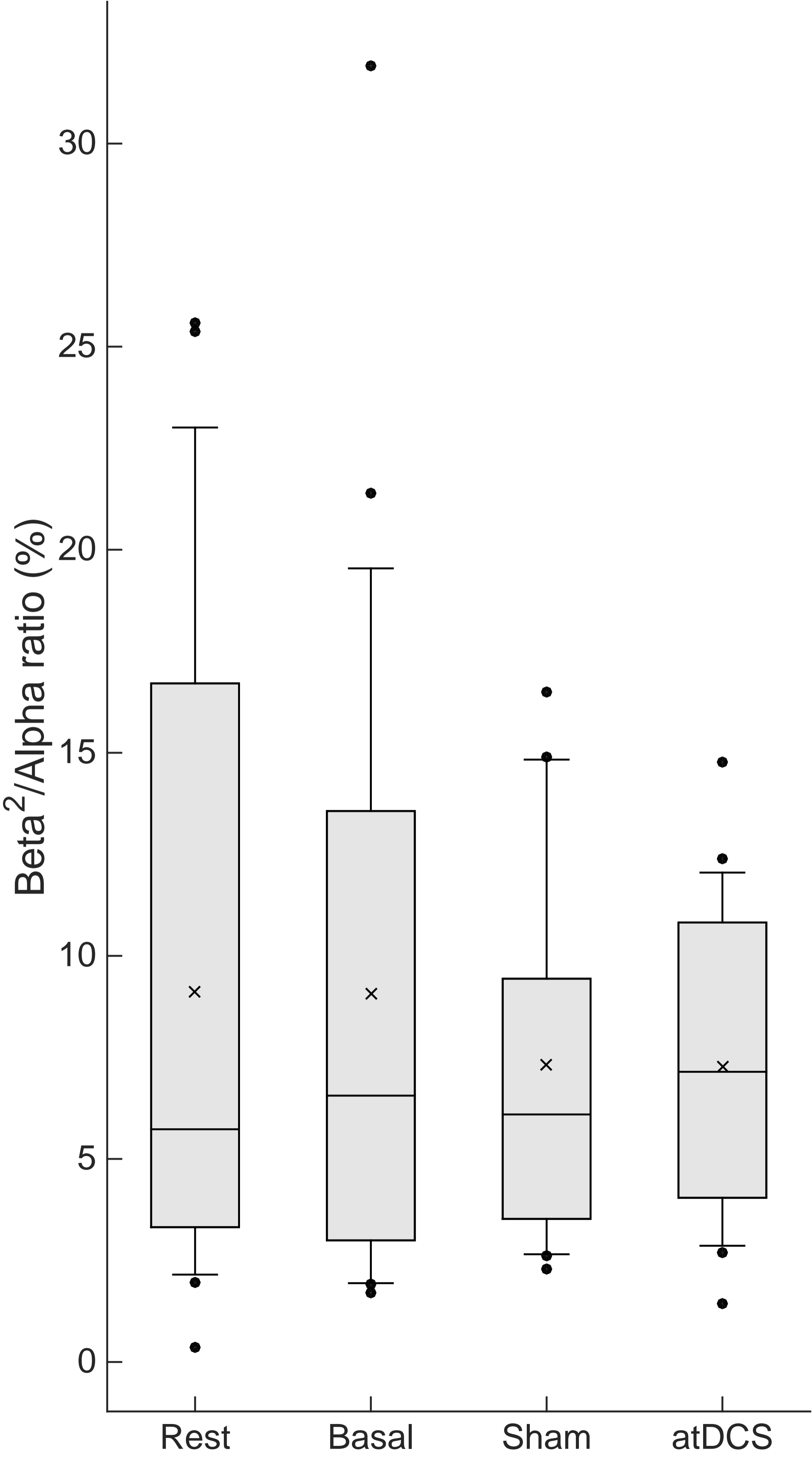

Supplement: Supplementary file 1 [file Data_Sheet_1.zip › Complementary_results/Band_ratios_average_PSD_windows/Beta^2-Alpha/Beta^2-Alpha_mean-win_Avg F3-F7-FC5.pdf]

**Beta<sup>2</sup>/Alpha ratio on average**  
**PSD windows for electrode: Avg F4-F8-FC6**

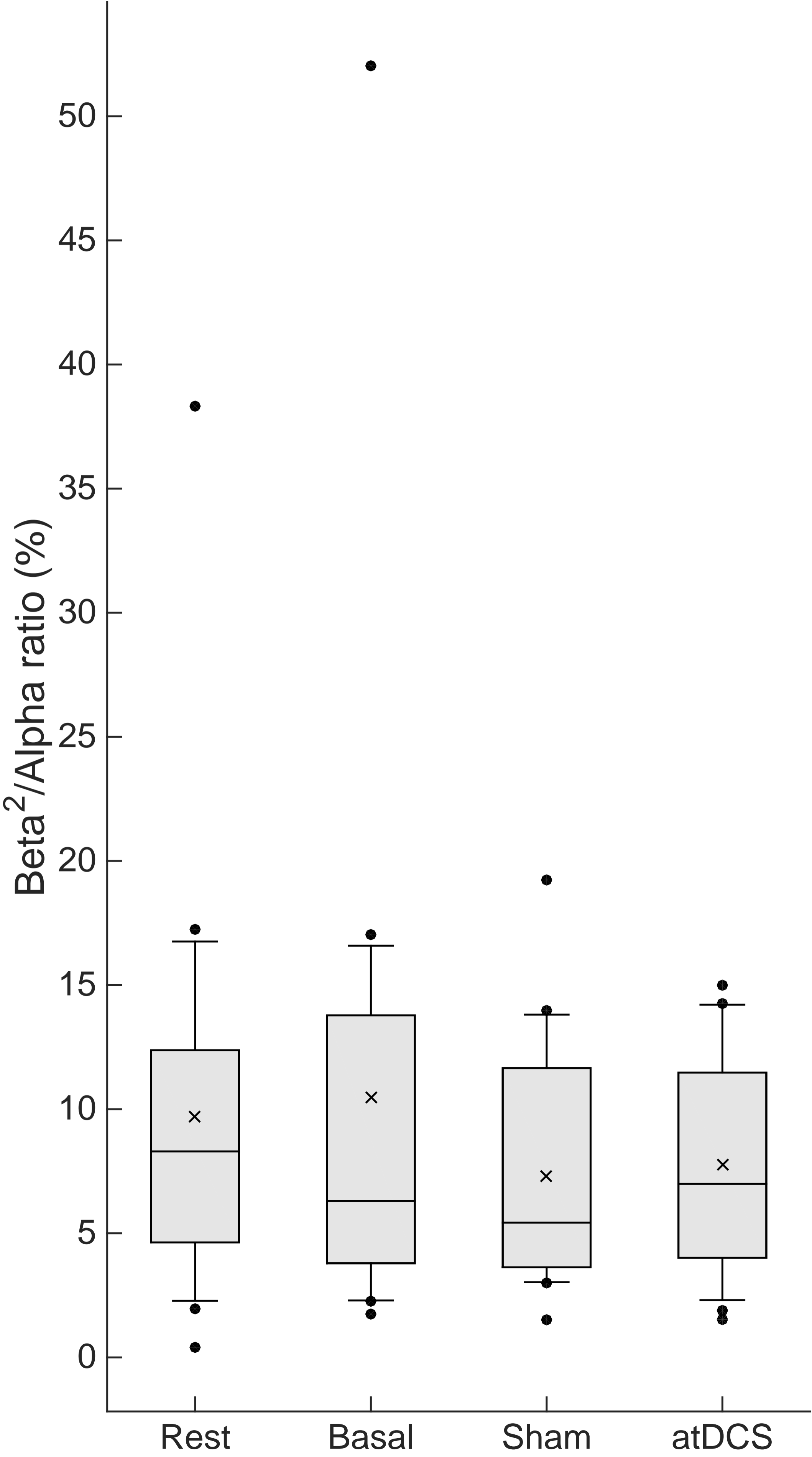

Supplement: Supplementary file 1 [file Data_Sheet_1.zip › Complementary_results/Band_ratios_average_PSD_windows/Beta^2-Alpha/Beta^2-Alpha_mean-win_Avg F4-F8-FC6.pdf]

**Beta<sup>2</sup>/Alpha ratio on average  
PSD windows for electrode: F3**

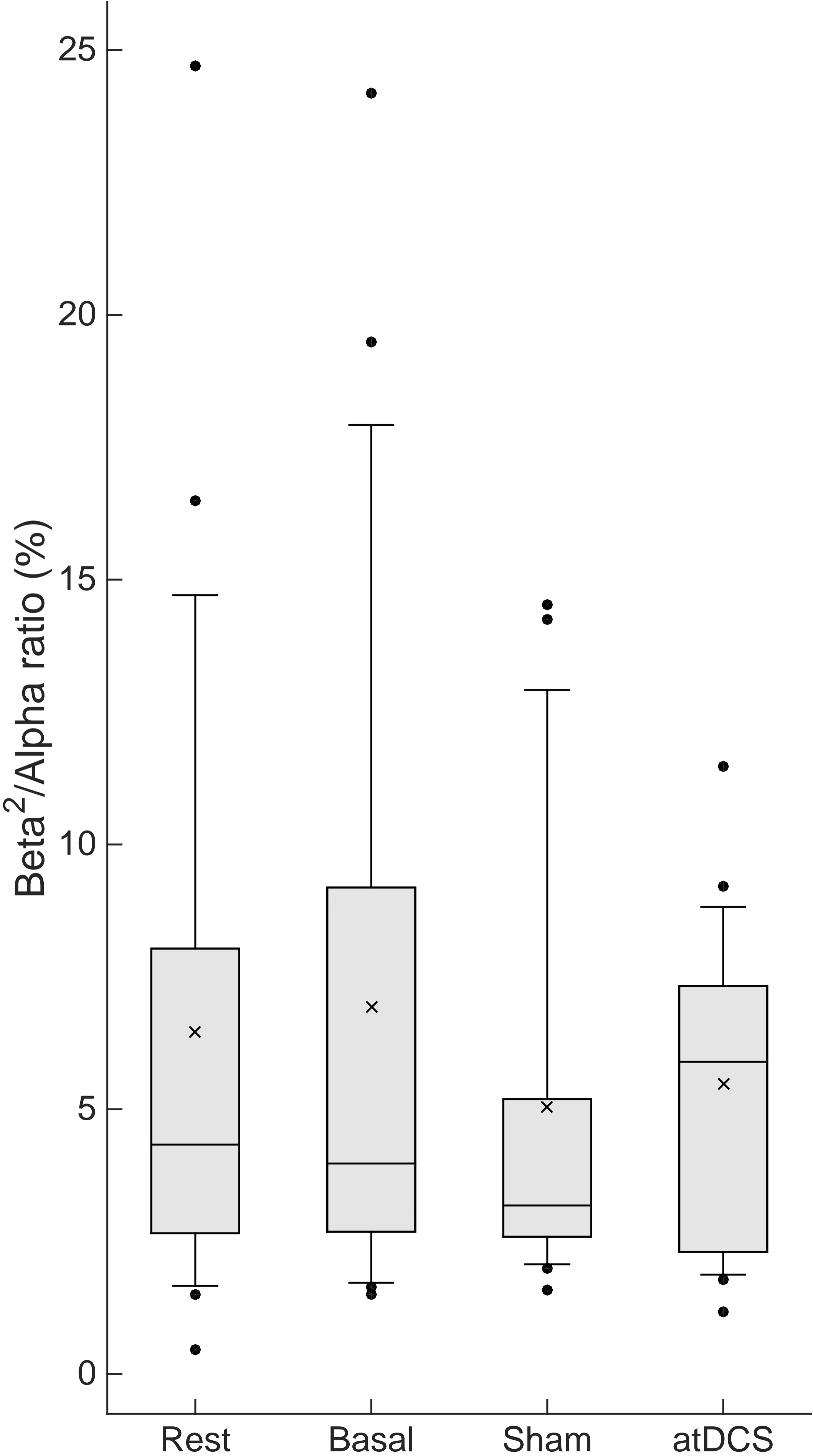

Supplement: Supplementary file 1 [file Data_Sheet_1.zip › Complementary_results/Band_ratios_average_PSD_windows/Beta^2-Alpha/Beta^2-Alpha_mean-win_F3.pdf]

**Beta<sup>2</sup>/Alpha ratio on average  
PSD windows for electrode: F4**

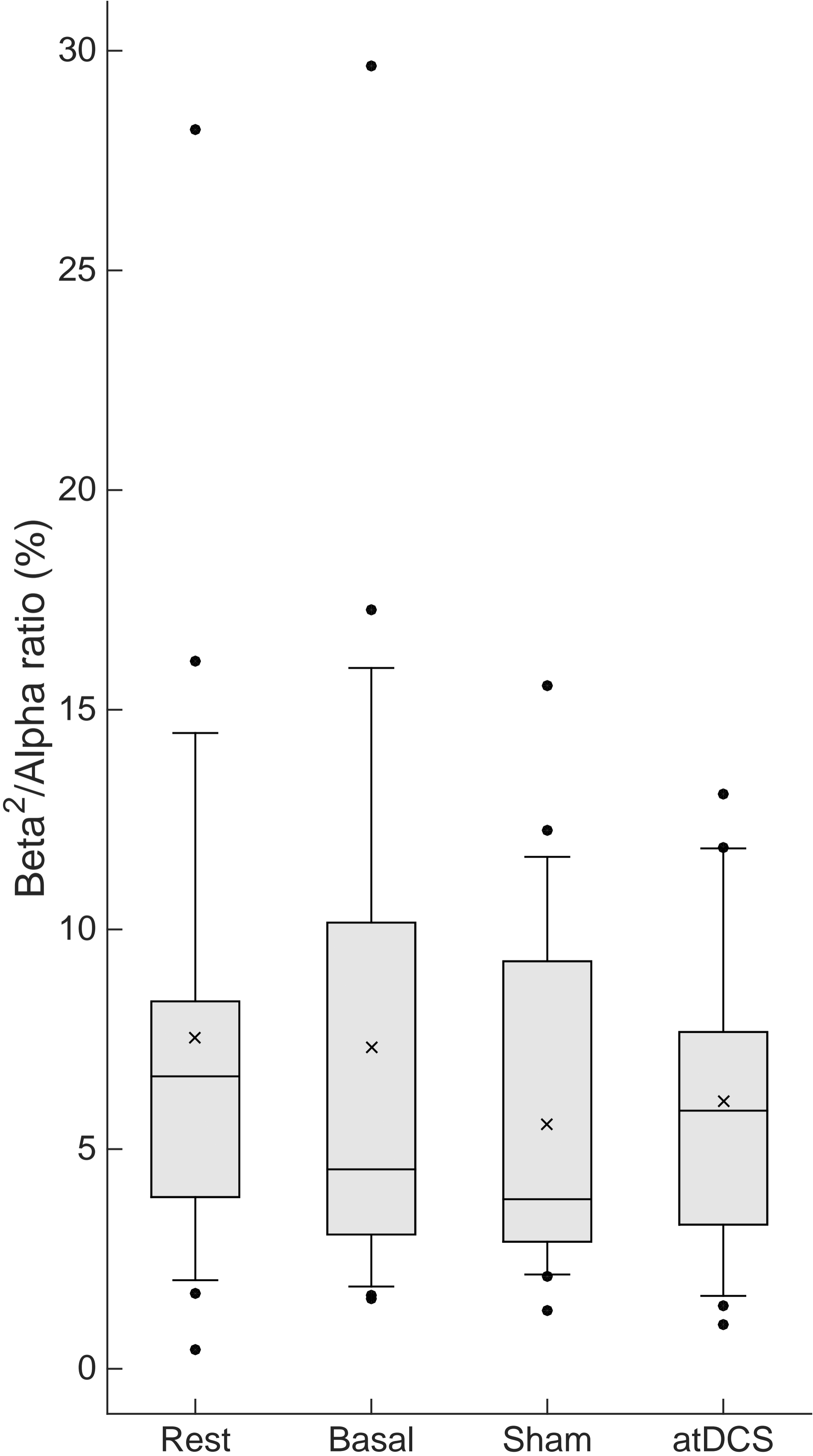

Supplement: Supplementary file 1 [file Data_Sheet_1.zip › Complementary_results/Band_ratios_average_PSD_windows/Beta^2-Alpha/Beta^2-Alpha_mean-win_F4.pdf]

**Beta<sup>2</sup>/Alpha ratio on average  
PSD windows for electrode: F7**

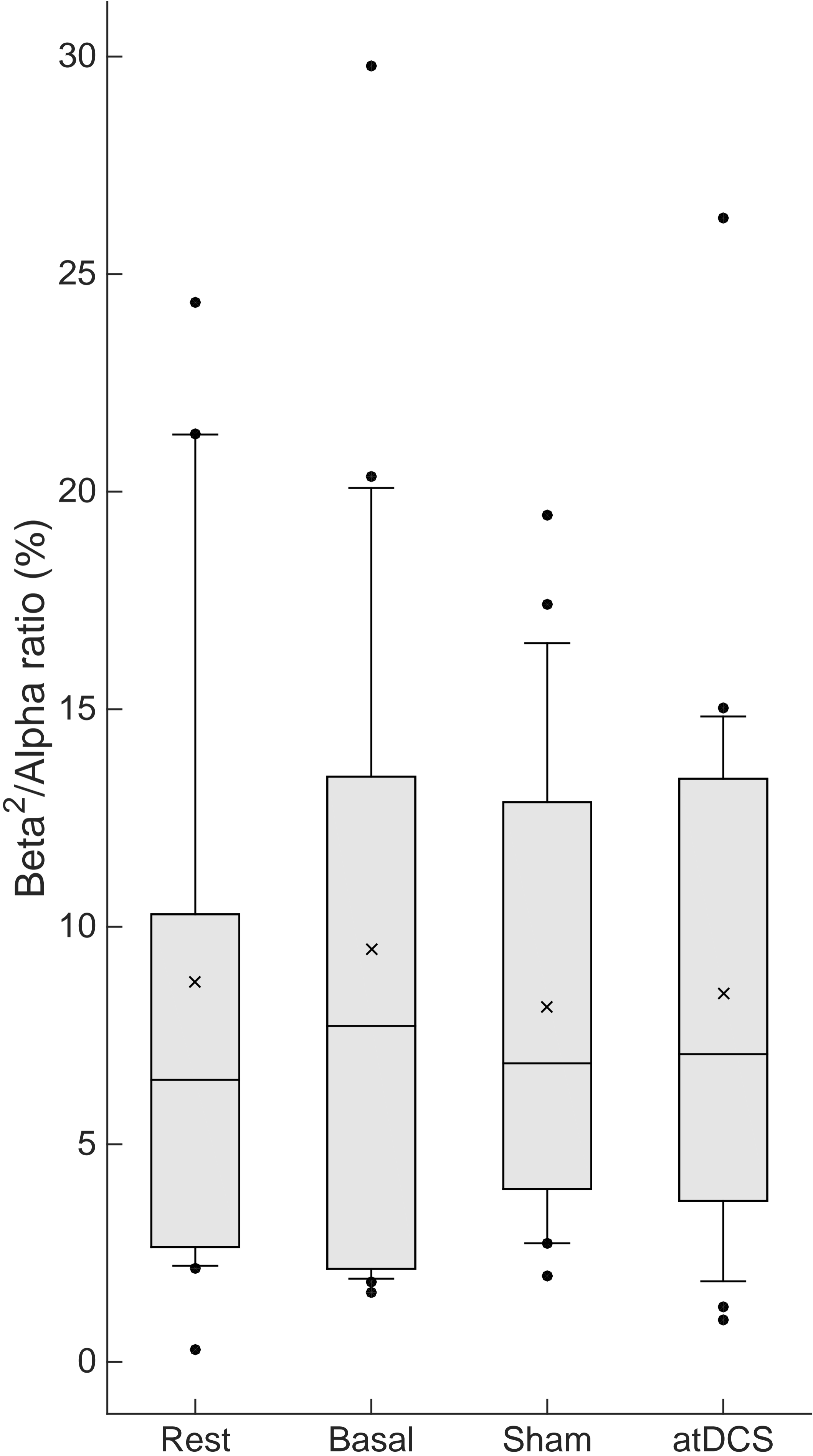

Supplement: Supplementary file 1 [file Data_Sheet_1.zip › Complementary_results/Band_ratios_average_PSD_windows/Beta^2-Alpha/Beta^2-Alpha_mean-win_F7.pdf]

**Beta<sup>2</sup>/Alpha ratio on average  
PSD windows for electrode: F8**

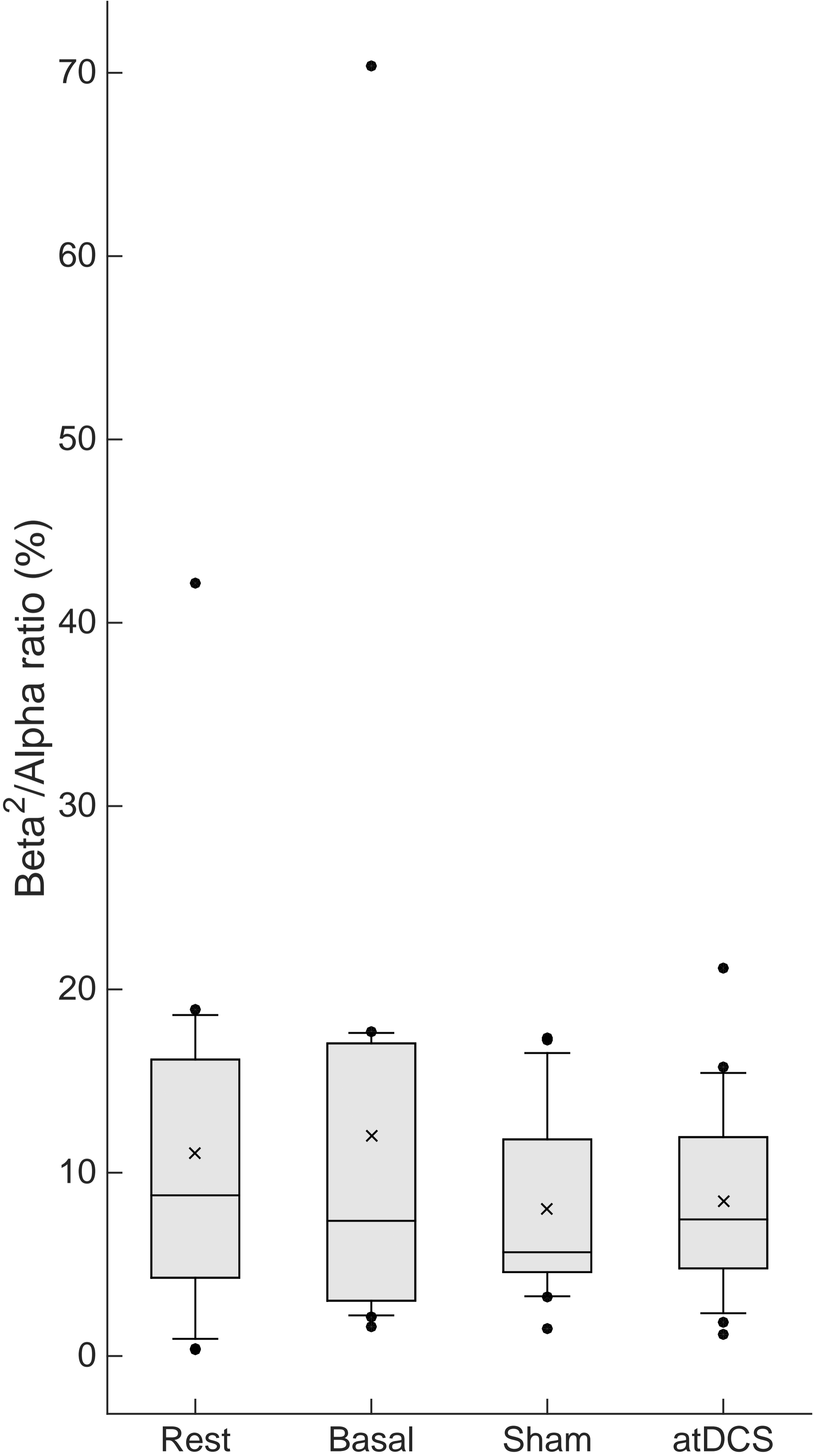

Supplement: Supplementary file 1 [file Data_Sheet_1.zip › Complementary_results/Band_ratios_average_PSD_windows/Beta^2-Alpha/Beta^2-Alpha_mean-win_F8.pdf]

**Beta<sup>2</sup>/Alpha ratio on average  
PSD windows for electrode: FC5**

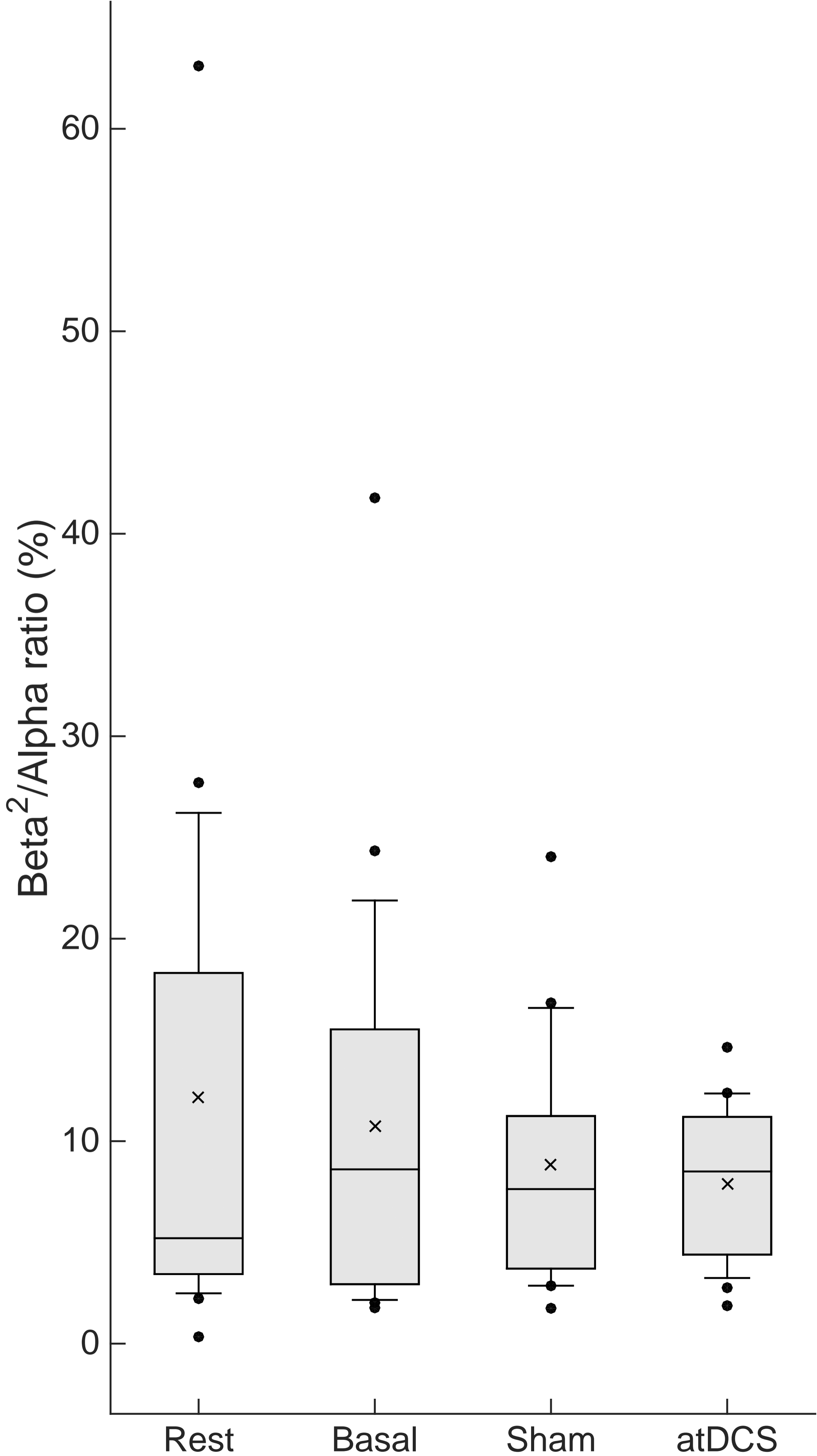

Supplement: Supplementary file 1 [file Data_Sheet_1.zip › Complementary_results/Band_ratios_average_PSD_windows/Beta^2-Alpha/Beta^2-Alpha_mean-win_FC5.pdf]

**Beta<sup>2</sup>/Alpha ratio on average  
PSD windows for electrode: FC6**

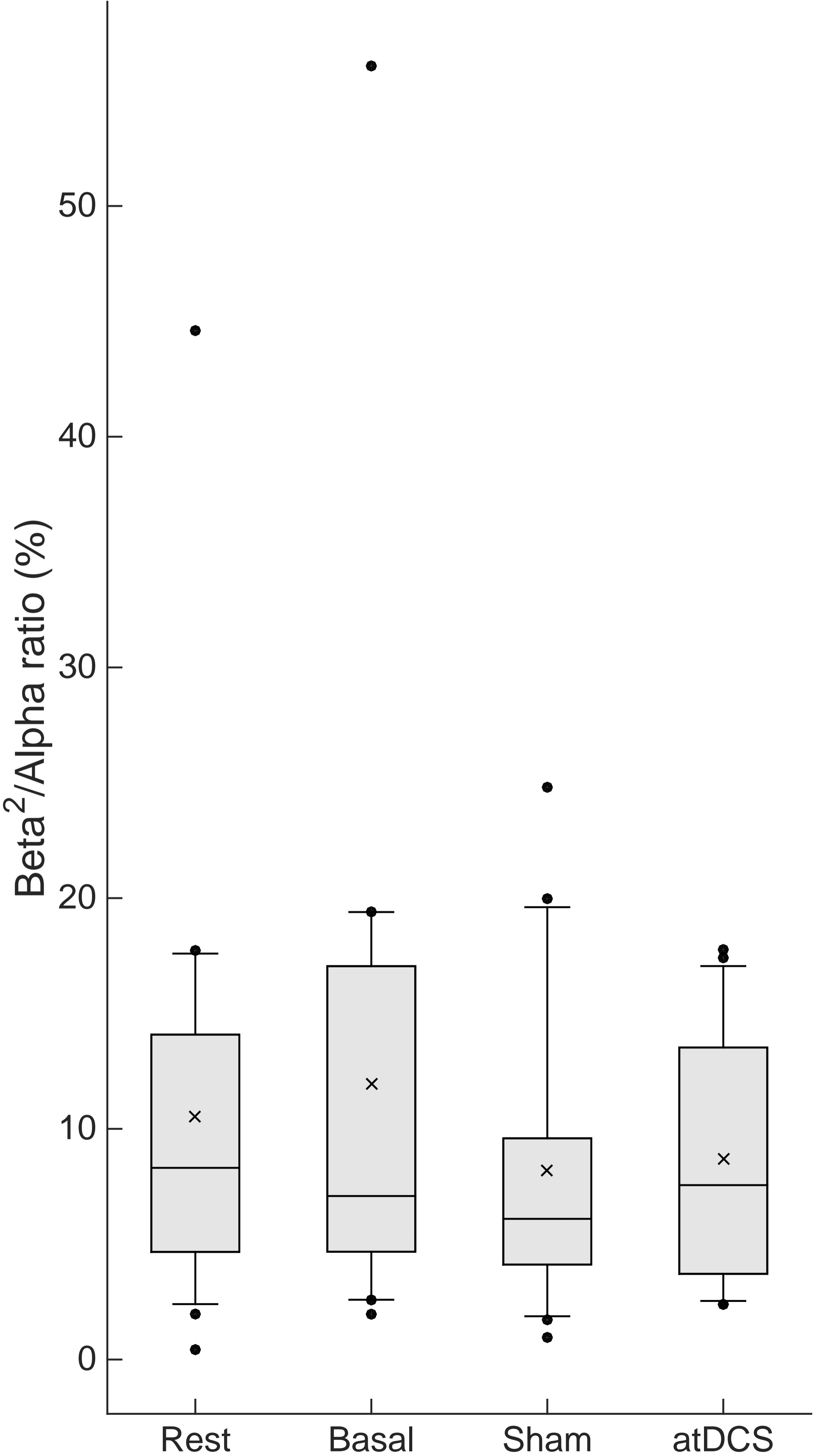

Supplement: Supplementary file 1 [file Data_Sheet_1.zip › Complementary_results/Band_ratios_average_PSD_windows/Beta^2-Alpha/Beta^2-Alpha_mean-win_FC6.pdf]

**Beta<sup>2</sup>/Alpha ratio on average  
PSD windows for electrode: O1**

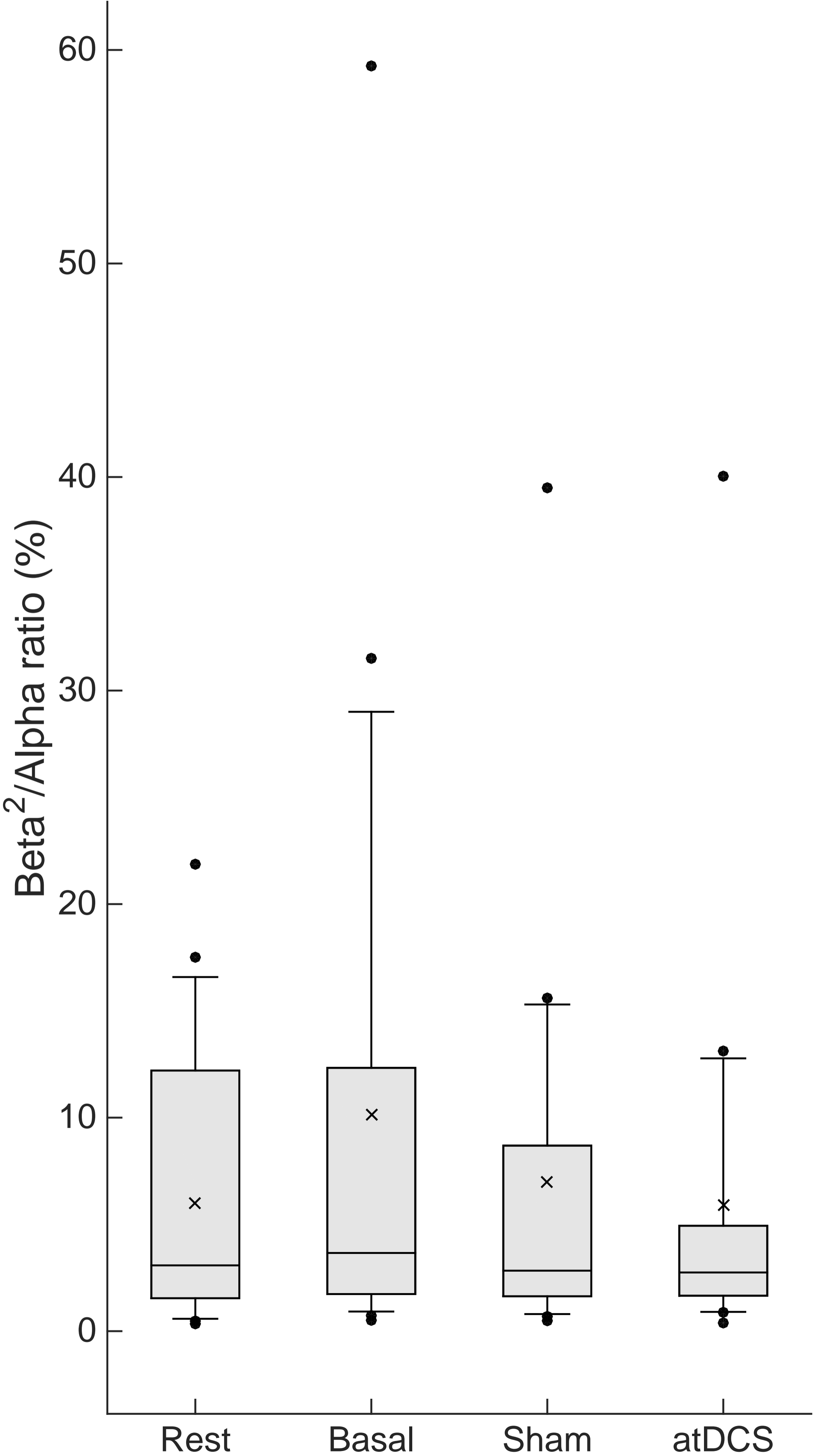

Supplement: Supplementary file 1 [file Data_Sheet_1.zip › Complementary_results/Band_ratios_average_PSD_windows/Beta^2-Alpha/Beta^2-Alpha_mean-win_O1.pdf]

**Beta<sup>2</sup>/Alpha ratio on average  
PSD windows for electrode: O2**

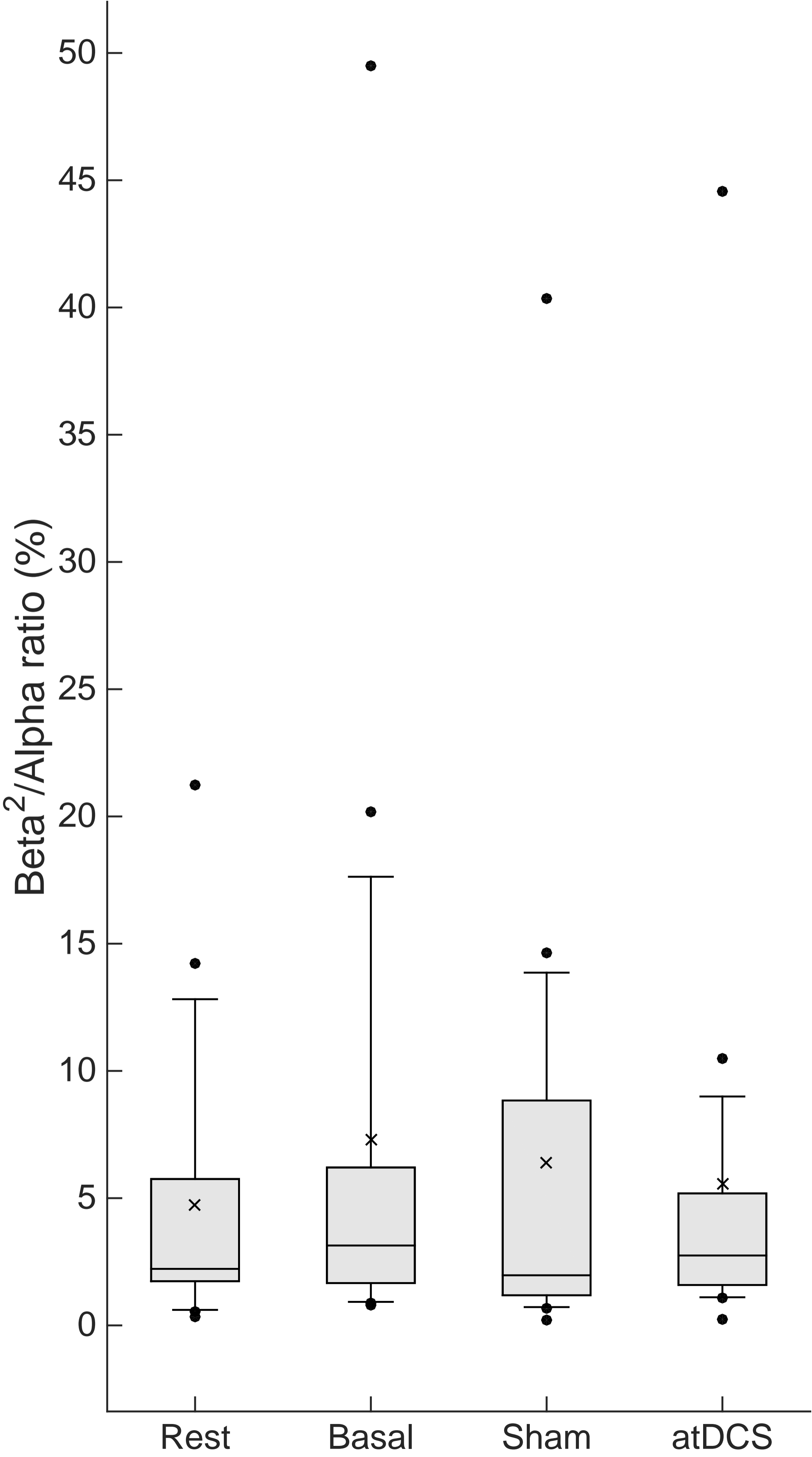

Supplement: Supplementary file 1 [file Data_Sheet_1.zip › Complementary_results/Band_ratios_average_PSD_windows/Beta^2-Alpha/Beta^2-Alpha_mean-win_O2.pdf]

**Beta<sup>2</sup>/Alpha ratio on average  
PSD windows for electrode: P7**

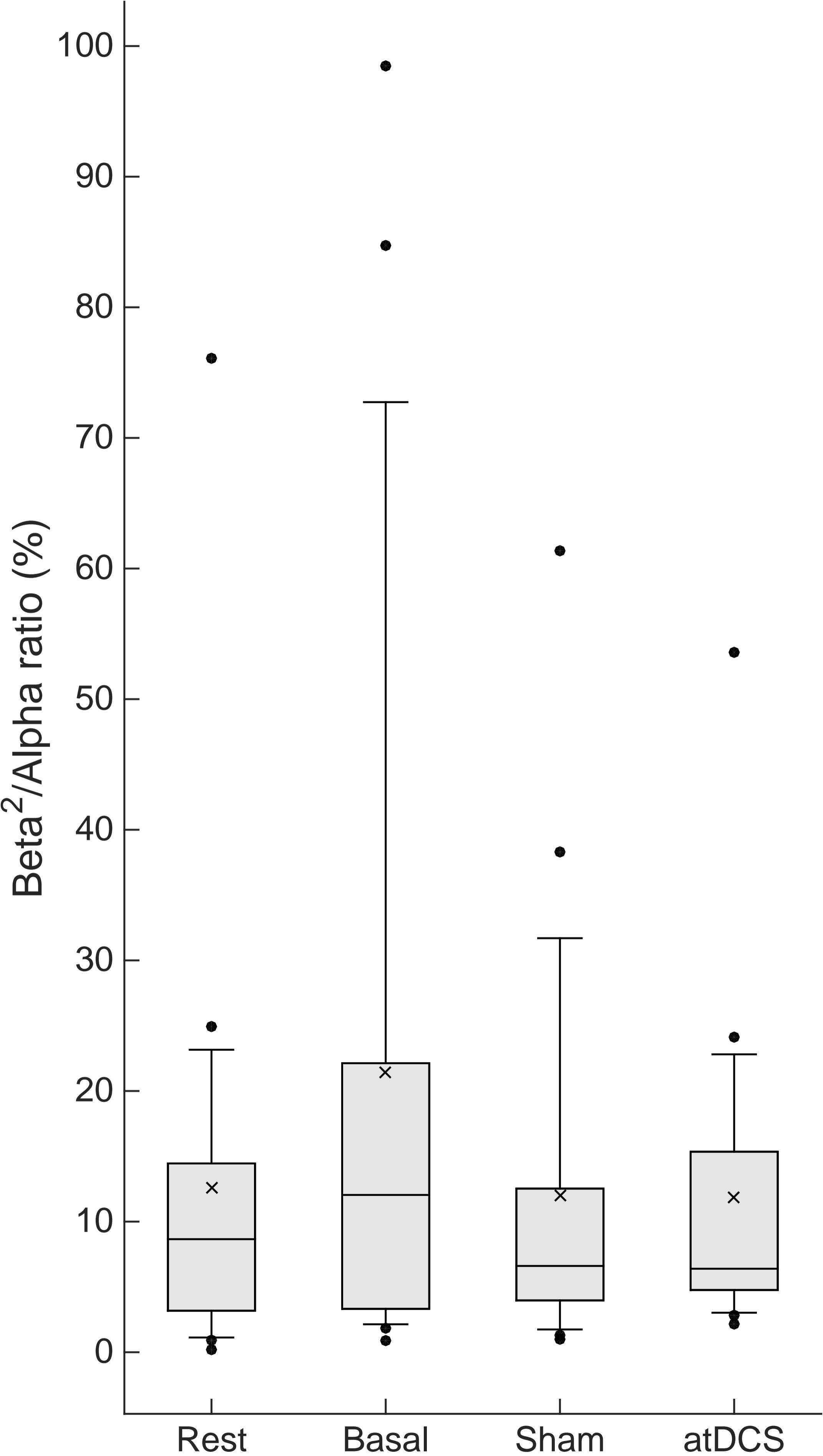

Supplement: Supplementary file 1 [file Data_Sheet_1.zip › Complementary_results/Band_ratios_average_PSD_windows/Beta^2-Alpha/Beta^2-Alpha_mean-win_P7.pdf]

**Beta<sup>2</sup>/Alpha ratio on average  
PSD windows for electrode: P8**

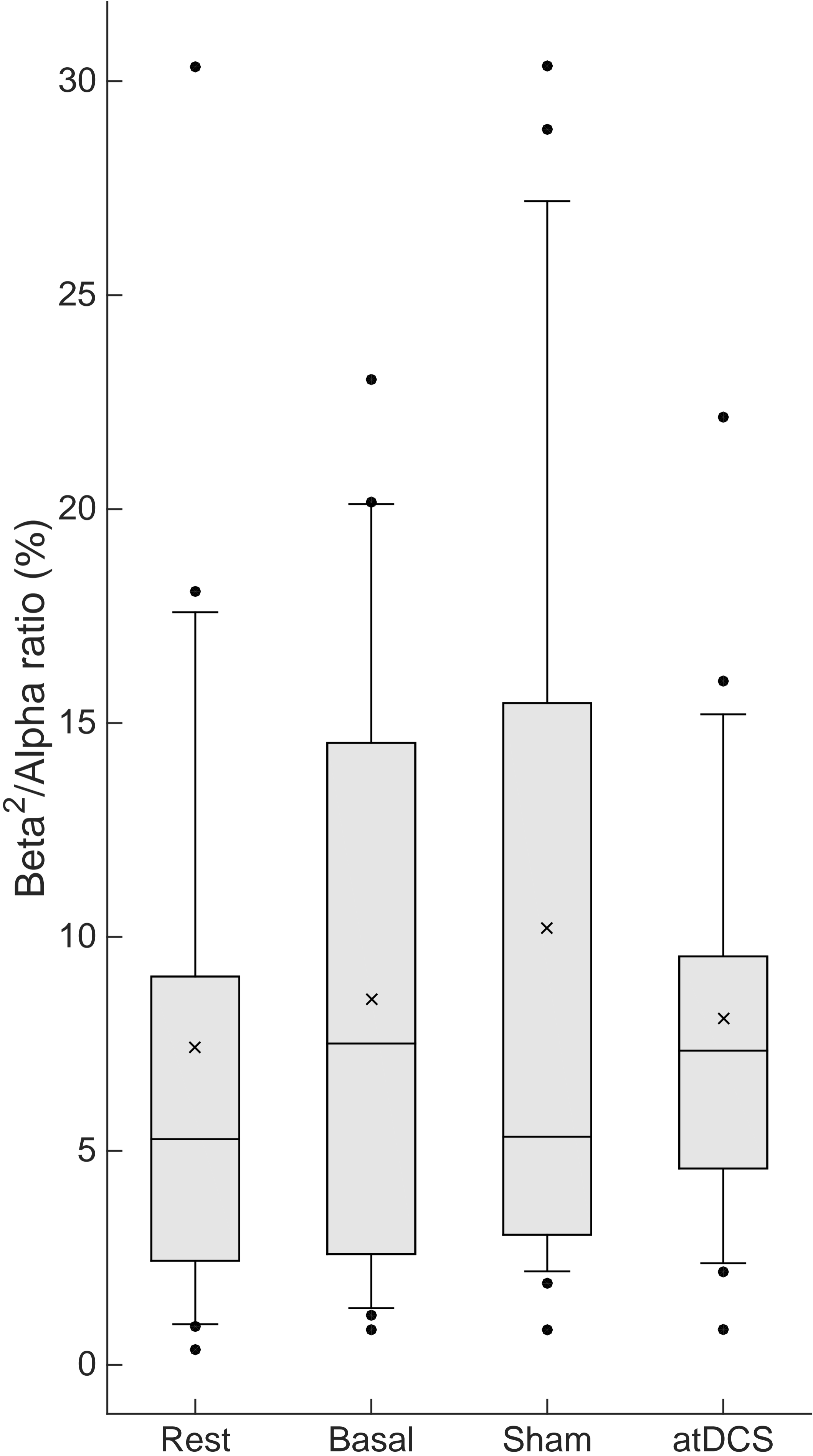

Supplement: Supplementary file 1 [file Data_Sheet_1.zip › Complementary_results/Band_ratios_average_PSD_windows/Beta^2-Alpha/Beta^2-Alpha_mean-win_P8.pdf]

**Beta<sup>2</sup>/Alpha ratio on average  
PSD windows for electrode: T7**

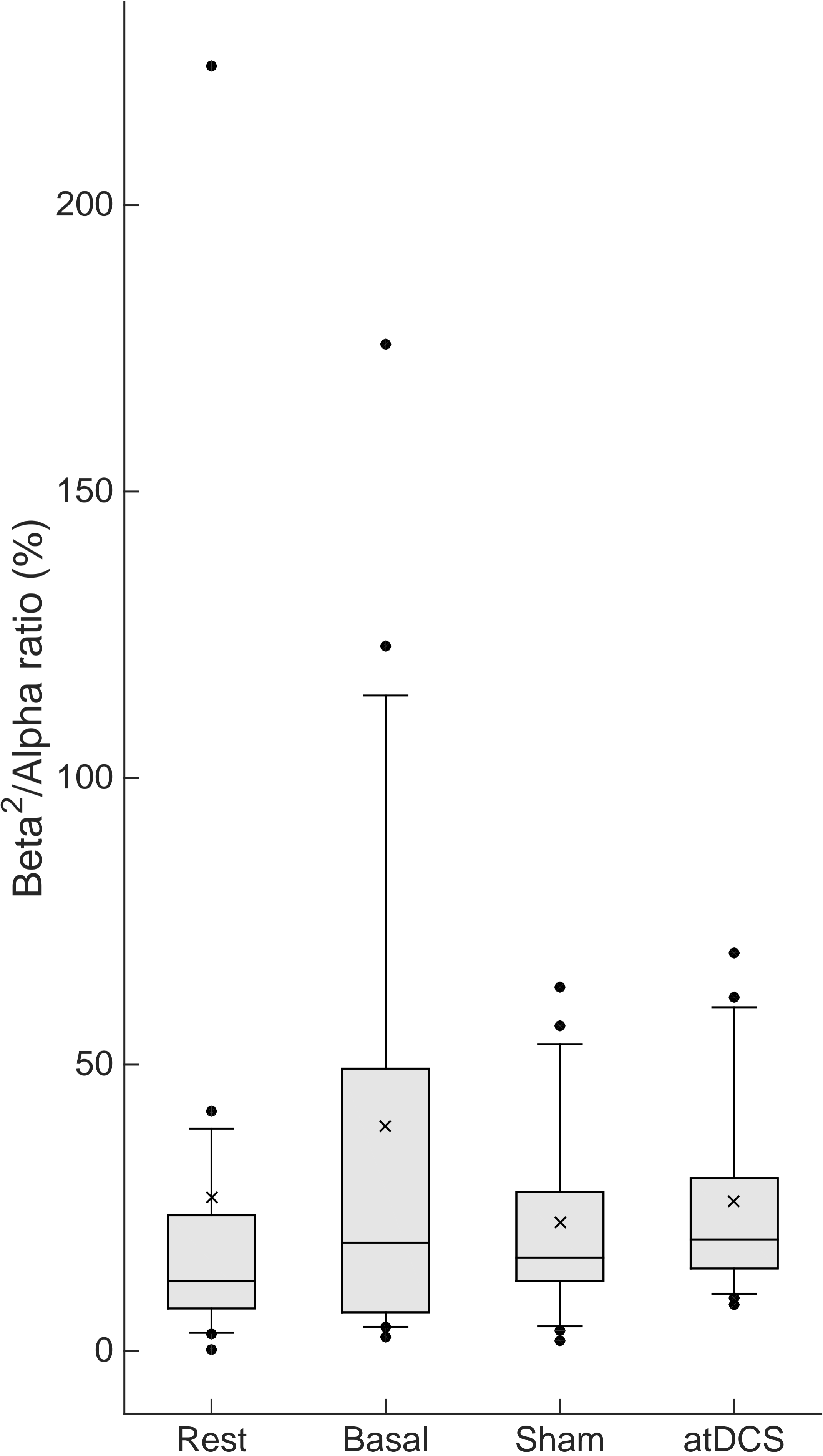

Supplement: Supplementary file 1 [file Data_Sheet_1.zip › Complementary_results/Band_ratios_average_PSD_windows/Beta^2-Alpha/Beta^2-Alpha_mean-win_T7.pdf]

**Beta<sup>2</sup>/Alpha ratio on average  
PSD windows for electrode: T8**

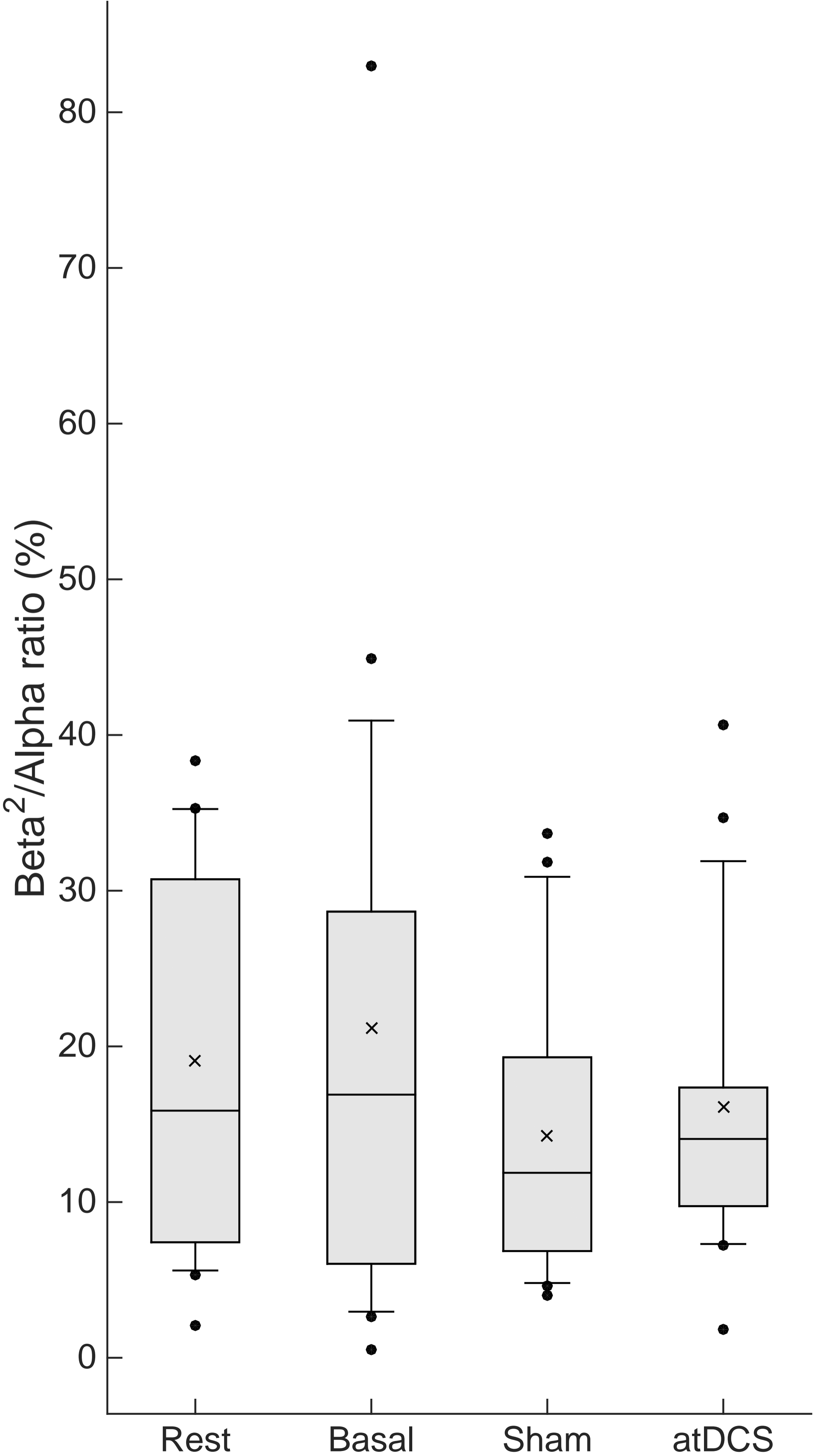

Supplement: Supplementary file 1 [file Data_Sheet_1.zip › Complementary_results/Band_ratios_average_PSD_windows/Beta^2-Alpha/Beta^2-Alpha_mean-win_T8.pdf]

**Beta/Alpha ratio on average  
PSD windows for electrode: AF3**

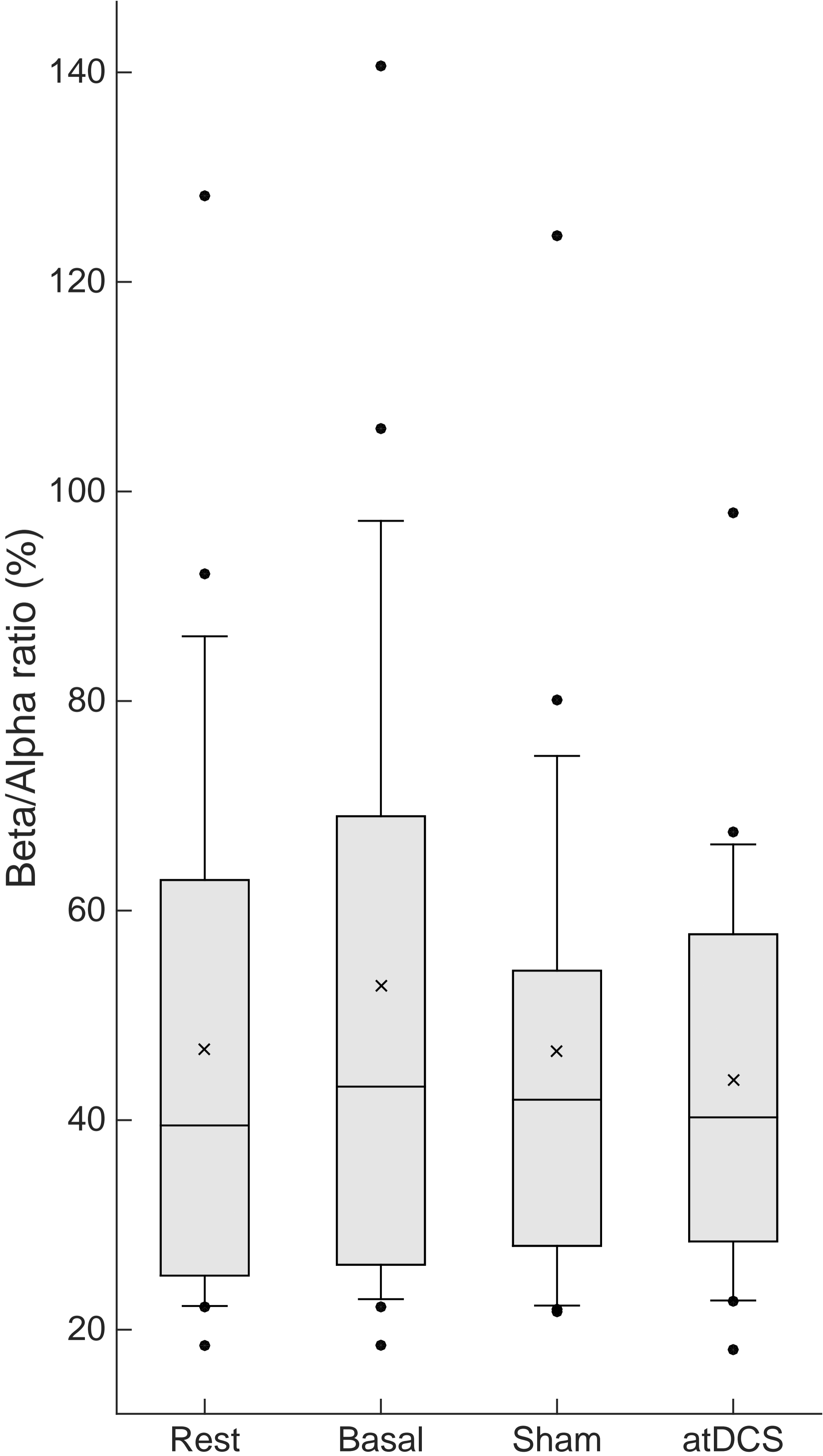

Supplement: Supplementary file 1 [file Data_Sheet_1.zip › Complementary_results/Band_ratios_average_PSD_windows/Beta_Alpha/Beta-Alpha_mean-win_AF3.pdf]

**Beta/Alpha ratio on average  
PSD windows for electrode: AF4**

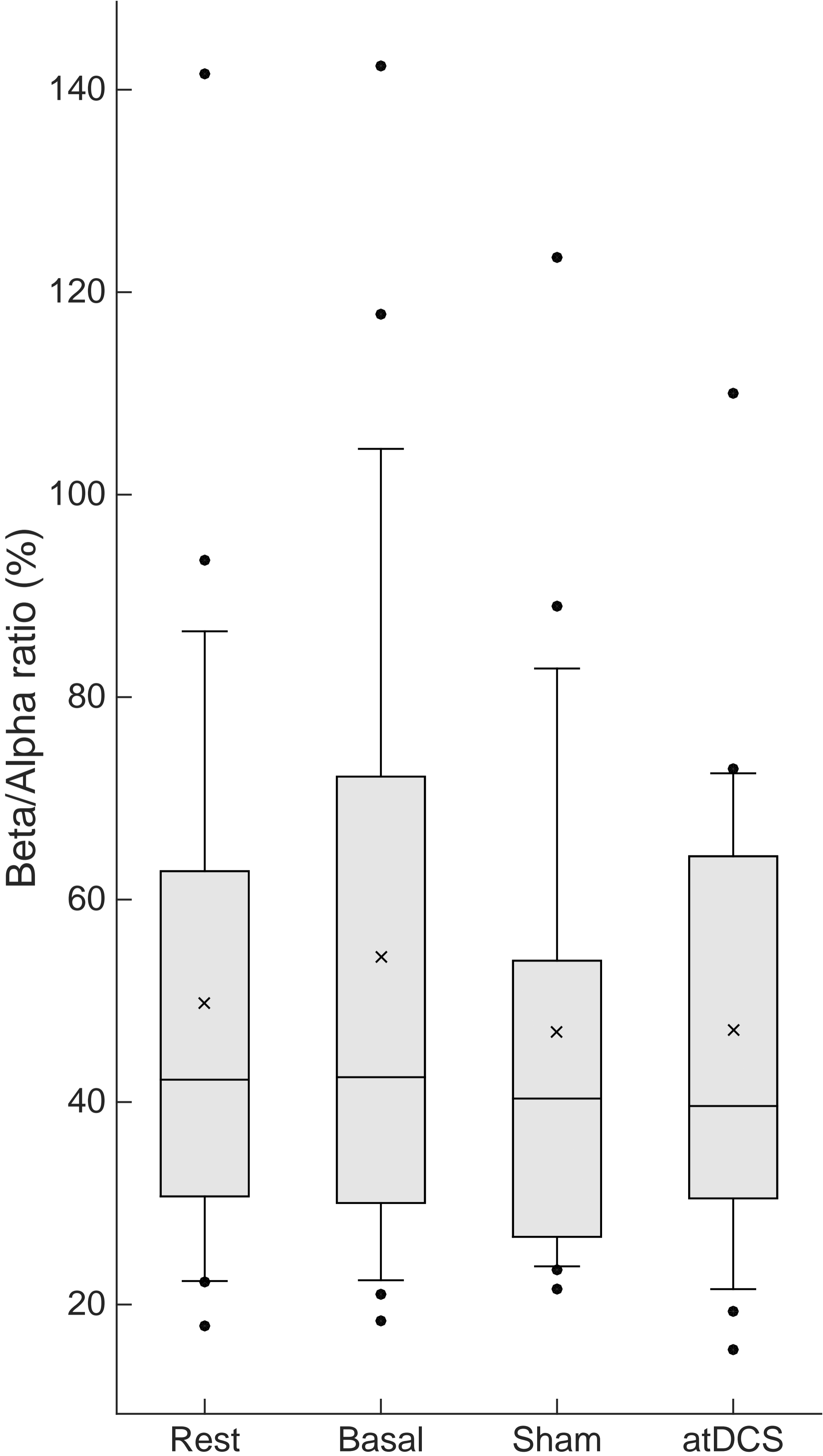

Supplement: Supplementary file 1 [file Data_Sheet_1.zip › Complementary_results/Band_ratios_average_PSD_windows/Beta_Alpha/Beta-Alpha_mean-win_AF4.pdf]

**Beta/Alpha ratio on average**  
**PSD windows for electrode: Avg AF3-F3-F7**

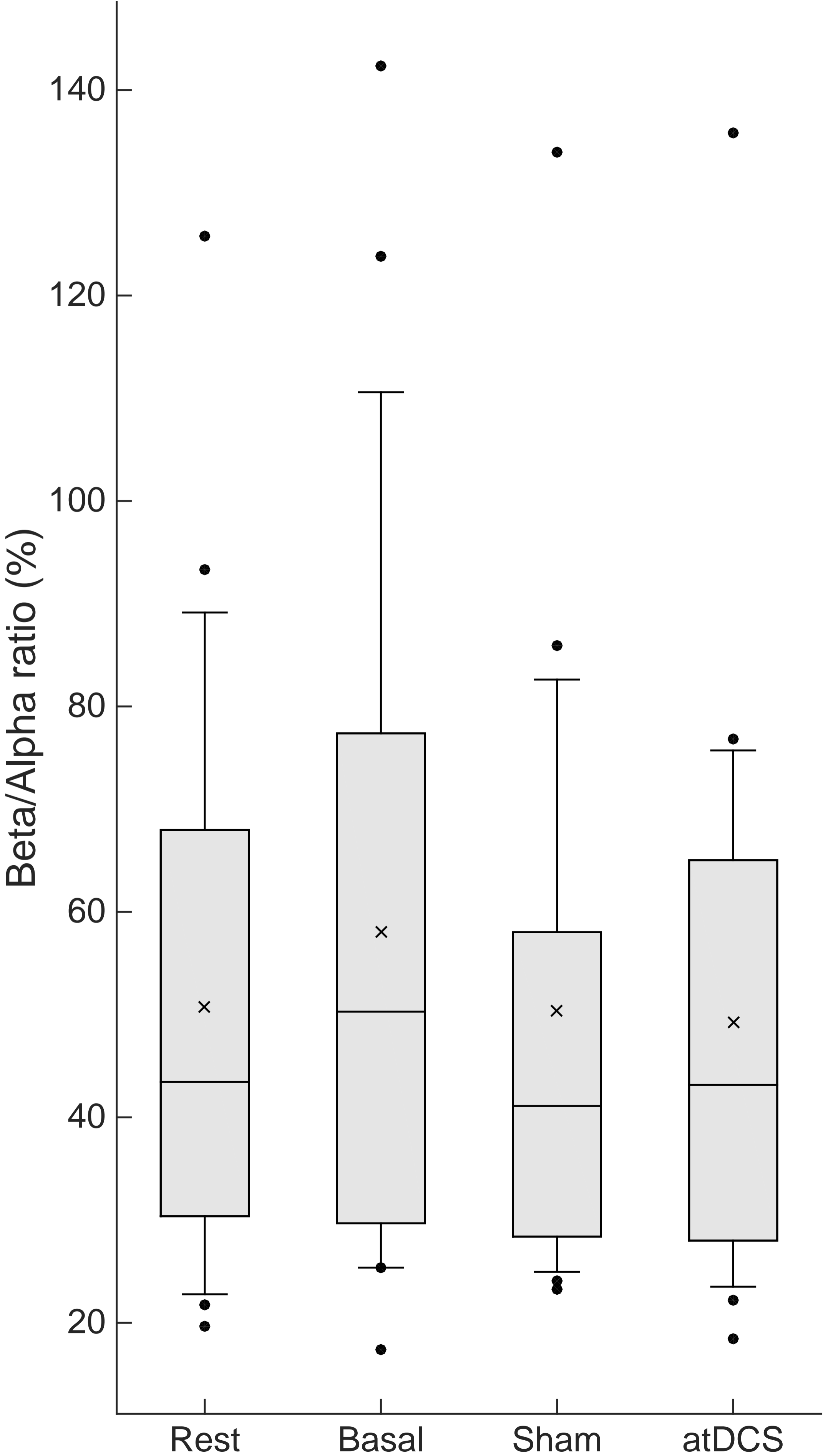

Supplement: Supplementary file 1 [file Data_Sheet_1.zip › Complementary_results/Band_ratios_average_PSD_windows/Beta_Alpha/Beta-Alpha_mean-win_Avg AF3-F3-F7.pdf]

**Beta/Alpha ratio on average**  
**PSD windows for electrode: Avg AF4-F4-F8**

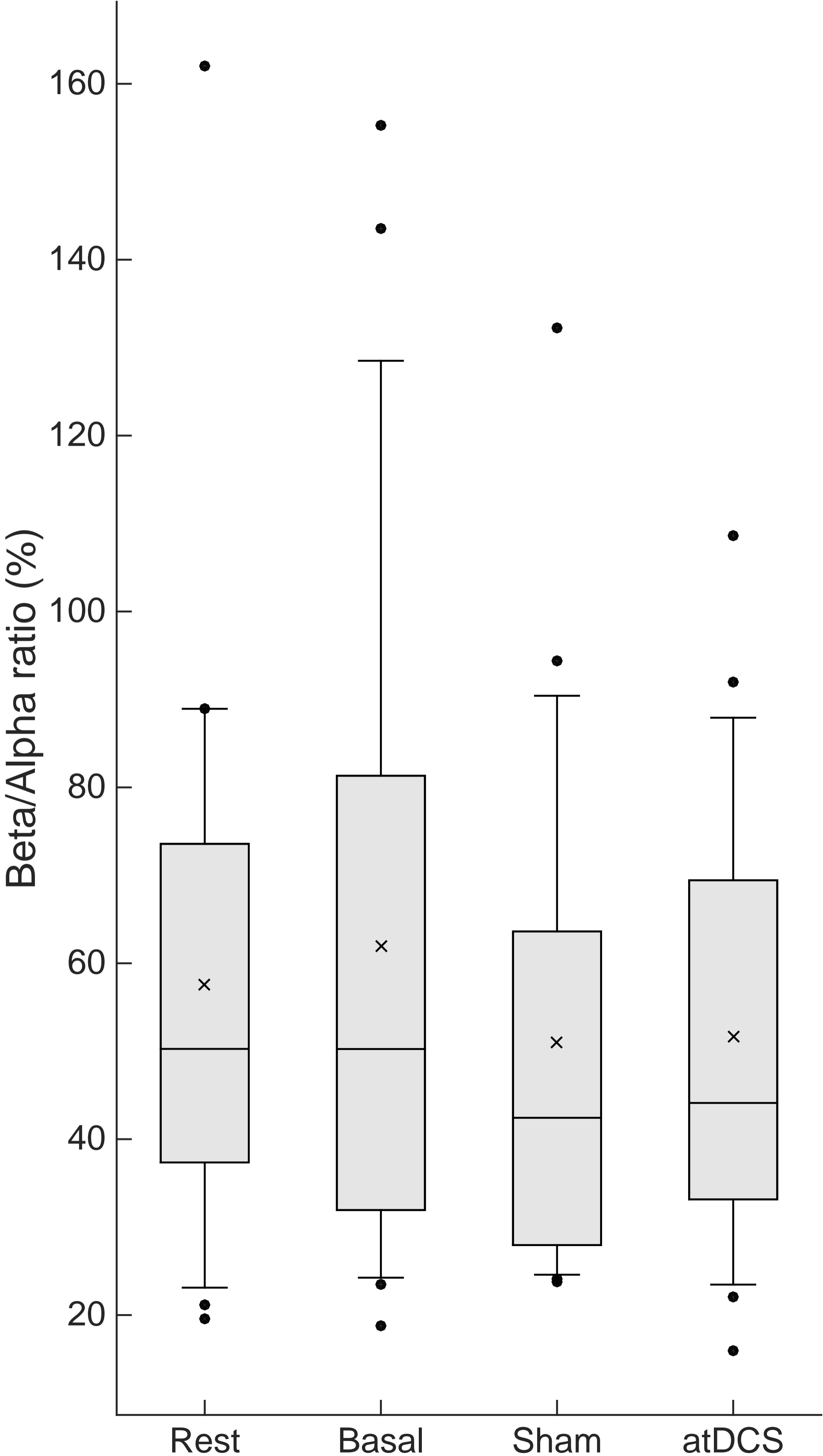

Supplement: Supplementary file 1 [file Data_Sheet_1.zip › Complementary_results/Band_ratios_average_PSD_windows/Beta_Alpha/Beta-Alpha_mean-win_Avg AF4-F4-F8.pdf]

**Beta/Alpha ratio on average**  
**PSD windows for electrode: Avg F3-F7-FC5**

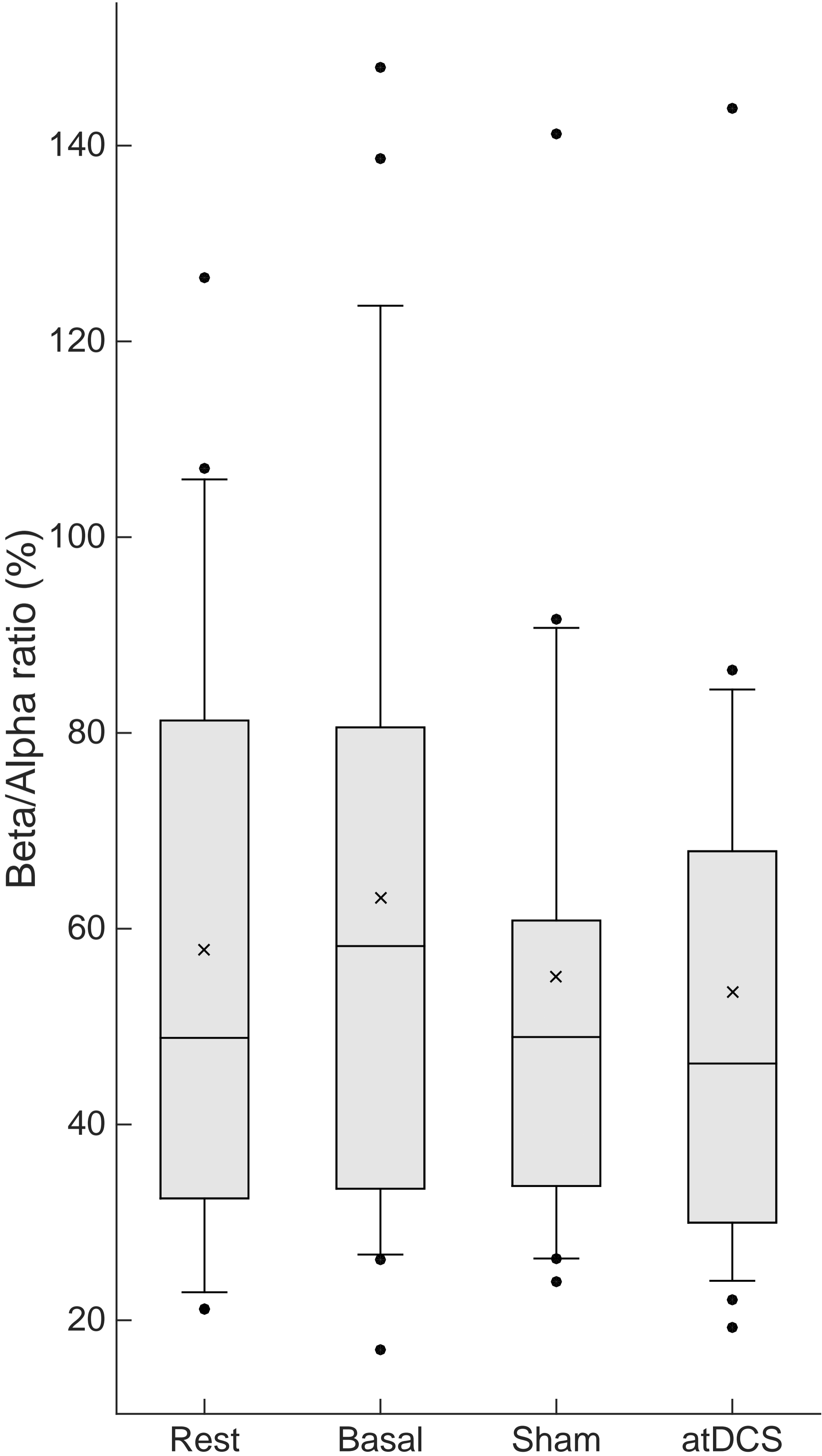

Supplement: Supplementary file 1 [file Data_Sheet_1.zip › Complementary_results/Band_ratios_average_PSD_windows/Beta_Alpha/Beta-Alpha_mean-win_Avg F3-F7-FC5.pdf]

**Beta/Alpha ratio on average**  
**PSD windows for electrode: Avg F4-F8-FC6**

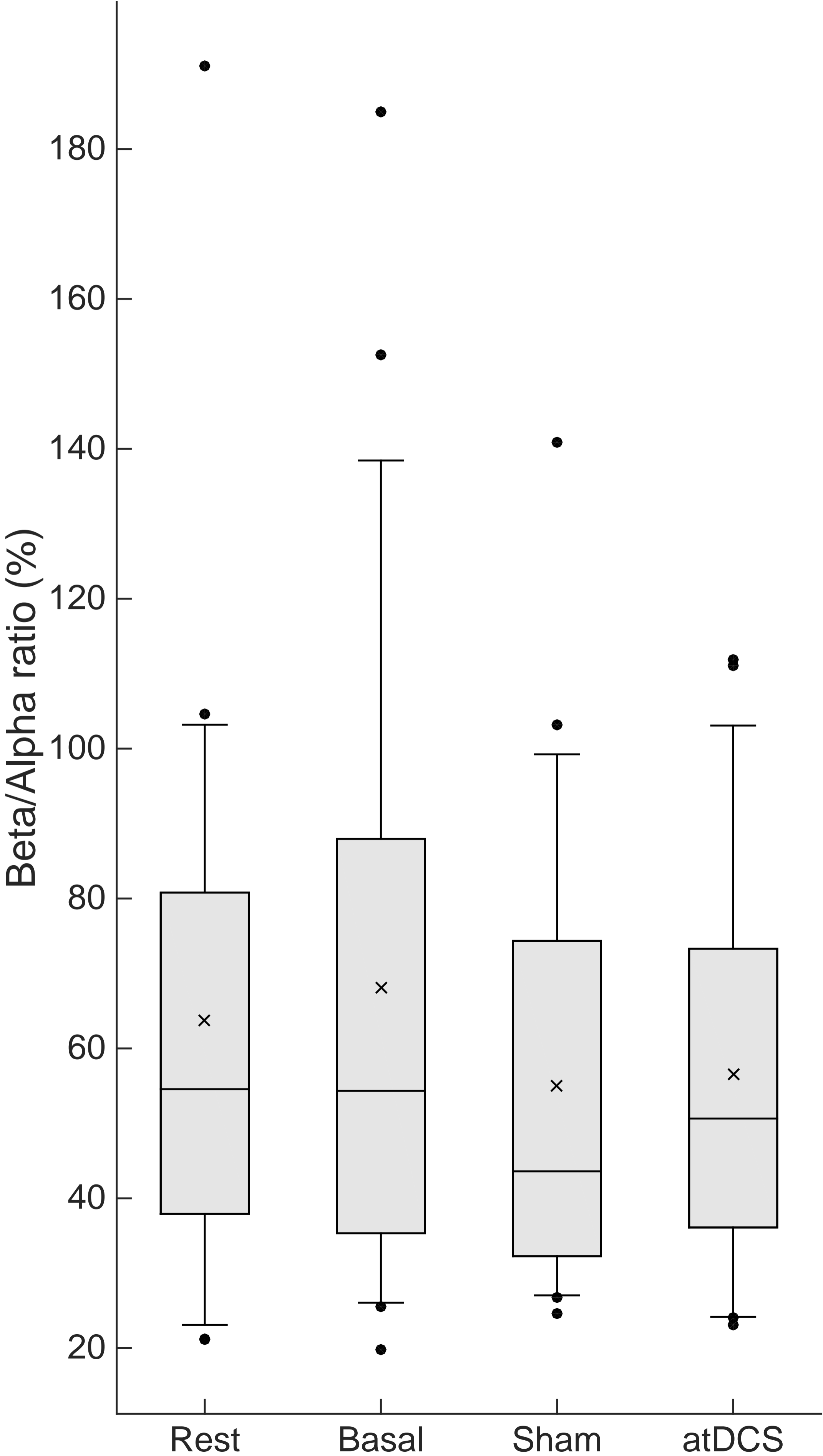

Supplement: Supplementary file 1 [file Data_Sheet_1.zip › Complementary_results/Band_ratios_average_PSD_windows/Beta_Alpha/Beta-Alpha_mean-win_Avg F4-F8-FC6.pdf]

**Beta/Alpha ratio on average  
PSD windows for electrode: F3**

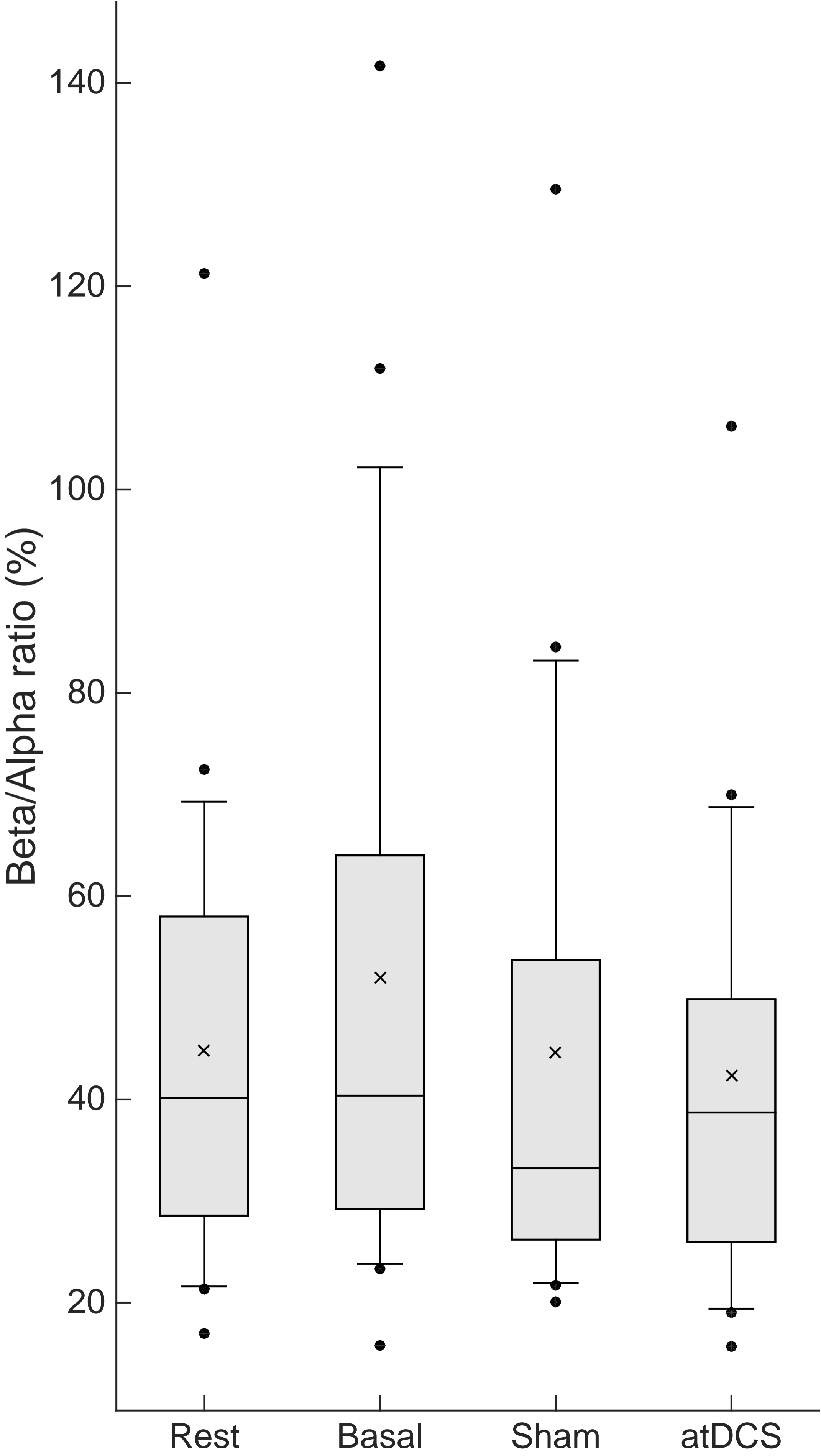

Supplement: Supplementary file 1 [file Data_Sheet_1.zip › Complementary_results/Band_ratios_average_PSD_windows/Beta_Alpha/Beta-Alpha_mean-win_F3.pdf]

**Beta/Alpha ratio on average  
PSD windows for electrode: F4**

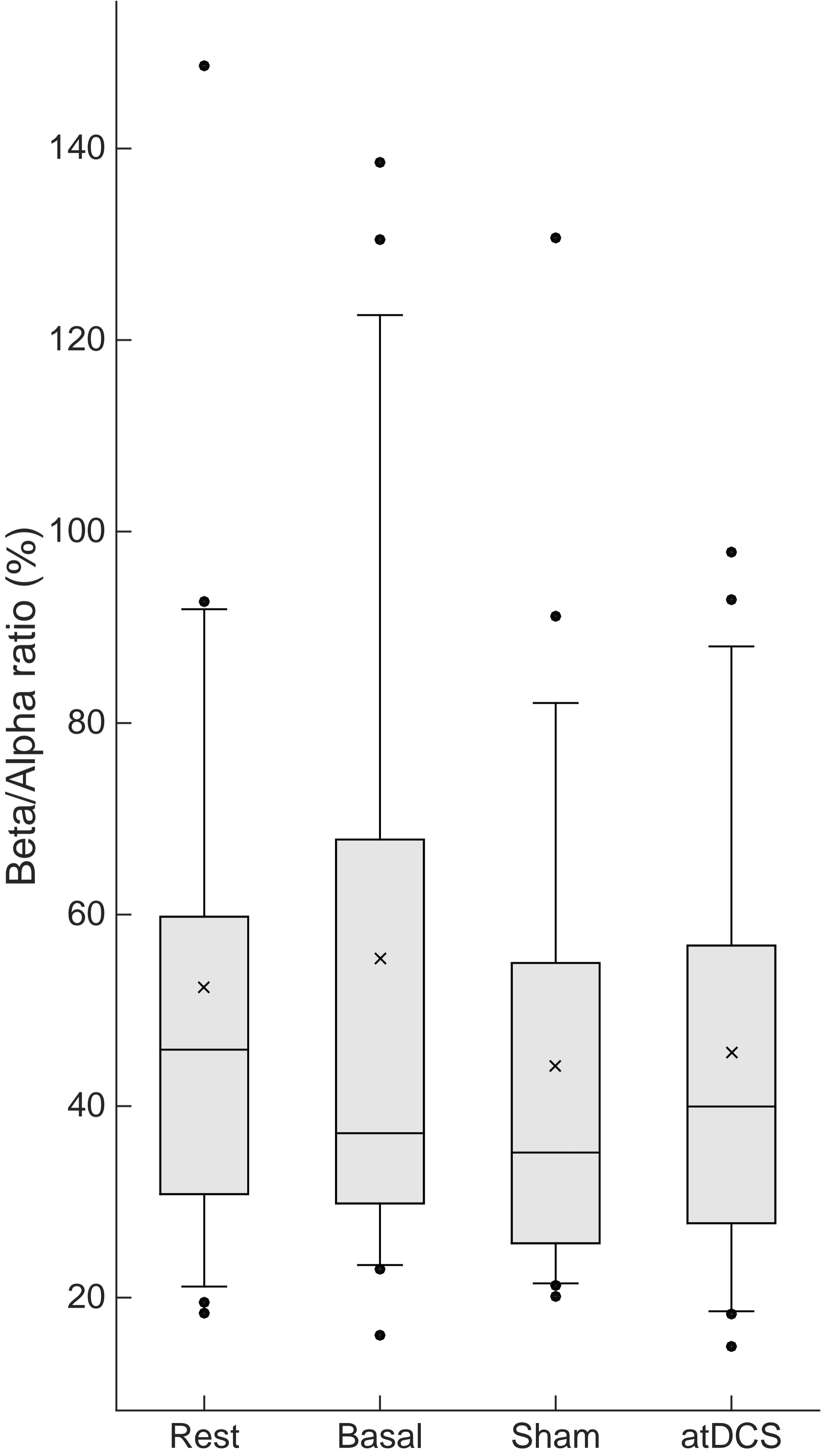

Supplement: Supplementary file 1 [file Data_Sheet_1.zip › Complementary_results/Band_ratios_average_PSD_windows/Beta_Alpha/Beta-Alpha_mean-win_F4.pdf]

**Beta/Alpha ratio on average  
PSD windows for electrode: F7**

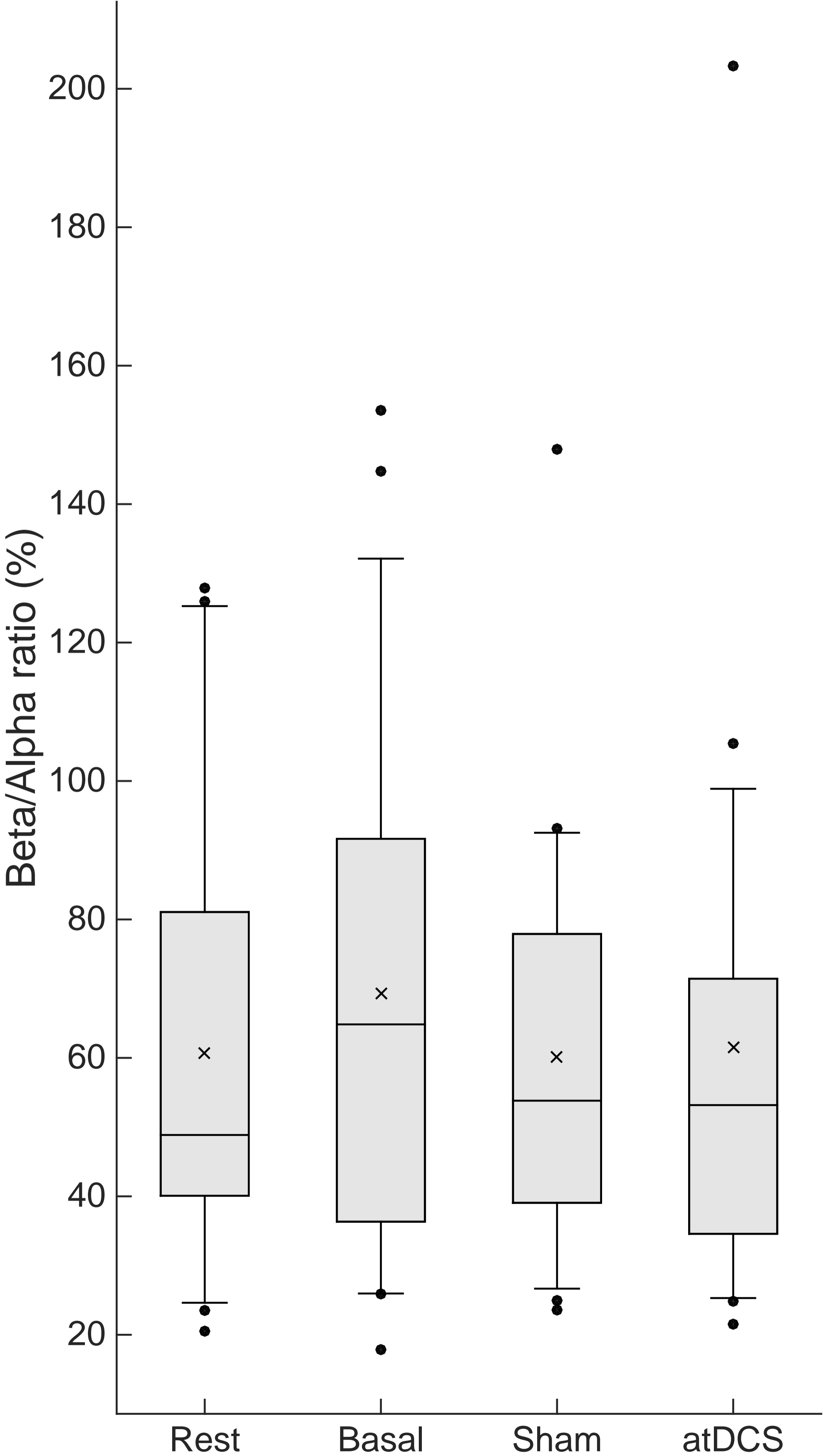

Supplement: Supplementary file 1 [file Data_Sheet_1.zip › Complementary_results/Band_ratios_average_PSD_windows/Beta_Alpha/Beta-Alpha_mean-win_F7.pdf]

**Beta/Alpha ratio on average  
PSD windows for electrode: F8**

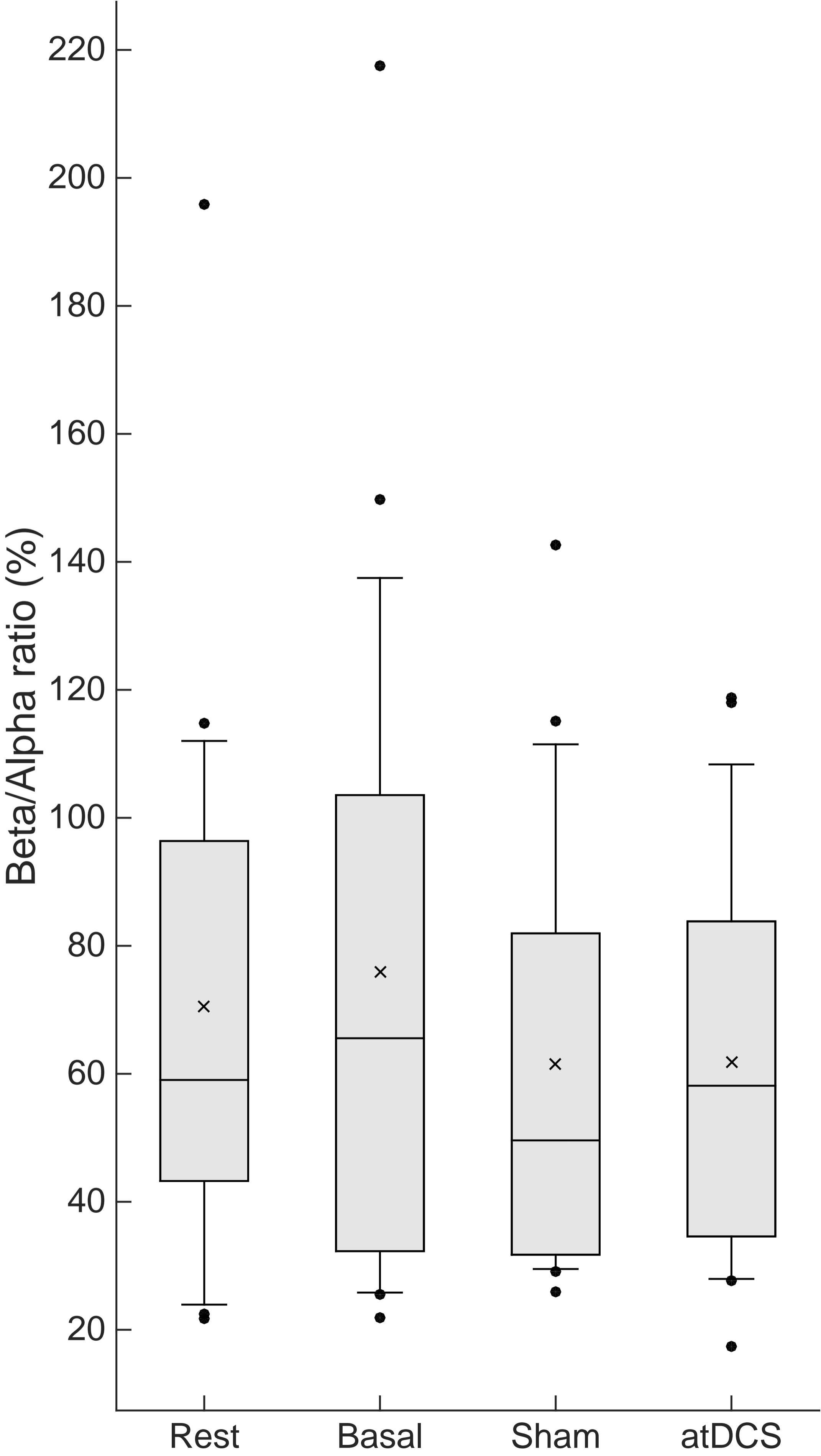

Supplement: Supplementary file 1 [file Data_Sheet_1.zip › Complementary_results/Band_ratios_average_PSD_windows/Beta_Alpha/Beta-Alpha_mean-win_F8.pdf]

**Beta/Alpha ratio on average  
PSD windows for electrode: FC5**

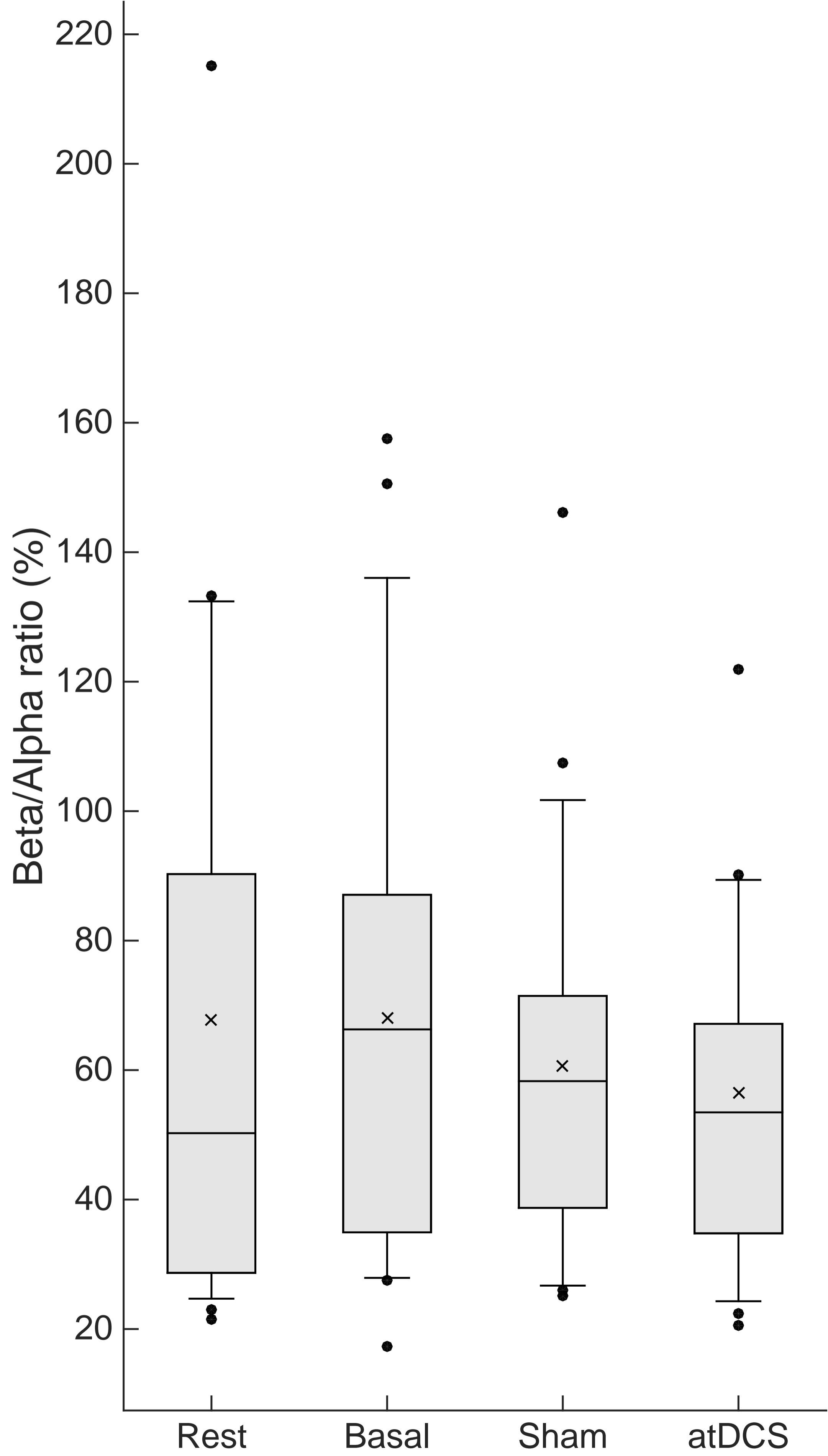

Supplement: Supplementary file 1 [file Data_Sheet_1.zip › Complementary_results/Band_ratios_average_PSD_windows/Beta_Alpha/Beta-Alpha_mean-win_FC5.pdf]

**Beta/Alpha ratio on average  
PSD windows for electrode: FC6**

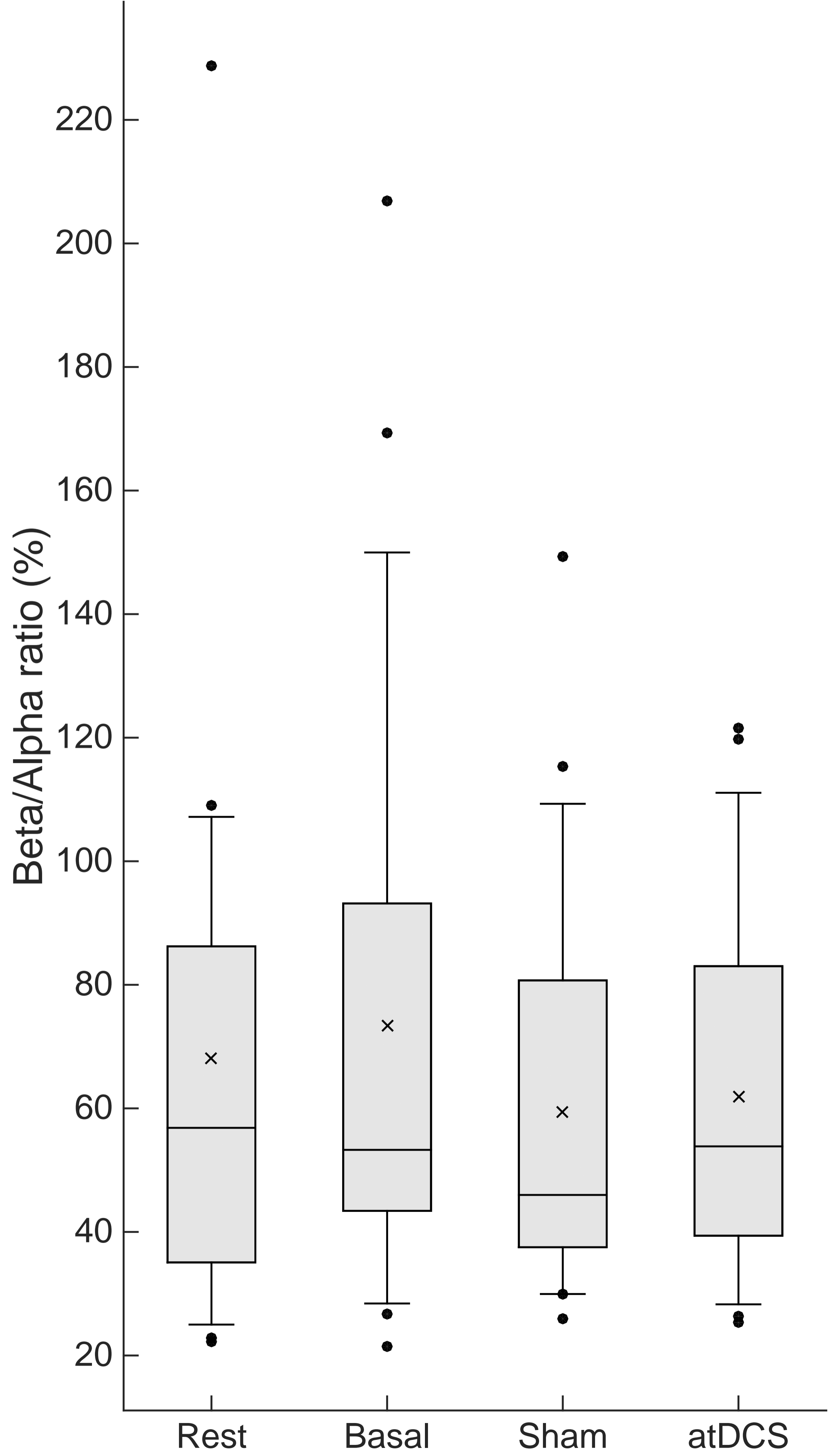

Supplement: Supplementary file 1 [file Data_Sheet_1.zip › Complementary_results/Band_ratios_average_PSD_windows/Beta_Alpha/Beta-Alpha_mean-win_FC6.pdf]

# Beta/Alpha ratio on average PSD windows for electrode: O1

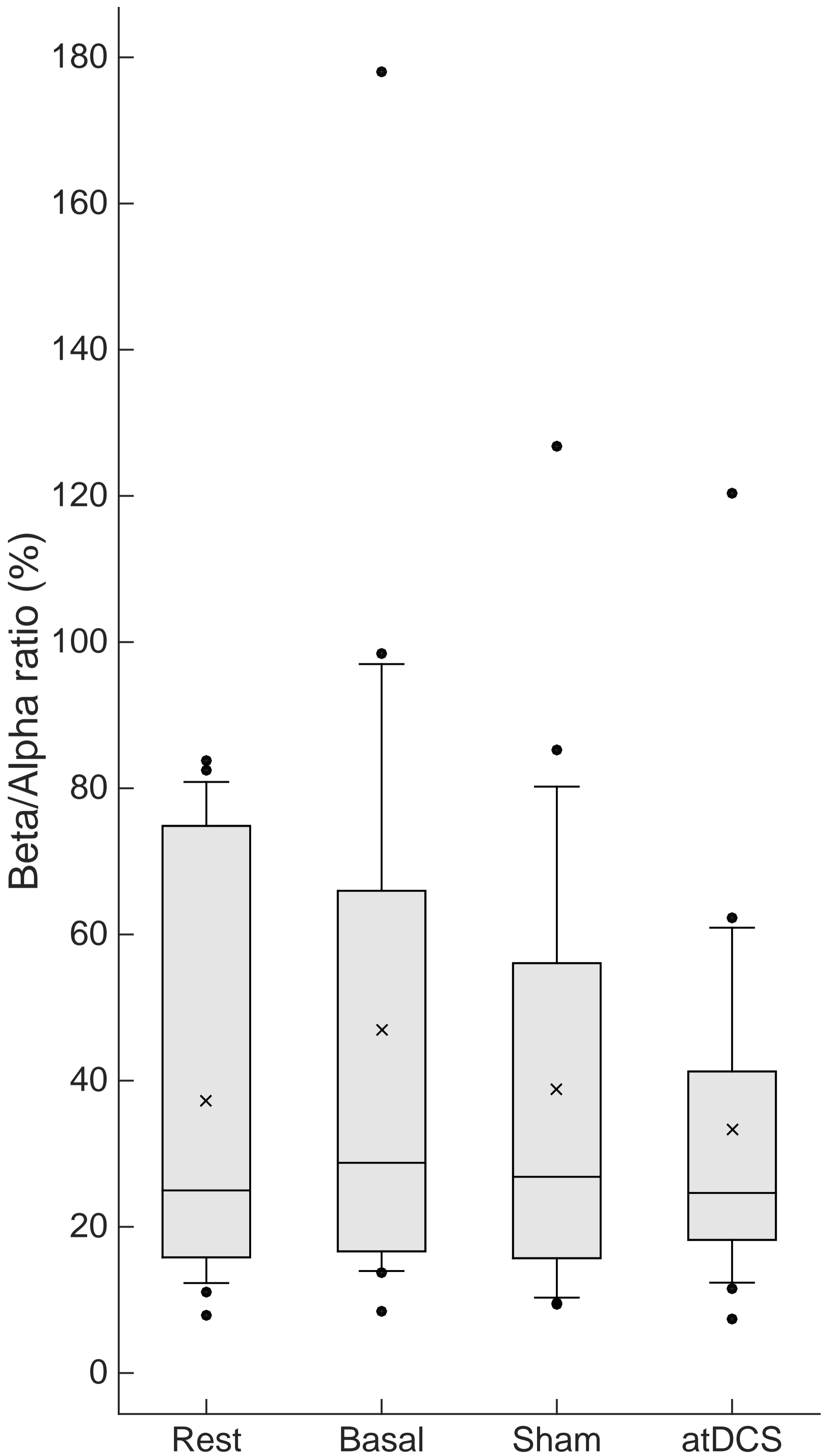

Supplement: Supplementary file 1 [file Data_Sheet_1.zip › Complementary_results/Band_ratios_average_PSD_windows/Beta_Alpha/Beta-Alpha_mean-win_O1.pdf]

# Beta/Alpha ratio on average PSD windows for electrode: O2

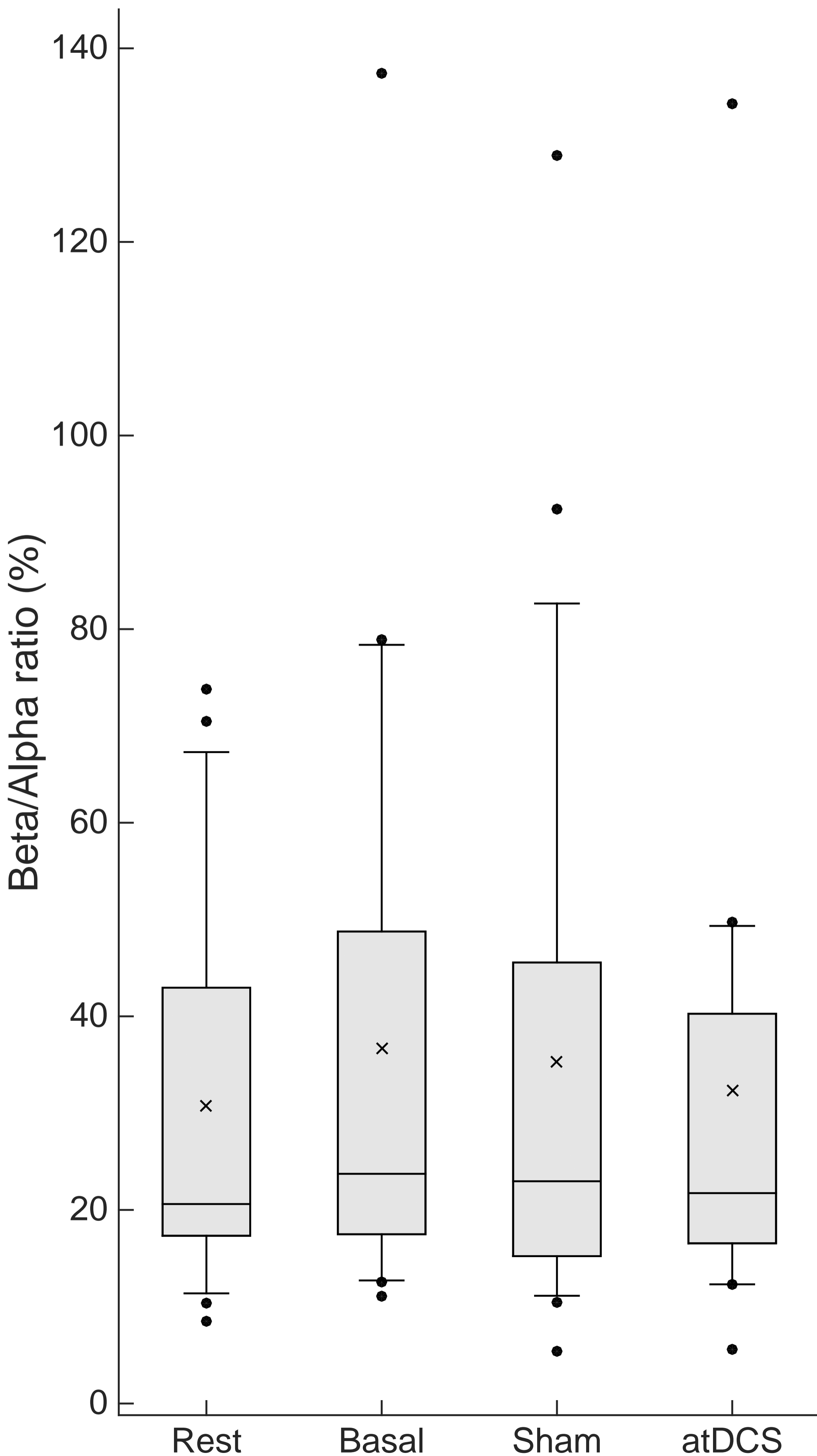

Supplement: Supplementary file 1 [file Data_Sheet_1.zip › Complementary_results/Band_ratios_average_PSD_windows/Beta_Alpha/Beta-Alpha_mean-win_O2.pdf]

**Beta/Alpha ratio on average  
PSD windows for electrode: P7**

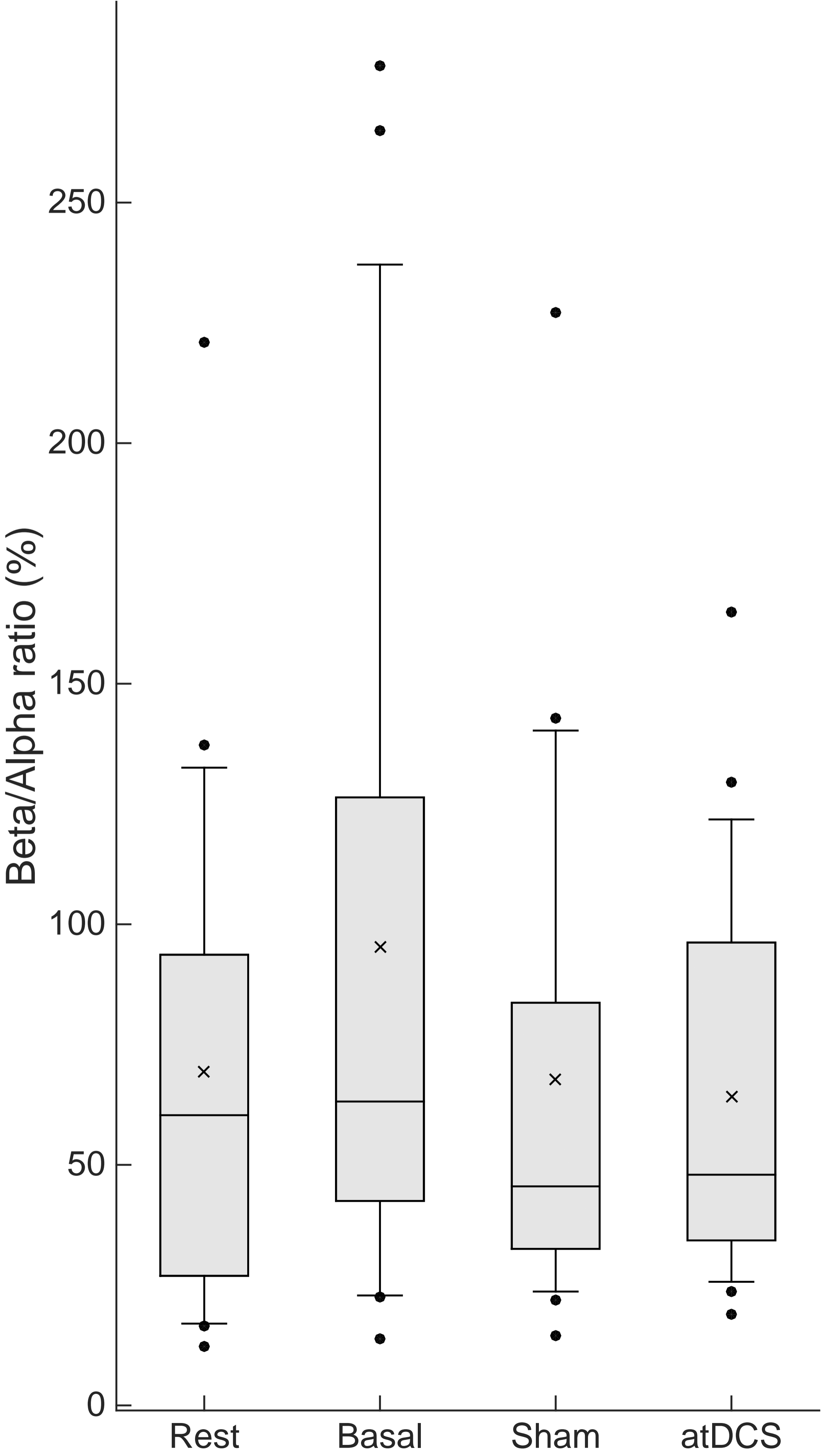

Supplement: Supplementary file 1 [file Data_Sheet_1.zip › Complementary_results/Band_ratios_average_PSD_windows/Beta_Alpha/Beta-Alpha_mean-win_P7.pdf]

**Beta/Alpha ratio on average  
PSD windows for electrode: P8**

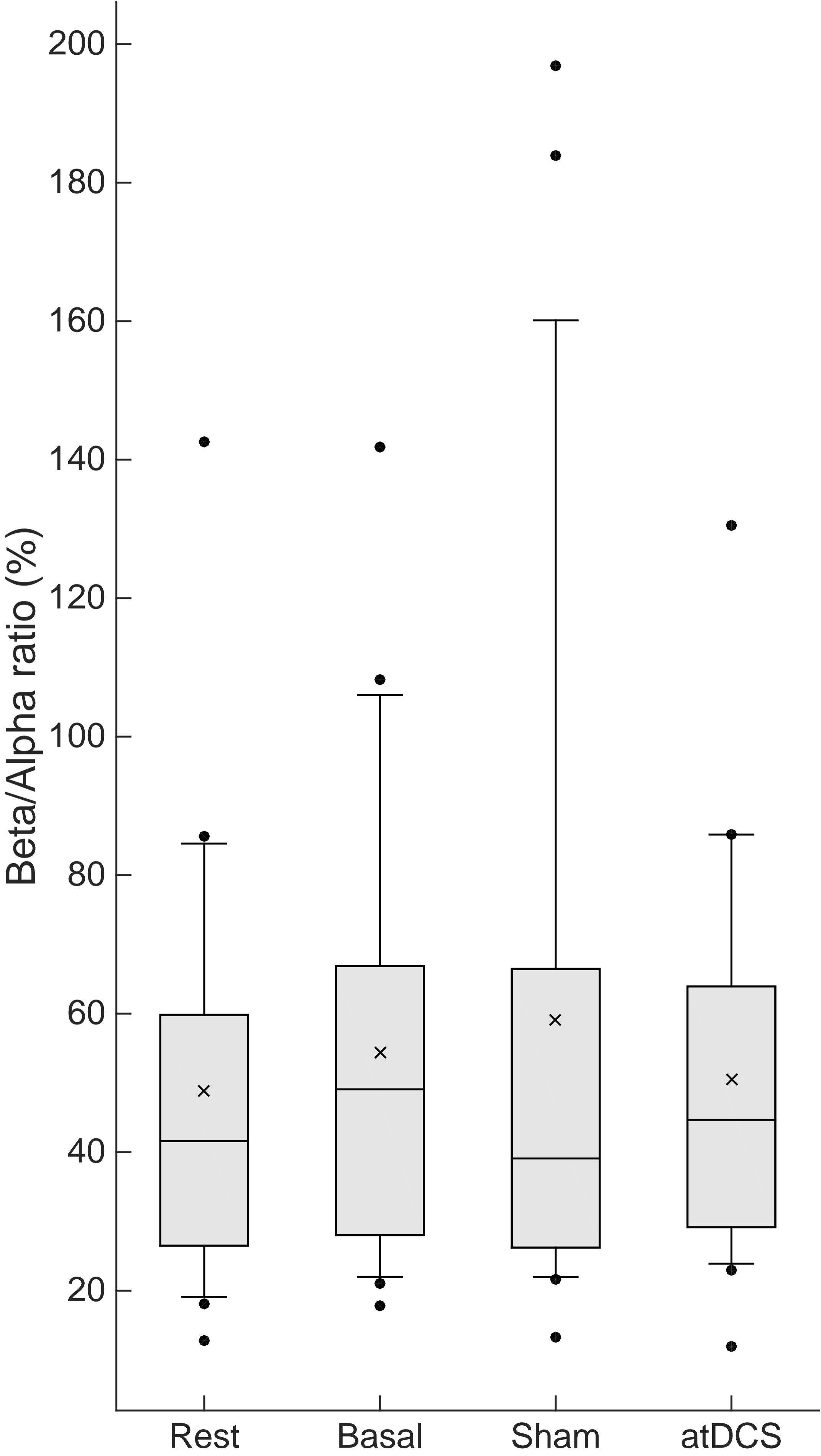

Supplement: Supplementary file 1 [file Data_Sheet_1.zip › Complementary_results/Band_ratios_average_PSD_windows/Beta_Alpha/Beta-Alpha_mean-win_P8.pdf]

**Beta/Alpha ratio on average  
PSD windows for electrode: T7**

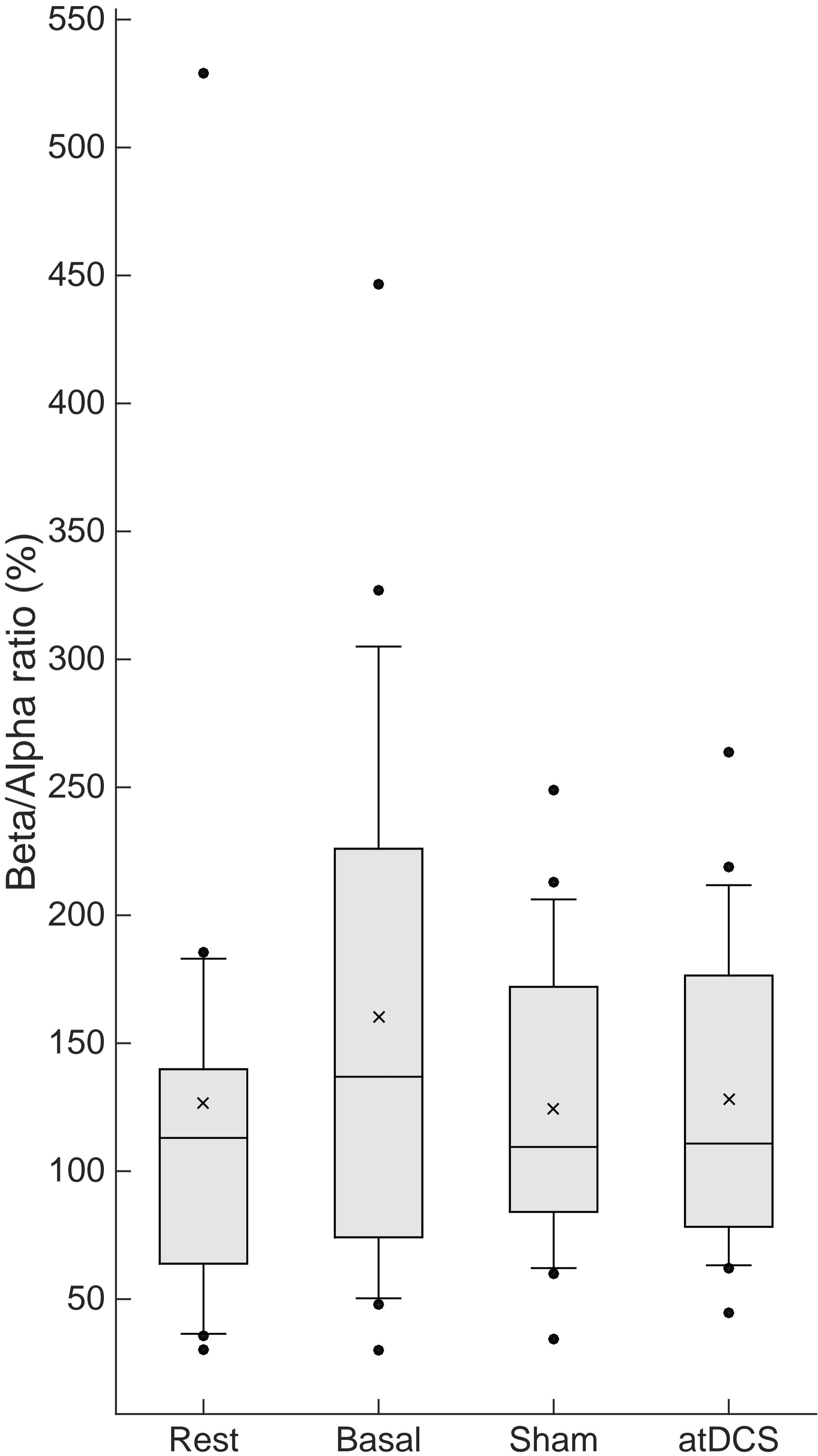

Supplement: Supplementary file 1 [file Data_Sheet_1.zip › Complementary_results/Band_ratios_average_PSD_windows/Beta_Alpha/Beta-Alpha_mean-win_T7.pdf]

**Beta/Alpha ratio on average  
PSD windows for electrode: T8**

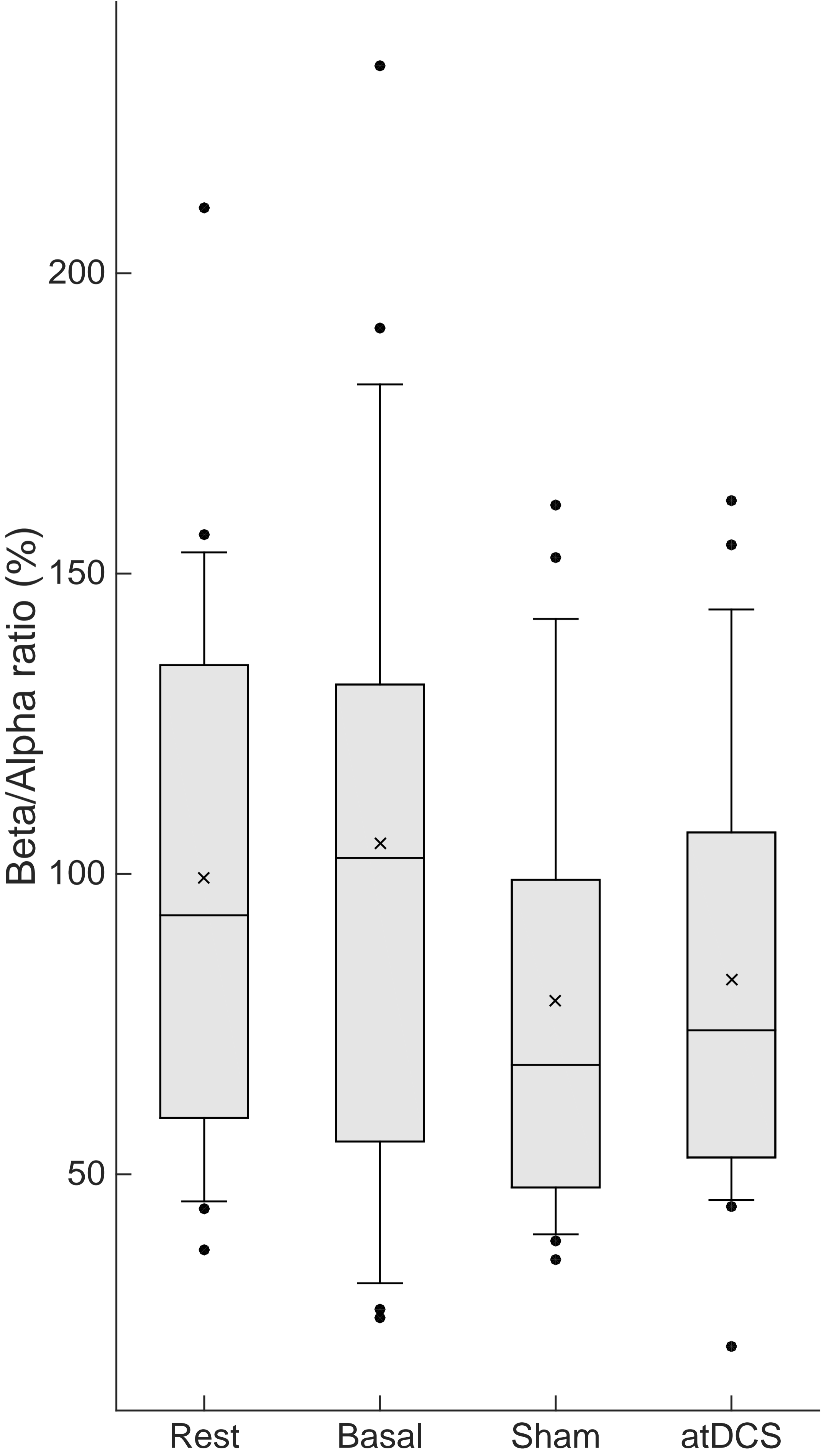

Supplement: Supplementary file 1 [file Data_Sheet_1.zip › Complementary_results/Band_ratios_average_PSD_windows/Beta_Alpha/Beta-Alpha_mean-win_T8.pdf]

**Beta/Delta ratio on average  
PSD windows for electrode: AF3**

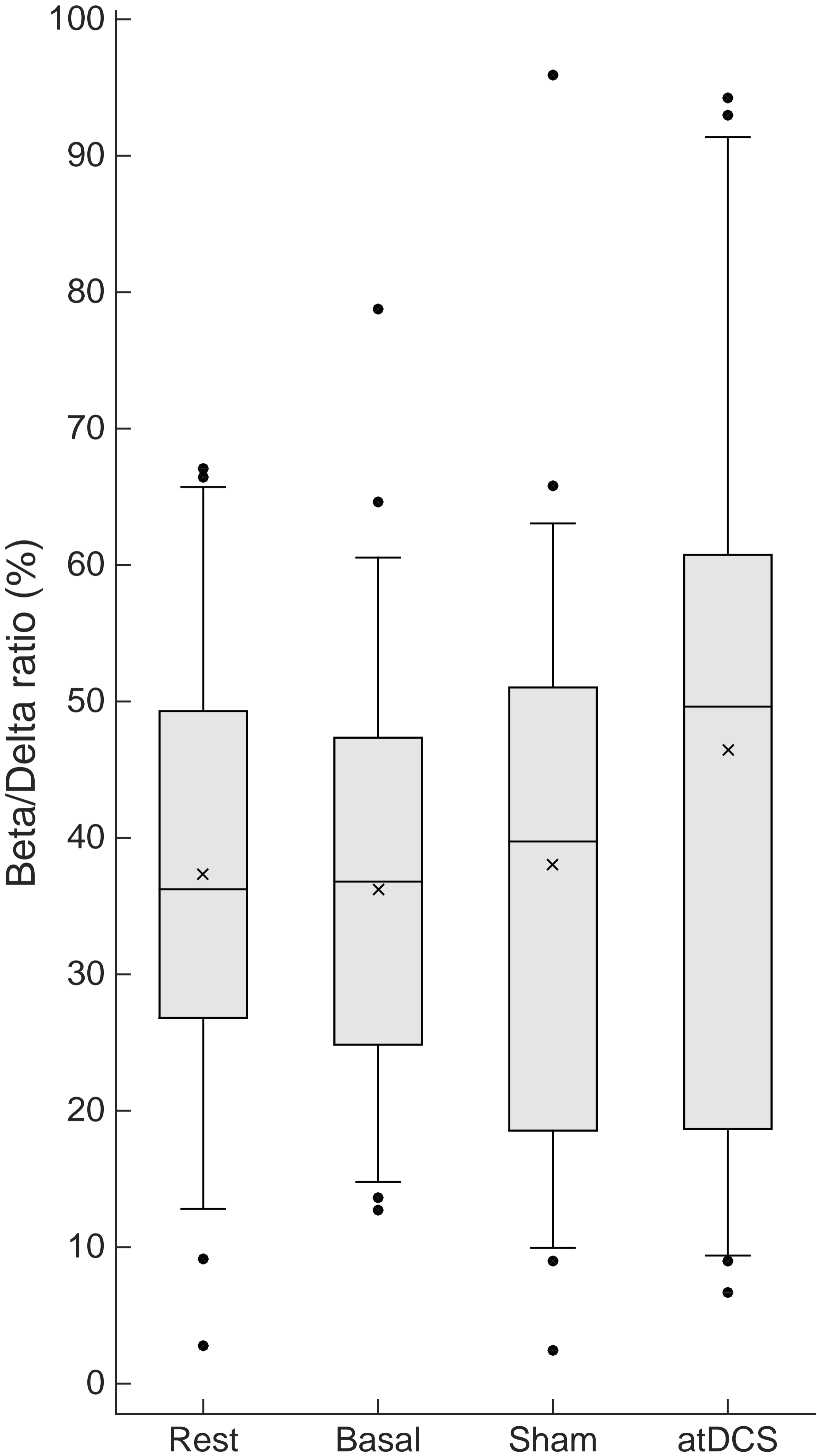

Supplement: Supplementary file 1 [file Data_Sheet_1.zip › Complementary_results/Band_ratios_average_PSD_windows/Beta_Delta/Beta-Delta_mean-win_AF3.pdf]

**Beta/Delta ratio on average  
PSD windows for electrode: AF4**

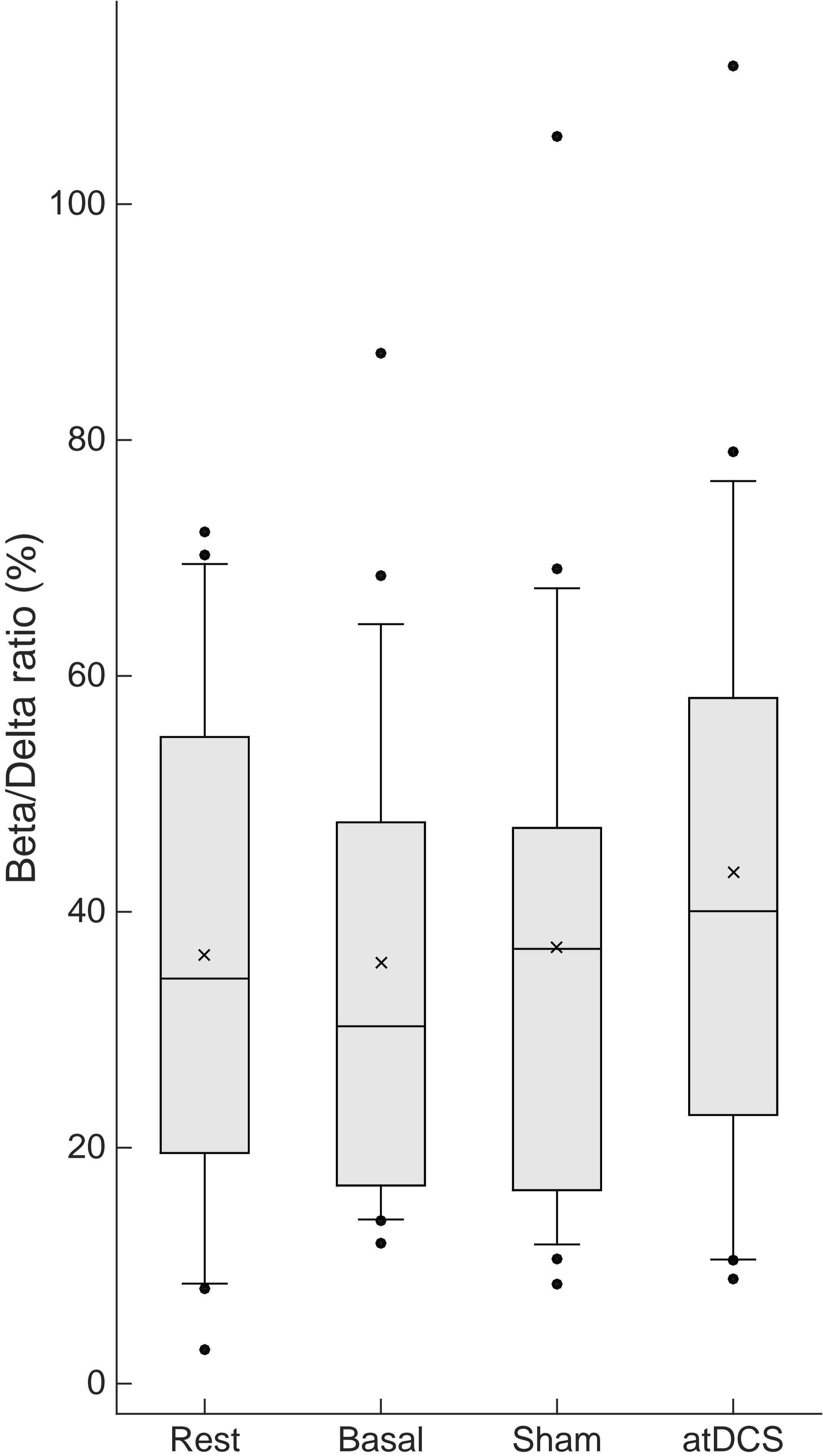

Supplement: Supplementary file 1 [file Data_Sheet_1.zip › Complementary_results/Band_ratios_average_PSD_windows/Beta_Delta/Beta-Delta_mean-win_AF4.pdf]

**Beta/Delta ratio on average**  
**PSD windows for electrode: Avg AF3-F3-F7**

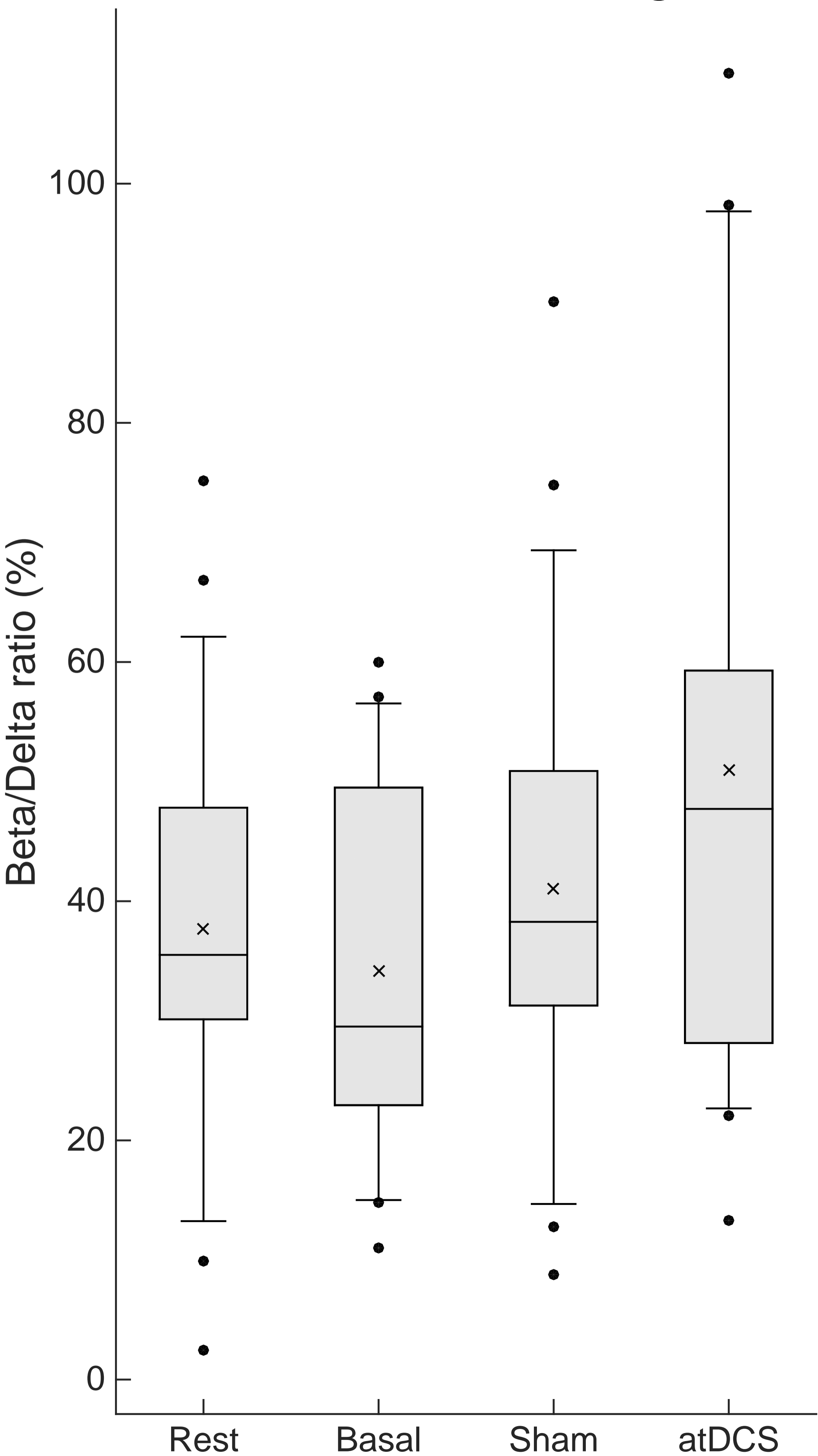

Supplement: Supplementary file 1 [file Data_Sheet_1.zip › Complementary_results/Band_ratios_average_PSD_windows/Beta_Delta/Beta-Delta_mean-win_Avg AF3-F3-F7.pdf]

**Beta/Delta ratio on average**  
**PSD windows for electrode: Avg AF4-F4-F8**

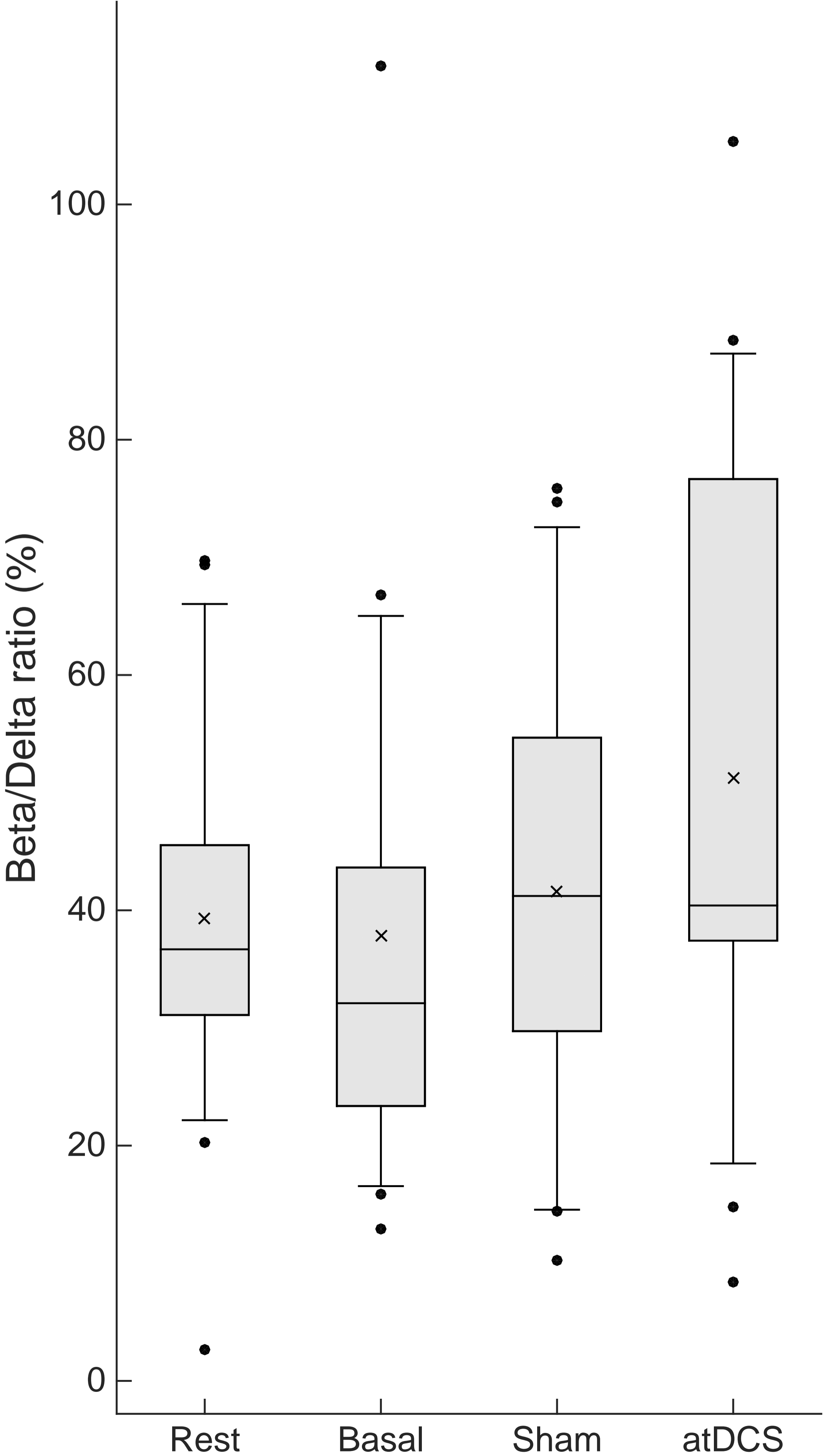

Supplement: Supplementary file 1 [file Data_Sheet_1.zip › Complementary_results/Band_ratios_average_PSD_windows/Beta_Delta/Beta-Delta_mean-win_Avg AF4-F4-F8.pdf]

**Beta/Delta ratio on average**  
**PSD windows for electrode: Avg F3-F7-FC5**

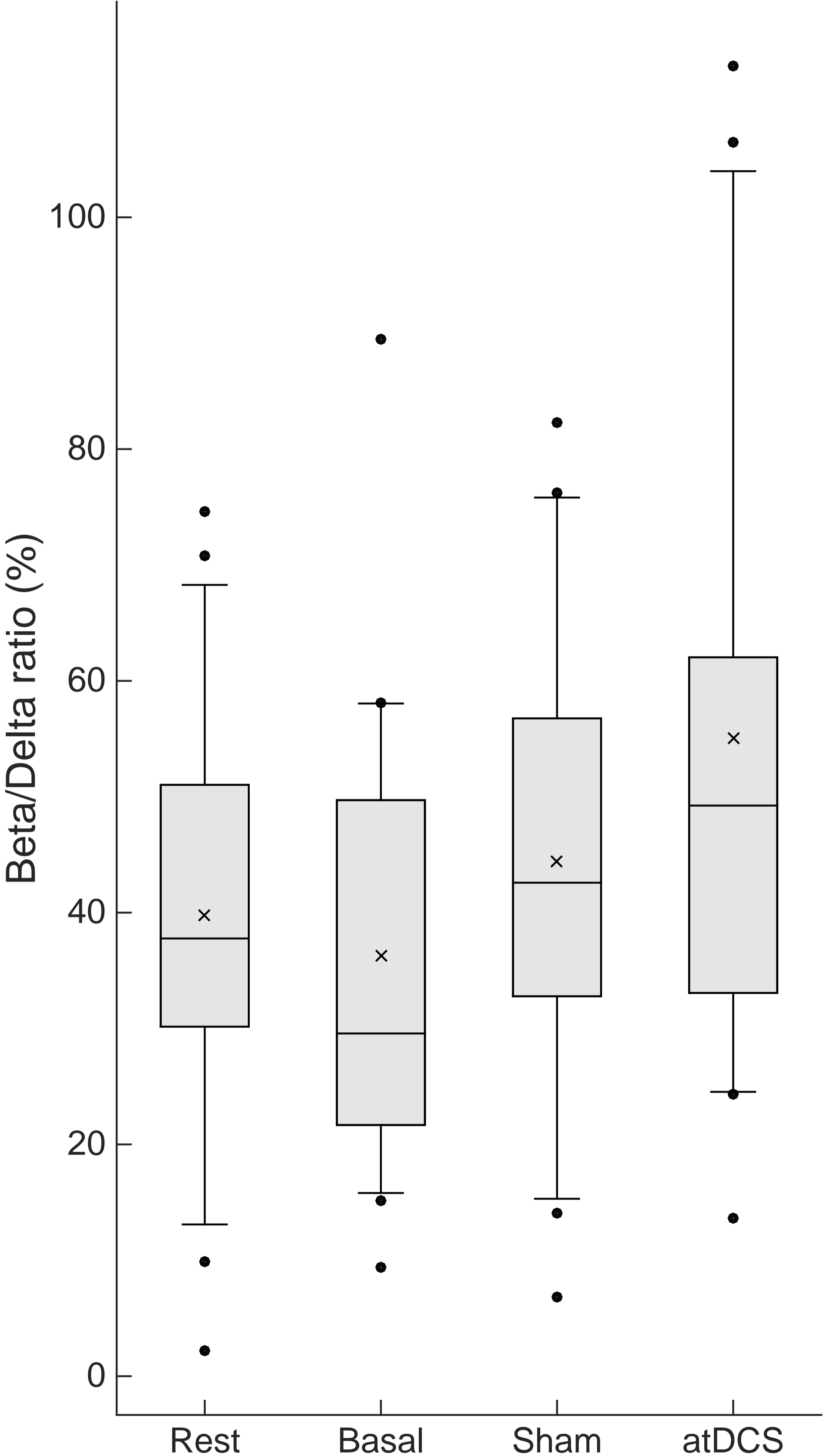

Supplement: Supplementary file 1 [file Data_Sheet_1.zip › Complementary_results/Band_ratios_average_PSD_windows/Beta_Delta/Beta-Delta_mean-win_Avg F3-F7-FC5.pdf]

**Beta/Delta ratio on average**  
**PSD windows for electrode: Avg F4-F8-FC6**

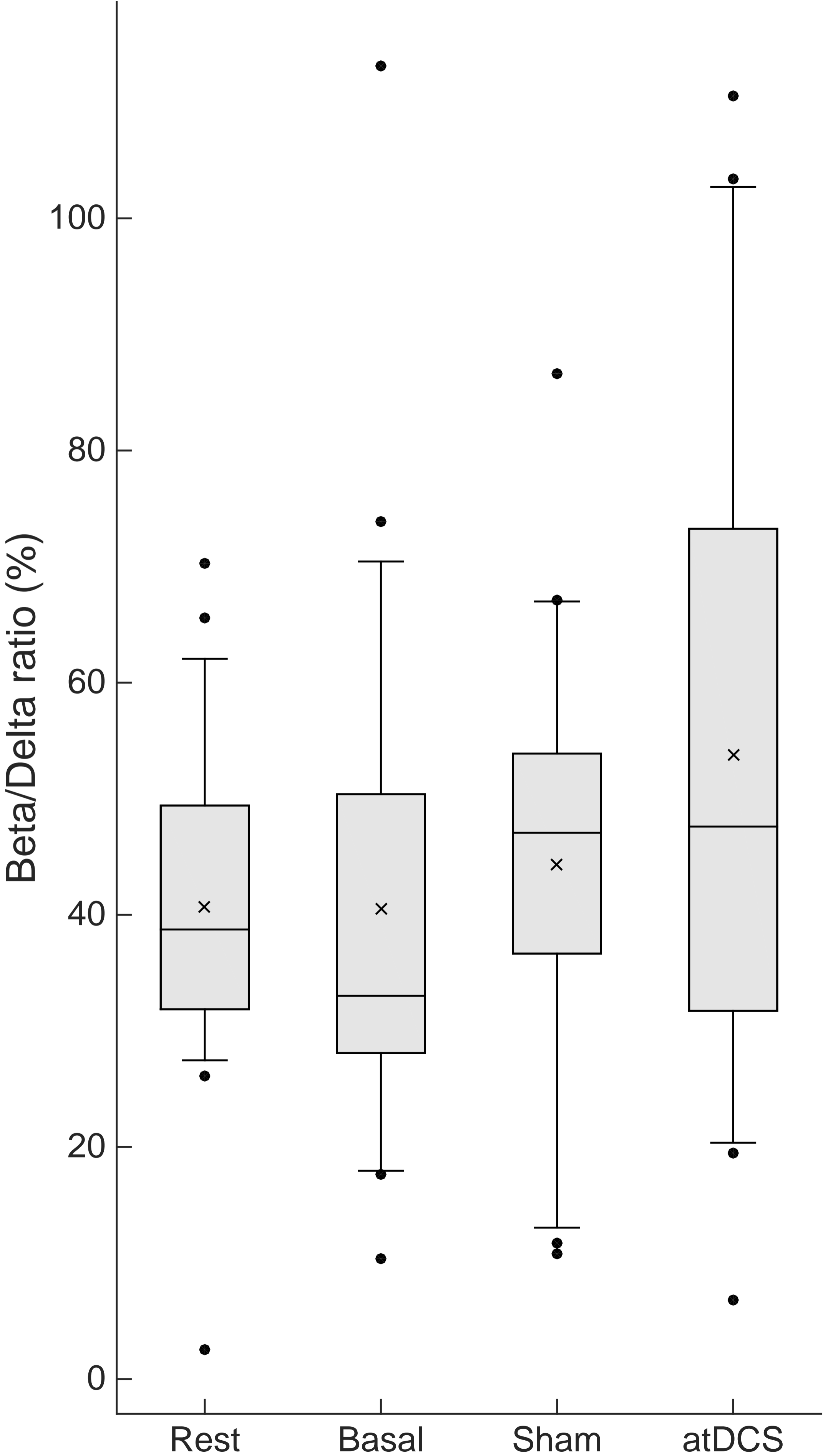

Supplement: Supplementary file 1 [file Data_Sheet_1.zip › Complementary_results/Band_ratios_average_PSD_windows/Beta_Delta/Beta-Delta_mean-win_Avg F4-F8-FC6.pdf]

**Beta/Delta ratio on average  
PSD windows for electrode: F3**

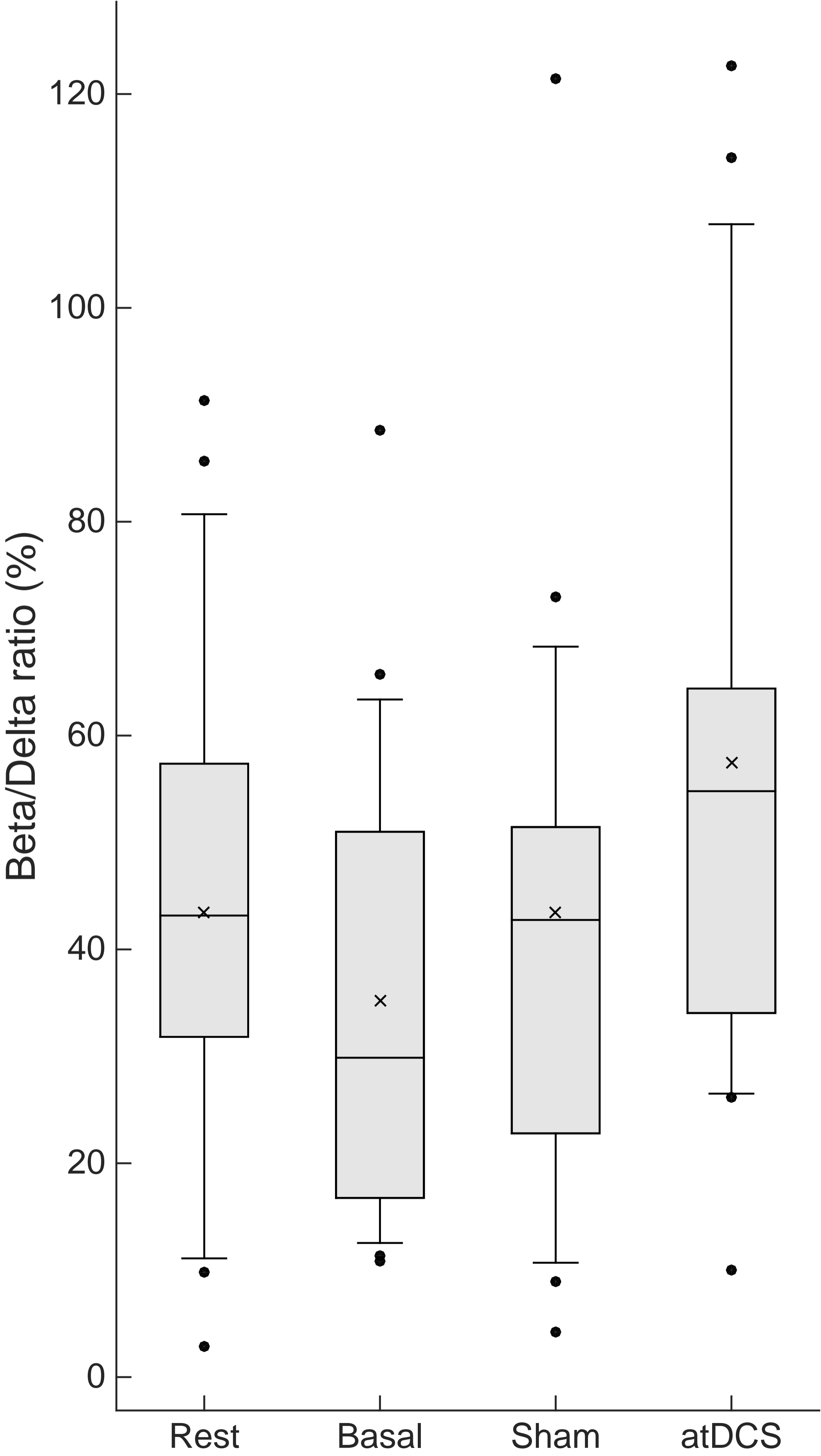

Supplement: Supplementary file 1 [file Data_Sheet_1.zip › Complementary_results/Band_ratios_average_PSD_windows/Beta_Delta/Beta-Delta_mean-win_F3.pdf]

**Beta/Delta ratio on average  
PSD windows for electrode: F4**

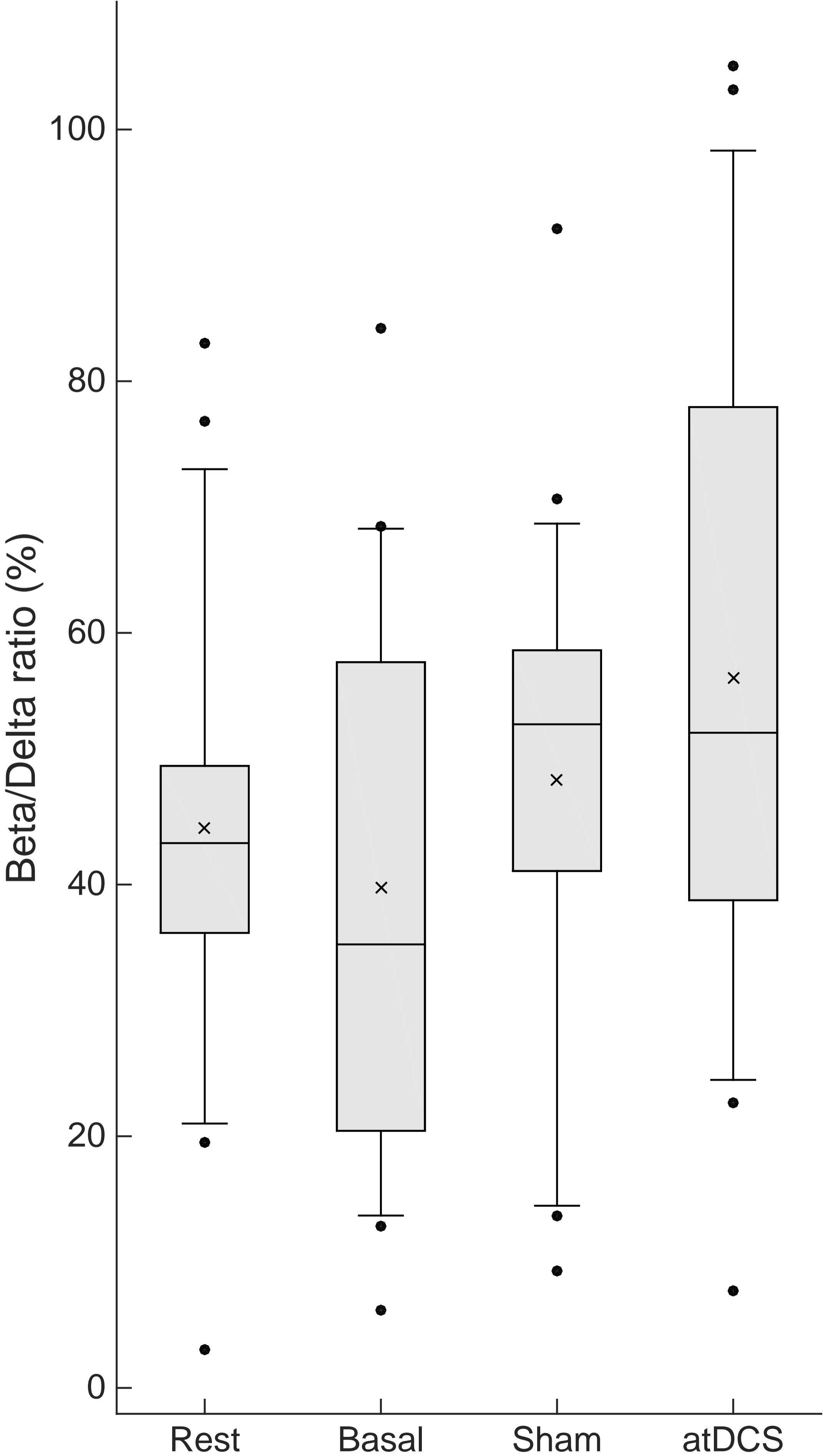

Supplement: Supplementary file 1 [file Data_Sheet_1.zip › Complementary_results/Band_ratios_average_PSD_windows/Beta_Delta/Beta-Delta_mean-win_F4.pdf]

**Beta/Delta ratio on average  
PSD windows for electrode: F7**

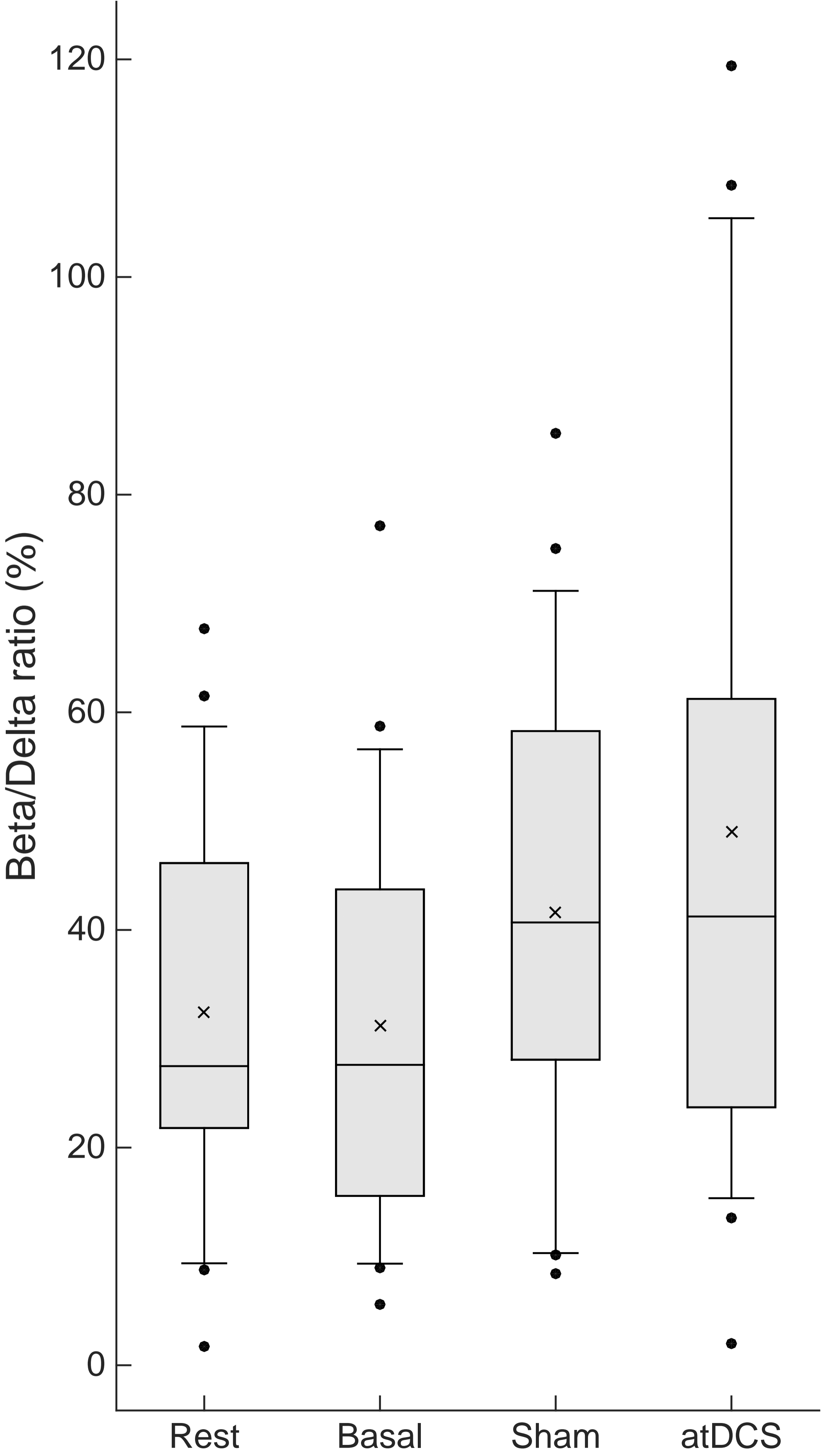

Supplement: Supplementary file 1 [file Data_Sheet_1.zip › Complementary_results/Band_ratios_average_PSD_windows/Beta_Delta/Beta-Delta_mean-win_F7.pdf]

**Beta/Delta ratio on average  
PSD windows for electrode: F8**

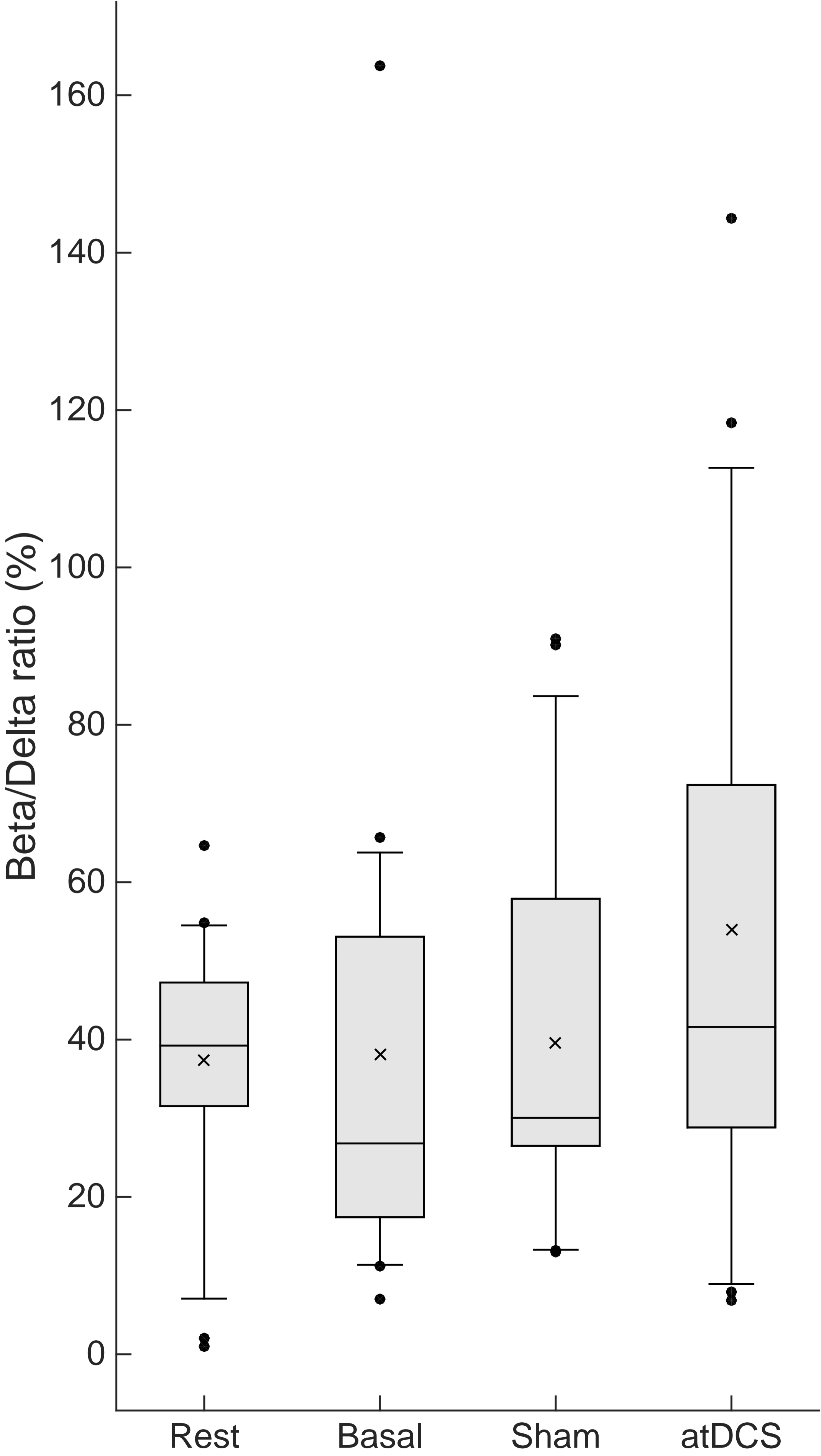

Supplement: Supplementary file 1 [file Data_Sheet_1.zip › Complementary_results/Band_ratios_average_PSD_windows/Beta_Delta/Beta-Delta_mean-win_F8.pdf]

**Beta/Delta ratio on average  
PSD windows for electrode: FC5**

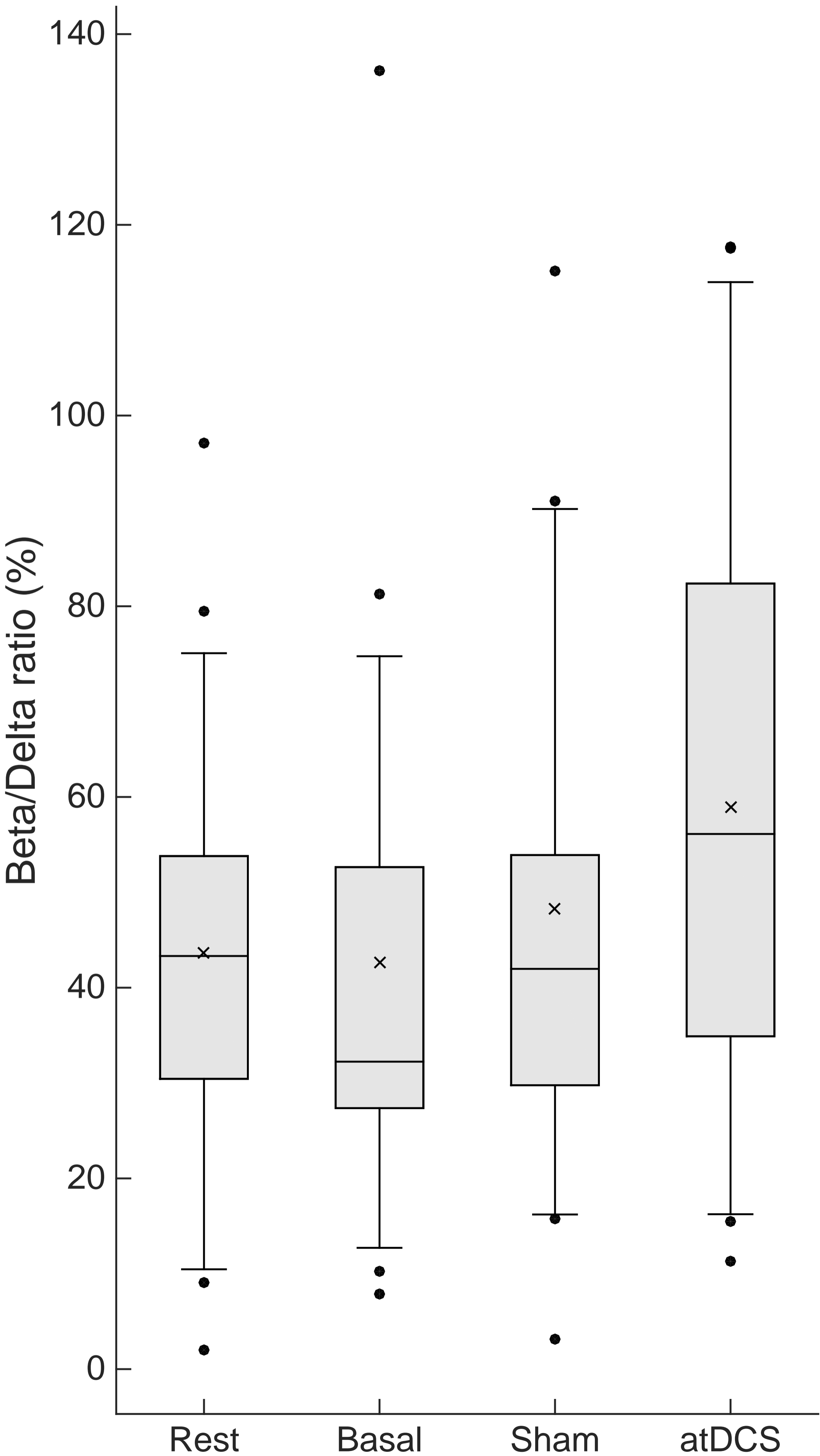

Supplement: Supplementary file 1 [file Data_Sheet_1.zip › Complementary_results/Band_ratios_average_PSD_windows/Beta_Delta/Beta-Delta_mean-win_FC5.pdf]

**Beta/Delta ratio on average  
PSD windows for electrode: FC6**

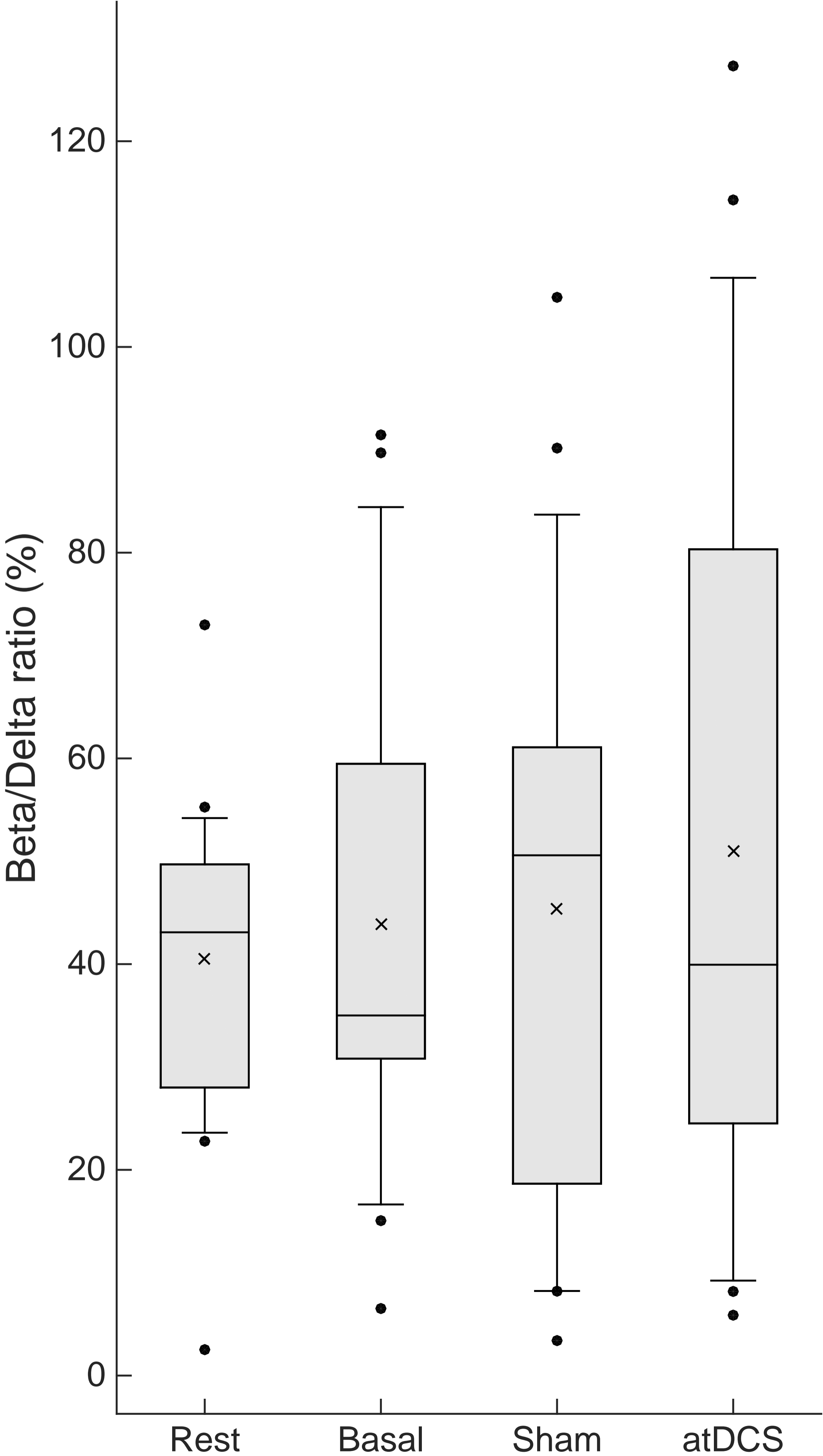

Supplement: Supplementary file 1 [file Data_Sheet_1.zip › Complementary_results/Band_ratios_average_PSD_windows/Beta_Delta/Beta-Delta_mean-win_FC6.pdf]

# Beta/Delta ratio on average PSD windows for electrode: O1

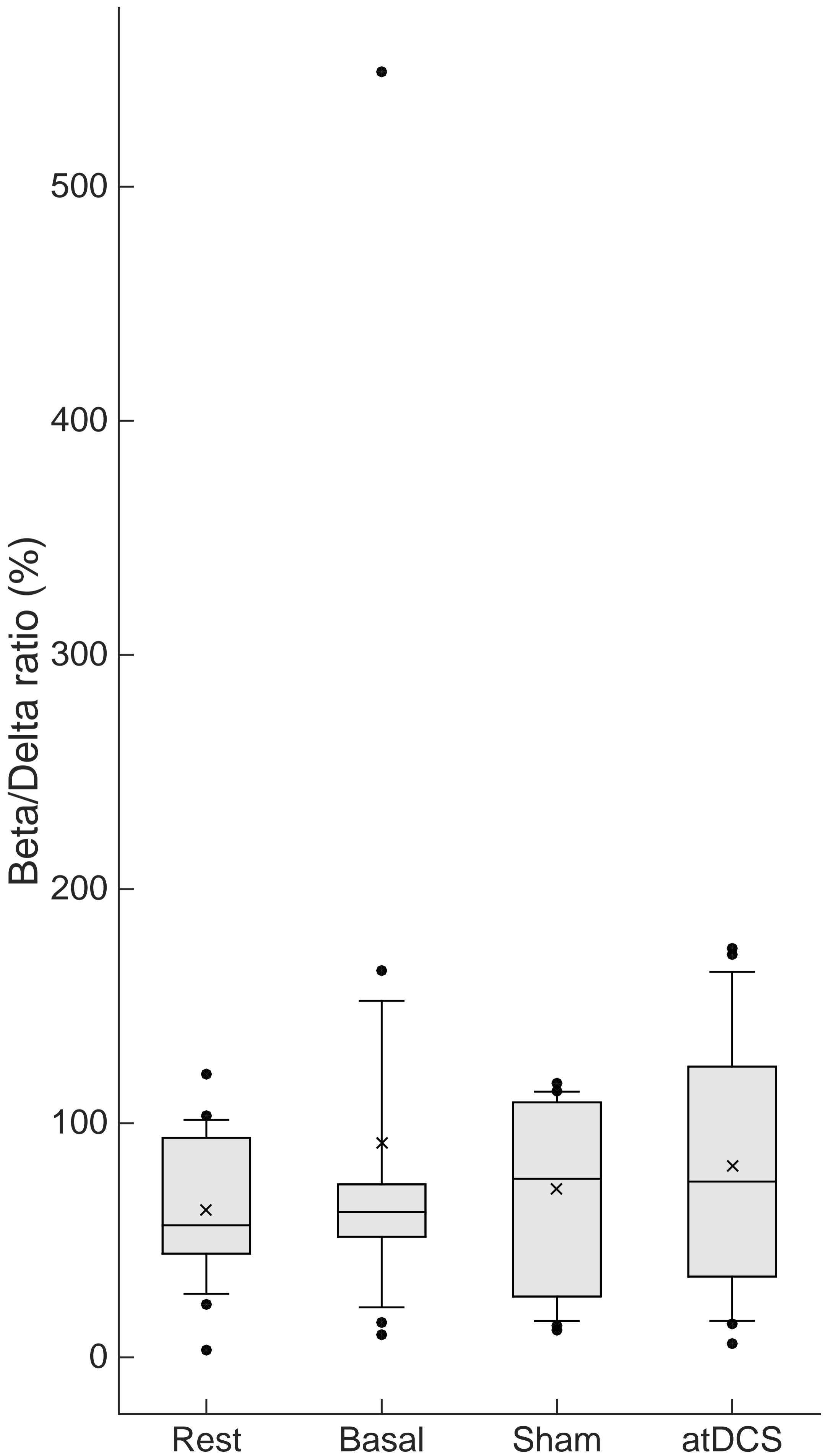

Supplement: Supplementary file 1 [file Data_Sheet_1.zip › Complementary_results/Band_ratios_average_PSD_windows/Beta_Delta/Beta-Delta_mean-win_O1.pdf]

**Beta/Delta ratio on average  
PSD windows for electrode: O2**

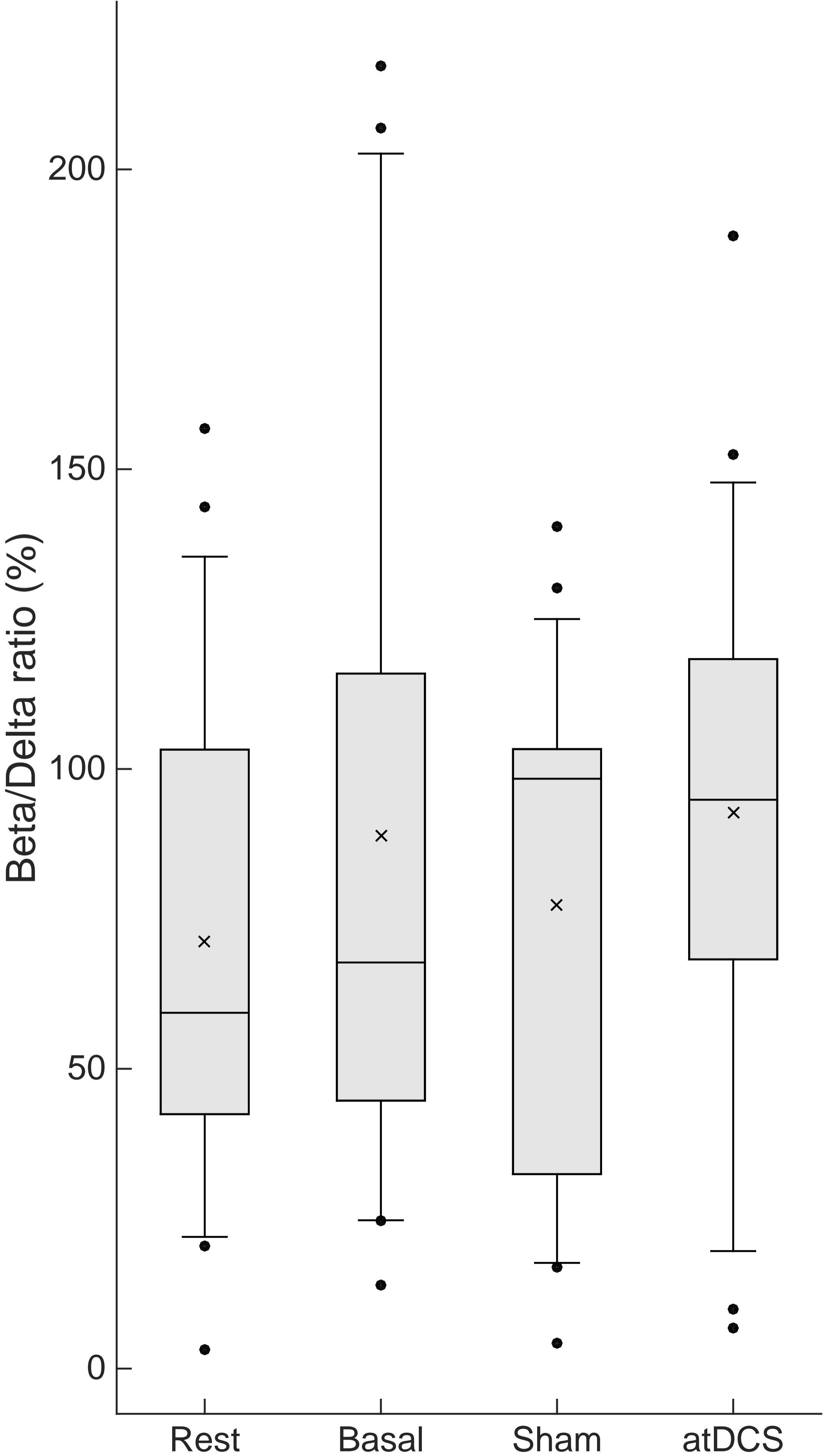

Supplement: Supplementary file 1 [file Data_Sheet_1.zip › Complementary_results/Band_ratios_average_PSD_windows/Beta_Delta/Beta-Delta_mean-win_O2.pdf]

**Beta/Delta ratio on average  
PSD windows for electrode: P7**

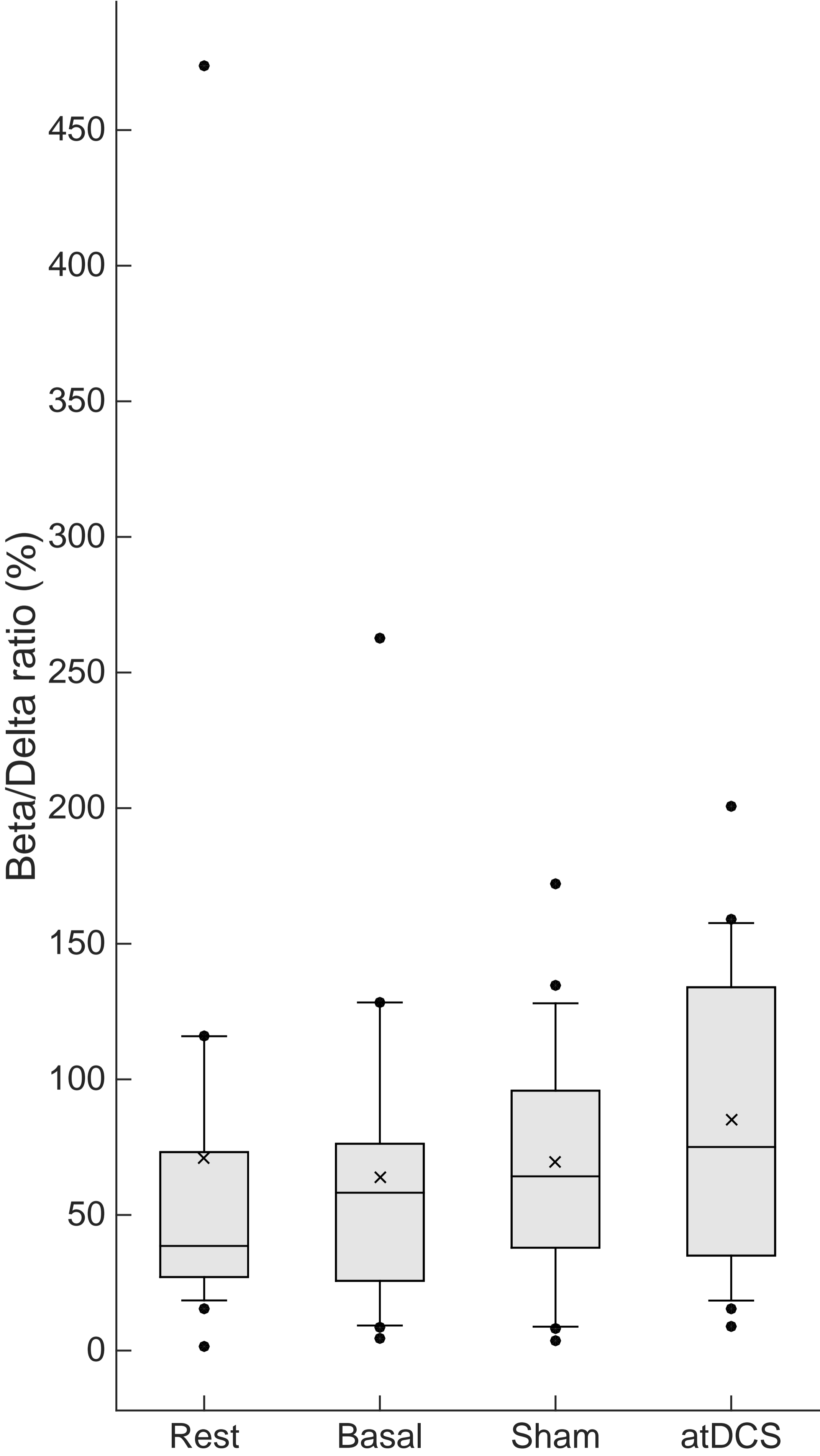

Supplement: Supplementary file 1 [file Data_Sheet_1.zip › Complementary_results/Band_ratios_average_PSD_windows/Beta_Delta/Beta-Delta_mean-win_P7.pdf]

**Beta/Delta ratio on average  
PSD windows for electrode: P8**

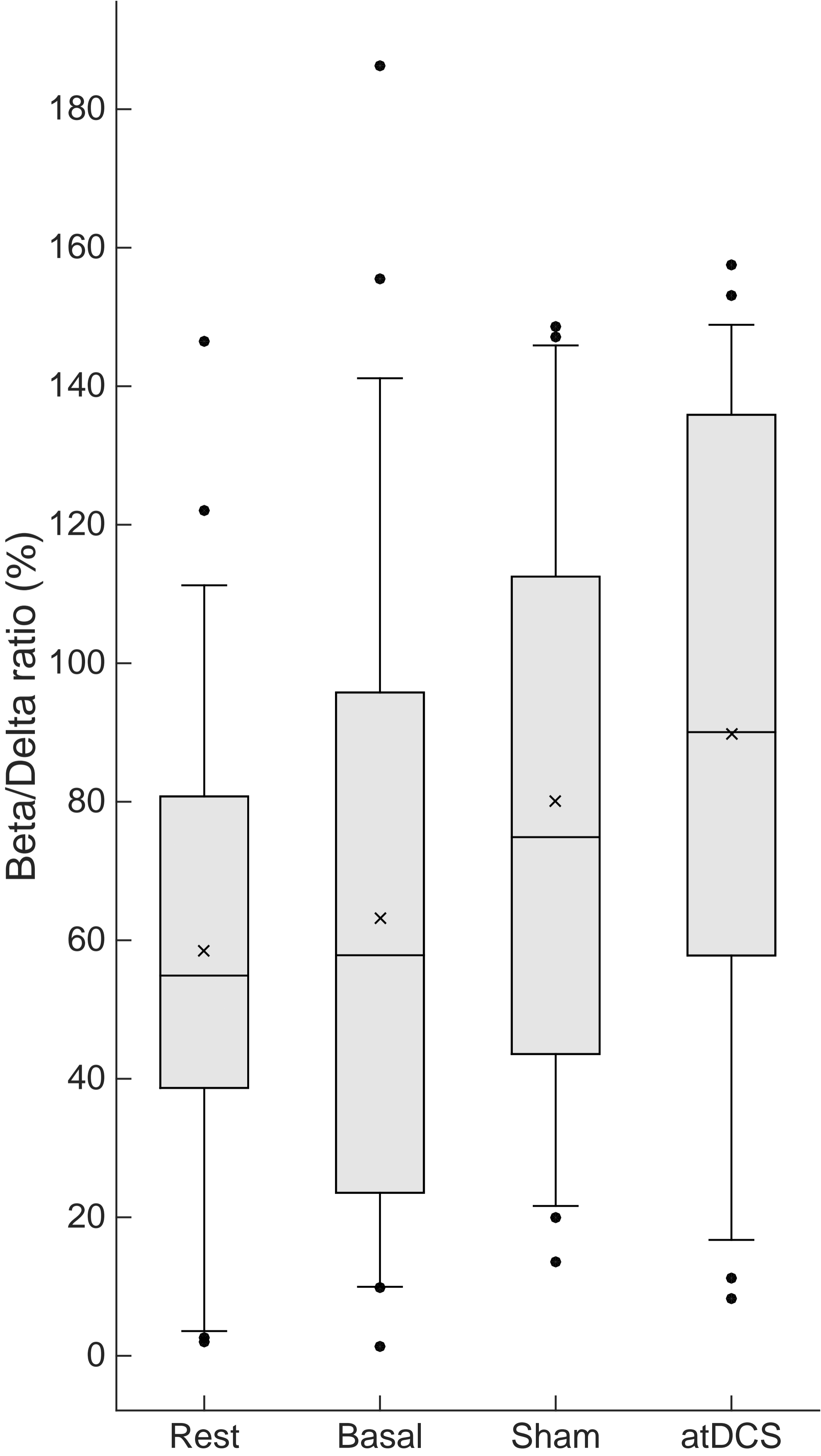

Supplement: Supplementary file 1 [file Data_Sheet_1.zip › Complementary_results/Band_ratios_average_PSD_windows/Beta_Delta/Beta-Delta_mean-win_P8.pdf]

**Beta/Delta ratio on average  
PSD windows for electrode: T7**

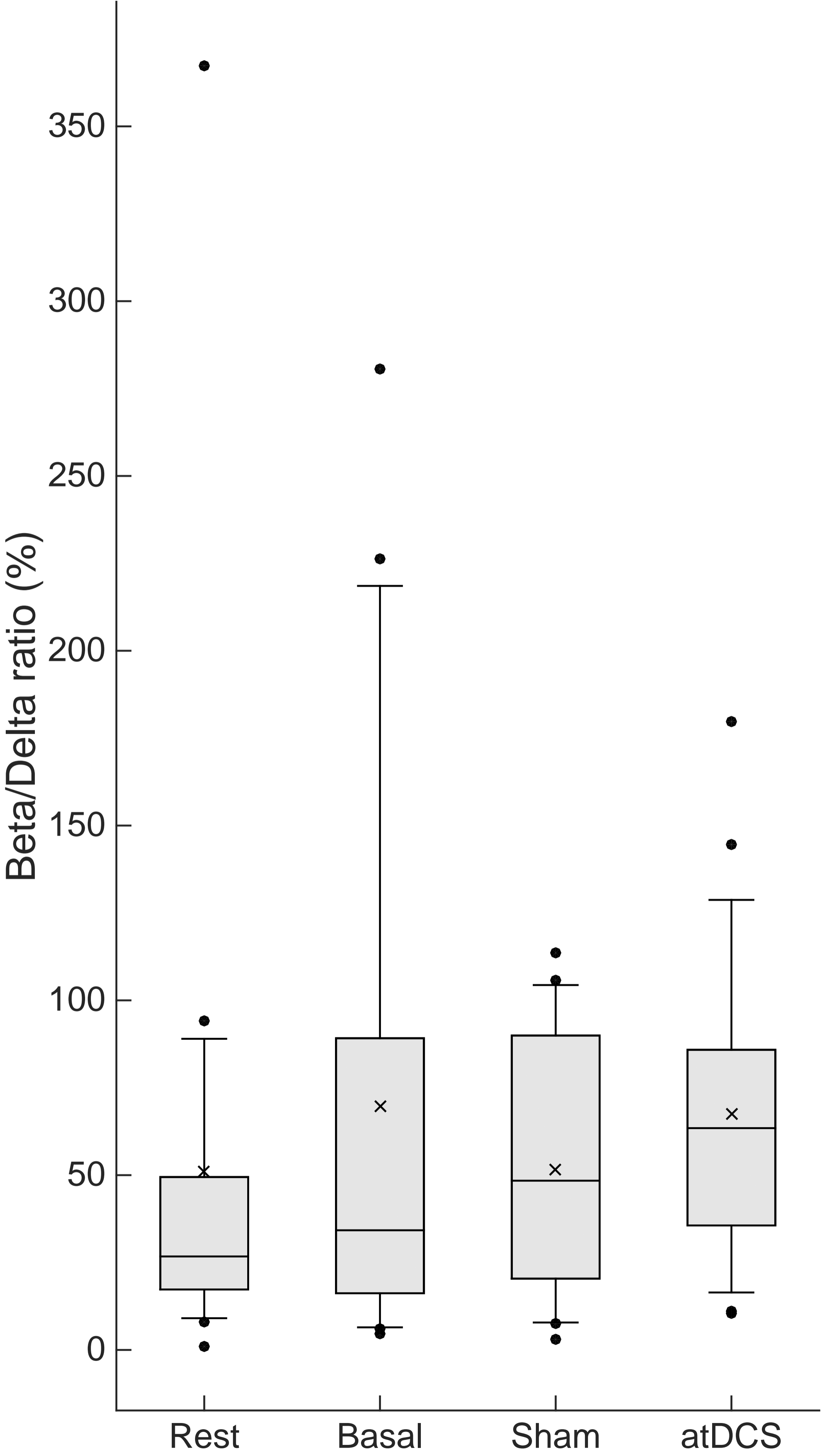

Supplement: Supplementary file 1 [file Data_Sheet_1.zip › Complementary_results/Band_ratios_average_PSD_windows/Beta_Delta/Beta-Delta_mean-win_T7.pdf]

**Beta/Delta ratio on average  
PSD windows for electrode: T8**

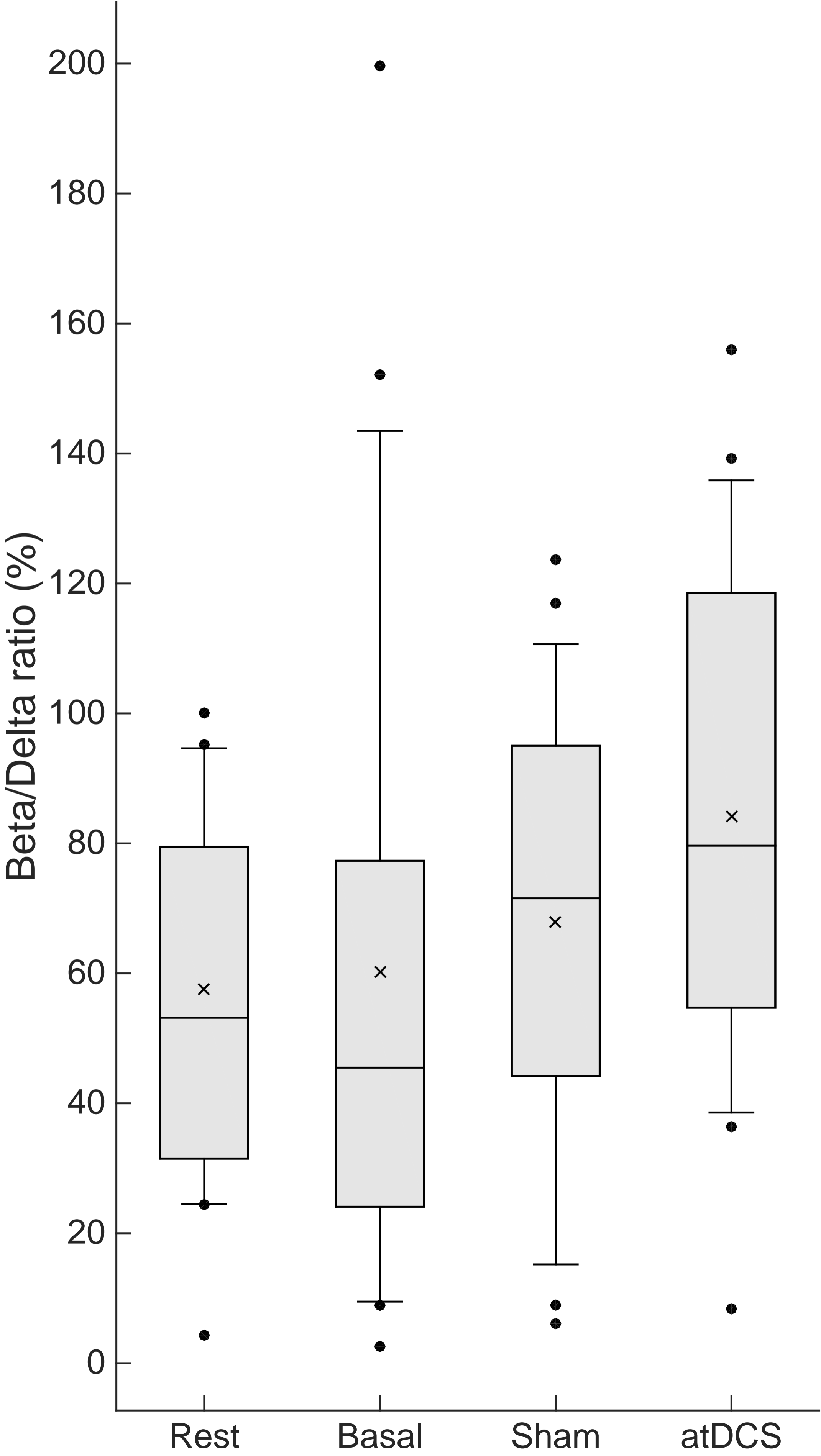

Supplement: Supplementary file 1 [file Data_Sheet_1.zip › Complementary_results/Band_ratios_average_PSD_windows/Beta_Delta/Beta-Delta_mean-win_T8.pdf]

**Gamma/Alpha ratio on average  
PSD windows for electrode: AF3**

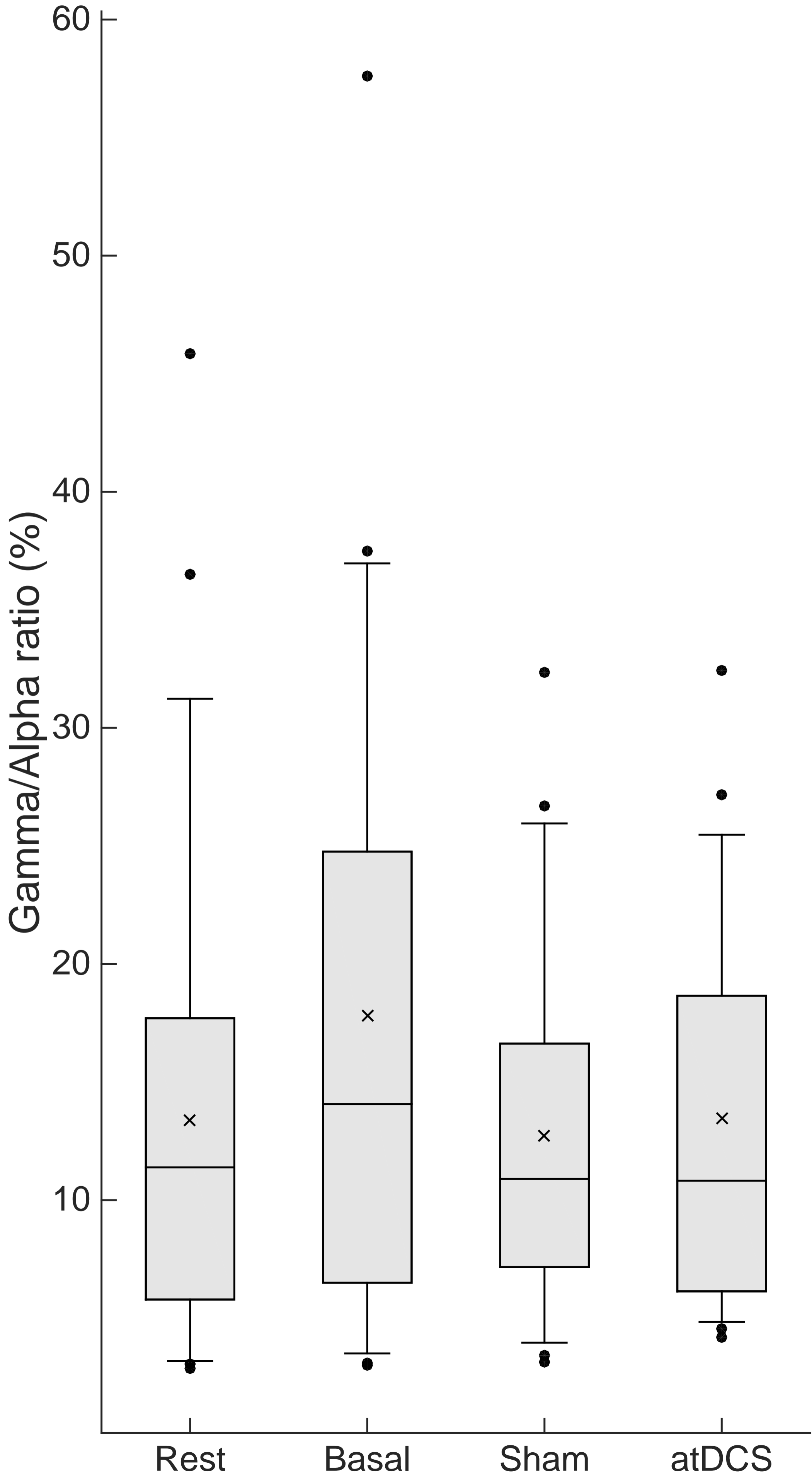

Supplement: Supplementary file 1 [file Data_Sheet_1.zip › Complementary_results/Band_ratios_average_PSD_windows/Gamma_Alpha/Gamma-Alpha_mean-win_AF3.pdf]

**Gamma/Alpha ratio on average  
PSD windows for electrode: AF4**

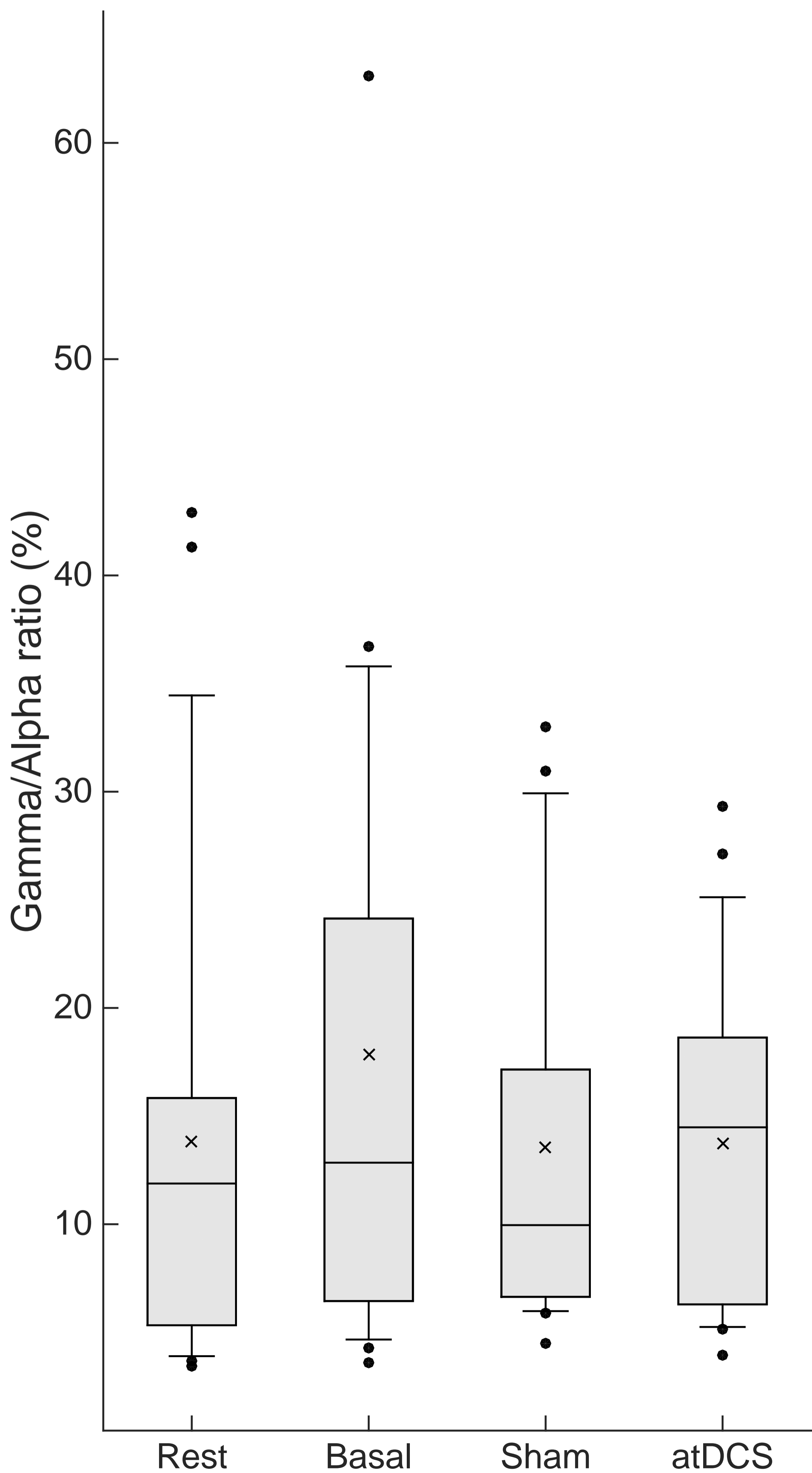

Supplement: Supplementary file 1 [file Data_Sheet_1.zip › Complementary_results/Band_ratios_average_PSD_windows/Gamma_Alpha/Gamma-Alpha_mean-win_AF4.pdf]

**Gamma/Alpha ratio on average  
PSD windows for electrode: Avg AF3-F3-F7**

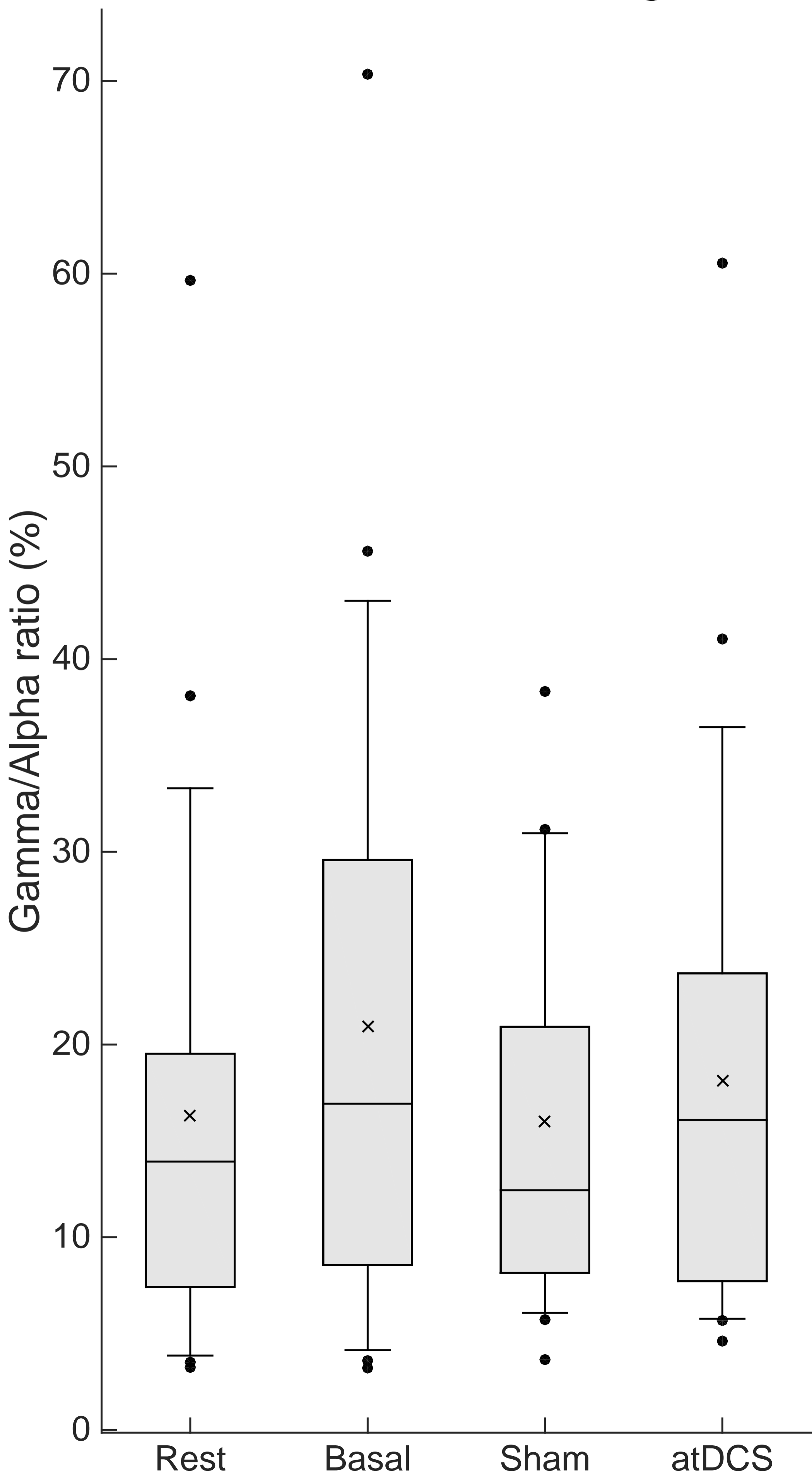

Supplement: Supplementary file 1 [file Data_Sheet_1.zip › Complementary_results/Band_ratios_average_PSD_windows/Gamma_Alpha/Gamma-Alpha_mean-win_Avg AF3-F3-F7.pdf]

**Gamma/Alpha ratio on average**  
**PSD windows for electrode: Avg AF4-F4-F8**

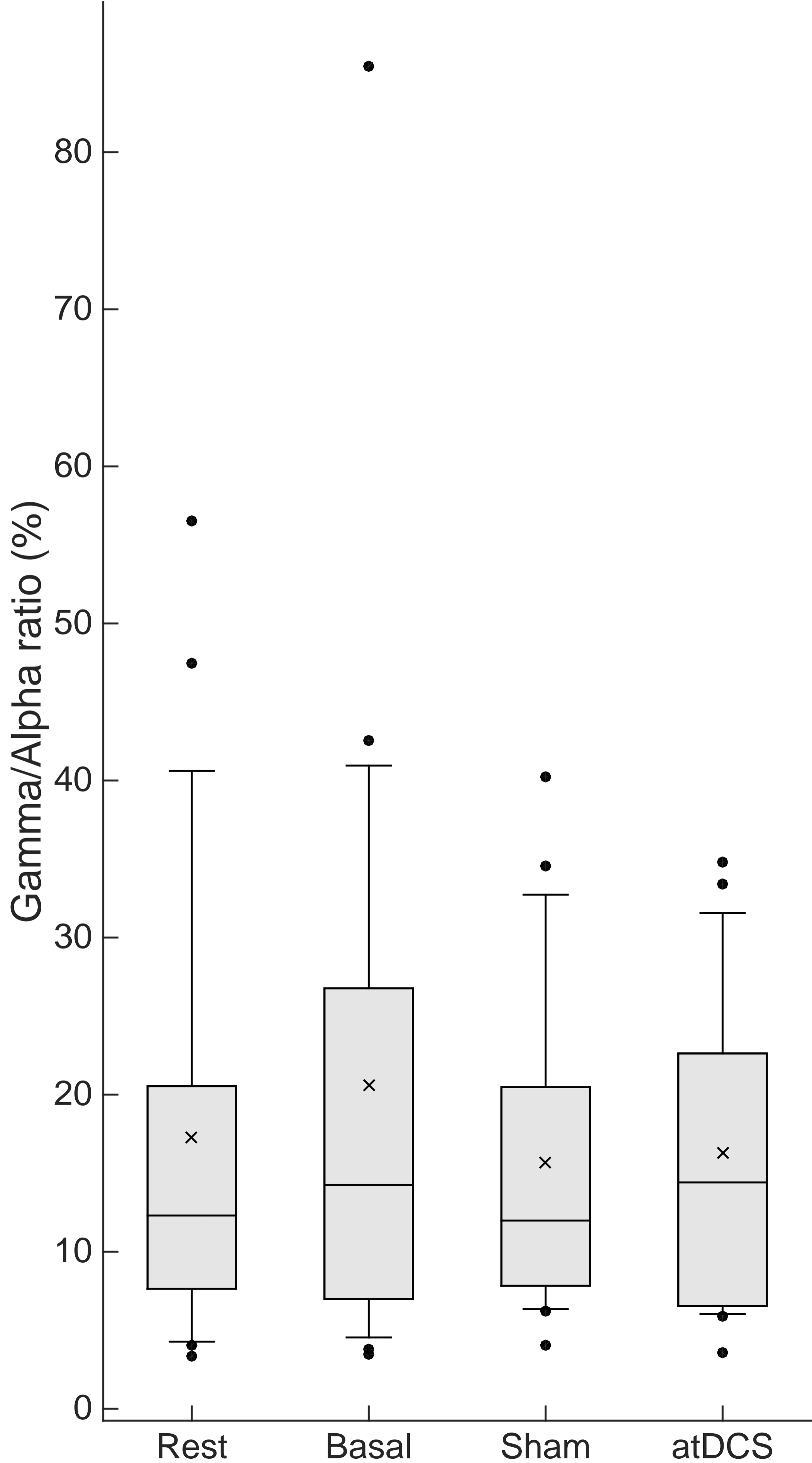

Supplement: Supplementary file 1 [file Data_Sheet_1.zip › Complementary_results/Band_ratios_average_PSD_windows/Gamma_Alpha/Gamma-Alpha_mean-win_Avg AF4-F4-F8.pdf]

**Gamma/Alpha ratio on average**  
**PSD windows for electrode: Avg F3-F7-FC5**

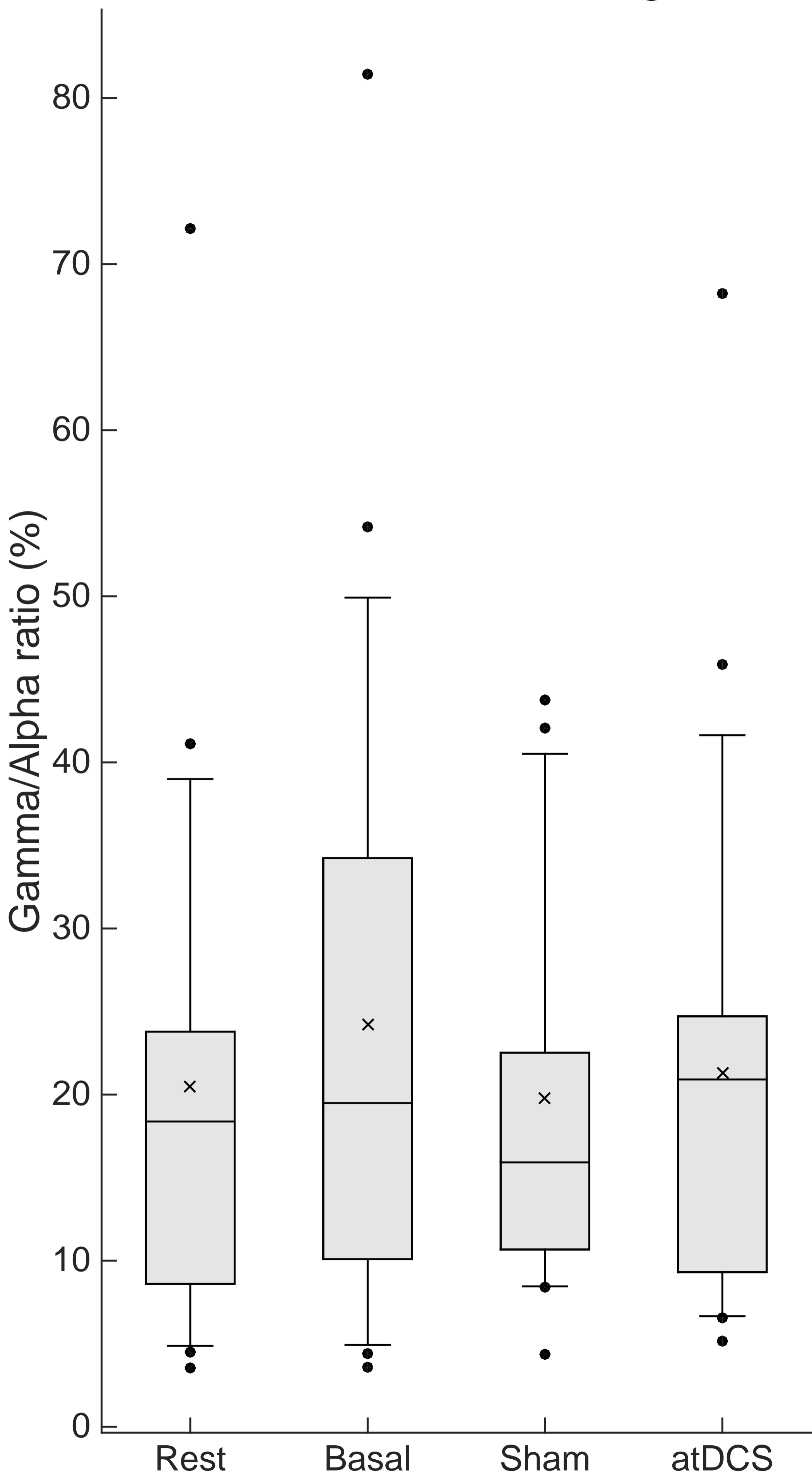

Supplement: Supplementary file 1 [file Data_Sheet_1.zip › Complementary_results/Band_ratios_average_PSD_windows/Gamma_Alpha/Gamma-Alpha_mean-win_Avg F3-F7-FC5.pdf]

**Gamma/Alpha ratio on average**  
**PSD windows for electrode: Avg F4-F8-FC6**

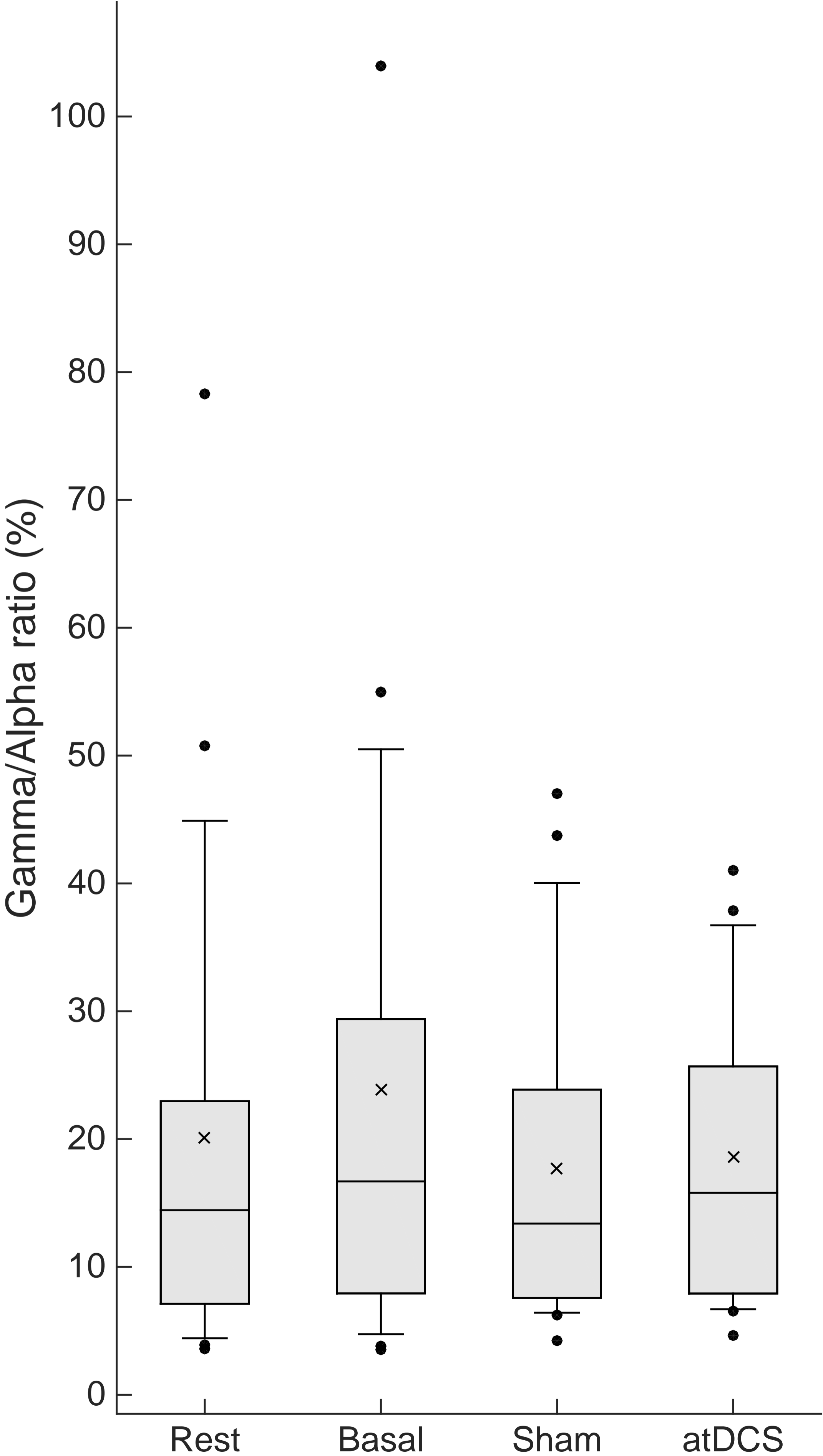

Supplement: Supplementary file 1 [file Data_Sheet_1.zip › Complementary_results/Band_ratios_average_PSD_windows/Gamma_Alpha/Gamma-Alpha_mean-win_Avg F4-F8-FC6.pdf]

**Gamma/Alpha ratio on average  
PSD windows for electrode: F3**

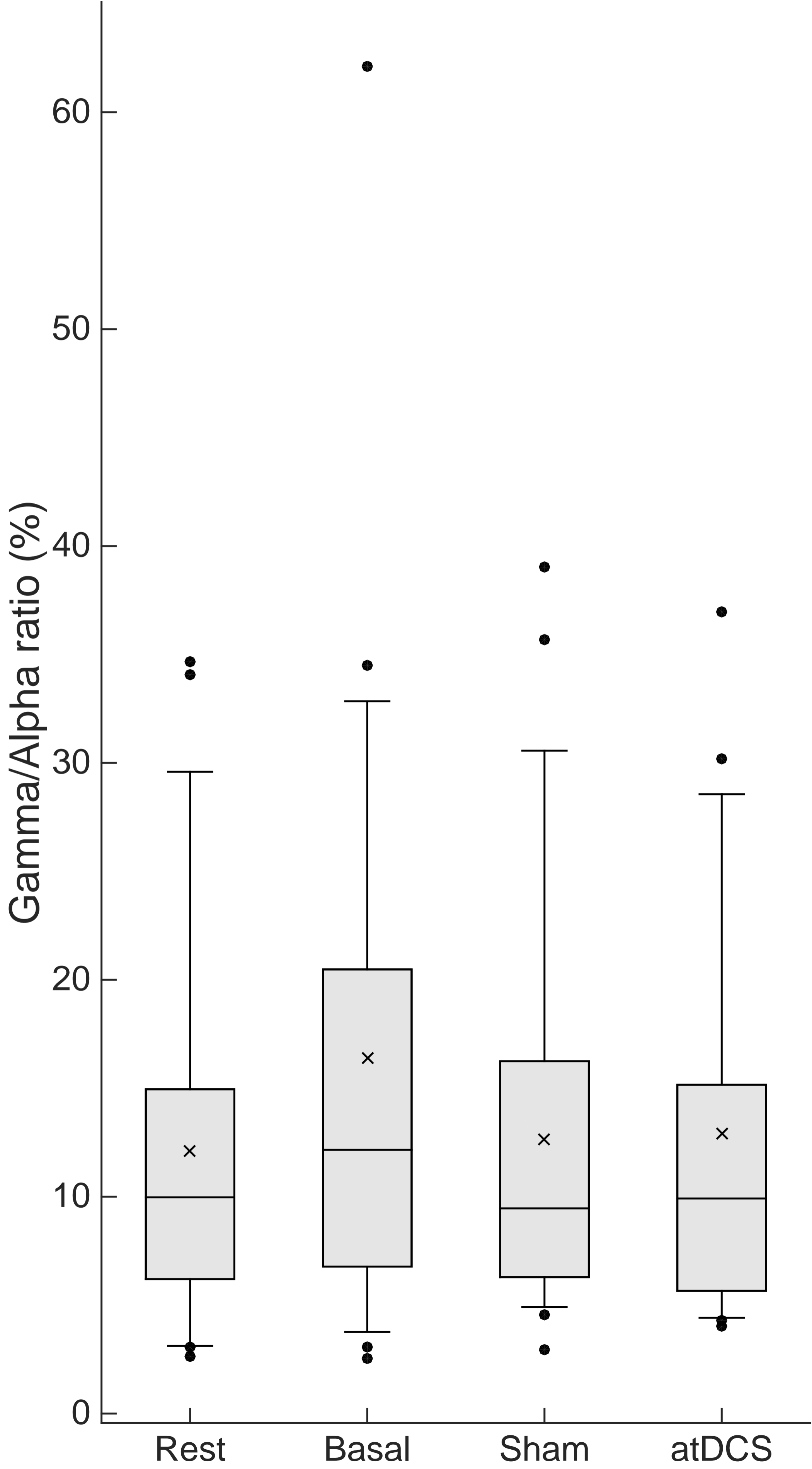

Supplement: Supplementary file 1 [file Data_Sheet_1.zip › Complementary_results/Band_ratios_average_PSD_windows/Gamma_Alpha/Gamma-Alpha_mean-win_F3.pdf]

**Gamma/Alpha ratio on average  
PSD windows for electrode: F4**

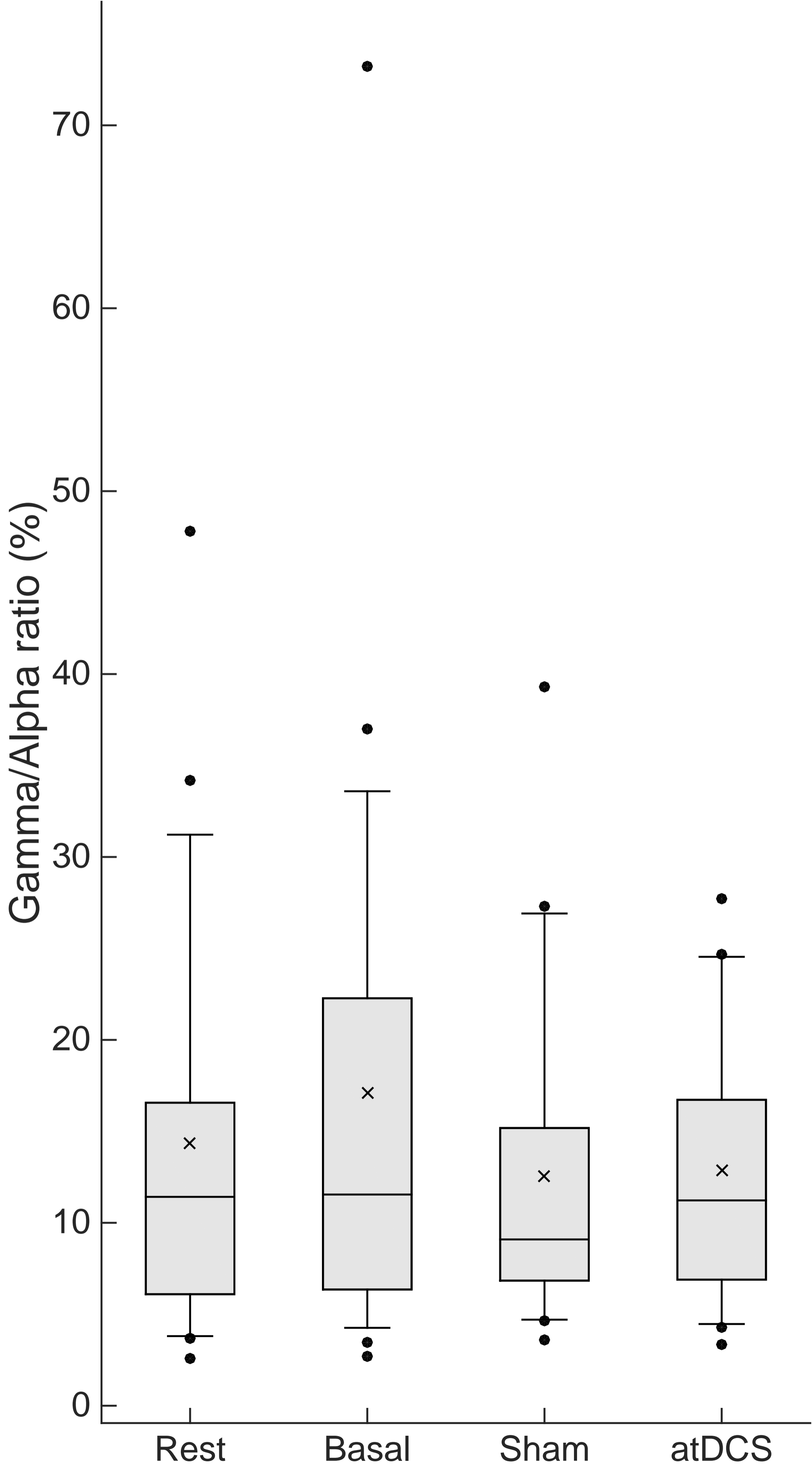

Supplement: Supplementary file 1 [file Data_Sheet_1.zip › Complementary_results/Band_ratios_average_PSD_windows/Gamma_Alpha/Gamma-Alpha_mean-win_F4.pdf]

**Gamma/Alpha ratio on average  
PSD windows for electrode: F7**

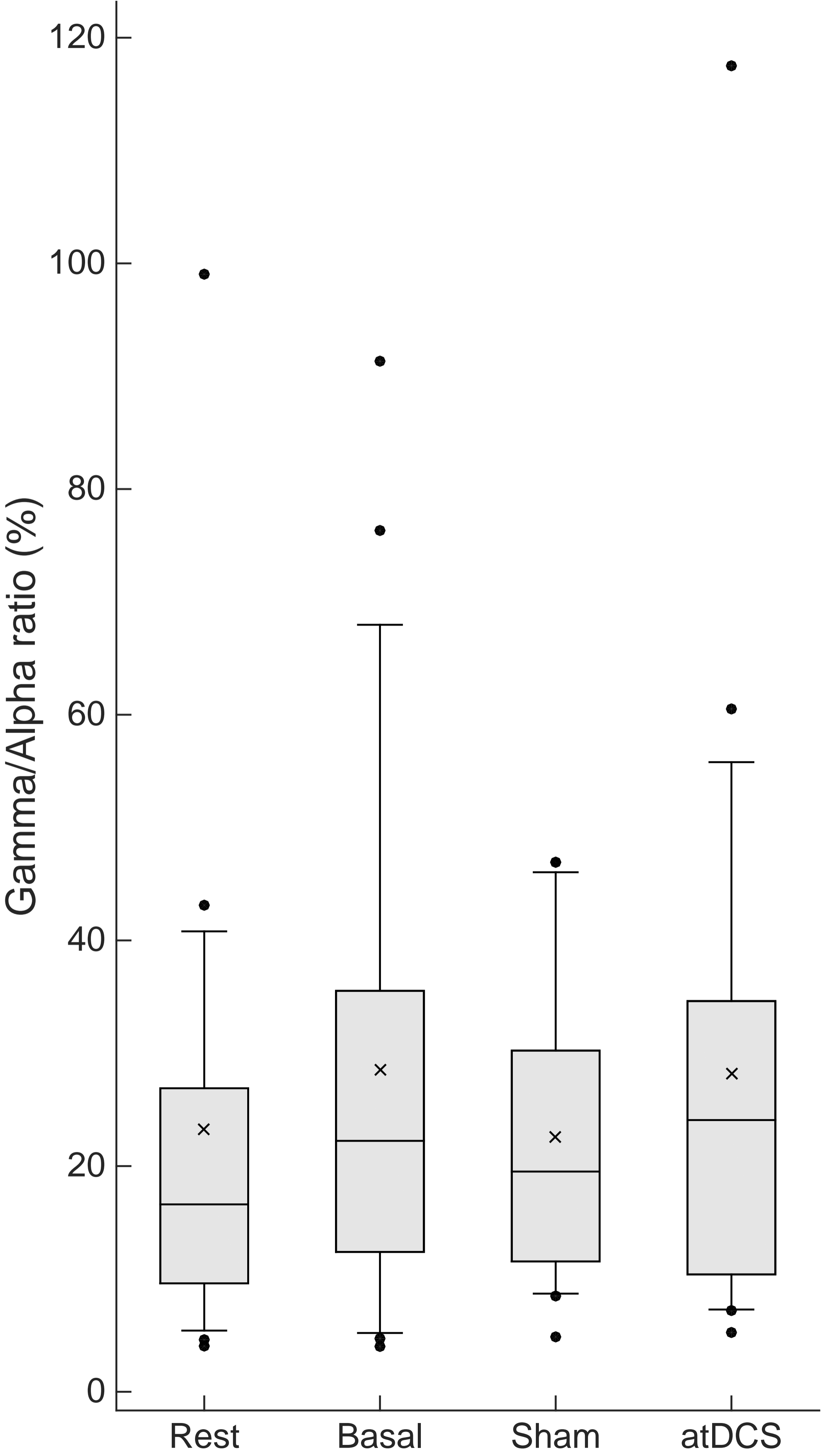

Supplement: Supplementary file 1 [file Data_Sheet_1.zip › Complementary_results/Band_ratios_average_PSD_windows/Gamma_Alpha/Gamma-Alpha_mean-win_F7.pdf]

**Gamma/Alpha ratio on average  
PSD windows for electrode: F8**

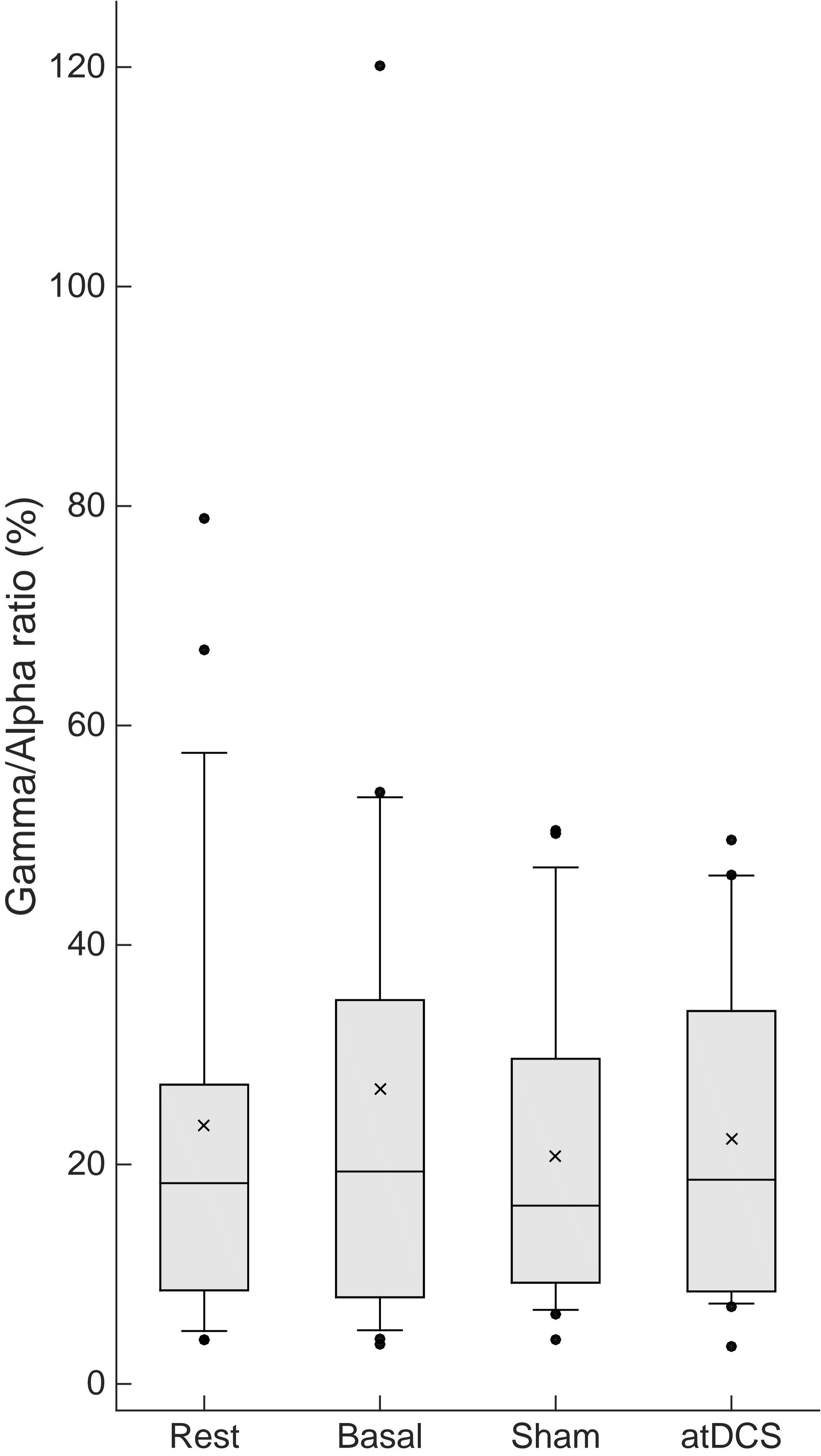

Supplement: Supplementary file 1 [file Data_Sheet_1.zip › Complementary_results/Band_ratios_average_PSD_windows/Gamma_Alpha/Gamma-Alpha_mean-win_F8.pdf]

**Gamma/Alpha ratio on average  
PSD windows for electrode: FC5**

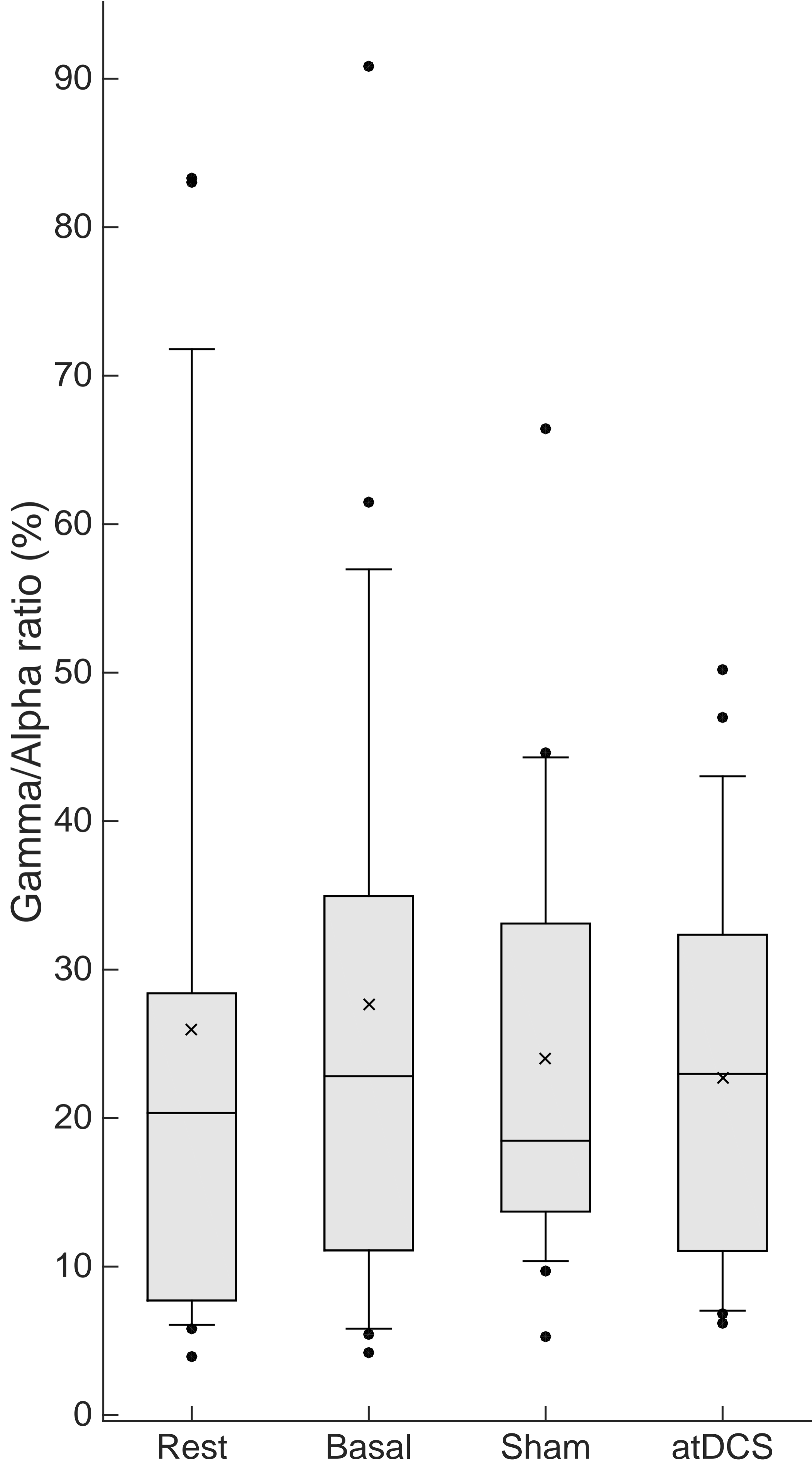

Supplement: Supplementary file 1 [file Data_Sheet_1.zip › Complementary_results/Band_ratios_average_PSD_windows/Gamma_Alpha/Gamma-Alpha_mean-win_FC5.pdf]

**Gamma/Alpha ratio on average  
PSD windows for electrode: FC6**

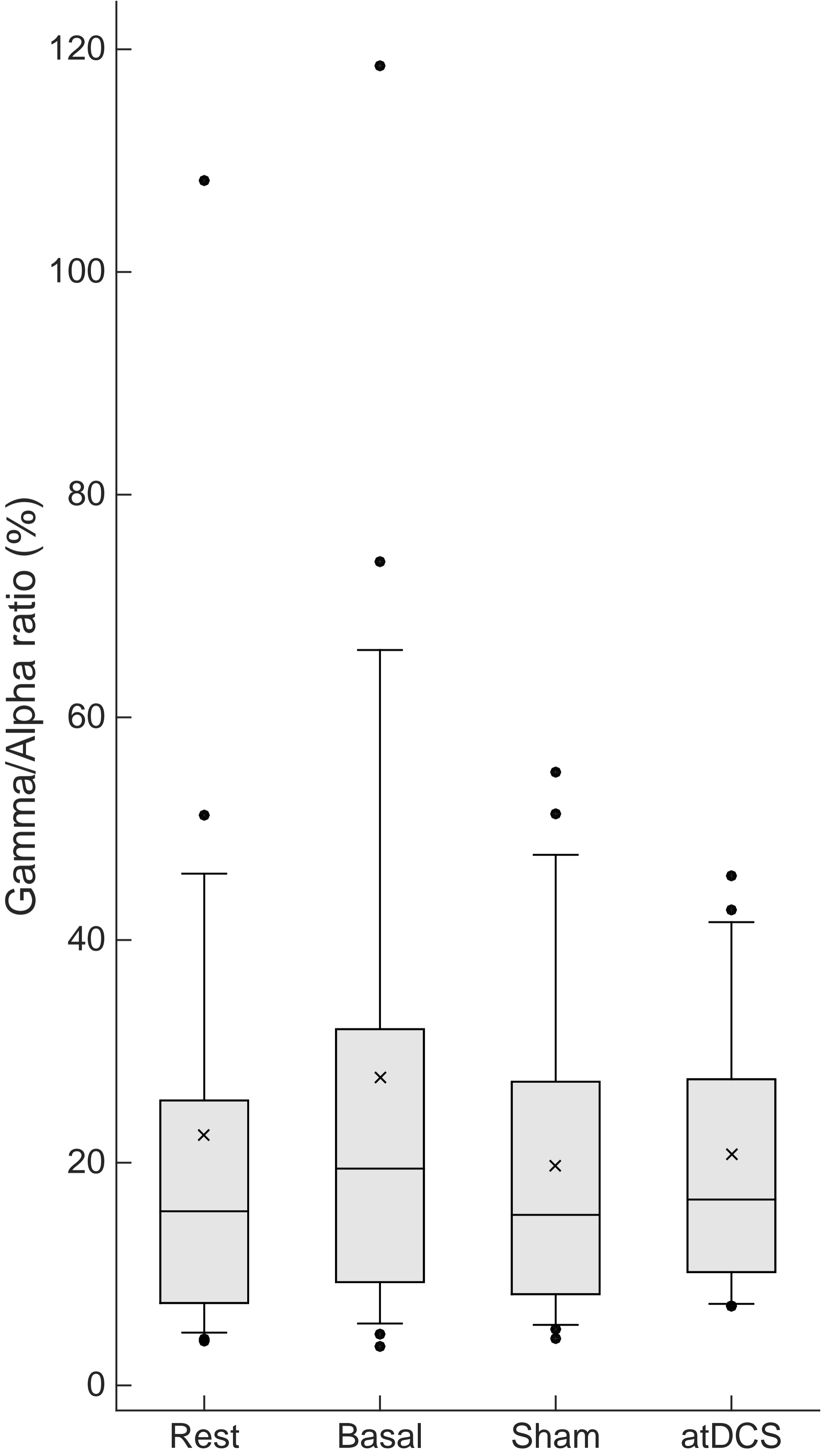

Supplement: Supplementary file 1 [file Data_Sheet_1.zip › Complementary_results/Band_ratios_average_PSD_windows/Gamma_Alpha/Gamma-Alpha_mean-win_FC6.pdf]

**Gamma/Alpha ratio on average  
PSD windows for electrode: O1**

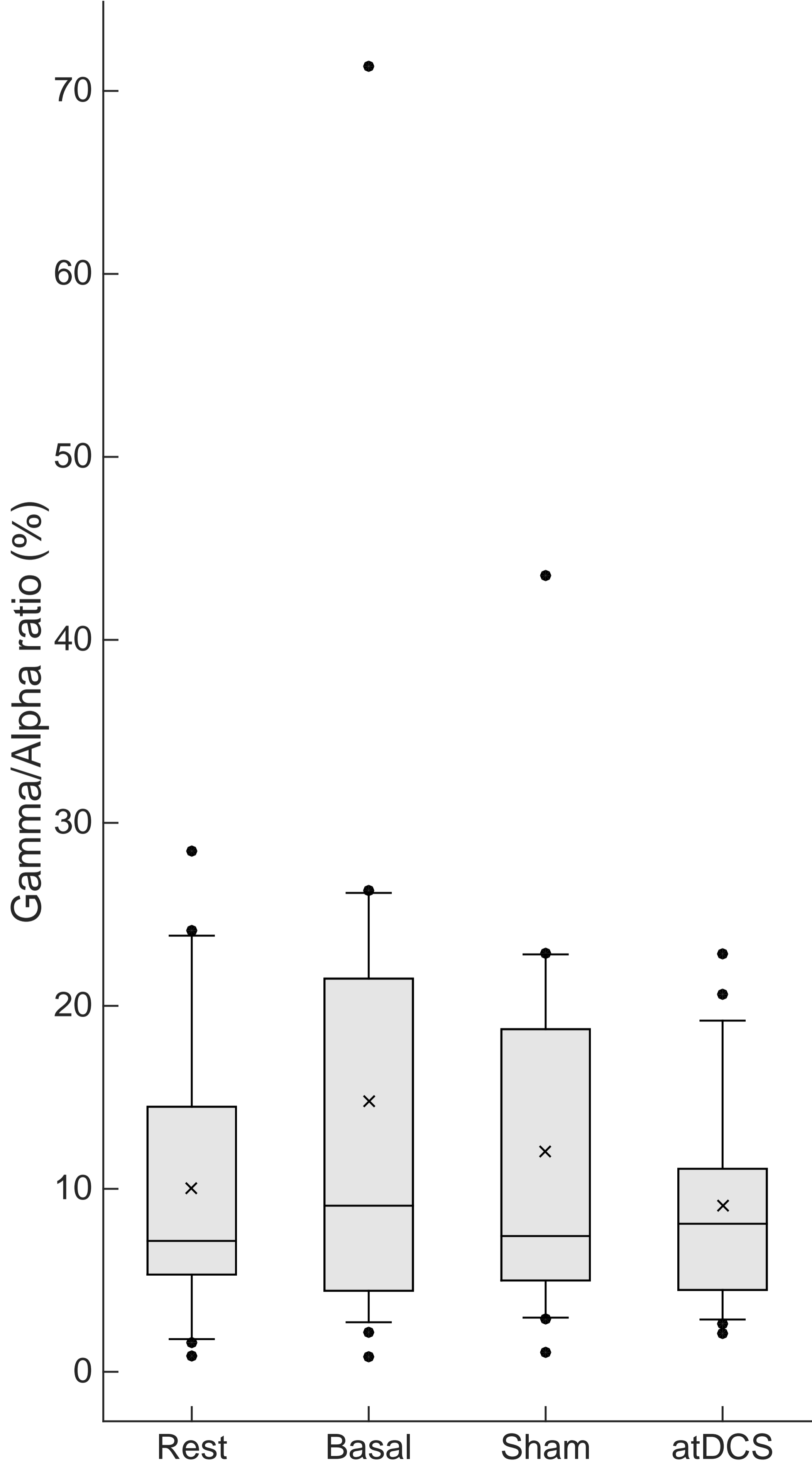

Supplement: Supplementary file 1 [file Data_Sheet_1.zip › Complementary_results/Band_ratios_average_PSD_windows/Gamma_Alpha/Gamma-Alpha_mean-win_O1.pdf]

**Gamma/Alpha ratio on average  
PSD windows for electrode: O2**

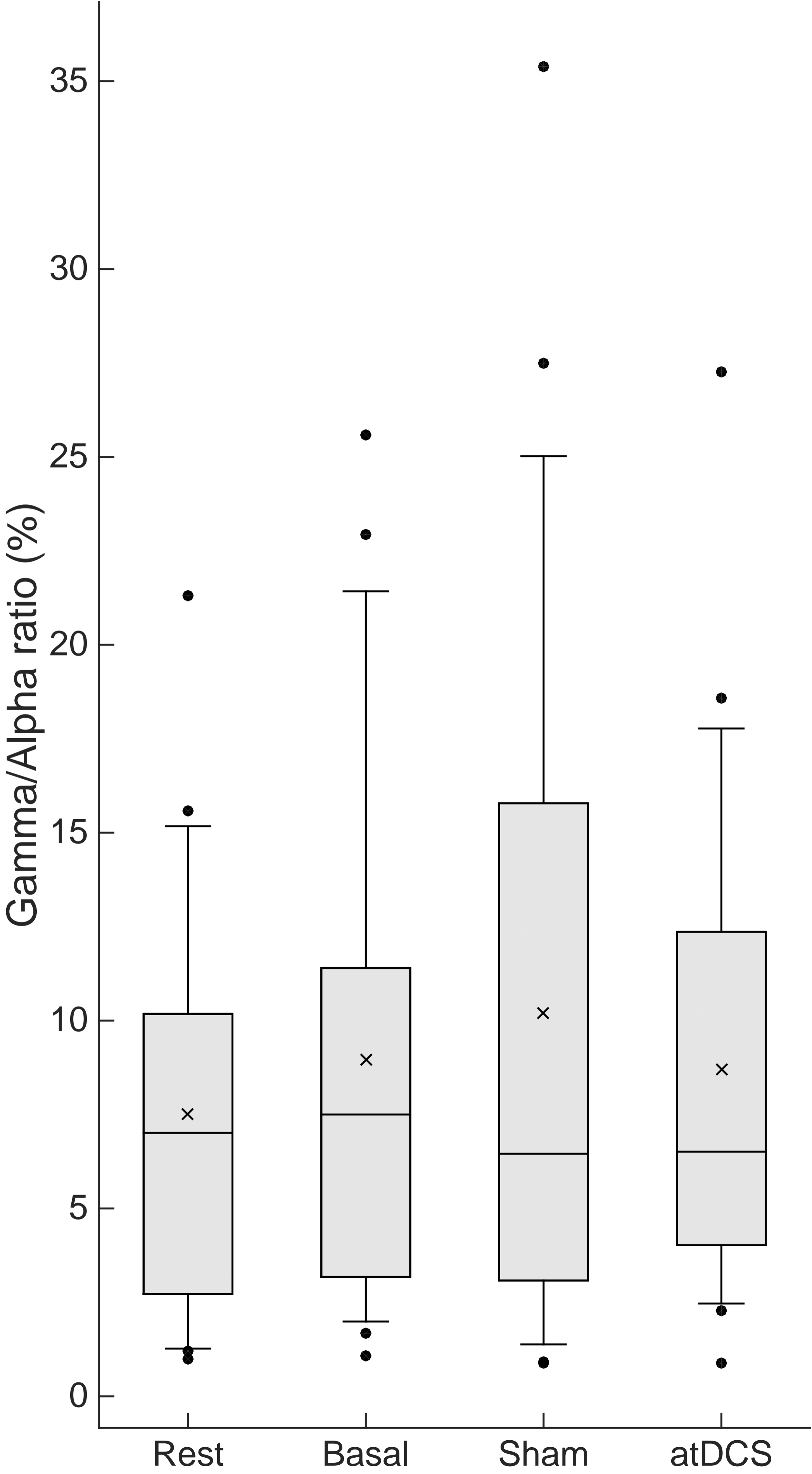

Supplement: Supplementary file 1 [file Data_Sheet_1.zip › Complementary_results/Band_ratios_average_PSD_windows/Gamma_Alpha/Gamma-Alpha_mean-win_O2.pdf]

**Gamma/Alpha ratio on average  
PSD windows for electrode: P7**

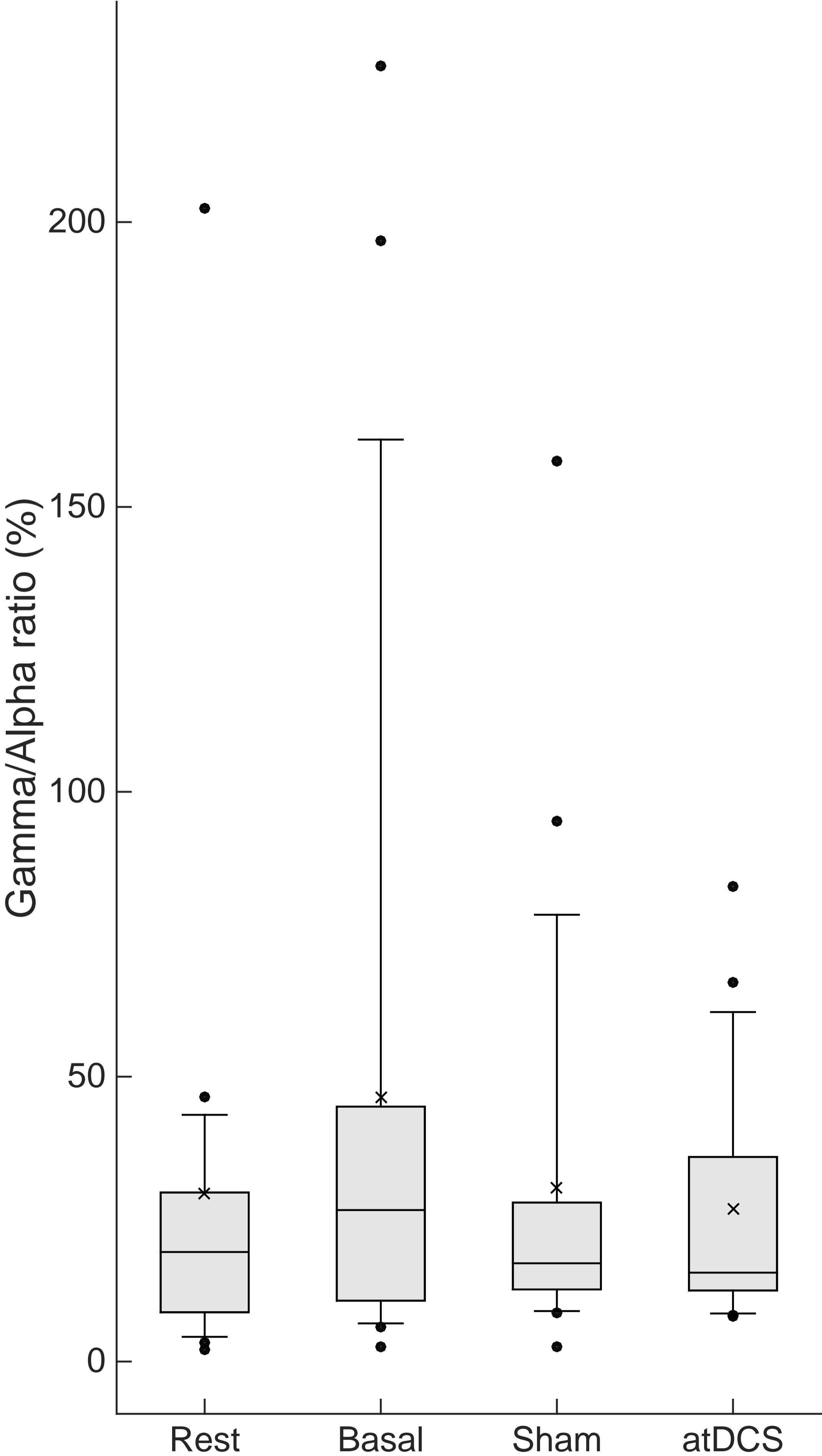

Supplement: Supplementary file 1 [file Data_Sheet_1.zip › Complementary_results/Band_ratios_average_PSD_windows/Gamma_Alpha/Gamma-Alpha_mean-win_P7.pdf]

**Gamma/Alpha ratio on average  
PSD windows for electrode: P8**

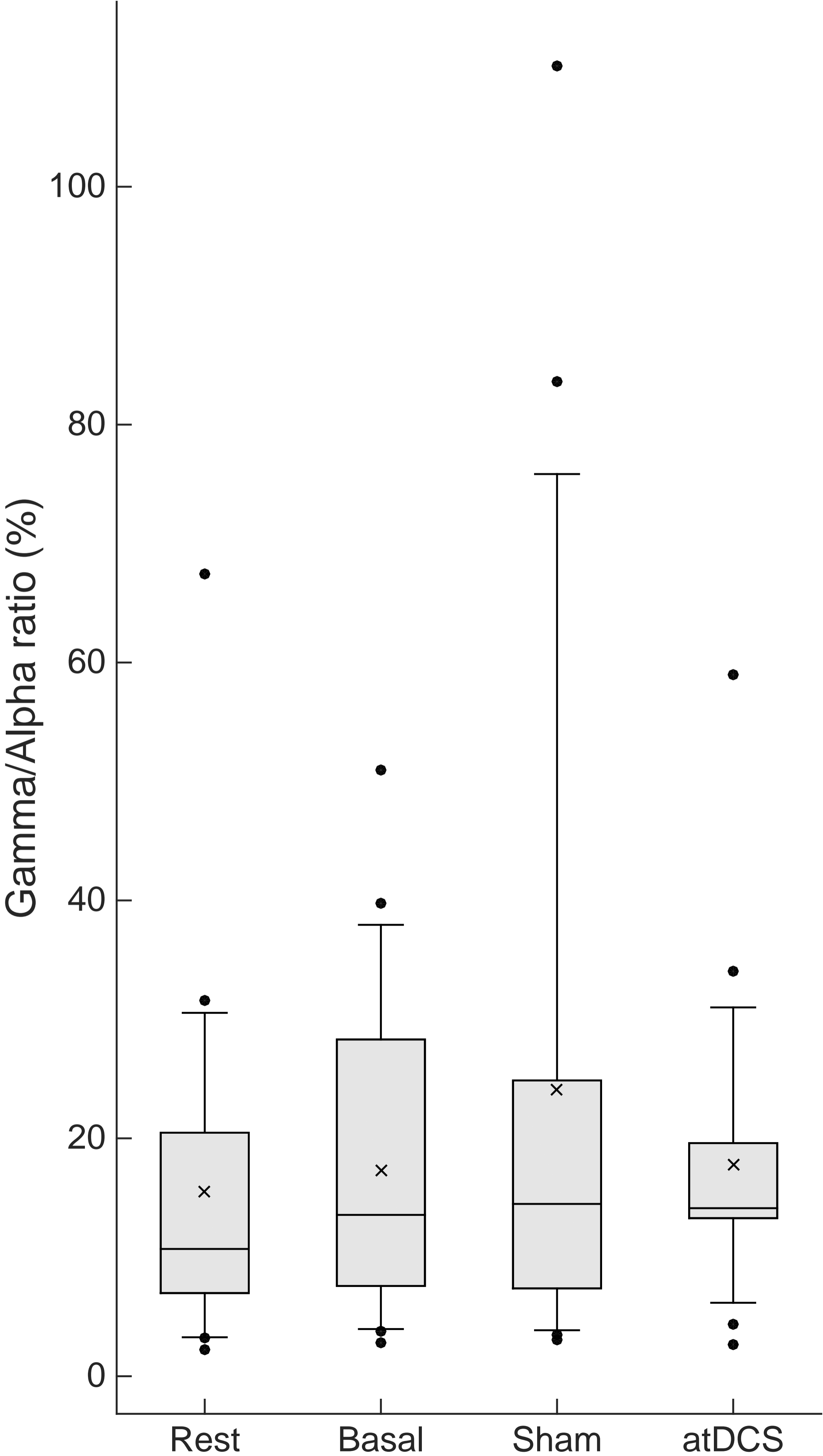

Supplement: Supplementary file 1 [file Data_Sheet_1.zip › Complementary_results/Band_ratios_average_PSD_windows/Gamma_Alpha/Gamma-Alpha_mean-win_P8.pdf]

**Gamma/Alpha ratio on average  
PSD windows for electrode: T7**

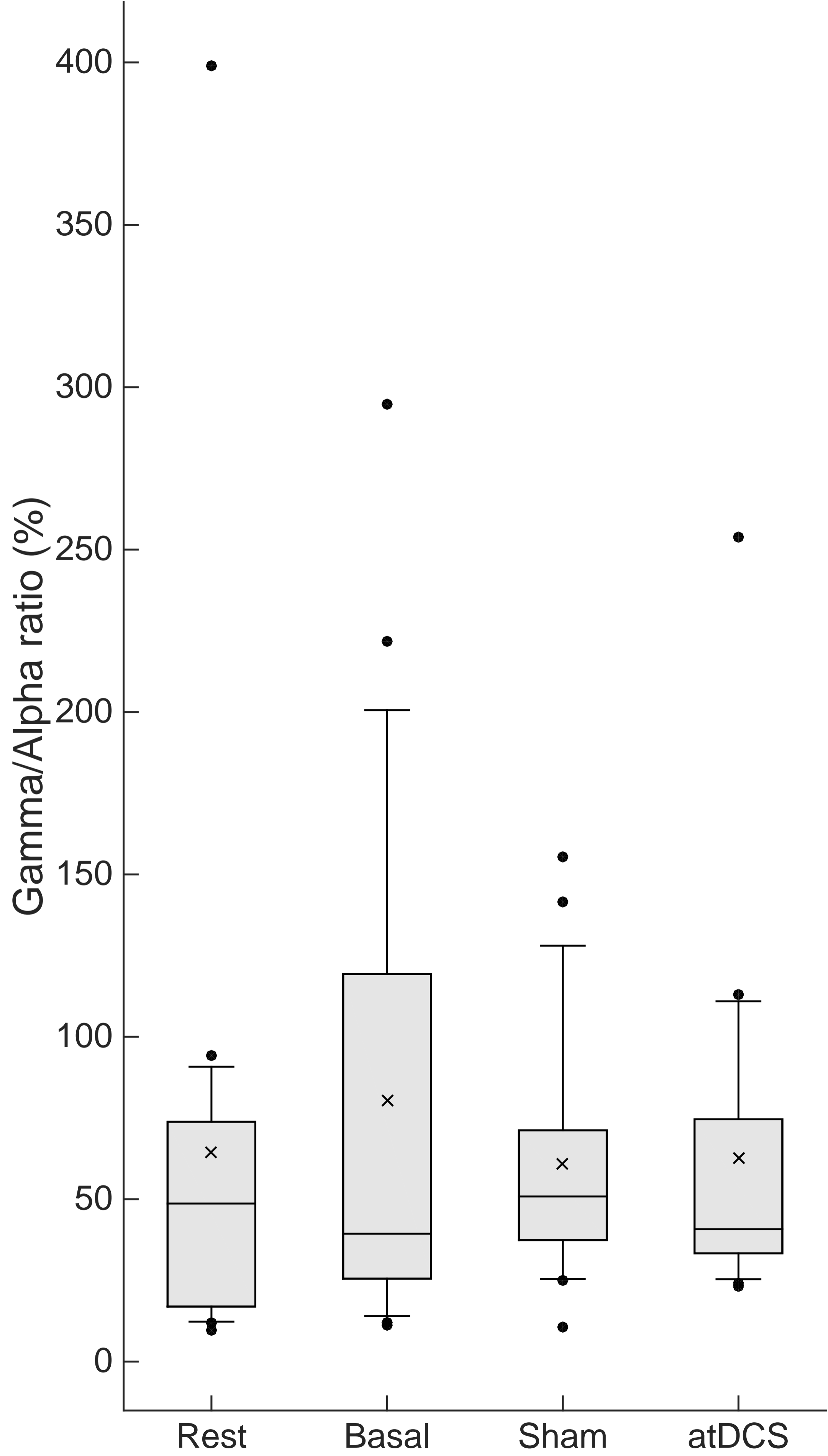

Supplement: Supplementary file 1 [file Data_Sheet_1.zip › Complementary_results/Band_ratios_average_PSD_windows/Gamma_Alpha/Gamma-Alpha_mean-win_T7.pdf]

**Gamma/Alpha ratio on average  
PSD windows for electrode: T8**

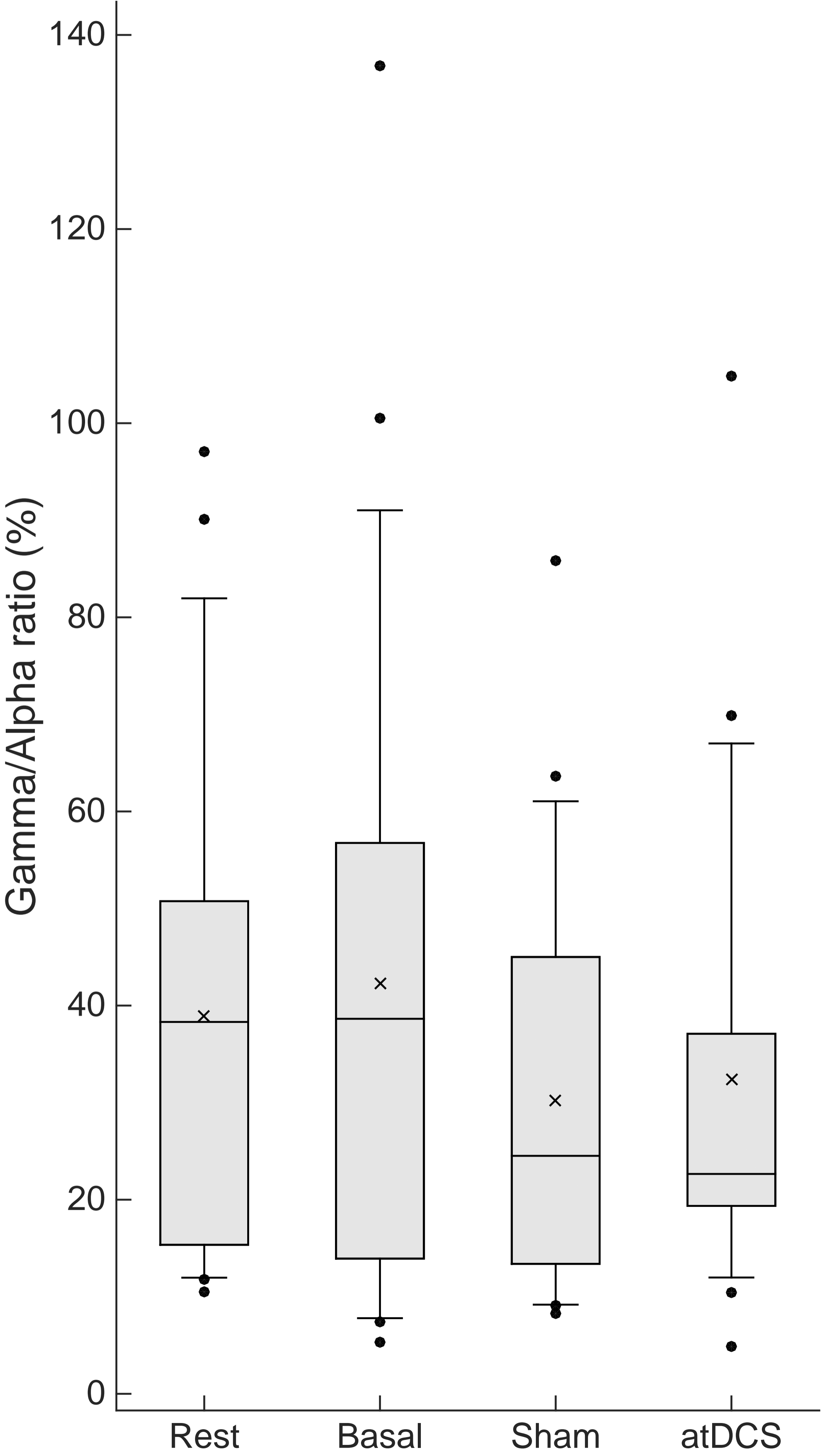

Supplement: Supplementary file 1 [file Data_Sheet_1.zip › Complementary_results/Band_ratios_average_PSD_windows/Gamma_Alpha/Gamma-Alpha_mean-win_T8.pdf]

**Gamma/Delta ratio on average  
PSD windows for electrode: AF3**

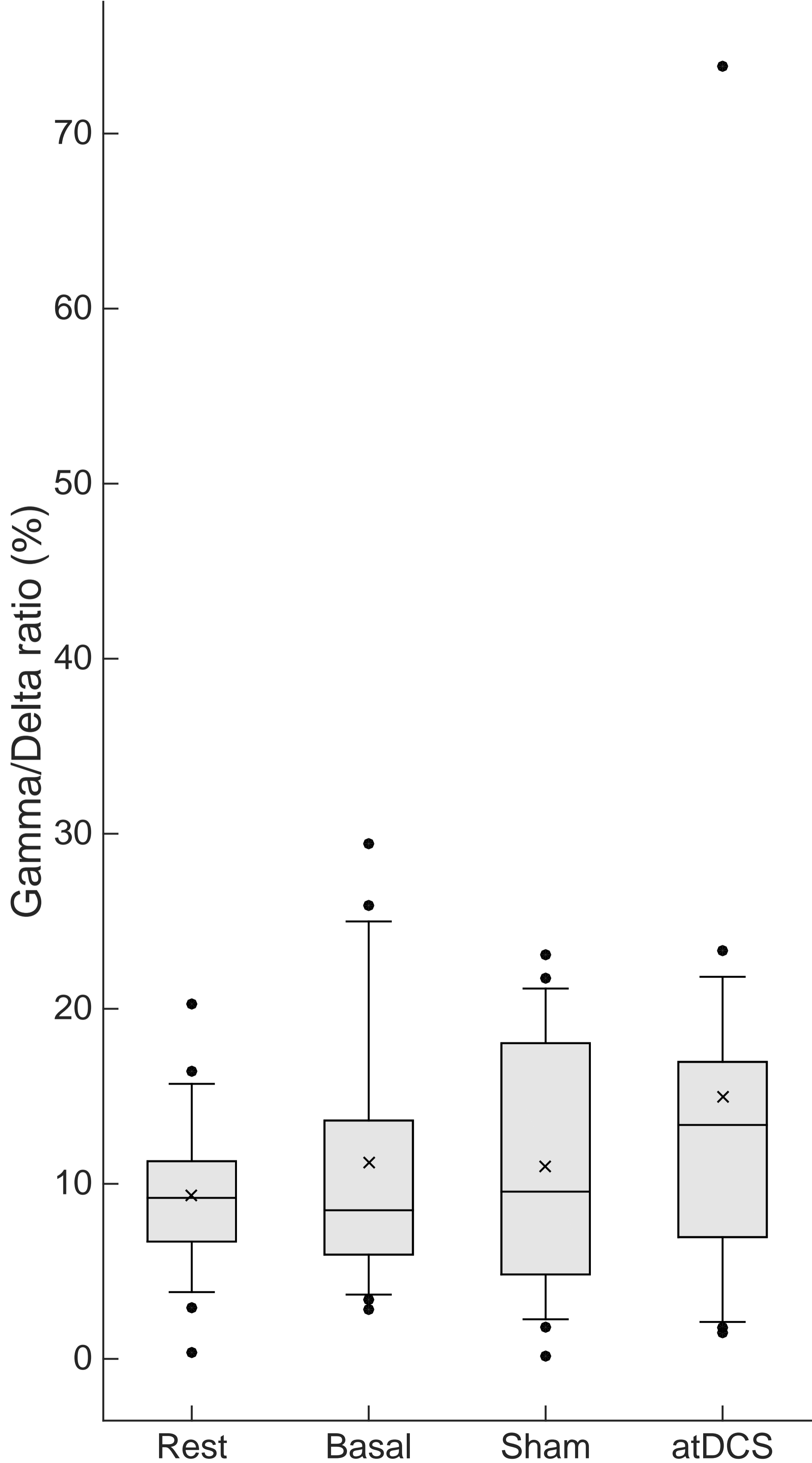

Supplement: Supplementary file 1 [file Data_Sheet_1.zip › Complementary_results/Band_ratios_average_PSD_windows/Gamma_Delta/Gamma-Delta_mean-win_AF3.pdf]

**Gamma/Delta ratio on average  
PSD windows for electrode: AF4**

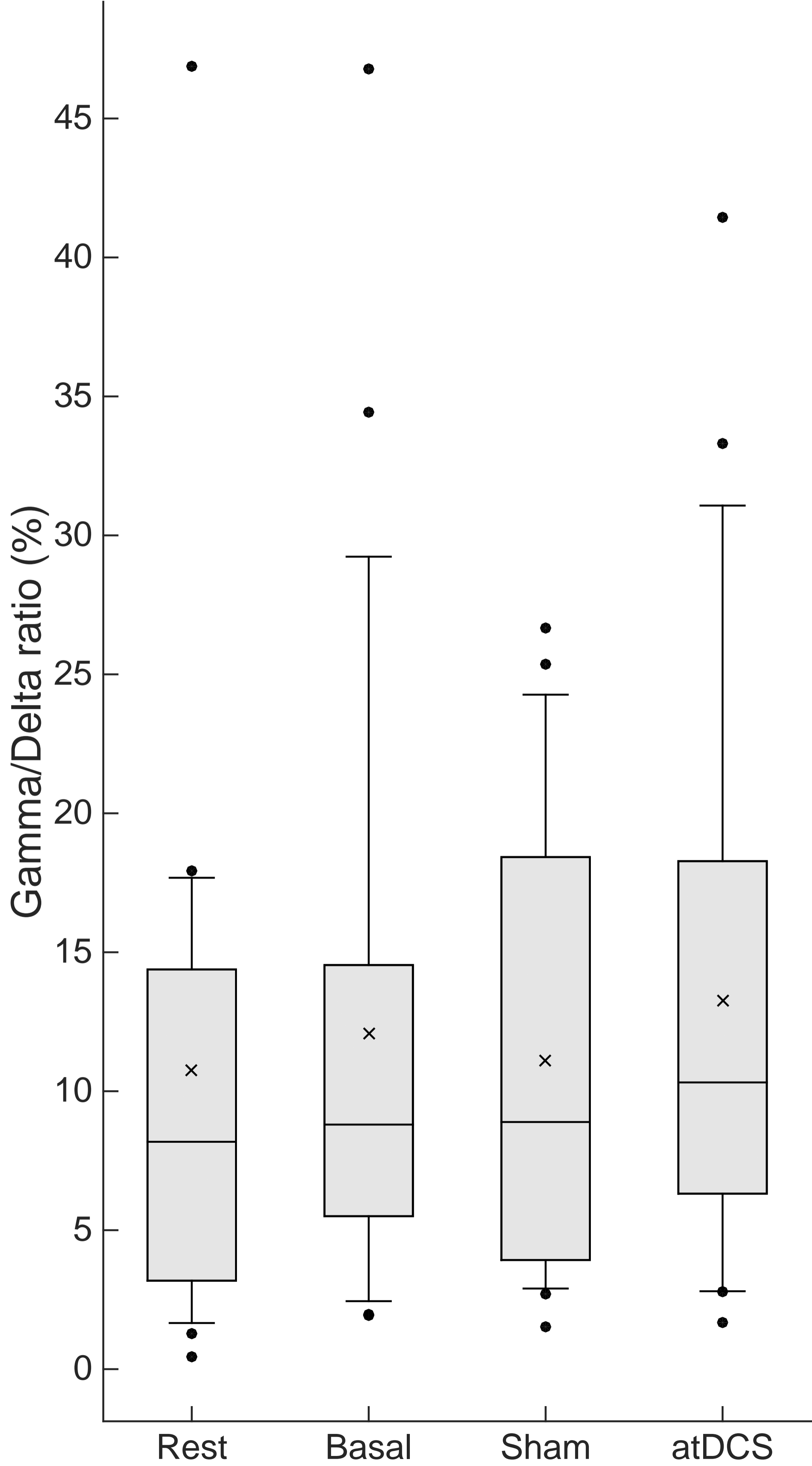

Supplement: Supplementary file 1 [file Data_Sheet_1.zip › Complementary_results/Band_ratios_average_PSD_windows/Gamma_Delta/Gamma-Delta_mean-win_AF4.pdf]

**Gamma/Delta ratio on average  
PSD windows for electrode: Avg AF3-F3-F7**

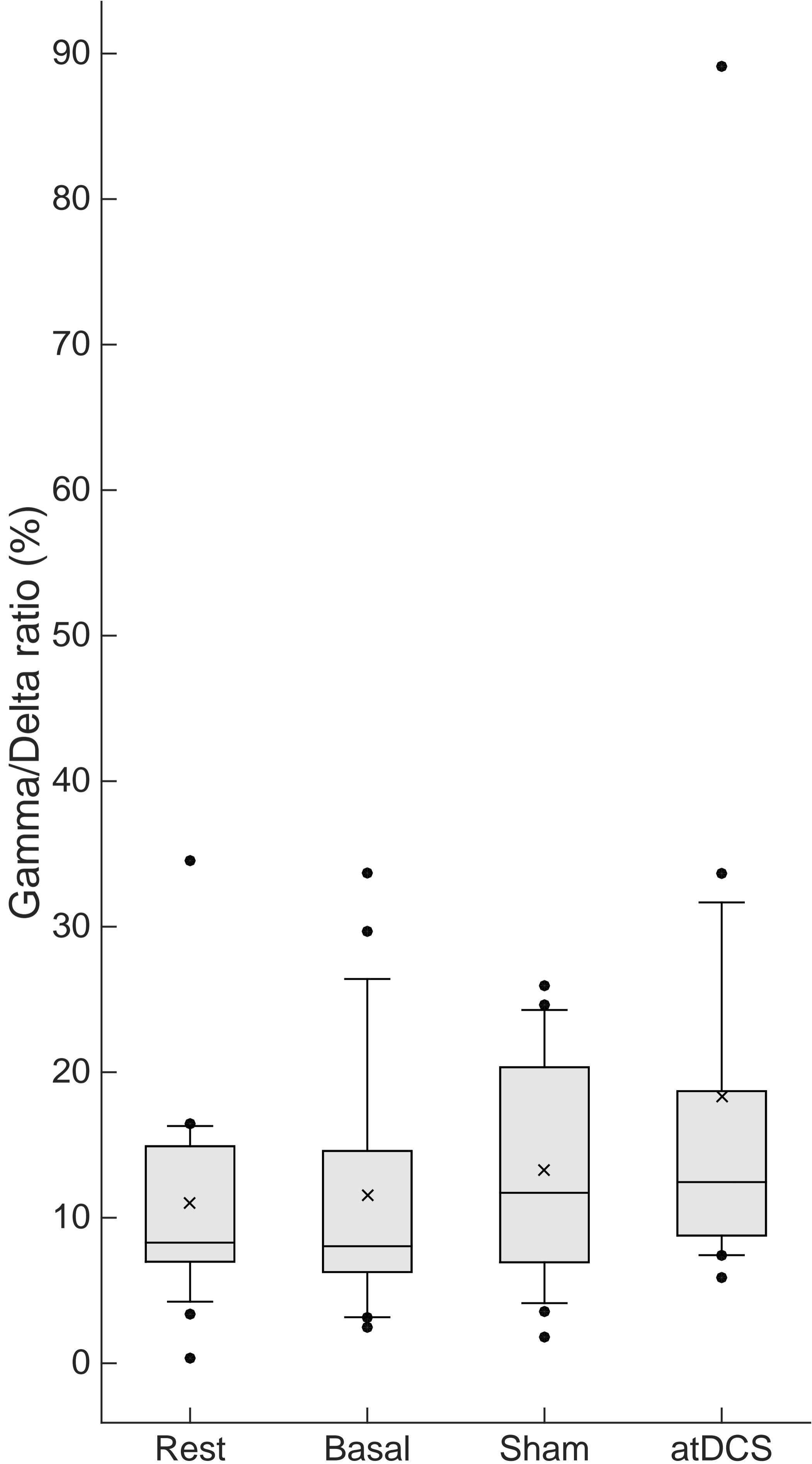

Supplement: Supplementary file 1 [file Data_Sheet_1.zip › Complementary_results/Band_ratios_average_PSD_windows/Gamma_Delta/Gamma-Delta_mean-win_Avg AF3-F3-F7.pdf]

**Gamma/Delta ratio on average**  
**PSD windows for electrode: Avg AF4-F4-F8**

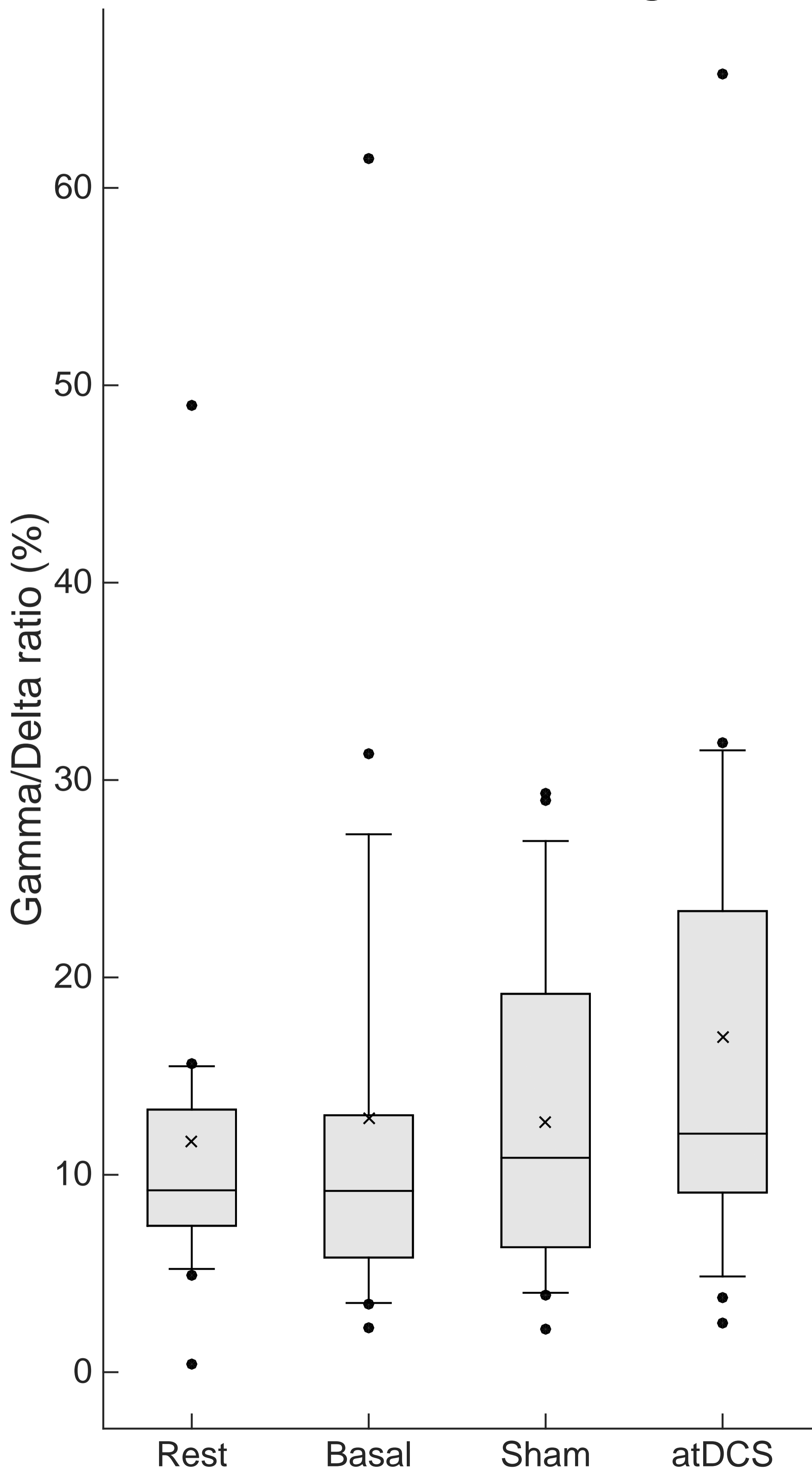

Supplement: Supplementary file 1 [file Data_Sheet_1.zip › Complementary_results/Band_ratios_average_PSD_windows/Gamma_Delta/Gamma-Delta_mean-win_Avg AF4-F4-F8.pdf]

**Gamma/Delta ratio on average**  
**PSD windows for electrode: Avg F3-F7-FC5**

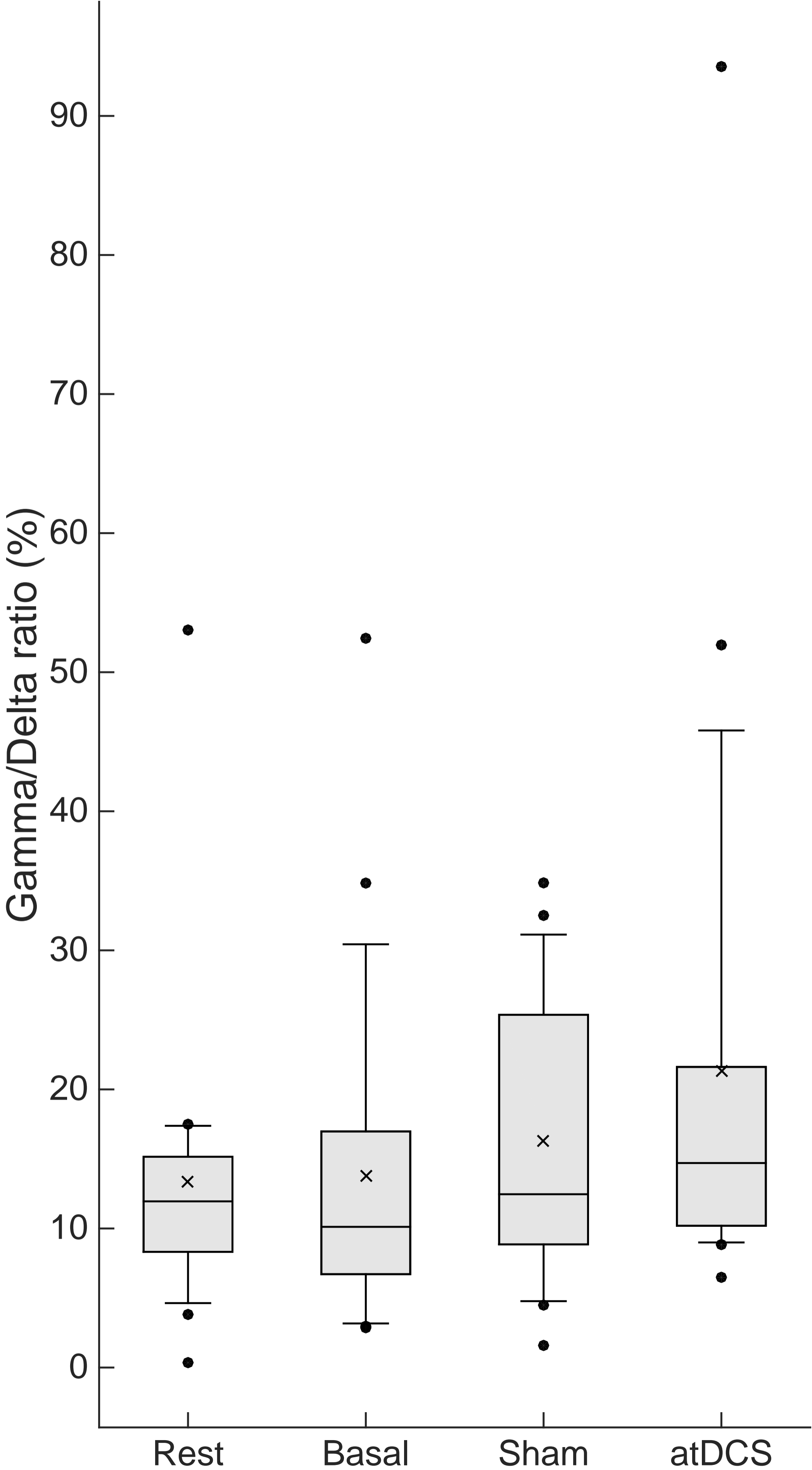

Supplement: Supplementary file 1 [file Data_Sheet_1.zip › Complementary_results/Band_ratios_average_PSD_windows/Gamma_Delta/Gamma-Delta_mean-win_Avg F3-F7-FC5.pdf]

**Gamma/Delta ratio on average**  
**PSD windows for electrode: Avg F4-F8-FC6**

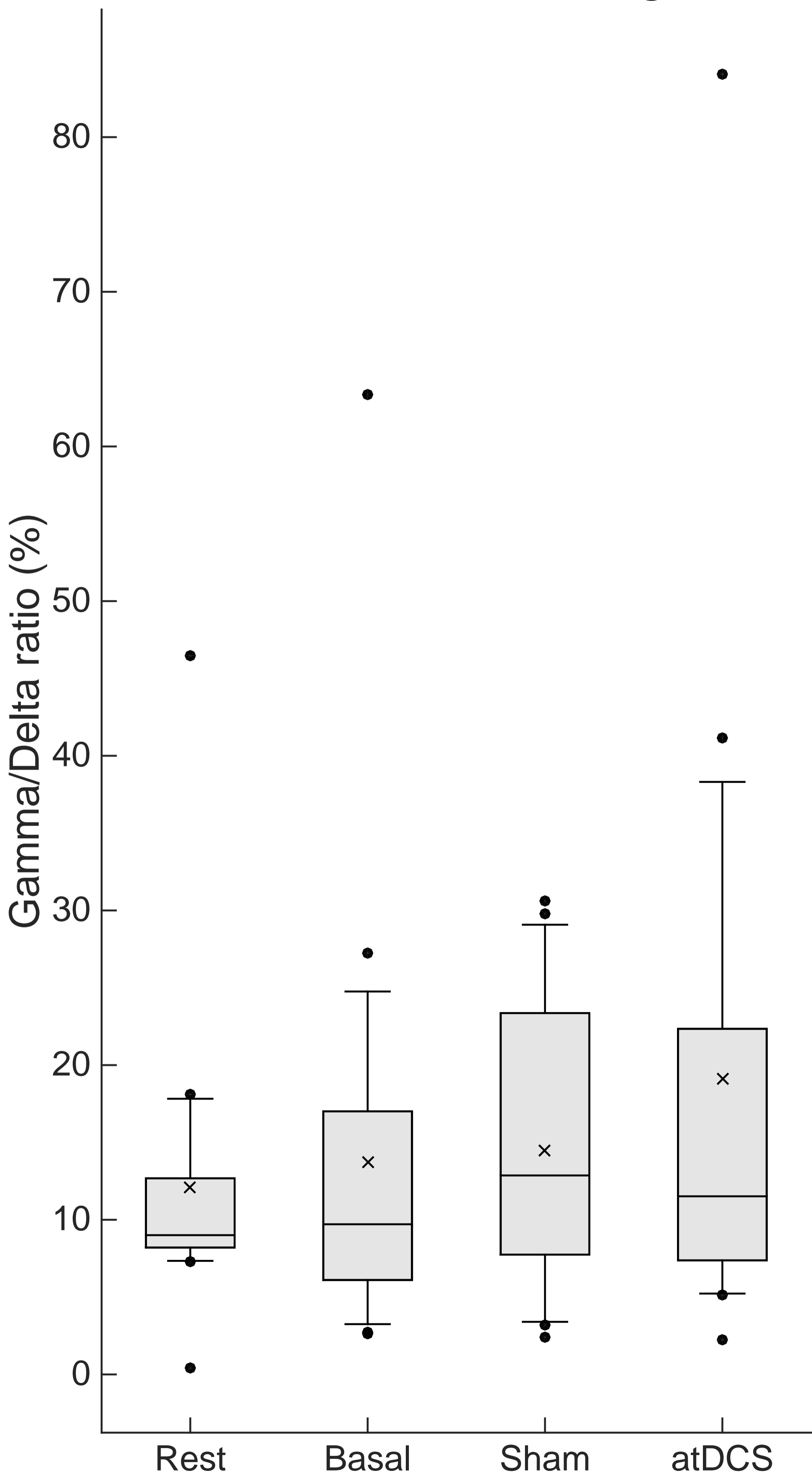

Supplement: Supplementary file 1 [file Data_Sheet_1.zip › Complementary_results/Band_ratios_average_PSD_windows/Gamma_Delta/Gamma-Delta_mean-win_Avg F4-F8-FC6.pdf]

**Gamma/Delta ratio on average  
PSD windows for electrode: F3**

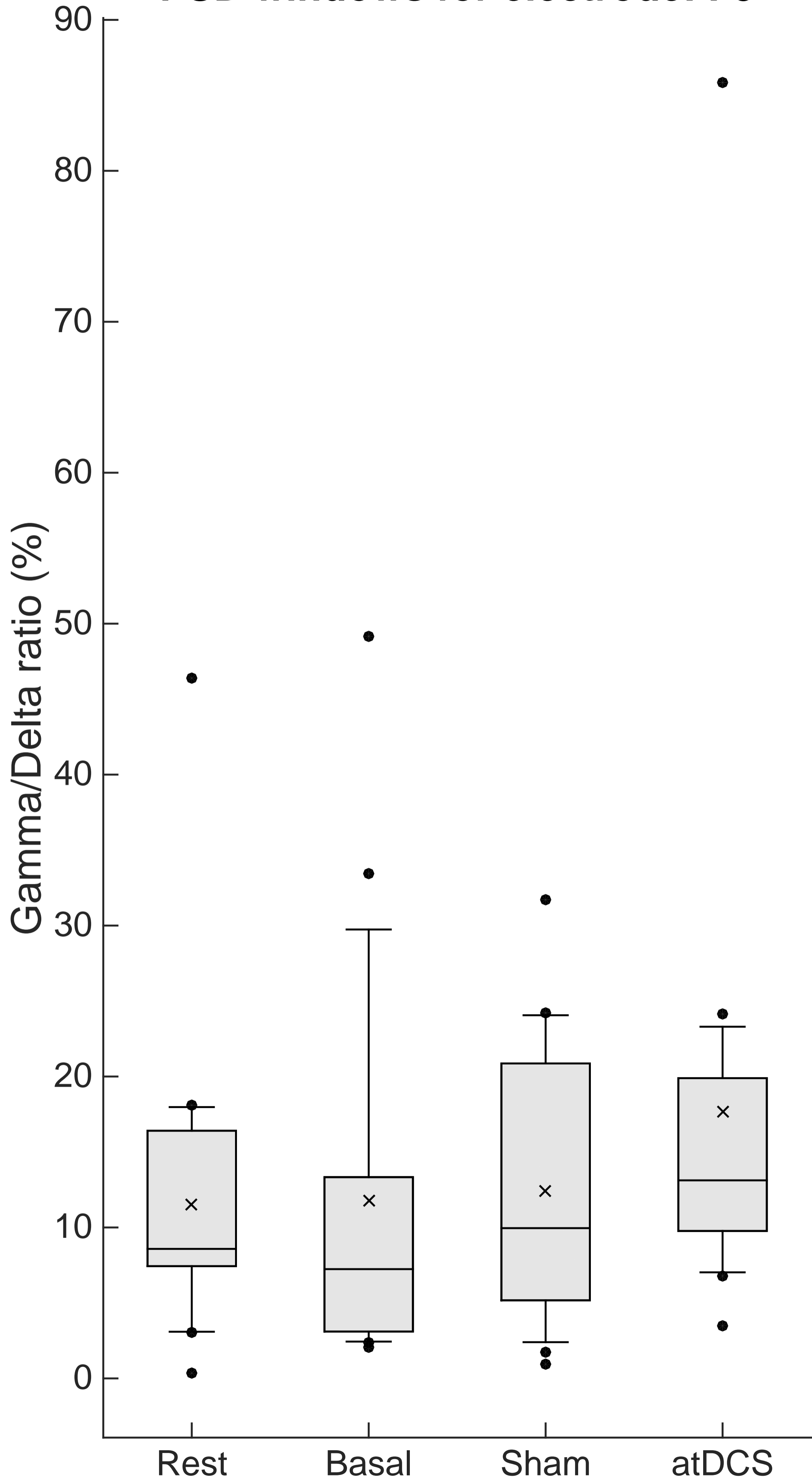

Supplement: Supplementary file 1 [file Data_Sheet_1.zip › Complementary_results/Band_ratios_average_PSD_windows/Gamma_Delta/Gamma-Delta_mean-win_F3.pdf]

**Gamma/Delta ratio on average  
PSD windows for electrode: F4**

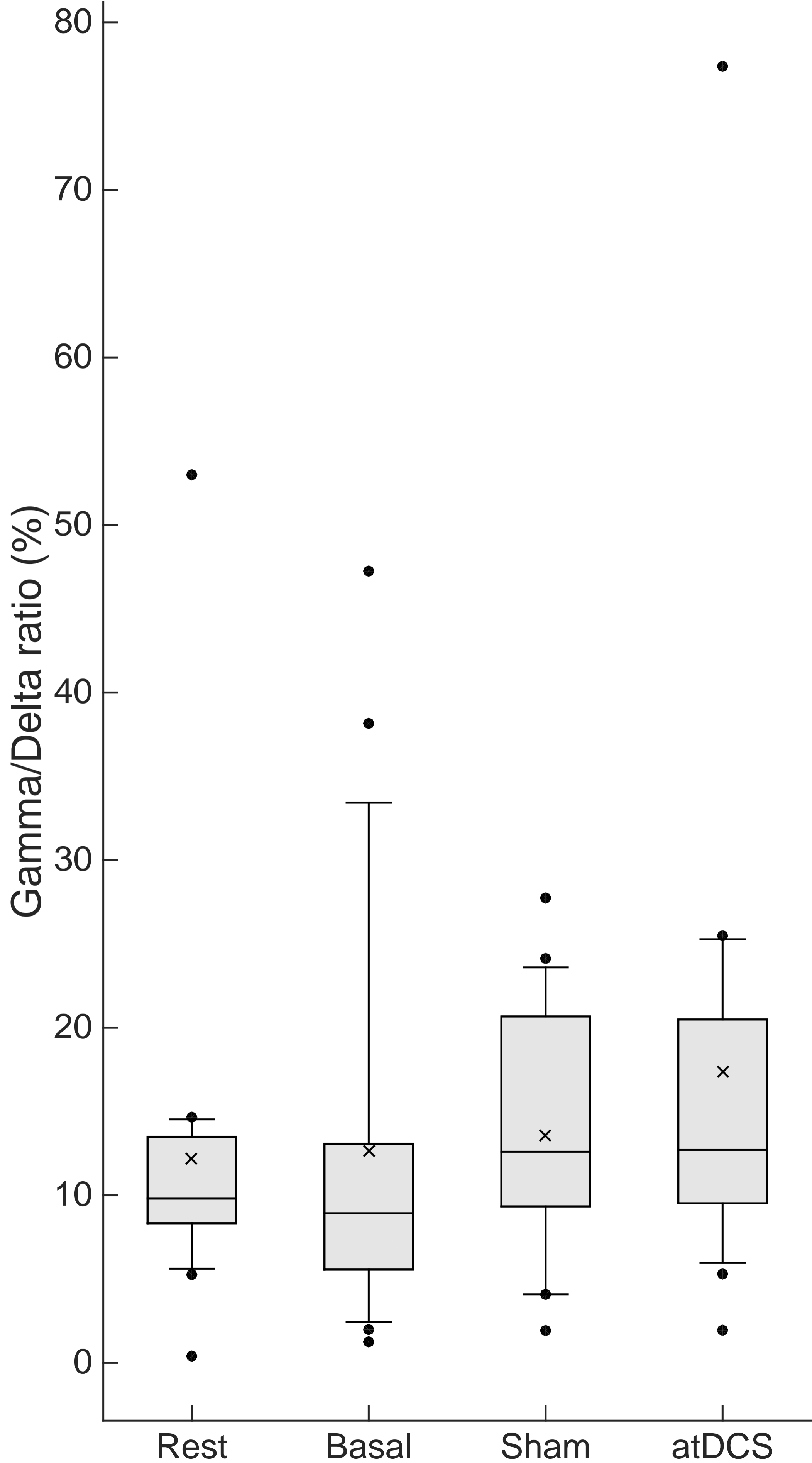

Supplement: Supplementary file 1 [file Data_Sheet_1.zip › Complementary_results/Band_ratios_average_PSD_windows/Gamma_Delta/Gamma-Delta_mean-win_F4.pdf]

**Gamma/Delta ratio on average  
PSD windows for electrode: F7**

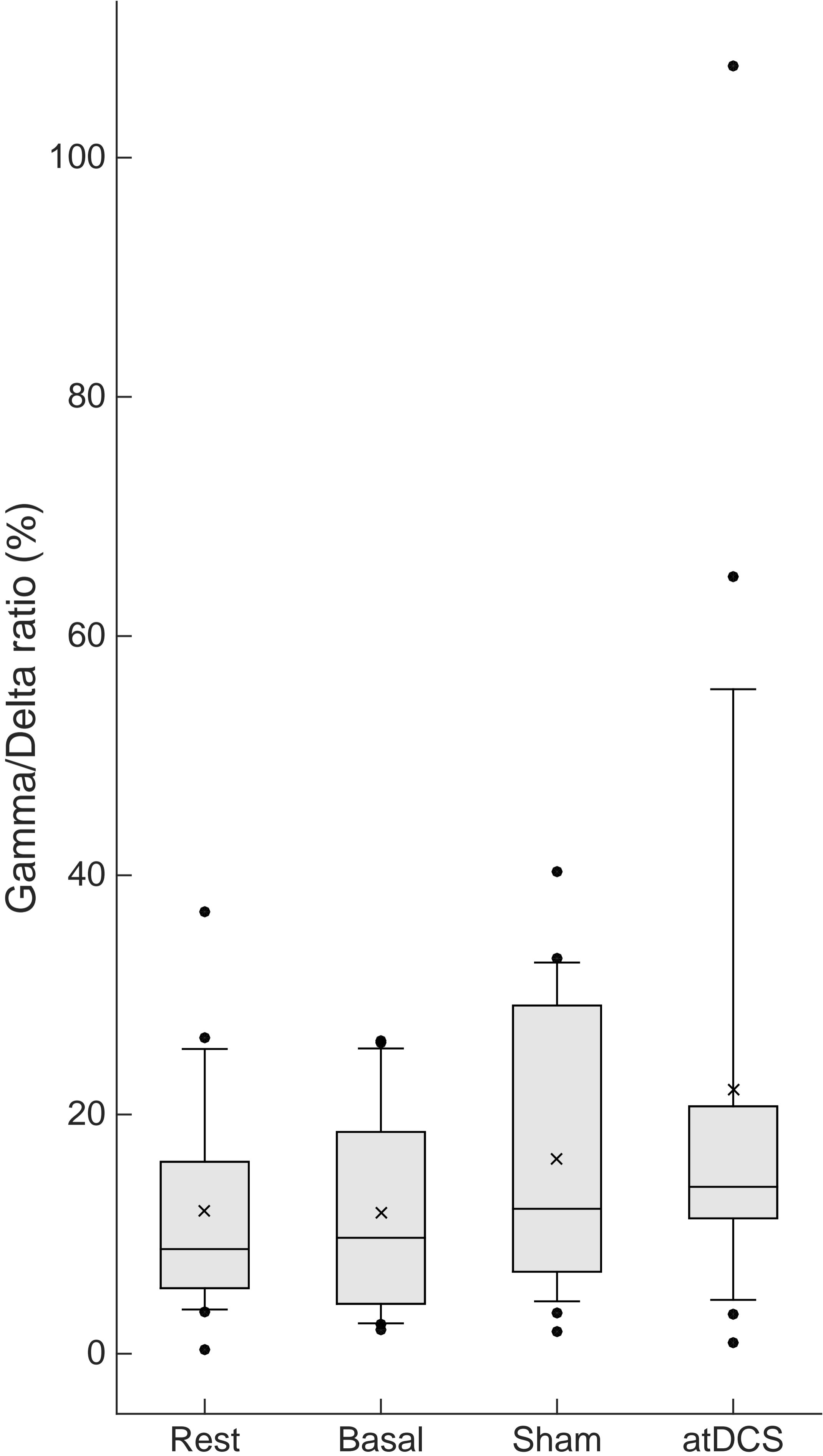

Supplement: Supplementary file 1 [file Data_Sheet_1.zip › Complementary_results/Band_ratios_average_PSD_windows/Gamma_Delta/Gamma-Delta_mean-win_F7.pdf]

**Gamma/Delta ratio on average  
PSD windows for electrode: F8**

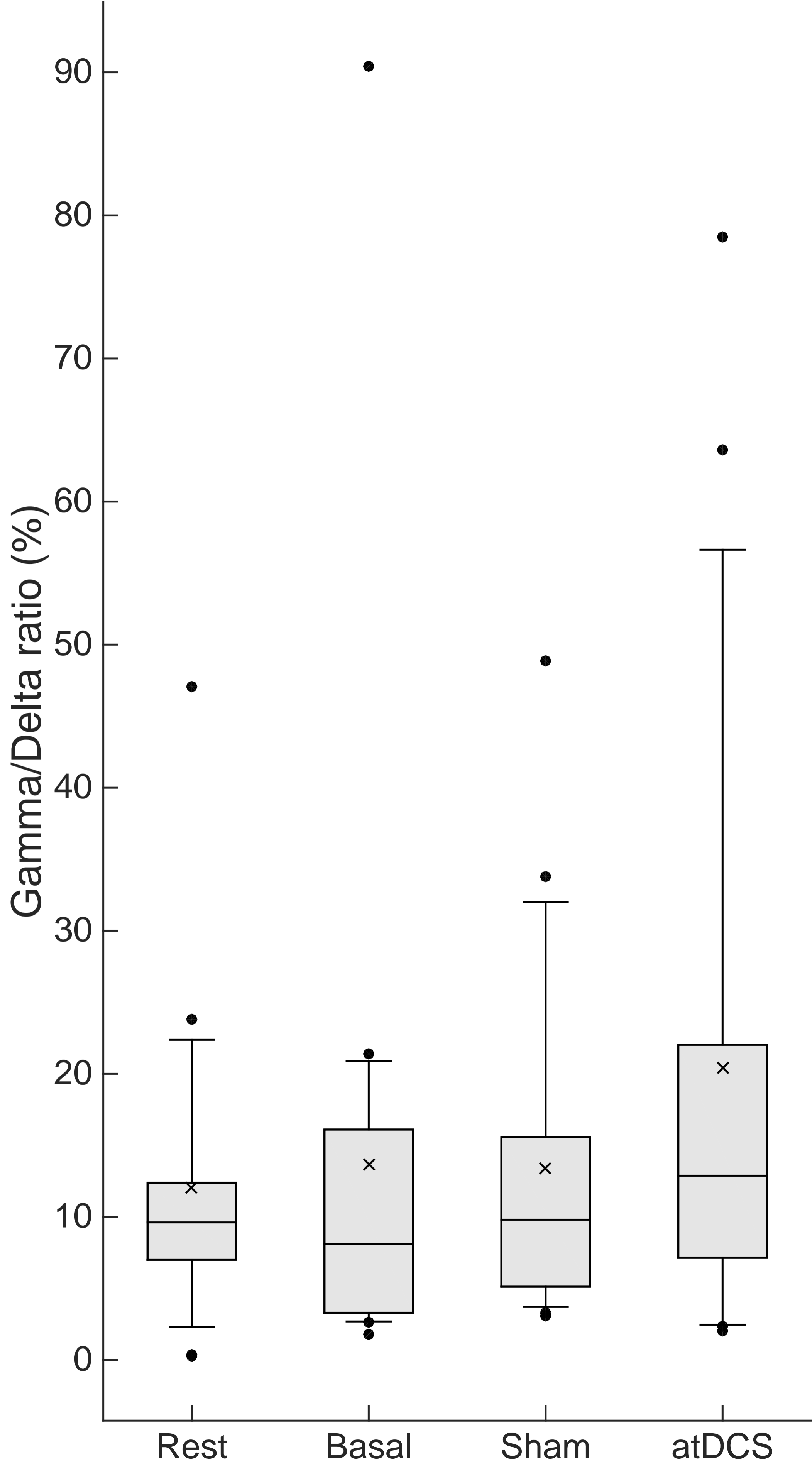

Supplement: Supplementary file 1 [file Data_Sheet_1.zip › Complementary_results/Band_ratios_average_PSD_windows/Gamma_Delta/Gamma-Delta_mean-win_F8.pdf]
